# Construction of JRG (Japanese reference genome) with single-molecule real-time sequencing

**DOI:** 10.1038/s41439-019-0057-7

**Published:** 2019-06-07

**Authors:** Masao Nagasaki, Yoko Kuroki, Tomoko F. Shibata, Fumiki Katsuoka, Takahiro Mimori, Yosuke Kawai, Naoko Minegishi, Atsushi Hozawa, Shinichi Kuriyama, Yoichi Suzuki, Hiroshi Kawame, Fuji Nagami, Takako Takai-Igarashi, Soichi Ogishima, Kaname Kojima, Kazuharu Misawa, Osamu Tanabe, Nobuo Fuse, Hiroshi Tanaka, Nobuo Yaegashi, Kengo Kinoshita, Shiego Kure, Jun Yasuda, Masayuki Yamamoto

**Affiliations:** 1 Tohoku Medical Megabank Organization, Tohoku University, Sendai, Japan; 2 Graduate School of Medicine, Tohoku University, Sendai, Japan; 3 Graduate School of Information Sciences, Tohoku University, Sendai, Japan; 4 Department of Genome Medicine, National Center for Child Health and Development, Tokyo, Japan; 5 International Research Institute of Disaster Science, Tohoku University, Sendai, Japan; 6 Tohoku University Hospital, Tohoku University, Sendai, Japan

**Keywords:** Genomics, DNA sequencing

## Abstract

In recent genome analyses, population-specific reference panels have indicated important. However, reference panels based on short-read sequencing data do not sufficiently cover long insertions. Therefore, the nature of long insertions has not been well documented. Here, we assembled a Japanese genome using single-molecule real-time sequencing data and characterized insertions found in the assembled genome. We identified 3691 insertions ranging from 100 bps to ~10,000 bps in the assembled genome relative to the international reference sequence (GRCh38). To validate and characterize these insertions, we mapped short-reads from 1070 Japanese individuals and 728 individuals from eight other populations to insertions integrated into GRCh38. With this result, we constructed JRGv1 (Japanese Reference Genome version 1) by integrating the 903 verified insertions, totaling 1,086,173 bases, shared by at least two Japanese individuals into GRCh38. We also constructed decoyJRGv1 by concatenating 3559 verified insertions, totaling 2,536,870 bases, shared by at least two Japanese individuals or by six other assemblies. This assembly improved the alignment ratio by 0.4% on average. These results demonstrate the importance of refining the reference assembly and creating a population-specific reference genome. JRGv1 and decoyJRGv1 are available at the JRG website.

## Reference genome: Reading longer sequences improves Japanese reference

Researchers in Japan have assembled a Japanese reference genome, which includes sequences missing from the international reference genome, as well as others specific to East Asian populations. A team led by Masao Nagasaki and Masayuki Yamamoto sequenced a Japanese individual using a method, which produces longer sequences than previous technologies. Using this approach, they identified thousands of sequences spanning 2.5 million bases, which were absent in the international reference genome. Many of these were sequences able to move within the genome. They showed that the majority of these sequences are also present in early humans and chimpanzees, demonstrating that their absence from the current reference is due to deletions or limitations of earlier sequencing methodologies. In addition to providing a population-specific reference, these findings demonstrate the importance of continually improving the international reference genome.

## Introduction

Since completion of the Human Genome Project^1,2^, the international reference genome has been continuously improved, facilitating the analysis of variations in accessible regions of the human genome. Furthermore, the development of second-generation sequencers^3–5^ has enabled large-scale genome analysis and allowed the construction of population-specific reference panels^6–10^. These efforts are expected to contribute to the realization of precision medicine, which considers differences in individual genetic backgrounds^11^.

To develop precision medicine in Japan, we conducted high-coverage whole-genome sequencing of 1070 Japanese individuals and constructed the 1 K Japanese population reference panel (1KJPN) cataloging ~25 million variants, including single-nucleotide variants (SNVs), short insertions, and deletions^12^. The 1KJPN datasets have been utilized for various genome analyses, such as accurate genotype imputation in genome-wide association studies. However, it should be noted that 1KJPN is based on short-read sequencing data and does not cover long insertions, while long deletions have been detected. In short-read sequencing, variant analysis is performed using a genome resequencing approach in which millions of reads are aligned to an international reference genome and variants are detected by comparing sequences against a reference genome. This approach can effectively detect the deletions, single-nucleotide variants, and short insertions compared to the reference genome in each individual but is less effective at detecting long insertions (more than 100 bases) and other complicated structural variants.

Insertions disturb the integrity of the genome structure and gene function, often causing diseases. It is well known that the integration of mobile transposons in human genes causes many diseases^13^. In the Japanese population, an ancient SVA retrotransposon in the FMCD gene causes Fukuyama muscular dystrophy^14^. Similarly, SVA retrotransposition causes other diseases, such as neurofibromatosis^15,16^. Thus, precise estimation of the number of retrotranspositions and their locations in a human genome should be an important consideration for clinical treatment because some locations of active transposons are ethnicity-specific^13^.

To overcome this challenge, the Genome Reference Consortium continuously maintains and updates the international human reference genome. In addition, long-read sequencers have provided another approach to genome analyses, especially to population genome analysis. The PacBio sequencer (Pacific Biosciences; Menlo Park, CA) with single-molecule real-time (SMRT) technology can generate reads longer than ten kilobases (kb), and the maximum length that can be obtained by RSII exceeds 50 kb with the latest version chemistry (P6-C4). This technology combined with assembly allowed us to construct long contigs covering whole-genome regions. In fact, population-specific assembly has already been conducted using PacBio sequencers in several countries^17,18^. These efforts have significantly contributed to the discovery of novel sequences missing from the international reference assembly. However, properties of novel sequences, such as their frequencies in the population and ancestral/derived statuses, have not been well established.

In this study, we assembled whole-genome sequencing data from a Japanese individual generated using a PacBio RSII system. We identified thousands of insertion sequences (TMMINSs; the name represents insertions found in the Tohoku Medical Megabank Project) in the assembled genome, which are difficult to detect directly using short-read sequencers. By integrating TMMINSs into GRCh38, we constructed the Japanese Reference Genome (JRG). Using this newly assembled sequence, by mapping the short-reads from large genomic population studies (e.g., 1KJPN and the international 1000 Genomes Project^12,19^), we cataloged the diverse frequencies of long insertions among populations. For the shared insertions among populations, we also conducted extensive analyses of these novel long sequences to infer their existence dating back to archaic humans.

To clarify the significance of constructing the JRG, we report the discovery of novel single-nucleotide variants hidden in the TMMINSs, including the coding regions, and additionally demonstrate the performance improvements achieved using decoyJRGv1.

## Materials and methods

### Sample information

This project was performed with the approval of the ethical committee of Tohoku Medical Megabank Organization, Tohoku University. The sample JPN00001 was from a Japanese individual who provided written consent for analysis of his whole genome. We confirmed that the sample belonged to the Japanese population by principal component analysis (PCA) conducted using the GCTA portal (a tool for Genome-wide Complex Trait Analysis) in PLINK^20^ ver1.90b3u (Supplementary Fig. 1).

To compare the genome of JPN00001 with those of other populations, we used NA12878, an individual from CEU. The cell line was commercially obtained and cultured in our laboratory.

The data from 1070 Japanese genomes were the same whole-genome sequence data collected on the HiSeq 2500 platform (Illumina Inc.; San Diego, CA) described in a previous study on 1KJPN^12^.

### DNA isolation

More than 100 μg of genomic DNA from JPN00001 was isolated from leukocytes suspended in TE buffer (10 mM Tris-HCl [pH 8.0] and 0.1 mM EDTA [pH 8.0]). Cells were treated with lysis buffer, followed by treatment with RNase A and proteinase K. After phenol/chloroform extraction, cold ethanol was added to the collected aqueous solution. The solution was mixed by inversion until DNA precipitated. The DNA was collected with an inoculating loop, washed with 70% ethanol, and diluted in TE. We avoided using a spin column to obtain the longest DNA molecules possible. The genomic NA12878 DNA was prepared for PCR from a cell line using the same method.

### SMRT sequencing

Genomic DNA was sheared to ~20 kb using a g-Tube (Covaris; Woburn, MA). The libraries for sequencing were constructed with the DNA Template Prep kit 2.0 (3–10 kb; Pacific Biosciences), and size selection was performed using BluePippin (Sage Science; Beverly, MA) according to standard instructions for 20-kb template preparation. For some libraries, the cutoff size was changed from 15 to 18 kb. Sequencing was performed using a PacBio RSII instrument (Pacific Biosciences) with P6-C4 chemistry and a 4 h movie time across 439 cells, yielding 303 Gb (101 × coverage) of data with an average ROI length of 12.7 kb.

### Grouping sequenced data and assembly

The assembly workflow is shown in Supplementary Fig. 2. The sequenced data from PacBio RSII were mapped to the international reference assembly GRCh38 without alternative loci using BWA-MEM^21^ (http://bio-bwa.sourceforge.net/) and separated into 24 groups corresponding to the mapped chromosomes (chr1–22, chrX, and chrY). The following assembly steps were performed for each data group.

Error correction for the data and assembly were performed using FALCON-0.2 (https://github.com/PacificBiosciences/FALCON) based on the general principle of HGAP^22^. The options of two read length cutoffs, “length_cutoff” used for seed reads in the initial mapping process and “length_cutoff_pr” used for seed reads in preassembly, were set at 15,000. Other options were set as follows: “pa_HPCdaligner_option = -v -dal128 -t16 -e.70 -l1000 -s1000”; “ovlp_HPCdaligner_option = -v -dal128 -t32 -h60 -e.96 -l500 -s1000”; “pa_DBsplit_option = -x500 -s50”; “ovlp_DBsplit_option = -x500 -s50”; “falcon_sense_option = --output_multi --min_idt 0.70 --min_cov 1 --local_match_count_threshold 2 --max_n_read 100”; and “overlap_filtering_setting = --max_diff 160 --max_cov 240 --min_cov 5”.

After assembly, contigs shorter than 15 kb were removed, the PacBio reads were aligned to the contigs with PBalign (https://github.com/PacificBiosciences/pbalign), and consensus contigs supported by the majority of the PacBio reads were generated using Quiver, which is included in the GenomicConsensus package ver. 0.9.2 (https://github.com/PacificBiosciences/GenomicConsensus).

To estimate the completeness of the assembly, dot plots were drawn using nucmer and mummerplot in the Mummer3 package^23^ with some modifications to add centromeres and the gap region of GRCh38.

### Insertion detection

The workflow for insertion detection is shown in Supplementary Fig. 2. We aligned the contigs to GRCh38 using BWA-MEM^21^ and identified inserted sequences compared with GRCh38 in the contigs as INSs followed by detection of the INSs using the generated SAM file via two methods, INTRA and INTER. The INTRA method was used to detect inserted sequences inside of continuously mapped regions of the contigs by counting CIGAR string “I”s. The sequences corresponding to the “I”s were identified as INSs. The INTER method was used to detect INSs from split mapping of the contigs. Specifically, for each pair of mapped fragments of the same contig, the clipped sequence between the fragments was identified as an INS when the fragments were mapped to adjacent positions (distance between them was <20% of the length of the clipped sequence) on the reference with the same orientation.

The INSs detected by both methods were filtered using several criteria. First, INSs that were at least 100 bp in length and for which the original contigs were mapped with a mapping quality (mapq) > 0 were selected, and 6700 INSs (3914 for the INTRA method and 2786 for the INTER method) remained. Second, when multiple INSs were located within a distance of 1000 bp from each other, all INSs were removed because these sequences were not considered reliable. After removal of these sequences, 4090 INSs remained. Finally, we integrated the remaining INSs and GRCh38 and constructed the prototype Japanese reference genome. The 8963 refined contigs were then mapped to the prototype. Furthermore, the INSs in the prototype, which were not identical to the corresponding regions in the contigs, were removed. After all of the above filtering steps, we identified 3691 INSs among 2,582,265 total bases (TMMINSs).

### Deletion detection

We investigated deletions in the assembly to confirm the validity of the method. The workflow for deletion detection is shown in Supplementary Fig. 3. Deletion detection was performed with the SAM files used in insertion detection via two methods, INTRA and INTER. The INTRA method was used to detect deleted sequences inside of continuously mapped regions of the contigs by CIGAR string counting “D”s. The sequences corresponding to the “D”s were identified as deletions. The INTER method was used to detect deletions that appeared as gaps or overlaps from the split mapping of contigs. If a pair of contigs generated by the split of one contig (length > 100,000 bases) mapped to the reference at some distance with the same orientation, the sequence of the unmapped region of the reference between the split mapping contigs was identified as a deletion. In the INTER method, the length of deletion (del_len) was defined as follows: (1) if a pair of mapped contigs had no gap compared with the original contig, del_len was equal to the length of the unmapped region in the reference (ref_len). (2) If a pair of mapped contigs had some overlap, del_len was the difference between rlen and the length of the overlap region in the contig. (3) If a pair of mapped contigs had some gap, del_len was the sum of rlen and the length of the gap region in the contig (Supplementary Fig. 3). In addition, the deletions of only those lengths were <0.2 times the original contig length. In both the INTRA and INTER methods, <100 base deletions were filtered.

After filtering, 4040 deletions (2543 for the INTRA method and 1497 for the INTER method) remained.

### Construction of JRGv0

After the detection and filtering steps, the 3691 novel TMMINSs were integrated with the sequence of GRCh38 to create JRGv0 for downstream analysis. TMMINSs detected by the INTRA method were inserted into the detected positions. For TMMINSs detected by the INTER method, the integration method varied depending on the relationships between the mapped positions of fragments generated from one contig (Supplementary Fig. 4) as follows: (i) for fragments adjacently mapped without any gaps (split contig distance = 0), a clipped sequence identified as an INS was inserted into the detected position (Supplementary Fig. 4a); (ii) for fragments mapped with a gap (split contig distance > 0), the gap region in the reference was replaced with the clipped sequence identified as an INS (Supplementary Fig. 4b); and (iii) for fragments with overlapping mapped regions (split contig distance < 0), the ends of the two fragments were mapped to a common reference sequence. The common sequence in the reference was replaced with the clipped sequence identified as an INS, with common sequences added to both ends of the INS (Supplementary Fig. 4c).

The decoyJRGv0 was also constructed by concatenating all 3691 TMMINSs with 20 N bases as a spacer for each TMMINS.

### Alignment of 1072 Japanese genomes to novel reference assemblies

The short-read data generated by the whole-genome sequencing of JPN00001, NA12878, and 1070 individuals in 1KJPN were aligned to three different reference genomes, GRCh38, JRGv0, and GRCh38 + decoyJRGv0. Alignments were conducted using Bowtie2^24^ (version 2.1.0), and variants were detected with Bcftools^25^ (ver. 0.1.17-dev). The alignment tool Bowtie2 with the “-X 2000’’ option was used for the alignment. To evaluate the mapped ratio of each reference assembly, the total, paired-read and single-read mapped ratios were calculated using the results from samtools^25^ with the flagstat option.

### Detection of variants in the TMMINS regions

Bcftools was used to detect the SNVs in the alignment results of 1072 individuals with Bowtie2. The results were merged with a custom script, and the numbers of biallelic SNVs were counted.

### Alignment of other populations and related species with JRGv0

Short-read data (fastq format) from individuals of the international 1000 Genomes Project were downloaded from the project sites, including ACB, BEB, YRI, KHV, CHB, JPT, CEU, and CLM, from a total of 767 individuals (Supplementary Table 1). These fastq data were aligned using the same procedure as that used for 1KJPN. The fastq data of Denisovan (http://cdna.eva.mpg.de/denisova/) and Neanderthal (http://cdna.eva.mpg.de/neandertal/altai/) genomes were downloaded and aligned using the alignment tool (Bowtie2 version 2.1.0) with the default options in the alignment mode.

### Validation of novel insertions by PCR

Ten TMMINSs selected from 3691 novel INSs in JRGv0 were validated in two genomes (JPN00001 and NA12878) by PCR followed by sequencing of the PCR product on the PacBio RSII instrument. We obtained PCR products for sequencing from two reactions. Primer design was carried out according to the Guidelines for Using PacBio Barcodes for SMRT Sequencing (PacBio) with some modifications. For the first PCR, we designed gene-specific primers tagged with two different 30-bp universal sequences. We did not add any modifications to the ends of the primers, but the manual recommended adding a NH_4_-C_6_ block at the 5′-end. PrimeSTAR GXL DNA Polymerase (TaKaRa, Shiga, Japan) was used with the following conditions: 25 cycles of 10 s at 98 °C and 10 min at 68 °C. For the second PCR, we designed primers with 16-bp index sequences based on the PacBio guideline for 48 paired barcodes at the 5′-end of the universal sequences. We used two indices for the two genomes. KOD FX Neo (Toyobo; Osaka, Japan) was used with the following conditions: 1 min at 94 °C, 25 cycles of 10 s at 94 °C and 10 min at 72 °C, followed by a final 5 min at 72 °C. The primer sequences for both PCRs are listed in Supplementary Table 2.

The PCR products for ten TMMINSs were mixed, and libraries were prepared and indexed separately for the two genomes according to the manufacturer’s protocol. For sequencing, two libraries were mixed and sequenced using one cell with P6-C4 chemistry and a 4-h movie time, generating 608 Mb of data.

### Annotation of TMMINSs

**Fig. 1:**
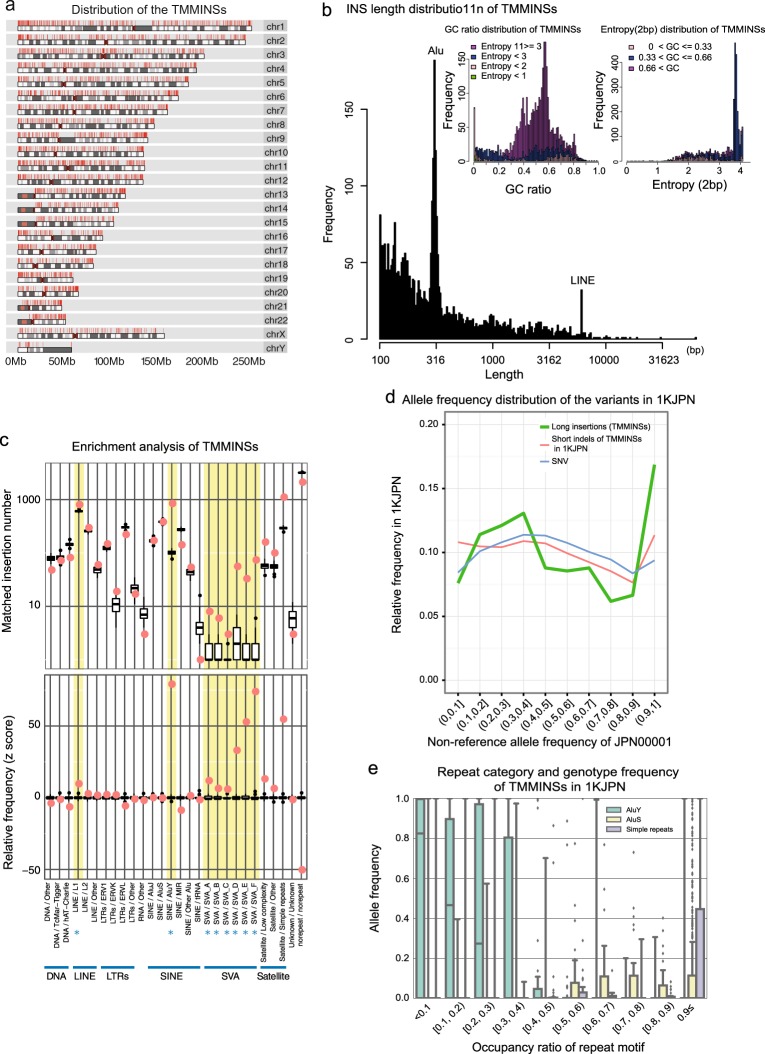
Features of the 3691 long insertions (TMMINSs). **a** Distribution of the 3691 insertions in the chromosomes. The red lines on the chromosomes indicate the locations of the insertions on each chromosome. The gray bands in each chromosome indicate its cytobands. **b** Distribution of the lengths of TMMINSs. The two prominent peaks correspond to Alus and LINEs. Left inner box: distribution of GC ratios accompanied by entropy information. Right inner box: TMMINSs with high entropy tended to show medium GC ratios of ~0.5. The distribution of entropy is accompanied by the GC ratio information. The peaks in the high-entropy region indicated that many TMMINSs had high complexity. **c** Repeat motif enrichment analysis of TMMINSs. The boxplot indicates the background distribution of the total number of motif classes. Each box represents the 25th and 75th percentiles of the total number of each motif. The notches represent the 1.5 × interquartile range. The red dots outside the notches indicate the enriched motif classes in TMMINSs. The other black dots show outliers. **d** The relative frequencies of nonreference alleles of TMMINSs, SNVs, and short indels are indicated as green, red, and blue lines, respectively. The nonreference allele frequencies of each variant were calculated from the genotypes of 1070 individuals, and only variants found in JPN00001 were used. **e** Repeat categories and allele frequencies of TMMINSs in 1KJPN. The horizontal axis shows the allele frequencies of TMMINS in 1KJPN, and the vertical axis shows the occupancy ratio of repeat motifs in TMMINS

The length distribution was created by counting the number of TMMINS bases. The GC ratios were calculated by dividing the total number of bases of each TMMINS by the total number of G and C bases. The entropy of the two bases was obtained by calculating the entropy of the counts of 16 patterns with all two-base combinations of A, T, G, and C. These results are plotted in Fig. 1b.

The repeat regions were annotated to JRGv0 with RepeatMasker^26^ (ver. 4.0.6). After the repeat annotation, the total number of each repeat class was counted for the TMMINS regions in JRGv0. For a TMMINS, if multiple repeat classes were assigned, each repeat class was counted multiple times. For comparisons with the null distribution, random locations in JRGv0 with a length distribution equal to that of TMMINSs were generated 1000 times, and the number of each repeat class was counted as described above. The results are plotted in Fig. 1c.

### Copy number analysis of TMMINSs

Sequence reads from 1KJPN, JPN00001, NA12878, 767 individuals from i1000g (Supplementary Table 1), Neanderthal, Denisovan and chimpanzee were subjected to copy number analysis of TMMINSs. For each individual, reads were aligned to JRGv0 using Bowtie2 (version 2.1.0) with the “-X 2000” option. The read coverage of every 50-bp window in JRGv0 was calculated, where reads were regarded as being aligned on a window if the midpoints of the reads were contained in the window. A window was called “alignable” if the ratio of aligned reads on the window with a mapping quality ≤ 1 to the total coverage did not exceed 40%.

To quantify read coverage at TMMINSs for each individual, a variation of the read coverage distribution with respect to the GC content in genomic regions, known as GC bias, was corrected as follows. The GC content in each window was calculated as the ratio of C and G bases in a 400 bp flanking region, which started from the 200 bp upstream of the window’s start position. The alignable windows were grouped by their GC content values, in which the width of the value for each group was set to 2%. The standard read coverage for a GC content value was defined as the 10% truncated mean of the read coverage for the corresponding group, in which at least 100 windows remained after the truncation. The normalized coverage for each alignable window was defined as the coverage divided by the standard coverage corresponding to the GC content value of the window. The normalized coverage of TMMINS was calculated as the average of the normalized coverage of the alignable windows, except that the calculated value was invalidated for less confident insertions, which were covered only by alignable windows with <50 bp or <30% of the insertion length.

The copy numbers of TMMINSs for individuals were analyzed based on a statistical model for the normalized coverage of multiple individuals. In the model, the normalized coverage of an insertion for individual *n* was assumed to follow a normal distribution with the mean of $y_n = a + b\mathop {\sum }\nolimits_{j = 1}^{A_n} z_{nj}$ and the variance of *c*^–1^, where *a*, *b*, and *c* were fitting parameters; *A*_*n*_ was the number of chromosomes, which was 2 for autosomals and between 0 and 2 for sex chromosomes; and *z*_*nj*_ was the haploid copy number of the insertion on the j-th chromosome of the individual, which utilized an integer value between 0 and *M* and followed the multinomial distribution with parameters *θ*_0_, …, *θ*_M_. The parameters *a*, *b*, *c*, and *θ* were determined with a maximum a posteri (MAP) estimation using the EM algorithm. The prior distributions of *a*, *b*, *c*, and *θ* were *a* ~ Norm(0, *λ*_*a*_), log *b* ~ LogNorm(0, *λ*_*b*_), *c* ~ Gamma (*λ*_*c*_), and *θ* ~ Dir(*θ*|*λ*_*θ*_), respectively. In this study, we set *λ*_*a*_ = 8.0, *λ*_*b*_ = 16.0, *λ*_*c*_ = 1.0, *λ*_*θ*_ = 1.0, and *M* = 10. The estimation was performed with three different initial values of *a* = (0, 0.4, 0.8), *b* = 1.0, and *c* = 1.0. The estimated results were accepted if the parameters *a* and *b* were |*a*| ≤ 0.75 and 0.6 ≤ *b* ≤ 1.4, respectively. We selected the best result in terms of the log likelihood. For each individual, the copy number of the insertion was set to *z*_*n1*_ + *z*_*n2*_ if the posterior probability P(*z*_*n1*_ + *z*_*n2*_ | *x*_*n*_) was >0.8; otherwise, the value was set to NaN. Copy number analysis was performed independently for the 1KJPN and i1000g datasets. For the analysis of Neanderthal, Denisovan and chimpanzee genomes, the estimated parameters from 1KJPN were used to calculate the posteriors of the copy numbers.

### Variant discovery rates for novel insertions

In a sample of *2n* chromosomes from an infinitely large population, the variant discovery rate was calculated as the sampling probability of polymorphic sites as follows:

$$P\left( {2n,q_{{{\rm min}}}} \right) = \frac{{\mathop {\int }\nolimits_{q_{{{\rm min}}}}^{1 - q_{{{\rm min}}}} \left\{ {1 - \left( {1 - q} \right)^{2n}} \right\}F\left( q \right)dq}}{{\mathop {\int }\nolimits_{q_{{{\rm min}}}}^{1 - q_{{{\rm min}}}} F\left( q \right)dq}},$$

where *q*_min_ is the minimum minor allele frequency (MAF) of interest and *F*(*q*) is the distribution of allele frequencies in a population with a demographic history. In this study, *F*(*q*) was numerically calculated based on the demographic model inferred from the site frequency spectrum of intergenic SNVs^12^.

Supposing that the reference genome sequence was assembled from *n* individuals, if these individuals had a homozygous deletion at a locus, the alternative insertion type sequence was considered to be missing from the reference genome assembly by chance. Such situations were more likely if *n* was small and/or if *q*_min_ was low. However, because the effective number of individuals, *n*, who contributed to the reference genome assembly was unknown and may have varied among loci, the variant discovery rates of such “missing” inserted sequences were estimated with a different *n* (1–20) and a different *q*_min_.

### Construction of JRGv1

For public release, we separately constructed JRGv1 and decoyJRGv1 in accordance with the policy of the ethical committee to avoid identification of the individual whose genome was sequenced in this study.

The construction procedure was as follows: from 3691 INSs, 903 found in at least one of 1070 Japanese individuals were integrated with GRCh38 to create JRGv1. The decoyJRGv1 was constructed by concatenating 3559 of 3691 TMMINSs that were found in at least one of 1070 Japanese individuals or in six other assemblies; 20 N bases were used as a spacer for each TMMINS. If a rare SNP in the Japanese population was included in the selected TMMINSs, it was changed to a major SNP.

## Results

### Sequencing and assembly

Several Japanese individuals in the prospective cohort study performed by the Tohoku Medical Megabank Organization (ToMMo) were recruited after providing written informed consent. One individual (JPN00001) was selected for verification that he was clustered into a Japanese population using principal component analysis (PCA; Supplementary Fig. 1). To construct the Japanese reference genome, deep whole-genome sequencing with 101 × coverage (303 Gb) was performed using a SMRT sequencer (PacBio RSII; Pacific Biosciences), yielding an average read of insert (ROI) length of 12.7 kb. These sequenced reads were aligned to the chromosomes, i.e., chr1–22, chrX, and chrY, in the international reference genome GRCh38. The reads, except for unmapped reads (2.98 M reads, 7.15 Gb in total), were separated into 24 groups corresponding to the mapped chromosomes in the reference genome (Supplementary Fig. 5a). Grouping the sequence reads by mapping to GRCh38 before assembly^27^ is considered to be an effective method for avoiding misassembly. The mean and median lengths of the mapped reads were 9158 kb and 7416 kb, respectively. In contrast, the mean and median lengths of unmapped reads were much shorter (2400 kb and 1939 kb, respectively).

The data coverage was 87.1–113.0 × for autosomes, 54.4 × for chromosome X, and 41.3 × for chromosome Y (Supplementary Table 3). Error correction, assembly, and refinement of the contigs were performed for each group (Supplementary Fig. 2), and in total, 8963 contigs were obtained. For each chromosome, the number of refined contigs ranged from 112 to 768, and the N50 contig length and average contig length were 627,963–2,797,989 bp and 191,766–467,954 bp, respectively (Supplementary Table 3). Our assembly covered the human genome at a magnitude equal to that of accessible regions in GRCh38. The ratio of the total contig length of our assembly to that of GRCh38 was higher than 1.00 in 17 chromosomes and 1.023 for all chromosomes (Supplementary Table 3 and Supplementary Fig. 6a, b). Our sequenced reads were also mapped to the centromere region and contributed to the assembly process (Supplementary Fig. 7) because, in contrast to GRCh37, the centromere regions were extensively updated by replacement with modeled centromere sequences^28,29^ in GRCh38.

Our assembled contigs were compared with other contigs assembled in previous studies (Supplementary Table 4)^17,18,30,31^. The constructed contigs in our assembly were longer than those previously reported for PRJNA253696 and CHM1_1.1^30,31^ in terms of the contig number, N50 contig length, and average contig length. This improvement could be explained by the use of the most recently developed chemistry (P6-C4) and higher sequence coverage (101 × coverage; Supplementary Table 4) than those of previous studies. In our analysis, the target insertions were 100 bases to 10 kb in length, and we did not apply scaffolding by combining ultralong spanning read technology (100 kb to 1 Mb) like Korean and Chinese assemblies^18,19^, e.g., BioNano^32,33^ and 10x genomics^34^.

### Novel long insertions and their features

We aligned the 8963 contigs to GRCh38 and detected insertion sequences not found in GRCh38 (Supplementary Fig. 2). After filtering out unreliable insertions from the candidates, 3691 insertions were identified from a total of 2,582,265 bases ranging from 100 bp to 62,338 bp (Supplementary Fig. 2 and Table 1a, b). TMMINSs were located in all regions of the chromosomes without prominent biases (Fig. 1a).

**Table 1a:** Length and ratio of genome repeat class to TMMINSs. Features of TMMINSs

Class	Subclass	Total	Mean length	Total number of normalized bases	Ratio of normalized length to total length	Active Mobile Element
No. of repeats	2922	811	2,370,250	0.2686		
SINEs	AluJ	171	1978	338,180	0.0383	
	AluS	382	1525	582,731	0.066	
	AluY	846	586	495,440	0.0561	Yes
	MIR	142	2103	298,660	0.0338	
	Other Alu	54	2245	121,255	0.0137	
	Total	1595	1151	1,836,266	0.2079	
LINEs	L1	804	1440	1,157,769	0.1312	Yes
	L2	297	1554	461,442	0.0523	
	Other	60	1810	108,581	0.0123	
	Total	1161	1488	1,727,792	0.1958	
LTR	ERVL	222	1838	408,002	0.0462	
	Other	179	1872	335,097	0.038	
	Total	401	1853	743,099	0.0842	
SVA	SVA_A	8	505	4,041	0.0005	Yes
	SVA_B	6	1837	11,024	0.0012	Yes
	SVA_C	3	1523	4,569	0.0005	Yes
	SVA_D	56	606	33,961	0.0038	Yes
	SVA_E	33	1899	62,654	0.0071	Yes
	SVA_F	74	1271	94,026	0.0107	Yes
	Total	180	1168	210,275	0.0238	
DNA	hAT-Charlie	82	2224	182,389	0.0207	
	TcMar-Tigger	72	1678	120,841	0.0137	
	Other	48	2002	96,113	0.0109	
	Total	202	1977	399,343	0.0453	
RNA	Total	4	2341	9,364	0.0011	
Satellite	Low complexity	160	1090	174,477	0.0198	
	Simple repeats	1119	1139	1,274,131	0.1444	
	Other	93	817	75,985	0.0086	
	Total	1372	1111	1,524,593	0.1728	
Unknown	Other	3	1363	4,089	0.0005	
All	Total	7840	1126	8,825,071	1

**Table 1b:** Statistics of TMMINSs

Chr	Number of TMMINSs	Sum of TMMINSs length	GRCh38 original length	JRGv1_len	Increased length
1	309	239,427	248,956,422	249,198,570	242,148
2	243	187,725	242,193,529	242,386,836	193,307
3	216	202,994	198,295,559	198,505,658	210,099
4	195	99,669	190,214,555	190,315,561	101,006
5	168	125,425	181,538,259	181,665,793	127,534
6	209	131,123	170,805,979	170,939,170	133,191
7	218	141,534	159,345,973	159,491,300	145,327
8	152	82,336	145,138,636	145,221,493	82,857
9	189	121,060	138,394,717	138,517,748	123,031
10	173	140,919	133,797,422	133,954,928	157,506
11	208	134,875	135,086,622	135,226,433	139,811
12	178	144,826	133,275,309	133,423,817	148,508
13	172	100,189	114,364,328	114,466,305	101,977
14	97	93,675	107,043,718	107,139,526	95,808
15	87	55,846	101,991,189	102,048,214	57,025
16	96	50,737	90,338,345	90,389,652	51,307
17	130	89,379	83,257,441	83,348,358	90,917
18	93	43,078	80,373,285	80,416,794	43,509
19	118	80,500	58,617,616	58,701,084	83,468
20	136	78,303	64,444,167	64,526,175	82,008
21	83	68,287	46,709,983	46,780,291	70,308
22	83	35,720	50,818,468	50,855,005	36,537
X	112	88,583	156,040,895	156,131,050	90,155
Y	26	46,055	57,227,415	57,280,509	53,094
Total	3691	2,582,265	3,088,269,832	3,090,930,270	2,660,438

The sum of TMMINSs length for each chromosome is not consistent with the increased length because some of original sequences in GRCh38 was removed when the TMMINSs inserted to GRCh38 (see Supplementary Fig. Integration of detected INSs to GRCh38)

We characterized TMMINSs, focusing on their length, sequence complexity, GC ratio, and repeat classes (Fig. 1b). As expected, the insertion length distribution for TMMINSs showed two clear peaks around the length of Alu and long interspersed element (LINE) repeats (Fig. 1b). Moreover, a substantial fraction of TMMINSs had very low GC ratios (GC < 10%; 233 out of 3691; 6.3%). In contrast, very few TMMINSs had very high GC ratios (GC > 90%; 6 out of 3691; 0.16%; Fig. 1c). This result is consistent with that of a previous study in which the GC and AT enrichment ratios were compared with the sequences of gap closures (Fig. 1b in ref. ^21^). The major TMMINSs had GC ratios within 20 to 80% (3306; 89.6%) and had high entropy, larger than 2.0 (3097; 83.9%; Fig. 1b). These results imply that the majority of the missing insertions in GRCh38 cannot be categorized as simple repeats.

In TMMINSs, active mobile elements (asterisks in Fig. 1c) were enriched. Figure 1c shows the enrichment analysis of the repeat subclasses and nonrepeat class in TMMINSs. SINE/VNTR/Alu (SVA) subclasses A–F were significantly enriched in TMMINSs. In the human assembly, more than 2000 full-length SVAs were identified^16^, likely resulting from continuous activity through 25 million years of hominoid evolution^35^. In addition, other known active mobile elements, LINE1^36^ and AluY^37^, were also enriched in TMMINSs. In contrast, the inactive mobile elements LINE2, AluJ, and AluS were not enriched. These results are consistent with the results of previous studies^36,37^.

Table 1c shows the genetic annotations to TMMINSs. In total, 59 and 1112 TMMINSs overlapped with exonic and intronic regions, indicating that some long insertions are still discoverable in coding regions. Table 1d summarizes the distance from the known variants registered in the Genome-Wide Association Studies catalog^38^. Fifty-three TMMINSs were discovered to be <1000 bases from these variants.

**Table 1c:** Summary of genetic annotations to TMMINSs^a^

Class	Count
Intergenic	1792
Motif	31
Transcript	1660
Gene	2245
Exon	59
Intron	1112
Upstream (5kb)	341
Downstream (5kb)	402

The software annotates multiple classes to one insertion

^a^In total, 3691 TMMINSs were annotated using SNPEff ver 4.3b

**Table 1d:** Summary of annotation with GWASCatalog^a^

Distance (base)	
<100	1
<1K	43
<10K	363
<100K	1751
<1M	1372
<10M	135
NA	26
Total	3691

^a^The version downloaded at 29/Jan/2016. NA is TMMINSs in chrY

### Evaluation of the detected deletions

The maximum size of the detected deletions was 172 kb. The size distribution of 4040 detected deletions is shown in Supplementary Fig. 8. It showed similar peaks of insertions, corresponding to Alu and LINE. The pattern of the peaks was also consistent with that reported for Korean genome data^17^. Although deletions longer than 100 kb were already detected with short-read data^12^ and investigation of deletions was not the aim of this study, this result suggests the reliability of the assembly in this study.

### Comparison with other human assemblies

Next, we searched for the existence of TMMINSs in the published Korean human genome assembly AK1 to clarify how many detected insertions were shared because the sequencing coverage and technology used in this study were almost equal to those used in our approach, i.e., SMRT long-read sequencing technology with ~100 × coverage. We found that 1873 of 3691 (50.7%) sequences shared insertion positions within 50 bases each other (Supplementary Fig. 9a). The square of the correlation coefficient for the insertion length of the shared 1834 insertions was 0.9606 (Supplementary Fig. 9c). This result implies that the length of many shared insertions is conserved between JPN00001 and AK1 and suggests that many of the TMMINSs are not specific to Japanese but are rather broadly shared with other populations. Some of these sequences may have been absent from the International Reference Genome because of the process required to construct the bacterial artificial chromosome (BAC) clones for the reference assemblies^39^ and the properties of the sequencing technology, e.g., polymerase chain reaction (PCR) bias and sequencing errors^40,41^. We overcame these drawbacks by using DNA from whole blood without any amplification and SMRT sequencing technology.

### Insertions in 1070 Japanese individuals and other populations

**Fig. 2:**
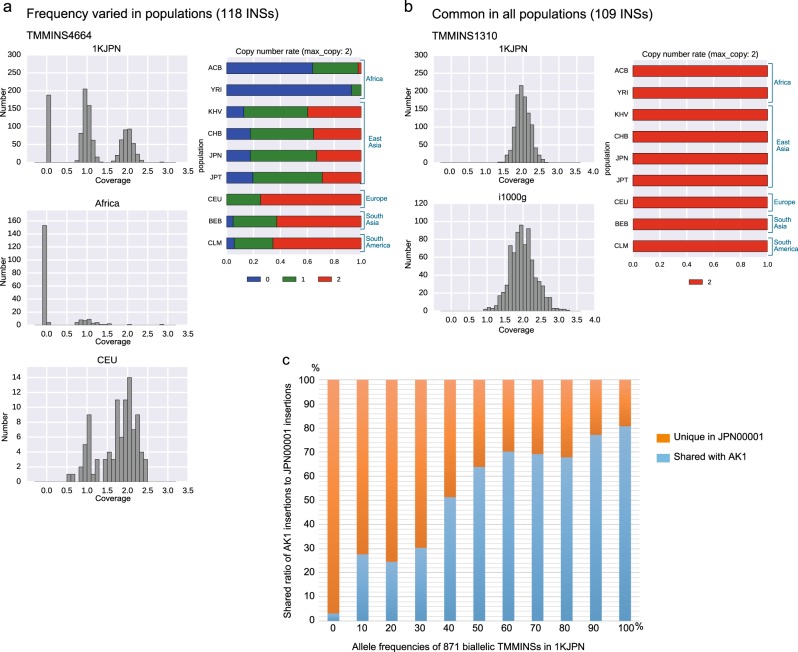
Typical patterns of read coverage distributions of TMMINSs in 1KJPN and other populations. The left side of each panel shows the normalized coverage distribution of a TMMINS in the 1KJPN (top) and i1000g (bottom) populations, and the right side shows the genotype frequencies in eight populations. Blue: null, green: hetero, red: homo. **a** The allele frequencies were rare, common, and abundant in the African, East Asian and CEU, and BEB and CLM populations, respectively. **b** A monomorphic sequence in modern humans. **c** Correlation of the allele frequencies of 871 biallelic TMMINSs in 1KJPN (*x*-axis) and the shared ratio between JPN00001 and AK1 (*y*-axis)

Upon comparing the assemblies of one Korean and one Japanese human genome, we detected numerous shared insertions (Fig. 2c). To investigate whether the identified TMMINSs are specific to Korean or Japanese populations or shared with other diverse populations, we tried to estimate the allele frequency of each TMMINS in various populations. To do this, all sequenced reads obtained from the whole-genome sequencing of 1070 Japanese individuals (1KJPN)^12^, JPN00001, and NA12878 (HapMap^42,43^ CEU sample) were aligned to JRGv0. The sequenced data were obtained using short-read sequencers (HiSeq 2500) with a 32.4 × mean coverage^12^. In addition, 767 individuals from different populations from the international 1000 Genomes Project (referred to as i1000g), including the African Caribbean in Barbados (ACB); Yoruba in Ibadan Nigeria (YRI); Colombian in Medellin, Colombia (CLM); Kinh in Ho Chi Minh City, Vietnam (KHV); Han Chinese in Beijing, China (CHB); Japanese in Tokyo, Japan (JPT); Bengali in Bangladesh (BEB) and Utah residents with Northern and Western European ancestry from the CEPH collection (CEU), were selected and analyzed with the same method to estimate the allele frequency of each TMMINS (Supplementary Table 1).

Figure 2a, b shows two of four typical patterns of depth distribution among 1070 Japanese individuals and the i1000g populations after normalization. The four patterns were as follows: (i) varied frequencies among populations, e.g., the CEU, BEB and CLM populations showed higher frequencies than East Asian populations, whereas African populations showed lower frequencies (Fig. 2a); (ii) mainly shared in East Asian and CLM populations but rare in other populations (Supplementary Fig. 10a); (iii) almost monomorphic in East Asian populations but not in other populations (Supplementary Fig. 10b and Supplementary Material 1); and (iv) common in all populations, indicating a monomorphic feature in modern humans (Fig. 2b).

For additional population genetics analysis, we applied a statistical method to estimate the allele counts of TMMINSs for each individual from the depth distribution (Supplementary Table 5). To avoid the influence of copy number variants, we focused on the biallelic variation, i.e., 0, 1, or 2 alleles being present in >95% of individuals, enabling direct comparison between biallelic SNVs and short insertions and deletions (<100 bases) detected in our previous study on 1KJPN^12^.

With the limitation of short-read sequencers, some repetitive sequences were still difficult to distinguish from other similar regions in the human genome assembly. Thus, insertions were selected with the following two conditions: (i) a certain number of unique reads were aligned, and (ii) the allele copy number of JPN00001 estimated from the short-reads was relevant, i.e., 1 or 2. Finally, 871 autosomal biallelic long insertions were obtained (Supplementary Table 5). Ten of these TMMINSs were validated by PCR amplification and sequencing with PacBio RSII (Supplementary Tables 6 and Supplementary Figs. 11 and 12). All ten insertions were successfully validated in JPN00001. Among them, one insertion (negative control), which does not exist in European individuals (NA12878), was not observed when tested with the same validation experimental protocol as that used for JPN00001.

Interestingly, a substantial fraction of these insertions presented as very common insertions among a wide range of modern human populations. We found that 166 insertions (19.1%) were homozygous for all 1070 Japanese individuals. Furthermore, among these insertions, 109 biallelic insertions (12.6%, length distributions are shown in Supplementary Fig. 13a) were homozygous for all individuals in the i1000g populations.

The alternative (i.e., sequences not present in GRCh38) allele frequencies within the 1KJPN population for the remaining 702 biallelic long insertions are shown in Fig. 1d. The allele frequencies of 871 novel insertions were compared with those of SNVs and short insertions/deletions (short INDELs). Because the allele frequency of novel insertions was estimated conditionally based on those observed in JPN00001, these spectra were expected to be skewed toward higher alternative alleles. Thus, the allele frequency spectra of SNVs and short INDELs were obtained from variants discovered in the JPN00001 individual. This result shows that the distributions among different variant classes, i.e., SNVs, short INDELs and long insertions, were consistent. Thus, variations in novel insertions observed among the 1070 individuals could be explained by polymorphisms. We further investigated the relationship between the allele frequencies of TMMINSs in the Japanese population and the shared insertions between JPN00001 and AK1. Figure 2c shows the positive correlation between the allele frequencies in the Japanese population and the ratio of shared insertions between JPN00001 and AK1 (see also Supplementary Fig. 9c). This result was expected because insertions with higher allele frequencies in a Japanese population are likely to be shared between Japanese and Korean populations.

We also compared the allele frequencies of 871 insertions between 1KJPN and i1000g (JPT) from both Japanese populations (Supplementary Fig. 13b). The genetic backgrounds of 1KJPN and i1000g (JPT) are similar, and the frequency of insertion should be correlated. The sequence length of i1000g was 75 bases based on the paired-end protocol. In contrast, the sequence length of 1KJPN was 162 bases based on the paired-end protocol. In addition, the mean coverage of i1000g was 3.6 × (low-depth protocol), while that of 1KJPN was 32.4 × (high-depth protocol). The difference sometimes causes critically impacts the alignment performance to the reference assembly. If some of the sequenced read covers the unique sequence in the human genome assembly, the sequenced read can be properly aligned to the human genome assembly using this unique sequence information. 1KJPN has a longer read length for each sequenced read than i1000g and can sometimes cover more unique regions in the human genome assembly. In addition, the low-depth protocol sometimes results in very low coverage of the target insertion region (in this case, the insertion is considered “not existent” in this sample).

Thus, the discovery ratio of TMMINs in 1KGP-JPT should be lower than that in 1KJPN. Supplementary Fig. 13b shows the relationship between the frequency of insertions in 1KJPN and 1KGP (JPT) for the 871 TMMINSs and satisfies this hypothesis. Importantly, many insertions with low frequency in i1000g (JPT) and high frequency in 1KJPN were also discovered in AK1 (Supplementary Fig. 13c, Supplementary Table 7 and Supplementary Material 3). Thus, these results strongly suggest that the 871 biallelic insertions selected with our method are applicable for the estimation of genotypes containing long insertions.

Among the 871 biallelic insertions, simple repeats and AluS were enriched in those with higher allele frequencies (Spearman’s correlations: 0.27 and 0.11, *p*-values: 7.63e^−19^ and 1.11e^−3^, respectively). In contrast, the occupancy ratio of AluY was significantly enriched (Spearman’s correlation: −0.270, *p*-value: 6.54e^−16^) in TMMINSs with lower allele frequencies (Fig. 1e), suggesting that the allele ages of these elements are relatively low. This result is consistent with the observation that active mobile elements were enriched (Fig. 1c).

### Insertions in archaic humans

**Fig. 3:**
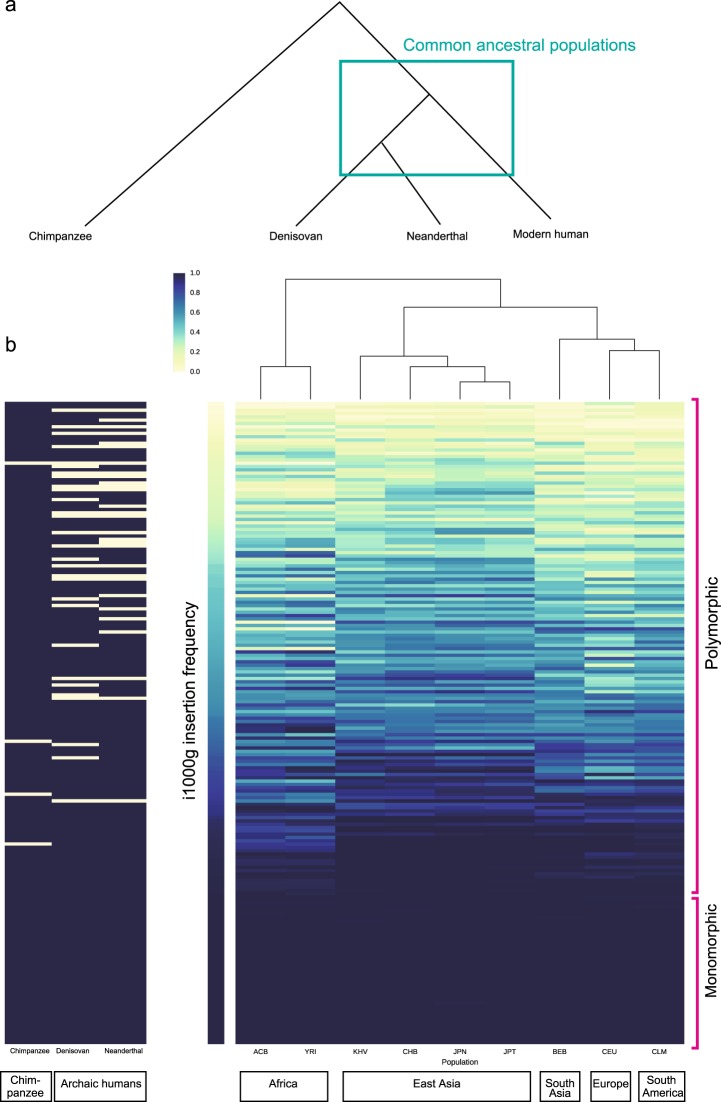
Insertion frequency heat map. **a** Phylogenetic relationship among chimpanzees, Denisovans, Neanderthals, and modern humans. **b** Heat map of the allele frequencies of 194 selected TMMINSs. The right panel shows the frequency in 1KJPN and other population data from HapMap projects^42,43^ (i1000g). The vertical axis is ordered by the allele frequencies in i1000g. The populations were clustered into three groups: Africa, East Asia, and others. The middle color bar indicates the frequency in all i1000g populations. The left panel shows the existence of each allele in the genomes of a chimpanzee, a Denisovan, and a Neanderthal

If a novel sequence was also discovered in the genome of a species having recent common ancestry with modern humans, the sequence retaining the ancestral state was considered to have been derived from an insertion event in human ancestry after divergence of these species. To determine the origin of the novel sequences, we aligned high-coverage whole-genome sequencing data from archaic humans (a Denisovan^44^ and a Neanderthal^45^) and a chimpanzee^46^ to JRGv0 (Fig. 3a) and estimated the existence of long insertions as 0 (not existing), 1 (existing), or not determined (caused by low mapping quality).

From 871 biallelic insertions, we identified 194 insertions with the same selection criteria as that used for i1000g (call rate ≥ 0.95) and a 0 or 1 call in all Denisovan, Neanderthal, and chimpanzee sequences (hereafter referred to as related species) (Supplementary Fig. 5c). The low passing rate (~22%) of the downstream analysis was likely a result of the low coverage sequencing, sequence length for each read or low quality of the DNA for the Denisovan and Neanderthal samples. Figure 3b shows a heat map of the allele frequencies in human populations (ACB, YRI, KHV, CHB, 1KJPN, JPT, CEU, BEB, and CLM) and the existence of insertions in the Denisovan, Neanderthal, and chimpanzee genomes.

The human populations were organized by hierarchical clustering (horizontal axis in Fig. 3b), and the absolute distance of the allele frequencies was used as a measure of dissimilarity between populations. As expected, the two samples representing Japanese populations, 1KJPN and JPT, in i1000g were locally clustered. Populations with similar genetic backgrounds were closely clustered, e.g., East Asia: KHV and CHB Africa: ACB and YRI.

As the allele frequency in modern human populations decreased, the insertions were more likely to be absent in Neanderthals and/or Denisovans (upper region in Fig. 3b), suggesting that these genetic components shared between modern and archaic humans are derived from a common ancestral population (rectangle in Fig. 3a). Notably, all but four insertions were observed in the chimpanzee genome (Fig. 3b). Assuming that the insertions found in the chimpanzee genome represent the ancestral state, these results imply that the deletion events occurred in human ancestors after branching from the chimpanzee lineage. Moreover, a potential reason underlying the missing sequences in the reference genome may be the polymorphic state of these sequences, i.e., sequenced individuals in the reference assembly did not have these insertions.

In contrast, 34 insertions were monomorphic among modern humans. These insertions were also shared with archaic humans and chimpanzee (lower region in Fig. 3b). As previously discussed, these insertions were missing from the reference assemblies because of technical limitations, e.g., the process of constructing the BAC clones^39^ and PCR amplification bias.

### Construction of the Japanese reference genome and decoy

We constructed the Japanese reference genome JRGv0 by integrating TMMINSs into the international reference assembly GRCh38 (Supplementary Fig. 5b and Methods). We also concatenated all TMMINSs with 20 N bases and constructed the virtual chromosome decoyJRGv0 (Supplementary Fig. 5b and Methods). The virtual chromosome could be used as a decoy^28^ sequence, which could reduce false-positive alignments to the reference assembly.

To validate the performance improvements using JRGv0 and GRCh38 with decoyJRGv0 instead of GRCh38, we compared the alignment statistics of 1,070 Japanese individuals (1KJPN), JPN00001 and NA12878. For all samples, the total alignment ratio and paired alignment ratio to JRGv0 were improved by 0.435% (SD 0.065%) and 0.424% (SD 0.063%), respectively (Table 2a, b, Supplementary Fig. 14 and Supplementary Material 2). Notably, these improvements reached 16% of all the unmapped sequenced reads. The single alignment ratio (i.e., the total ratio for paired sequenced reads in which only one sequence was aligned) did not increase (Table 2c and Supplementary Fig. 14) in JRGv0 compared with that in GRCh38. In addition, the proper alignment ratio in JRGv0 compared with that in GRCh38 was 0.407% (SD 0.060%). These observations imply that the valid alignment ratio increased without increasing invalid alignments using JRGv0 as the reference genome for 1KJPN and JPN00001. The same behavior was also observed for the NA12878 CEU sample. Thus, JRGv0 may also be valuable for use with other populations.

**Table 2:** Alignment performance of JRGv0 and GRCh38 + decoyJRGv0

(a) The comparision of alighment ratio with GRCh38, GRCh38 + decoyJRGv1 and JRGv1				
	Mean	S.D.	Improvement (compared to total reads)	Improvement (compared to unmapped reads)
Alignment ratio with GRCh38	96.92%	0.51%	-	-
Alignment ratio with GRCh38 + decoyJRGv1	97.35%	0.50%	0.43%	16.22%
Alignment ratio with JRGv1	97.36%	0.50%	0.44%	16.47%

(b) The comparision of alignment reads with GRCh38, GRCh38 + decoyJRGv0 and JRGv0			
	Mean	S.D.	Improvement
Alignment reads with GRCh38	562,209,444	19,701,509	-
Alignment reads with GRCh38 + decoyJRGv1	564,573,770	20,176,172	2,364,326
Alignment reads with JRGv1	564,752,883	19,766,532	2,543,439

(c) The comparison of the relative proper alignment ratio and single alignment ratio with JRGv0-GRCh38 and GRCh38 + decoyJRGv0-GRCh38		
	Diff	S.D.
Relative proper alignment ratio improvement from GRCh38 to JRGv0	0.382%	0.060%
Relative proper alignment ratio improvement from GRCh38 to GRCh38 + decoyJRGv0	0.024%	0.006%
Relative single alignment ratio difference from GRCh38 to JRGv0	0.016%	0.006%
Relative single alignment ratio difference from GRCh38 to GRCh38 + decoyJRGv0	0.011%	0.006%

We also applied a different aligner, BWA-MEM (ver. 0.7.12-r1039), with the default option and evaluated the performance. The aligner tried to align more ambiguous reads to the reference assembly than the former aligner Bowtie2. Consequently, the total alignment ratio using GRCh38 was 99.2% (SD 0.257%) in BWA-MEM and 96.9% (SD 0.005%) in Bowtie2. The total alignment ratio to JRGv0 and decoyJRGv0 were slightly improved by 0.009% (SD 0.003%) and 0.008% (SD 0.004%), respectively. Interestingly, the total proper alignment ratio to JRGv0 and decoyJRGv0 were improved by 0.273% (SD 0.04%) and 0.244% (SD 0.041%), respectively (Supplementary Figs. 14, 15, and Supplementary Material 2). This result suggests that misaligned sequenced reads in GRCh38 were aligned to correct positions using these custom reference assemblies.

**Fig. 4:**
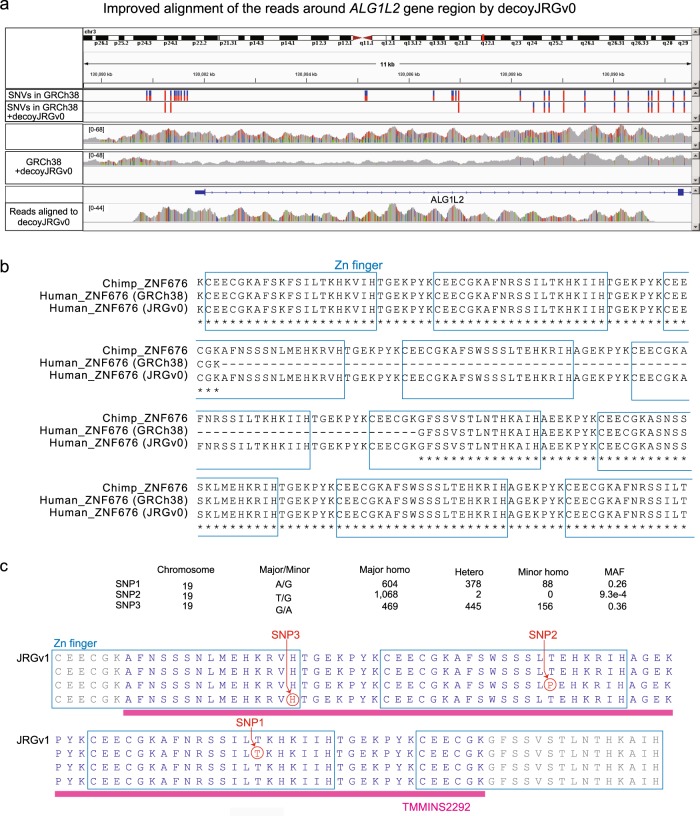
Insertions in the ZNF676 region and functional single-nucleotide variants in 1KJPN. **a** GRCh38 + decoyJRGv0 improved the alignment around the *ALG1L2* gene region. Some of the sequence reads that mapped to *ALG1L2* when the reference was GRCh38 were mapped to the decoy sequence when decoyJRGv0 was added to the reference. **b** Multiple alignment of a portion of the ZNF676 protein from a chimpanzee, GRCh38, and JRGv0. **c** Suggested functional variants of a novel insertion, TMMINS2292, in the *ZNF676* gene coding region

Figure 4a shows the alignment improvements when using GRCh38 and decoyJRGv0 in the gene body of *ALG1* (*CD96* and *ADRA1B* are shown in Supplementary Fig. 16). Many incorrect alignments in GRCh38, spanning from the promoter regions of *ALG1* to the near region of the second exon (chr3: 130,081 to 130,090 kb), were filtered, and 14 invalid SNVs were removed using GRCh38 and decoyJRGv0. In JRGv0, the sequenced reads aligned to decoyJRGv0 were correctly aligned to TMMINS3279 (chr3: 75,289,663 in GRCh38 coordinate), TMMINS4733 (chr8: 127,521,605) and TMMINS5108 (chrX: 67,762,929; Supplementary Fig. 17).

### Variants in TMMINSs among the Japanese population

Long insertions are difficult to detect using a short-read sequencer. However, like in the former 1KJPN alignment analysis, once long insertions are discovered, alignments of short sequence reads to these regions are possible. In TMMINS regions, SNVs, short insertions and deletions should exist, similar to other regions in the international reference genome. An estimated 56,846 SNVs were detected in the TMMINS regions.

Some of the insertions were detected around the transcription start sites, intronic regions and exonic regions (Table 1c). The *METTL21C* gene had a 2093-bp insertion (TMMINS1406) in the 5′-untranslated region (UTR), the *MEIS3* gene had a 4389-bp insertion (TMMINS2339) ten bases before the transcription start site, and the last exon of the *ZNF676* gene had a 252-bp insertion (TMMINS2292). All of these insertions were also detected in the Denisovan, Neanderthal, and chimpanzee genomes.

Variants located in protein coding regions may have some biological effects. TMMINS2292 was located in the third exon of the *ZNF676* gene, with variants in the coding region (Fig. 4b). TMMINS2292 (252-bp insertion) encodes 84 amino acids, adding three zinc finger motifs (21 amino acids for each zinc finger motif) to *ZNF676* in GRCh38. This insertion was shared in the 1KJPN and i1000g populations (Supplementary Fig. 18).

Figure 4b shows a comparative analysis between the chimpanzee reference genome and TMMINS2292 (Supplementary Fig. 19). The sequence of the detected insertion was completely conserved between the JPN00001 and chimpanzee genomes. In 1KJPN, one nonsynonymous SNP (SNP2) and two synonymous SNPs (SNP1 and SNP3) were detected (Fig. 4c). The minor allele (G) of SNP2 caused threonine to be replaced with proline in a zinc finger region encoded by TMMINS2292. Threonine is a hydrophilic amino acid, whereas proline is hydrophobic. Therefore, this variant may affect the structure and function of the protein product, consistent with the very rare MAF of SNP2 (0.00093) compared with those of SNP1 and SNP3 (0.258 and 0.353, respectively). Previous studies have reported the association of *ZNF676* with telomere length and, moreover, SNP2 is thought to affect diseases involving telomere length^47,48^.

## Discussion

### Detected insertions

High-coverage sequencing with a short-reads approach can efficiently detect SNVs, short insertions, and short and long deletions^12,49^. However, it is difficult to discover insertions longer than 100 bp using short-reads (Fig. 3a in ref. ^12^). From high-coverage sequencing with longer reads, 3,691 insertions of 100 bp or longer, totaling 2.58 million bases, were successfully discovered with clear peaks around Alus and LINEs (Fig. 1b).

Once the reliable insertions were integrated into reference assemblies, a resequencing approach with a short-read sequencer was applicable. We constructed the Japanese reference genome JRGv0 by integrating 3,691 insertions into the international reference assembly GRCh38. With a resequencing approach that aligned the high-coverage short-read data from 1070 Japanese individuals to JRGv0, 871 long insertions, which were biallelic in the Japanese data, were selected. Approximately 20% of these insertions were shared among all 1070 individuals. The mobile elements SVA, LINE1, and AluY were significantly enriched in these insertions. In interpopulation analysis that included related species, performed after the application of strict quality filtering, among 194 insertions, dozens (polymorphic area in Fig. 3b) were estimated to be derived from deletion events after branching from the chimpanzee lineage.

### Application of decoyJRG and JRG

Generally, the decoy sequence could be considered the fragment of the human genome missing from the reference genome. Thus, the addition of the decoy sequence to the reference genome will increase the proper alignment and decrease the number of mismatches and unmapped reads compared with those generated using only the reference genome.

Using decoyJRG, the decoy sequence constructed with TMMINSs, we demonstrated a reduction in the number of improper alignments in coding regions, e.g., *ALG9*, *CD96*, and *ADRA1B*. JRGv0 increased the proper alignment ratio by ~0.43% in 1KJPN and resulted in the discovery of 56,846 novel SNVs in insert regions. Among TMMINSs, the exonic insertion in *ZNF676*, which was conserved in the chimpanzee genome, had three novel SNVs, including one nonsynonymous rare variant in the Japanese population. Thus, ongoing efforts to discover long insertions and variants in these sequences are promising.

### Insertions shared with other populations

Many insertions in our analysis were also observed in other populations. These common insertions, which are observable even in archaic humans and chimpanzees, could be reasonably included in the future international reference genome. Contrary to expectations, which suggest that the whole-genome assembly from a single individual produces novel sequences unique to that individual (i.e., private variants), most of the novel insertions were polymorphic among modern human populations (Fig. 3b). Moreover, the allele frequency distributions of these insertions were similar to those of SNVs and short indels (Fig. 1e). Interestingly, the vast majority of these insertion sequences were also found in archaic humans and chimpanzees (Fig. 3b). Thus, the sequences discovered in this study represent the ancestral state, and insertions absent from the current reference genome assembly were derived from deletion events that occurred in the ancestral human population. The abundance of polymorphic novel insertions implies that there are a substantial number of undiscovered sequences, which are missing from not only the reference assembly (GRCh38) genome but also from JRGv0. The whole-genome sequence of GRCh38 was assembled by contigs from a relatively large number of individuals. If these individuals had a homozygous deletion at a locus, the sequence at that locus was missing from the reference genome assembly. This situation was more likely if the number of individuals contributing to the reference sequence (*n*_ref_) was smaller and/or if the MAF of the inserted sequence (MAF_ins_) was lower. To address this possibility, the discovery rates of novel insertions were estimated using the demographic model of the 1KJPN population^12^. When *n*_ref_ was assumed to be 10, the discovery rates were estimated to be 76.9%, 92.3%, 97.7%, 99.8%, 100%, and 100% for MAFs_ins_ of 1%, 5%, 10%, 20%, 30%, and 40%, respectively (Supplementary Fig. 20). Although these rates were nearly saturated for very common variants (~20%), more discoveries are expected for rare variants if more individuals are added. For example, the addition of one individual to the novel sequence discovery (*n*_ref_ = 11) increased the discovery rates by 1.7, 1.2, and 0.6% for MAF_ins_s of 1%, 5%, and 10%, respectively. The advances of third-generation sequencers and the decrease in sequencing cost will allow population-scale sequencing and complement these estimated population-scale long insertions and other more complex structures, such as inversions and translocations.

### Accession code

All insertions and their annotations, the Japanese reference genome JRGv1 and the decoy sequence decoyJRGv1 with some anonymization are available at our website https://jrg.megabank.tohoku.ac.jp/en. The same data will also be deposited onto the National Bioscience Database Center website (https://biosciencedbc.jp/en). JRGv1, decoyJRGv1, and sequence data will be available upon request after approval by the Ethical Committee and the Materials and Information Distribution Review Committee of the Tohoku Medical Megabank Project.

## Supplementary information

Supplementary Material

Supplementary Figures1-20

Supplementary Tables1-7

Supplementary Material 2

Supplementary Material 3

Supplementary Material 1
