# Supplementary material for: Construction of JRG (Japanese reference genome) with single-molecule real-time sequencing
Source: Hum Genome Var. 2019 Jun 7;6:27. doi: 10.1038/s41439-019-0057-7 (PMC6555796; doi:10.1038/s41439-019-0057-7)
Supplement: Supplementary file 1 — Supplementary Material [file 41439_2019_57_MOESM1_ESM.docx]

# **Supplementary Materials**

## Supplementary Fig. 1. PCA plot.

Principal component analysis (PCA) was conducted to test whether the sample was included in the Japanese population. A total of 1,070 Japanese individuals (1KJPN^12^) and the sample in this study (red arrow) were plotted. CDX (Chinese Dai in Xishuangbanna, China), CHB (Beijing, China), CHS (Southern Han Chinese), JPT (Tokyo, Japan), and KHV (Kinh in Ho Chi Minh City, Vietnam) data from the HapMap project^42,43^ were also plotted.

## Supplementary Fig. 2. Outlines of the process of the assembly of the sequence data, detection of novel insertions, and construction of JRGv0.

Raw reads were mapped to the international reference genome, GRCh38, and were separated into 24 groups corresponding to mapped chromosomes (chr1–22, chrX, and chrY). Then, assembly was performed in each group and generated contigs were aligned to GRCh38 to detect insertions in the contigs. The detected insertions were filtered and integrated from GRCh38 into JRGv0. The decoyJRGv0 was also constructed by concatenating the insertion sequences.

## Supplementary Fig. 3 Detection method of deletions

Deletions were detected with two methods, INTRA and INTER. The INTRA method detected CIGAR string counting "D" s in a continuously mapped contig as deletions. In this case, the deletion length was equal to the number of "D"s. INTER method detected gaps or overlaps from split mapping of contigs. When a contig was split and mapped to the reference, "qlen" was defined as the coordinate c (left end of the right mapped split contig) minus b (right end of the left mapped split contig) while the coordinates of the ends of the original contig were a and d (d > a). When "rlen" was defined as the length of the region between the two regions where the split contigs were mapped, the deletion length was defined as rlen minus qlen.

## Supplementary Fig. 4. Integration methods of TMMINSs from GRCh38 to construct JRGv0.

The methods were different depending on the split contig distance when mapped to GRCh38. (a) When the split contig distance was 0, the detected insertions were simply inserted into GRCh38 at the location where the contig was split. (b) When the split contig distance was greater than 0, indicating a microdeletion accompanied with insertion of retrotransposition, the region in GRCh38 between the split contig mapped regions was replaced with the detected insertion. (c) When split contig distance was less than 0, the contig had redundant sequences at the both ends of the insertions. In such cases, the set of the insertion and end sequences was integrated into GRCh38.

## Supplementary Fig. 5. The assembly, detection of long insertions and construction of the JRGv0 genome.

(a) The processes of grouping of the raw reads and assembling and mapping contigs to GRCh38 to detect novel insertions (TMMINSs). (b) Construction of JRGv0 and decoyJRGv0. JRGv0 was constructed by integrating TMMINSs with GRCh38. DecoyJRGv0 was constructed by concatenating TMMINSs. (c) The schematic diagram of selection criteria for downstream analysis and the number of selected insertions.

## Supplementary Fig. 6. Performance of the assembly.

(a) Comparison of the total length of the contigs, GRCh38 scaffolds (including Ns) and GRCh38 contigs (without Ns). (b) Ratio of the total contig length to GRCh38 scaffolds and contigs.

## Supplementary Fig. 7. Dot plots comparing the assembled contigs and GRCh38 for each chromosome.

X-axes indicate the chromosome coordinates of GRCh38. Y-axes indicate contigs. Blue and pink regions display centromeres and gap regions in GRCh38, respectively.

## Supplementary Fig. 8. Size distribution of detected insertions and deletions.

## Distribution of the lengths of 3,691 TMMINSs (right) and 4,040 deletions (left). Both showed two prominent peaks correspond to Alus and LINEs.

## Supplementary Fig. 9. Allele frequencies and the number of shared TMMINSs with AK1.

## (a) Numbers of TMMINSs shared with AK1 within different distances, 0 bp, 50 bp and 100 bp each other. (b) The correlation of the insertion length to 1,873 shared insertions between TMMINSs (X-axis) and AK1 (Y-axis) (within 50bp from the insertion point). (c) The correlation of allele frequencies of 871 biallelic TMMINSs in 1KJPN (X-axis) and the number of shared insertions between JPN00001 and AK1 (within 50bp from the insertion point) (Y-axis).

## Supplementary Fig. 10. Other patterns of read coverage distributions of TMMINSs in 1KJPN and other populations.

(a) A less common sequence, mainly shared in East Asian and CLM populations, compared with (a) in Fig. 3. (b) An almost monomorphic sequence in the East Asian population, but not in other populations.

## Supplementary Fig. 11. Genotype frequencies of PCR validated 10 TMMINSs in 1KJPN and an alignment snapshot in JPN00001 and NA12878.

Left panel: genotype frequencies of 1KJPN (gray), JPN00001 (red dot) and NA12878 (blue dot). Right panel: iGV snapshot of JPN00001 (top) and NA12878 (bottom) to each PCR validated insertion.

## Supplementary Fig. 12. Repeat classes of PCR validated 10 TMMINSs.

For each PCR validated insertion, the length for repeat classes, i.e. LINE, nonrepeat, LTR, SINE, Satellite and DNA, and their ratio to the total insertion length are shown.

## Supplementary Fig. 13. Length distribution of the shared insertions in 1KJPN and i1000g.

The length distribution (X-axis) and counts (Y-axis) of shared insertions in 1KJPN and all i1000g population length distribution.

## Supplementary Fig. 14 Alignment performance of decoyJRGv0 and JRGv0 using Bowtie2.

(a-c) Box plots showing the alignment ratios of short reads to the three references, GRCh38, GRCh38 + decoyJRGv0 and JRGv0, respectively. (a) Total alignment ratios. (b) Paired reads alignment ratios. (c) Single reads alignment ratios. (d) Proper read alignment ratios. (e-f) The relative alignment performance to GRCh37. (e) Total alignment ratios. (f) Paired reads alignment ratios. (g) Singleton reads alignment ratios. (h) Proper read alignment ratios.

## Supplementary Fig. 15 Alignment performance of decoyJRGv0 and JRGv0 using BWA-MEM.

(a-c) Box plots showing the alignment ratios of short reads to the three references, GRCh38, GRCh38 + decoyJRGv0 and JRGv0, respectively. (a) Total alignment ratios. (b) Paired reads alignment ratios. (c) Single reads alignment ratios. (d) Proper read alignment ratios. (e-f) The relative alignment performance to GRCh37. (e) Total alignment ratios. (f) Paired reads alignment ratios. (g) Singleton reads alignment ratios. (h) Proper read alignment ratios.

## Supplementary Fig. 16. Improved alignments using decoyJRGv0 in *ALG1, CD96,* and *ADRA1B* regions.

Comparison of the mapping results to only GRCh38 and GRCh38 + decoyJRGv0 in two gene regions. A considerable number of misaligned mapped reads were aligned to decoyJRGv0 (i.e. not aligned to the sequence in GRCh38 chromosomes) and the number of false positive SNVs was reduced. (a) *ADRA1B* region and (b) *CD96* region.

## Supplementary Fig. 17. Correct alignments using JRGv0 for the incorrect reads in *ADRA1B*, *ALG1L2,* and *CD96* regions.

Using JRGv0 as a reference, misaligned reads when the reference was GRCh38 mapped to the TMMINS regions correctly. (a) *ADRA1B* region, (b) *ALG1L2* region and (c) *CD96* region.

## Supplementary Fig. 18. Read coverage distributions of TMMINS2292 in 1KJPN and other populations.

TMMINS2292, which is located in the last exon of *ZNF676*, was shared between 1KJPN and i1000g populations. (a) Call rate of TMMINS2292 in each population. Green: ratio of the individuals called as "existing" or "not existing", blue: existence was not determined. (b) Copy number of TMMINS2292 in individuals for whom the existence was called in each population. All of the individuals called had two copies. (c) Normalized coverage distribution of TMMINS2292 in 1KJPN population. It had a single peak around coverage = 2. (d) Normalized coverage distribution of TMMINS2292 in the i1000g population. It also had a single peak around coverage = 2.

## Supplementary Fig. 19. Comparison of the ZNF676 gene among chimpanzee, GRCh38 and JRGv0.

Multiple alignments of all amino acids in the ZNF676 protein of the chimpanzee reference assembly, GRCh38 and JRGv0. Asterisks indicate the conserved amino acids.

## Supplementary Fig. 20. Estimated discovery rates of novel sequences.

The rates of novel sequence discovery (Y-axis) are plotted against the number of samples for the discovery study (X-axis). These rates are plotted using different colors according to the minimum minor allele frequency of undiscovered sequences among a population.

## Supplementary Table 1 Analyzed individuals and populations in the international 1000 genomes.

## Supplementary Table 2 Primer list for PCR validation.

## Supplementary Table 3. Statistics of the assembly.

## Supplementary Table 4 Comparison of the assembled contigs with published datasets.

## Supplementary Table 5 Estimated allele frequency of 871 biallelic TMMINSs in international populations.

## Supplementary Table 6 Results of validation for 10 TMMINSs by short read mapping and PCR.

## Supplementary Table 7 Features of 871 biallelic TMMINSs for 1KJPN, JPT and Korean (AK1).

## Supplementary Material 1. Depth distribution of TMMINSs in 1KJPN.

## Supplementary Material 2. Alignment performance of 1KJPN individuals, JRG1 and NA12878

## Supplementary Material 3. TMMINS information.
