# Supplementary material for: Construction of JRG (Japanese reference genome) with single-molecule real-time sequencing
Source: Hum Genome Var. 2019 Jun 7;6:27. doi: 10.1038/s41439-019-0057-7 (PMC6555796; doi:10.1038/s41439-019-0057-7)
Supplement: Supplementary file 2 — Supplementary Figures1-20 [file 41439_2019_57_MOESM2_ESM.pdf]

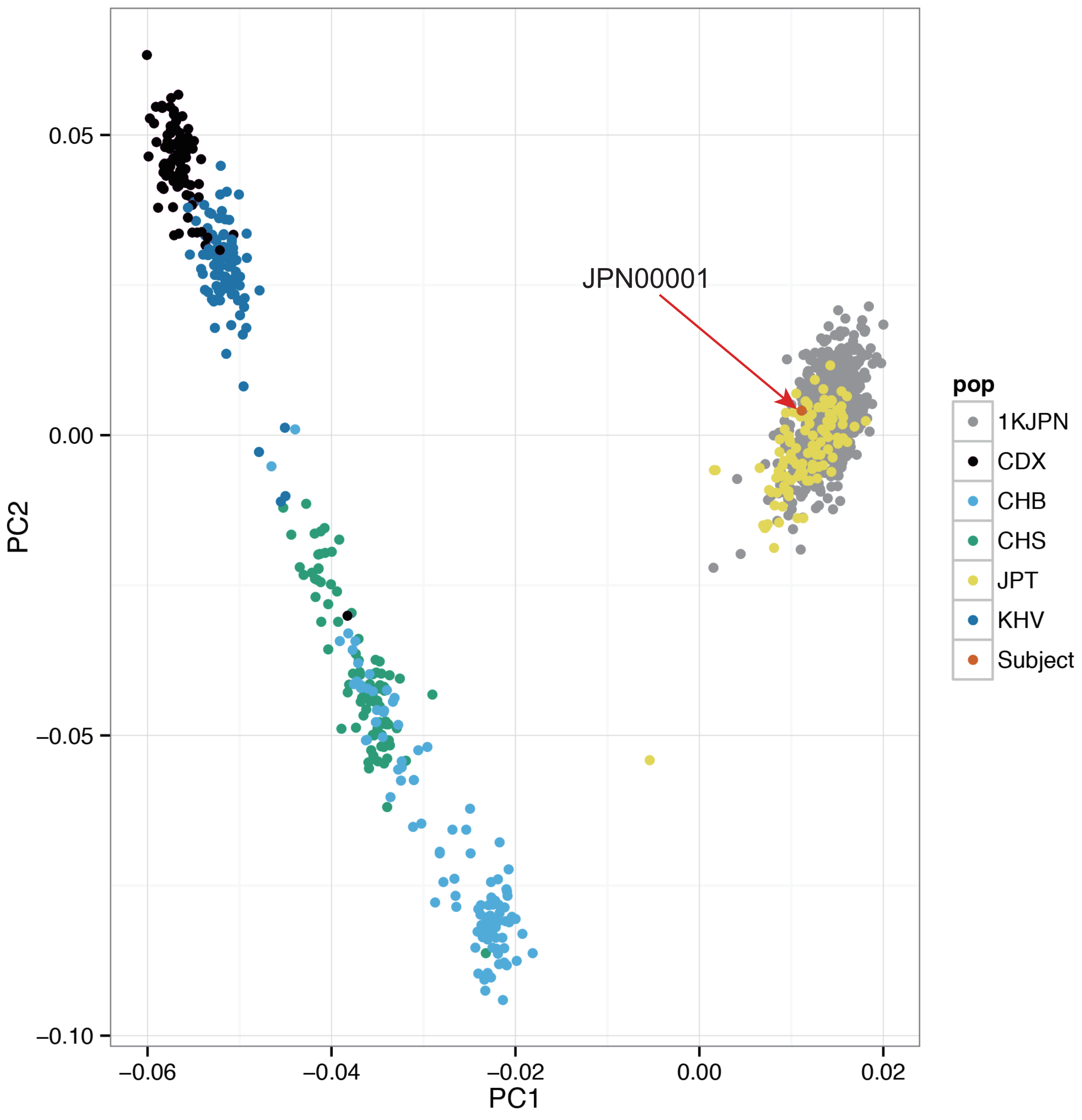

Supplementary Fig. 1. PCA plot. Principal component analysis (PCA) was conducted to test whether the sample was included in the Japanese population. A total of 1,070 Japanese individuals (1KJPN12) and the sample in this study (red arrow) were plotted. CDX, CHB, CHS, JPT, and KHV data from the HapMap project36,37 were also plotted.

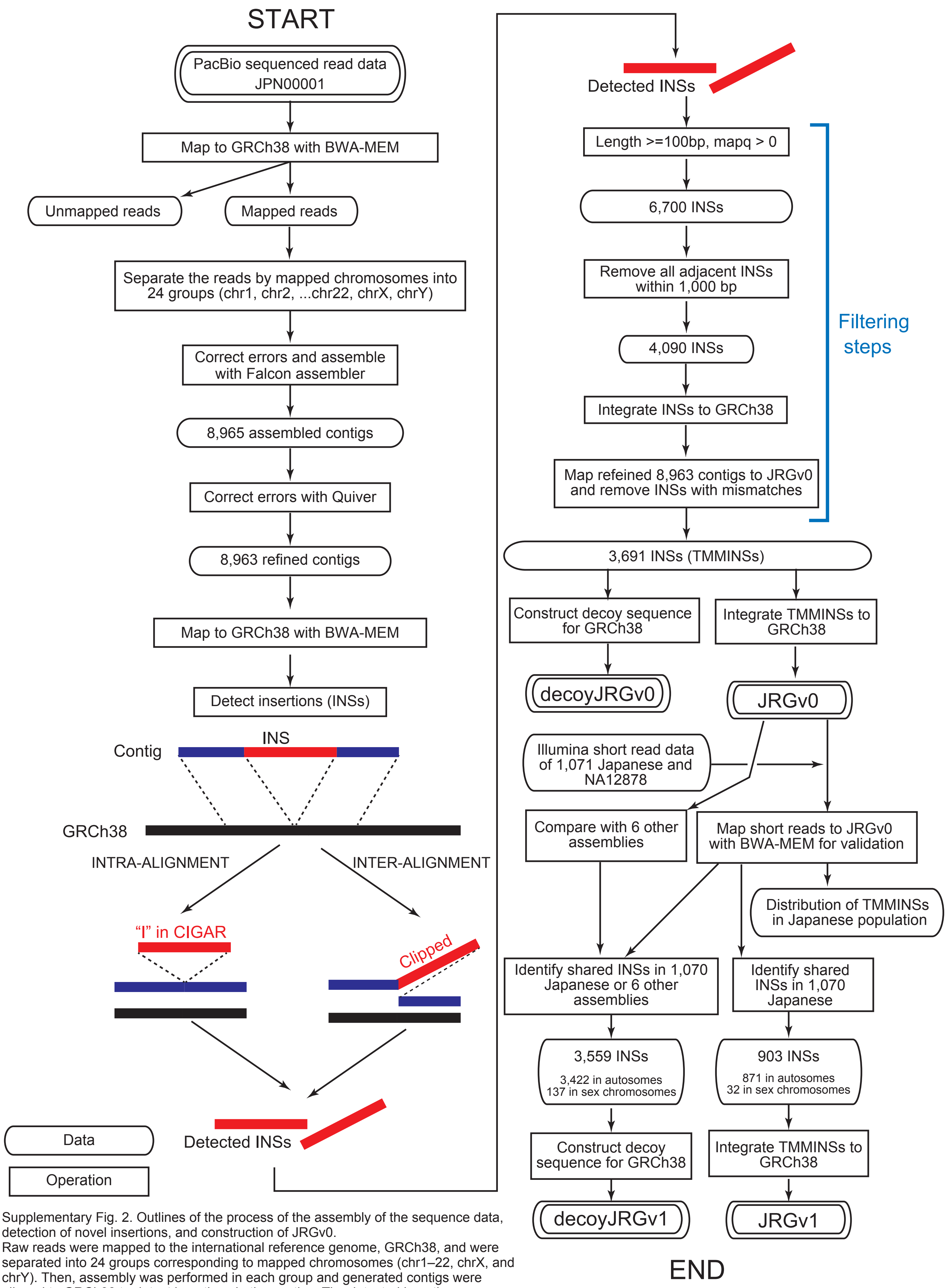

Supplementary Fig. 2. Outlines of the process of the assembly of the sequence data, detection of novel insertions, and construction of JRGv0.
Raw reads were mapped to the international reference genome, GRCh38, and were separated into 24 groups corresponding to mapped chromosomes (chr1–22, chrX, and chrY). Then, assembly was performed in each group and generated contigs were aligned to GRCh38 to detect insertions in the contigs. The detected insertions were filtered and integrated from GRCh38 into JRGv0. The decoyJRGv0 was also constructed by concatenating the insertion sequences.

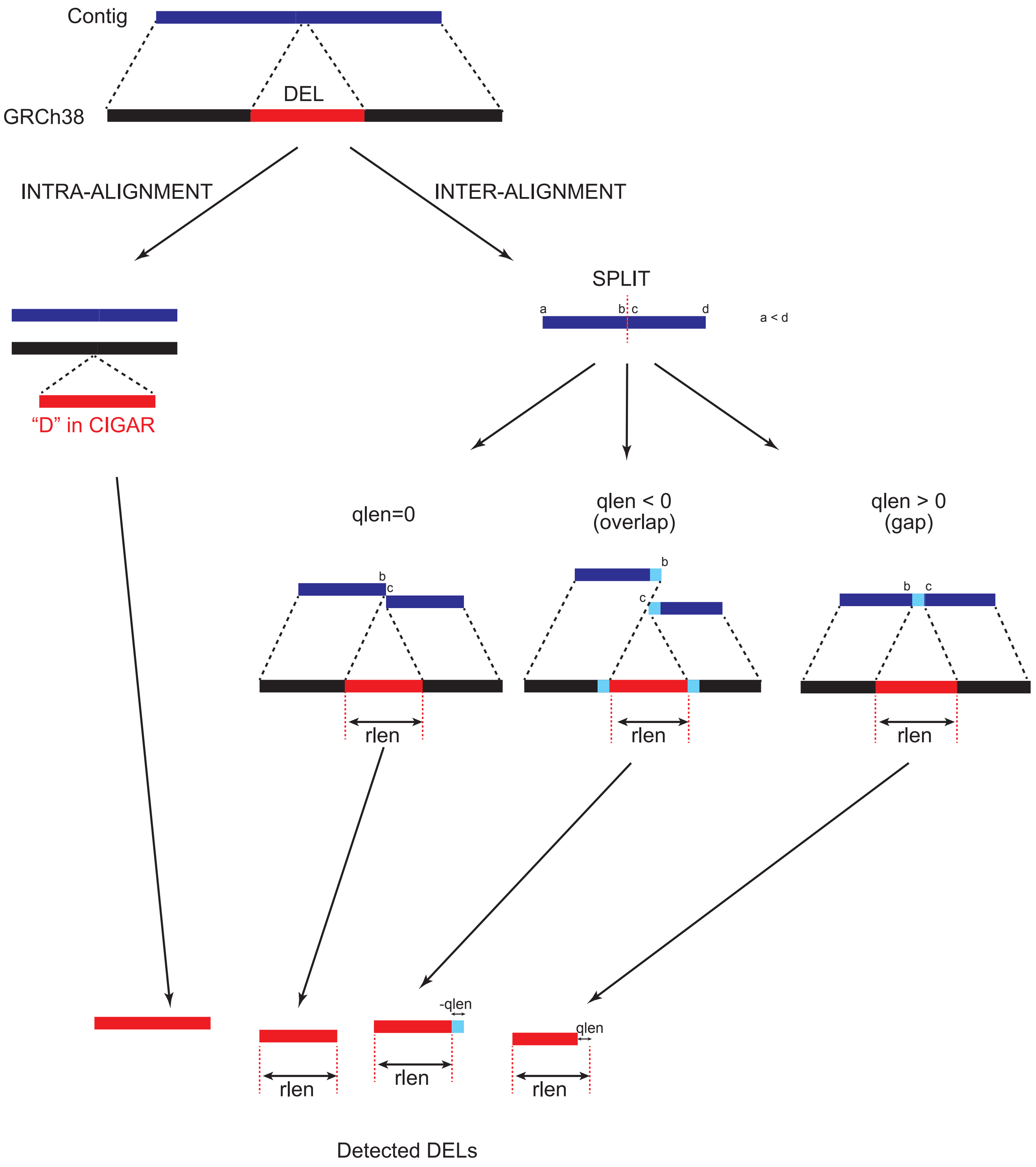

Supplementary Fig. 3 Detection method of deletions

Deletions were detected with two methods, INTRA and INTER. The INTRA method detected CIGAR string counting "D" s in a continuously mapped contig as deletions. In this case, the deletion length was equal to the number of "D"s. INTER method detected gaps or overlaps from split mapping of contigs. When a contig was split and mapped to the reference, "qlen" was defined as the coordinate c (left end of the right mapped split contig) minus b (right end of the left mapped split contig) while the coordinates of the ends of the original contig were a and d (d > a). When "rlen" was defined as the length of the region between the two regions where the split contigs were mapped, the deletion length was defined as rlen minus qlen.

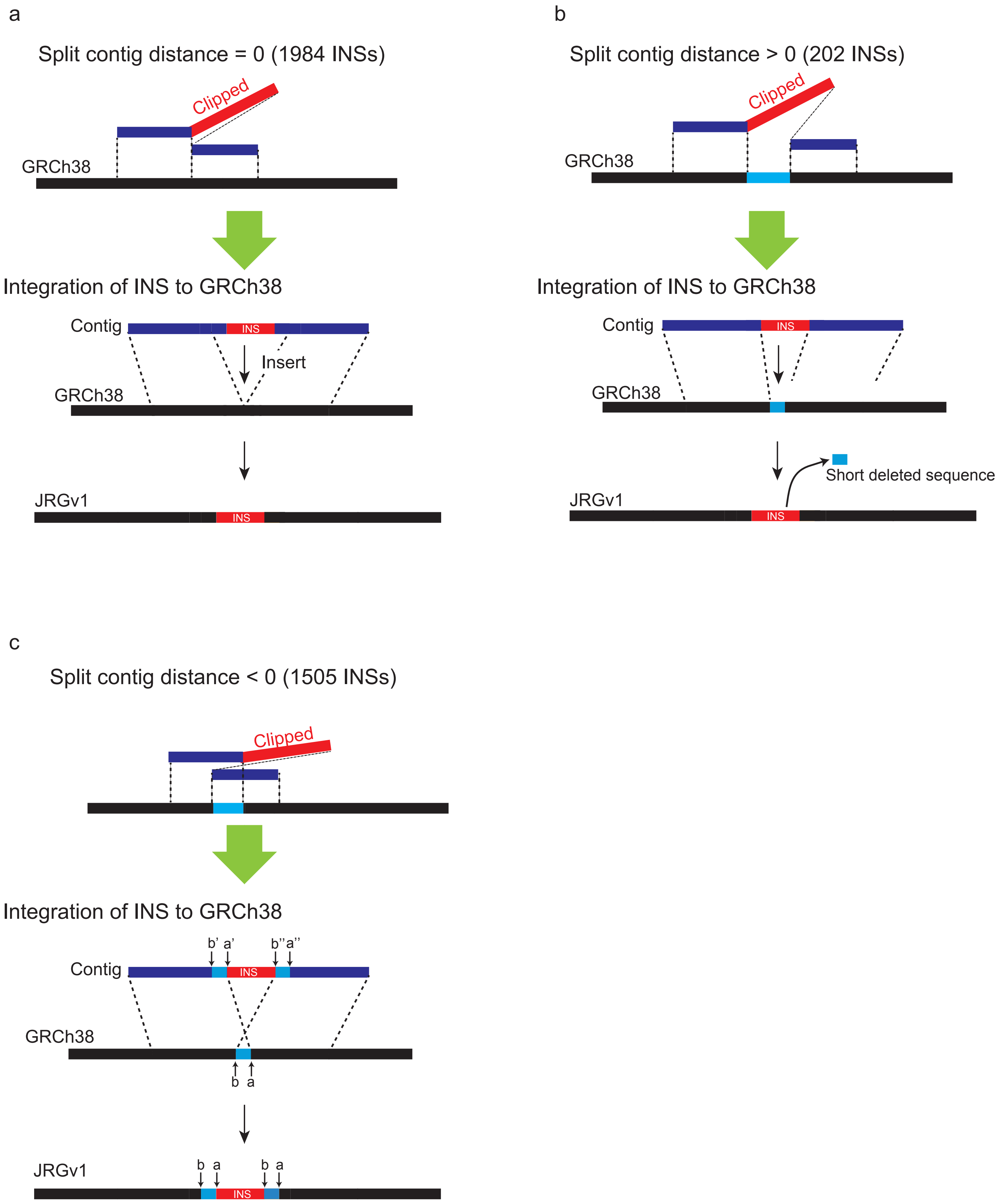

Supplementary Fig. 4. Integration methods of TMMINSs from GRCh38 to construct JRGv1.
The methods were different depending on the split contig distance when mapped to GRCh38. (a) When the split contig distance was 0, the detected insertions were simply inserted into GRCh38 at the location where the contig was split. (b) When the split contig distance was greater than 0, indicating a microdeletion accompanied with insertion of retrotransposition, the region in GRCh38 between the split contig mapped region was replaced with the detected insertion. (c) When split contig distance was less than 0, the contig had redundant sequences at the both ends of the insertions. In such cases, the set of the insertion and end sequences was integrated into GRCh38.

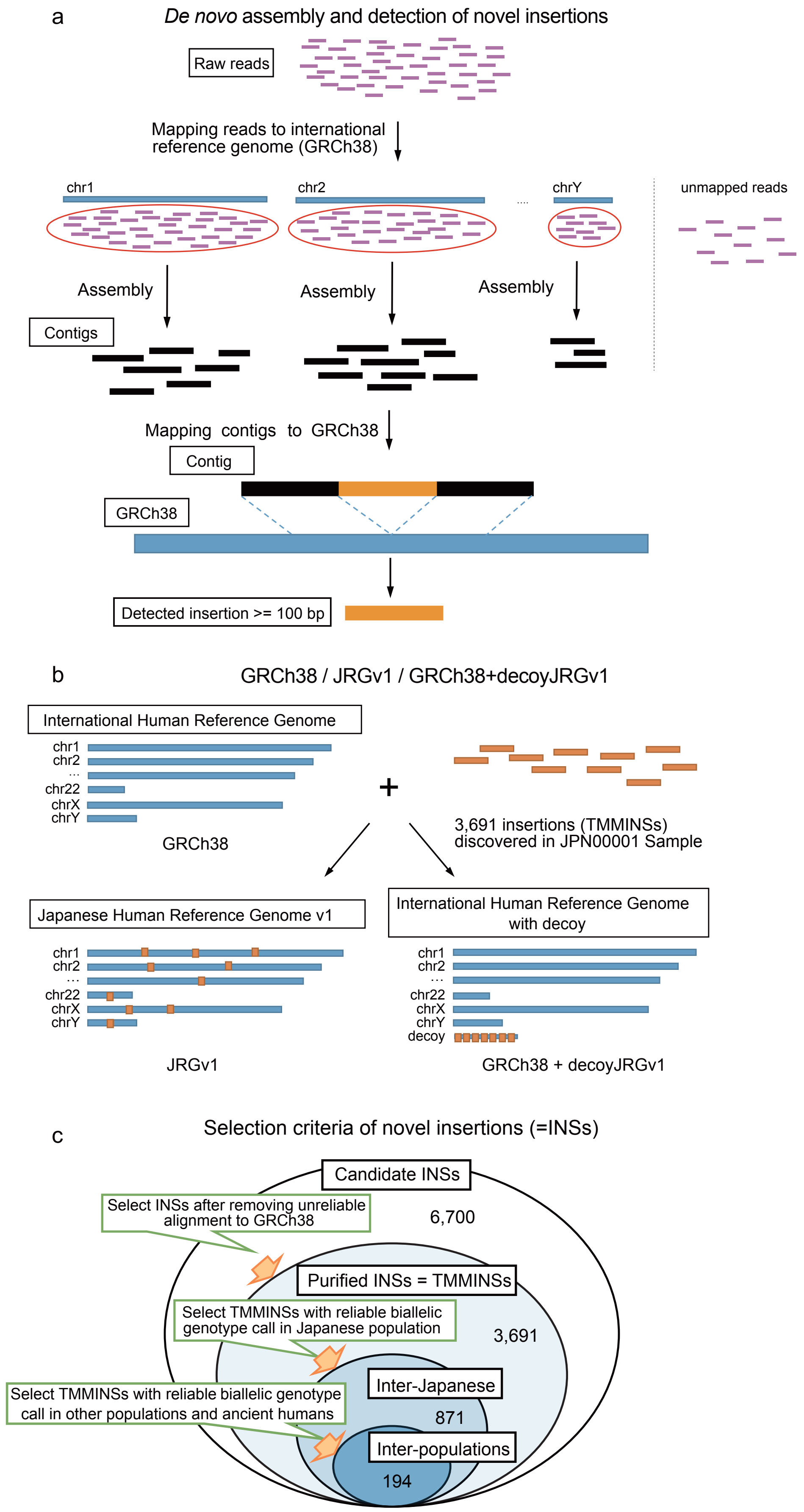

Supplementary Fig. 5. The de novo assembly detection of long insertions and construction of the JRGv1 genome. (a) The processes of grouping of the raw reads and assembling and mapping contigs to GRCh38 to detect novel insertions (TMMINSs). (b) Construction of JRGv1 and decoyJRGv1. JRGv1 was constructed by integrating TMMINSs with GRCh38. DecoyJRGv1 was constructed by concatenating TMMINSs. (c) The schematic diagram of selection criteria for downstream analysis and the number of selected insertions.

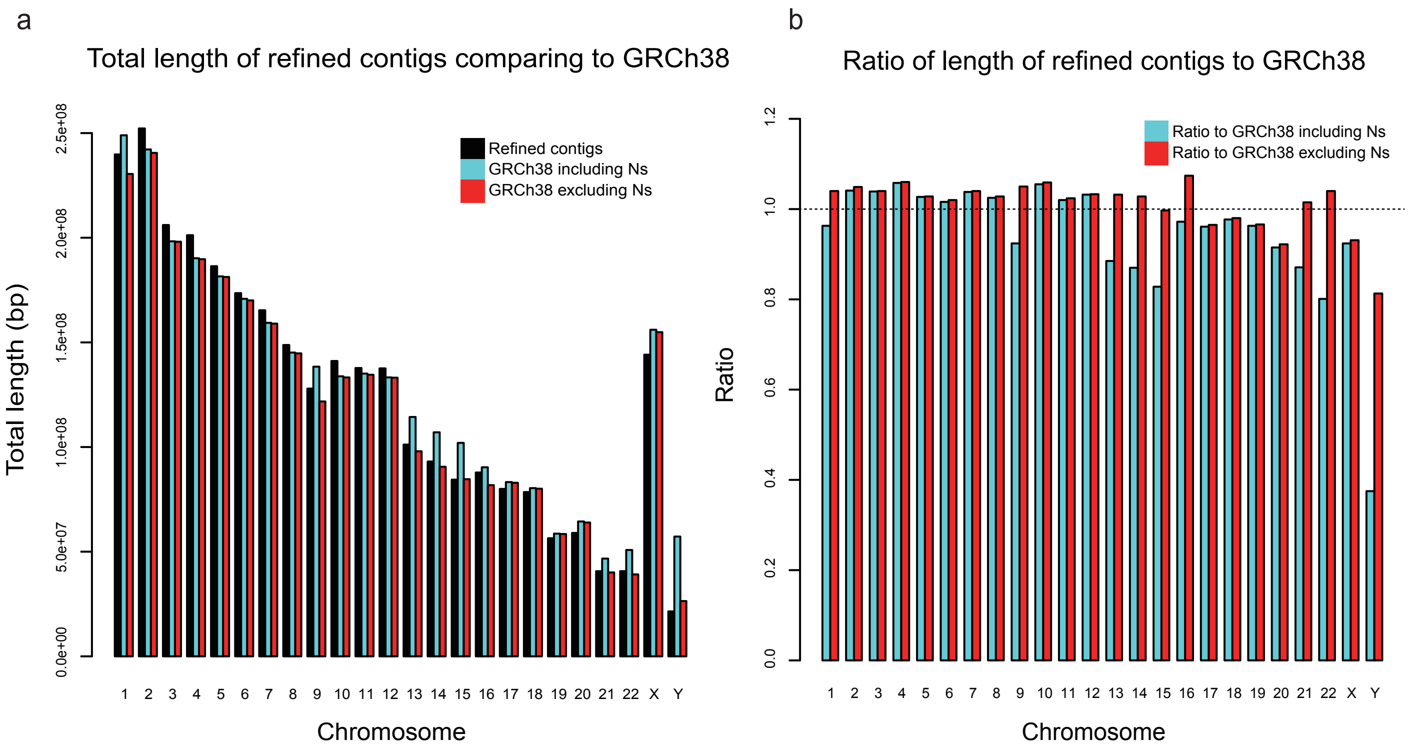

Supplementary Fig. 6. Performance of de novo assembly. (a) Comparison of the total length of the contigs, GRCh38 scaffolds (including Ns) and GRCh38 contigs (without Ns). (b) Ratio of the total contig length to GRCh38 scaffolds and contigs.

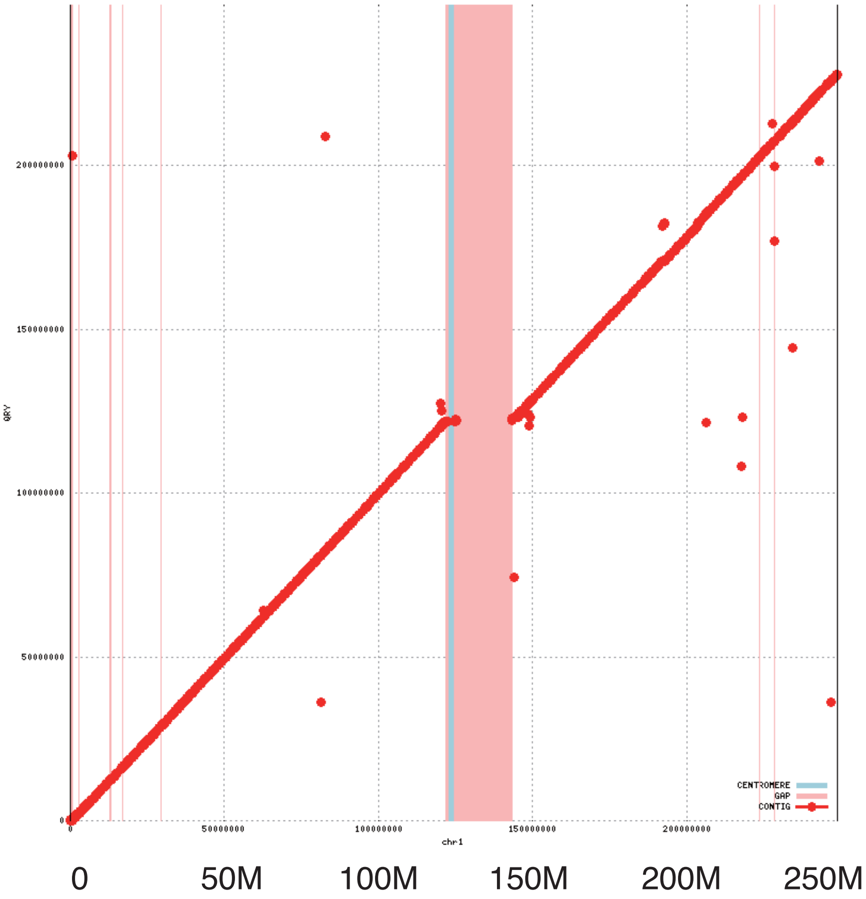

Chr 1

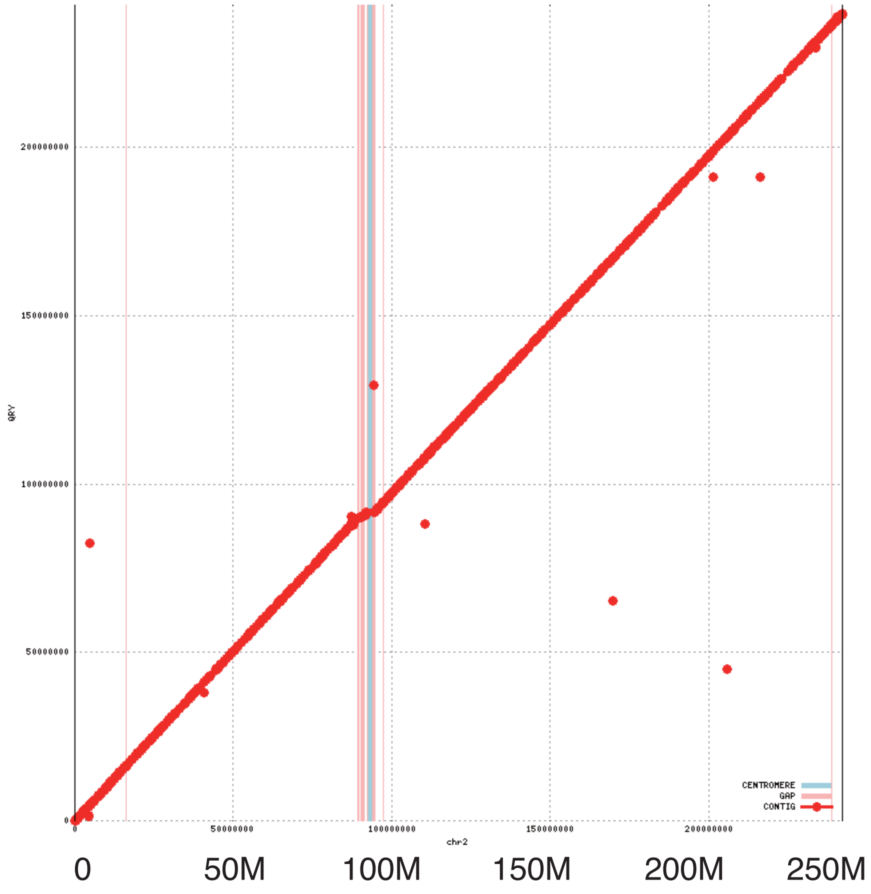

Chr 2

Supplementary Fig. 7. Dot plots comparing the assembled contigs and GRCh38 for each chromosome. X-axes indicate the chromosome coordinates of GRCh38. Y-axes indicate contigs. Blue and pink regions display centromeres and gap regions in GRCh38, respectively.

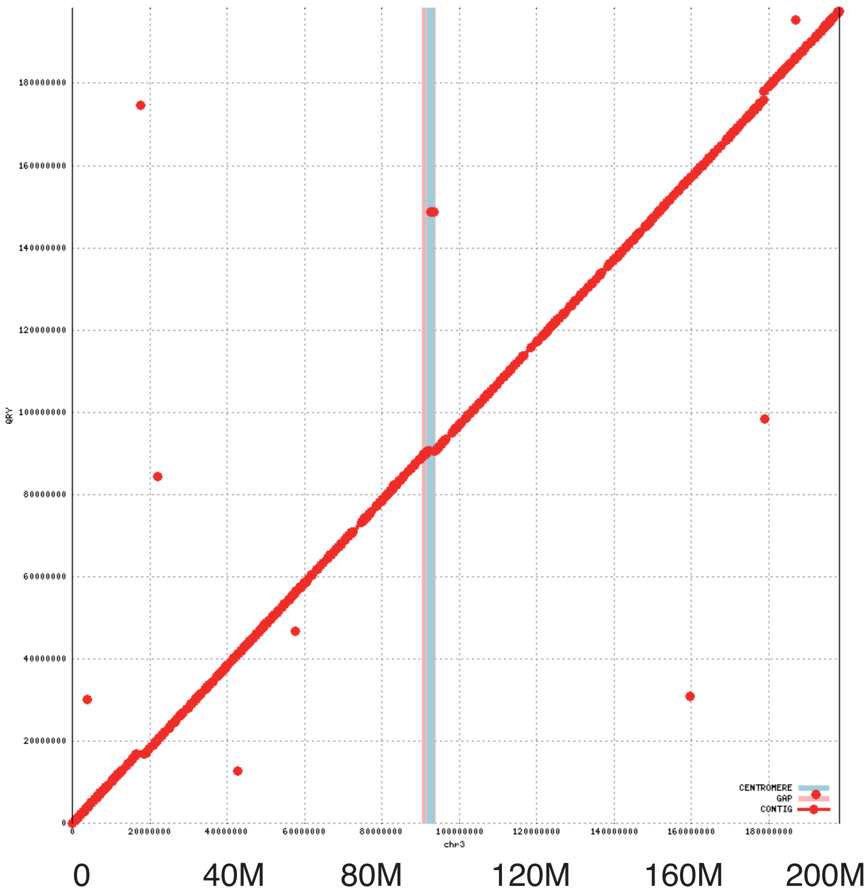
180000000
160000000
140000000
120000000
100000000
80000000
60000000
40000000
20000000
0
QRY
0
20000000
40000000
60000000
80000000
100000000
120000000
140000000
160000000
180000000
chr3
CENTROMERE
GAP
CONTIG
0
40M
80M
120M
160M
200M

Chr 3

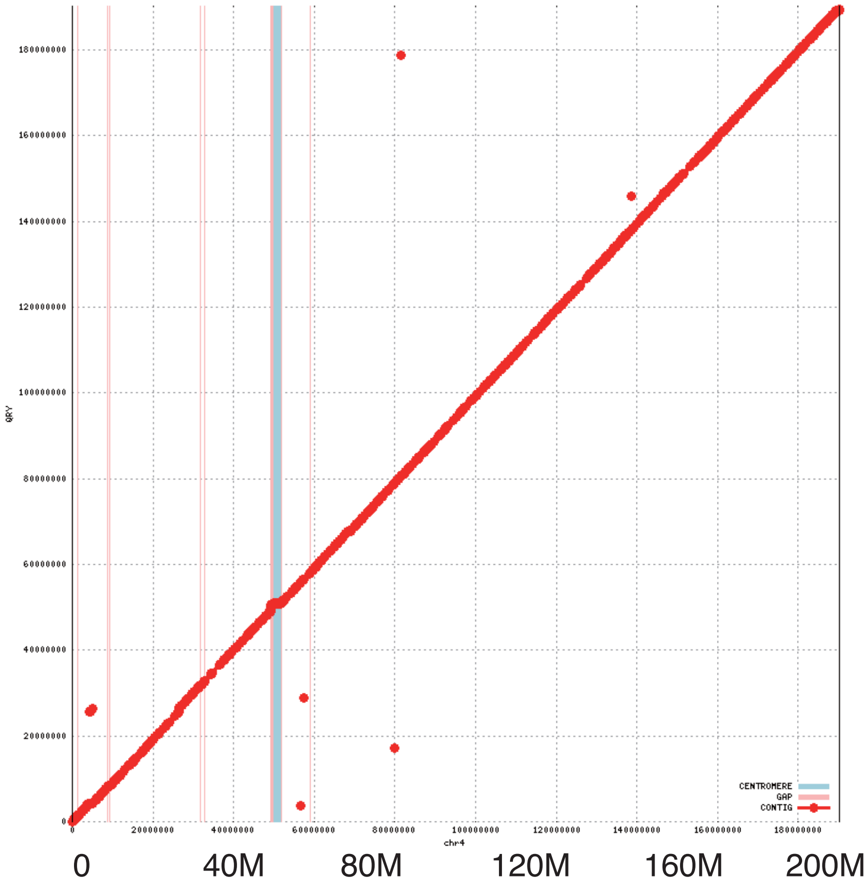
180000000
160000000
140000000
120000000
100000000
80000000
60000000
40000000
20000000
0
QRY
0
20000000
40000000
60000000
80000000
100000000
120000000
140000000
160000000
180000000
chr4
CENTROMERE
GAP
CONTIG
0
40M
80M
120M
160M
200M

Chr 4

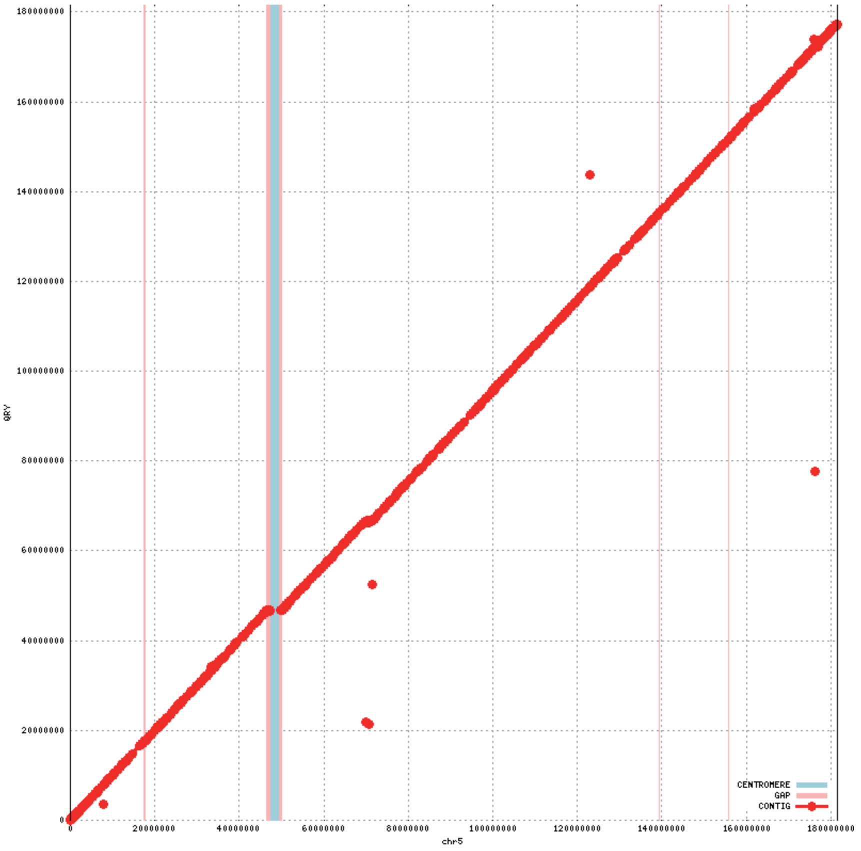

0 40M 80M 120M 160M

Chr 5

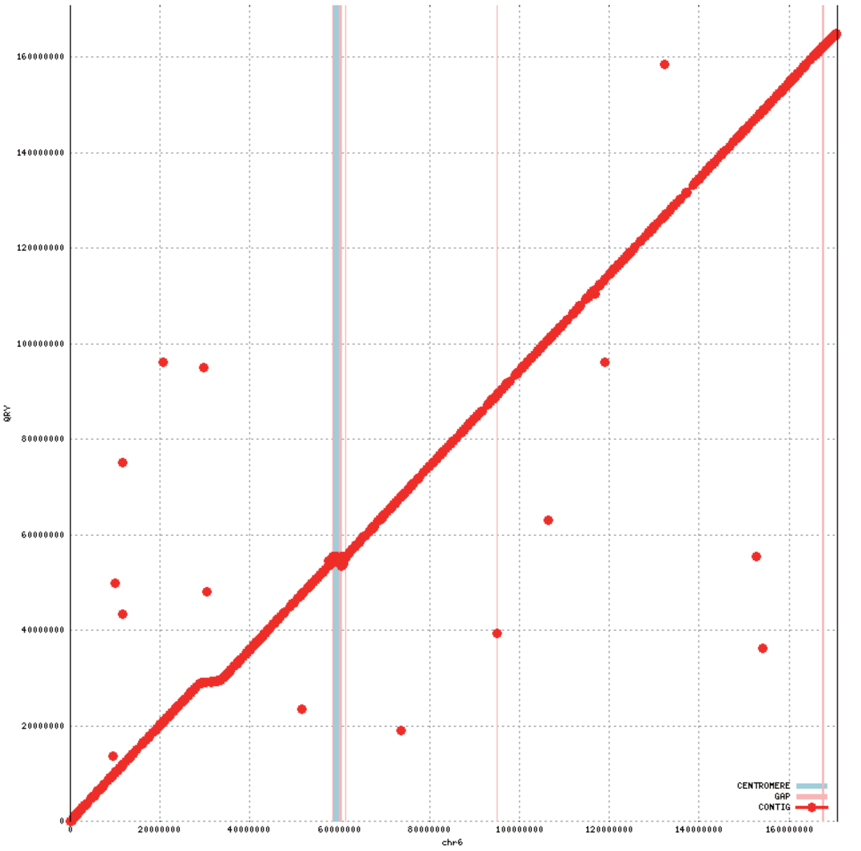

0 40M 80M 120M 160M

Chr 6

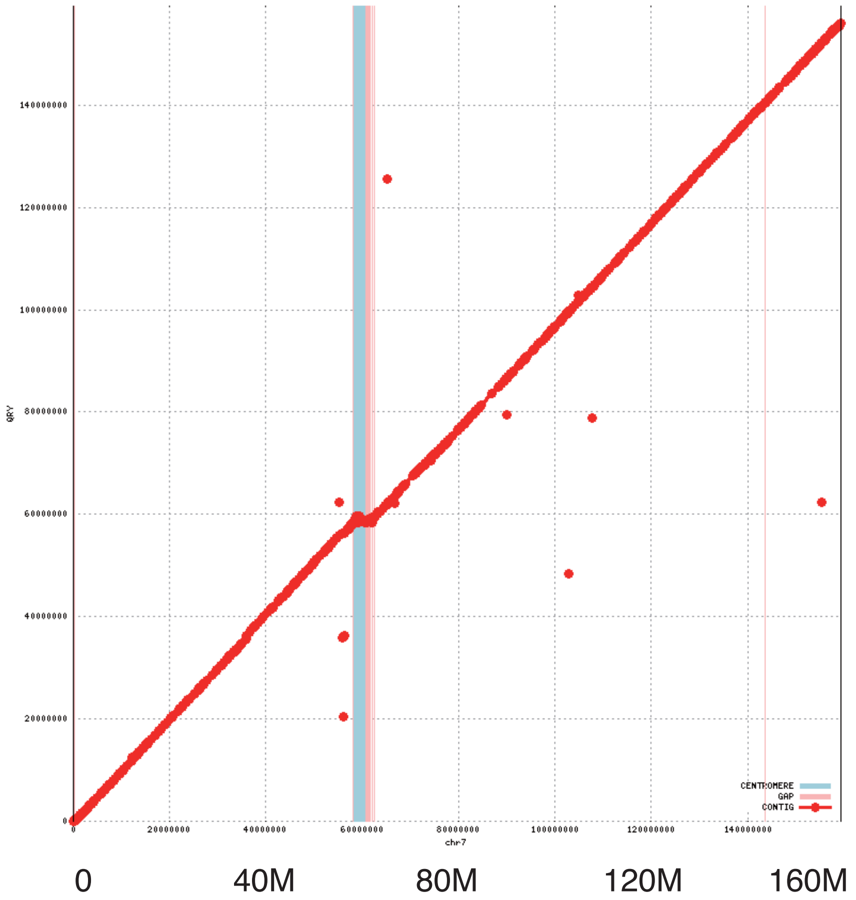

CENTROMERE
GAP
CONTIG
QRY
chr7
0
40M
80M
120M
160M

Chr 7

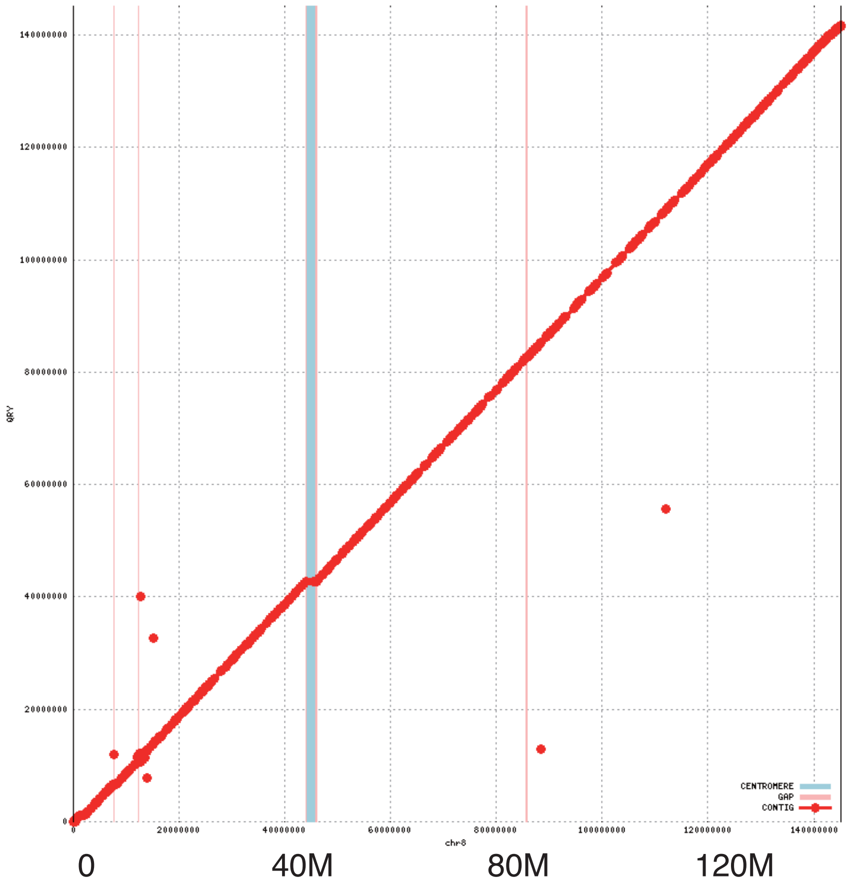

CENTROMERE
GAP
CONTIG
QRY
chr8
0
40M
80M
120M

Chr 8

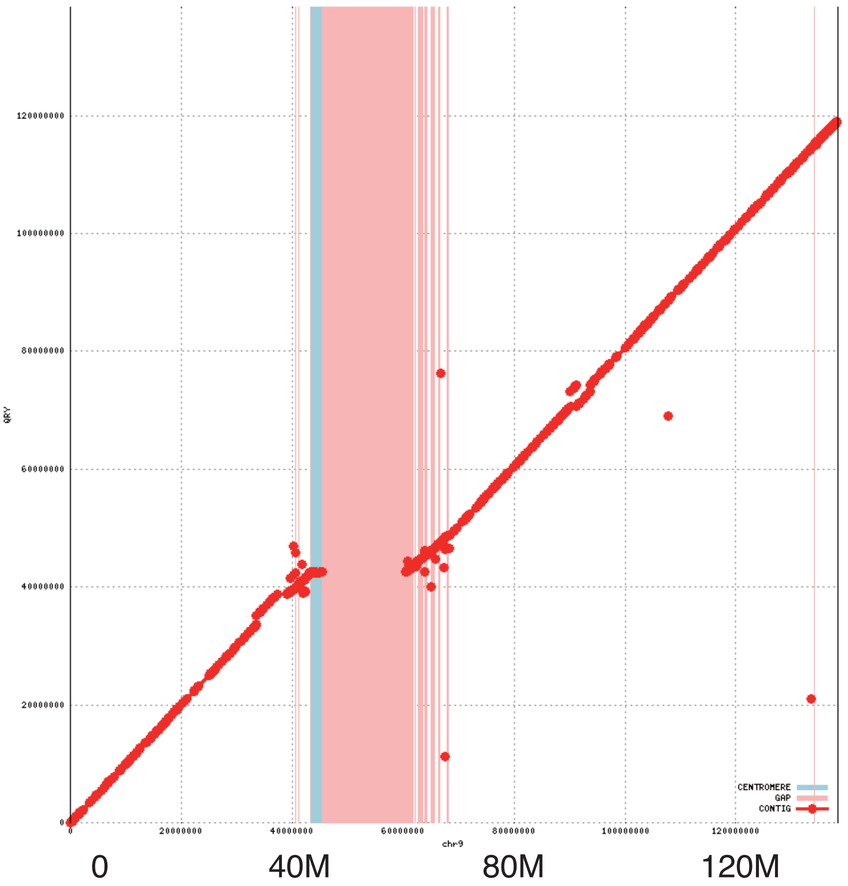

120000000
100000000
80000000
60000000
40000000
20000000
QRY
20000000
40000000
60000000
80000000
100000000
120000000
chr9
CENTROMERE
GAP
CONTIG
0
40M
80M
120M

Chr 9

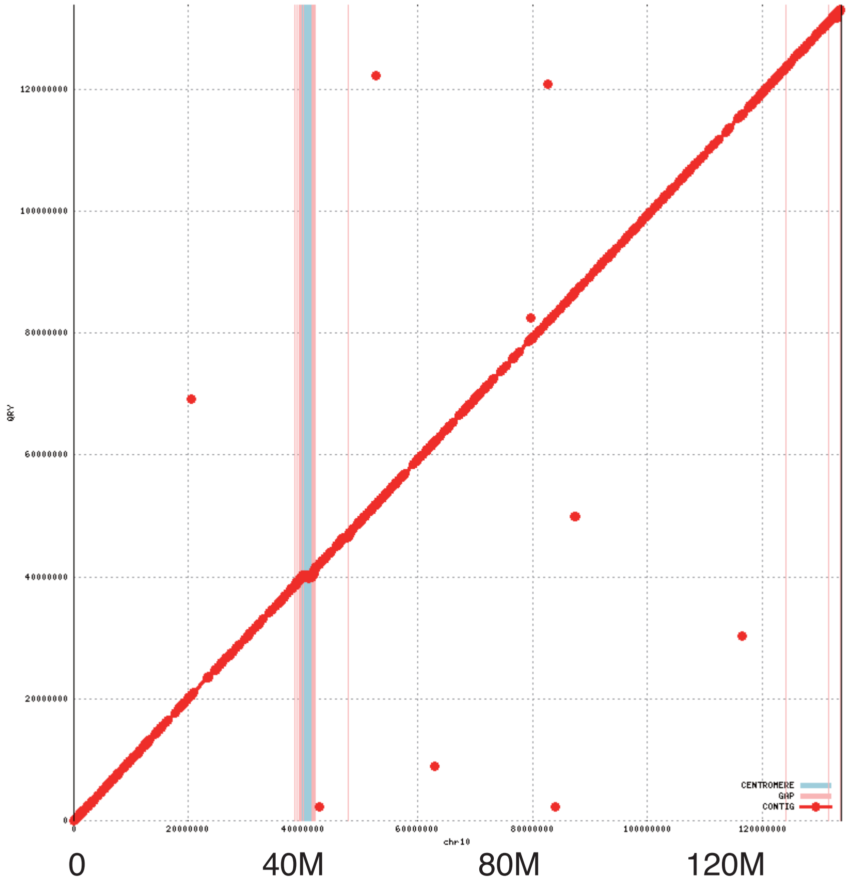

120000000
100000000
80000000
60000000
40000000
20000000
QRY
20000000
40000000
60000000
80000000
100000000
120000000
chr10
CENTROMERE
GAP
CONTIG
0
40M
80M
120M

Chr 10

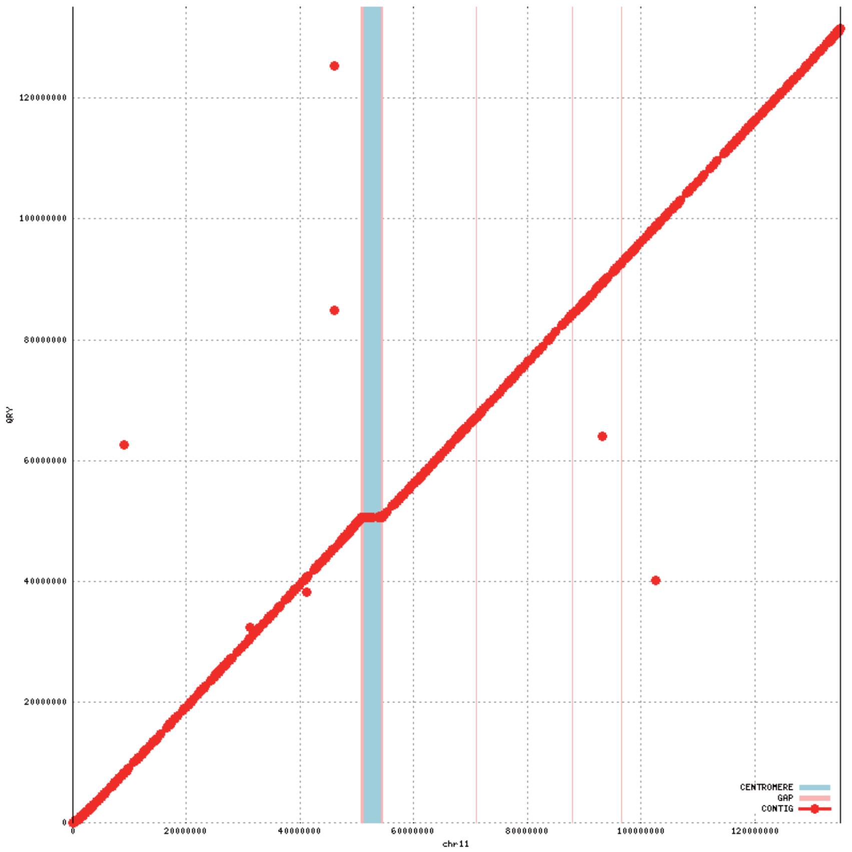

0 40M 80M 120M

Chr 11

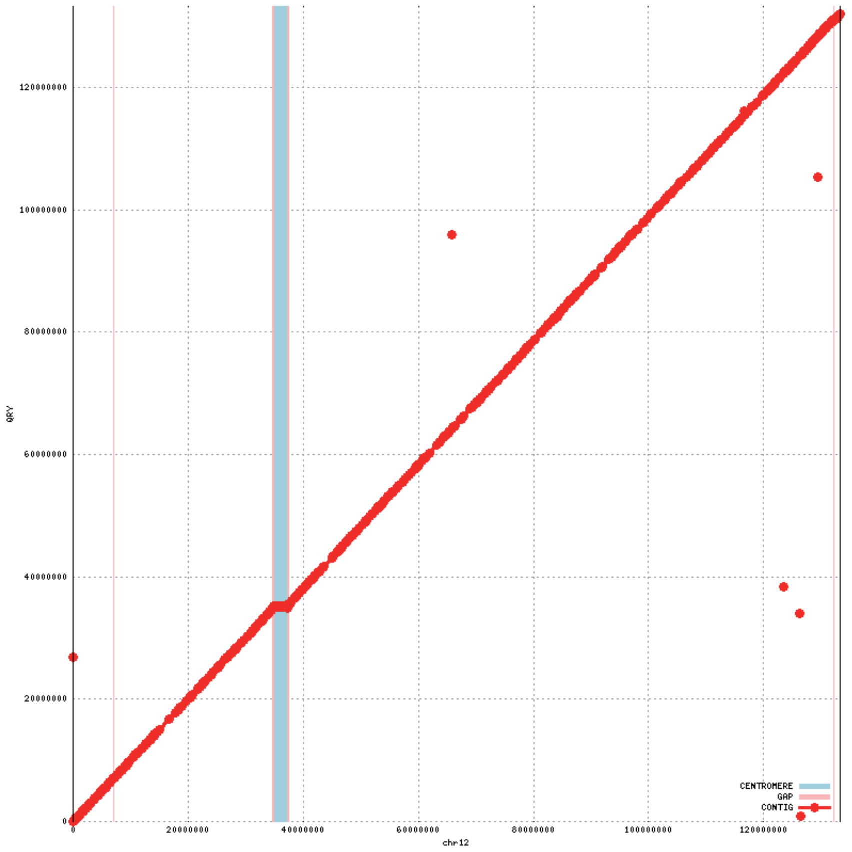

0 40M 80M 120M

Chr 12

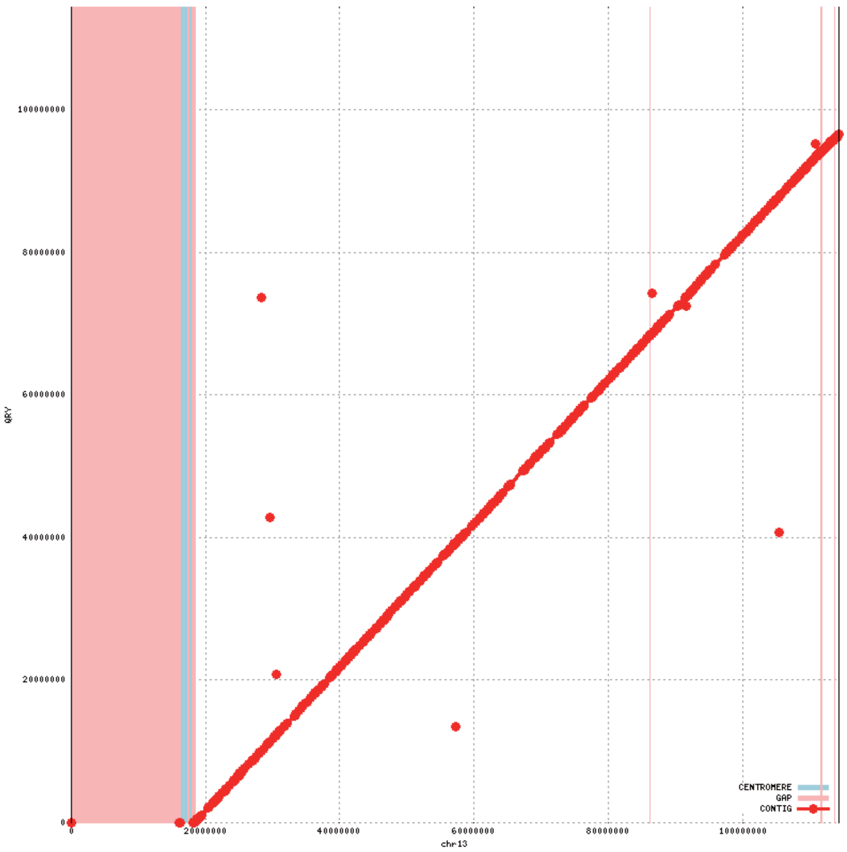

0 40M 80M 120M

Chr 13

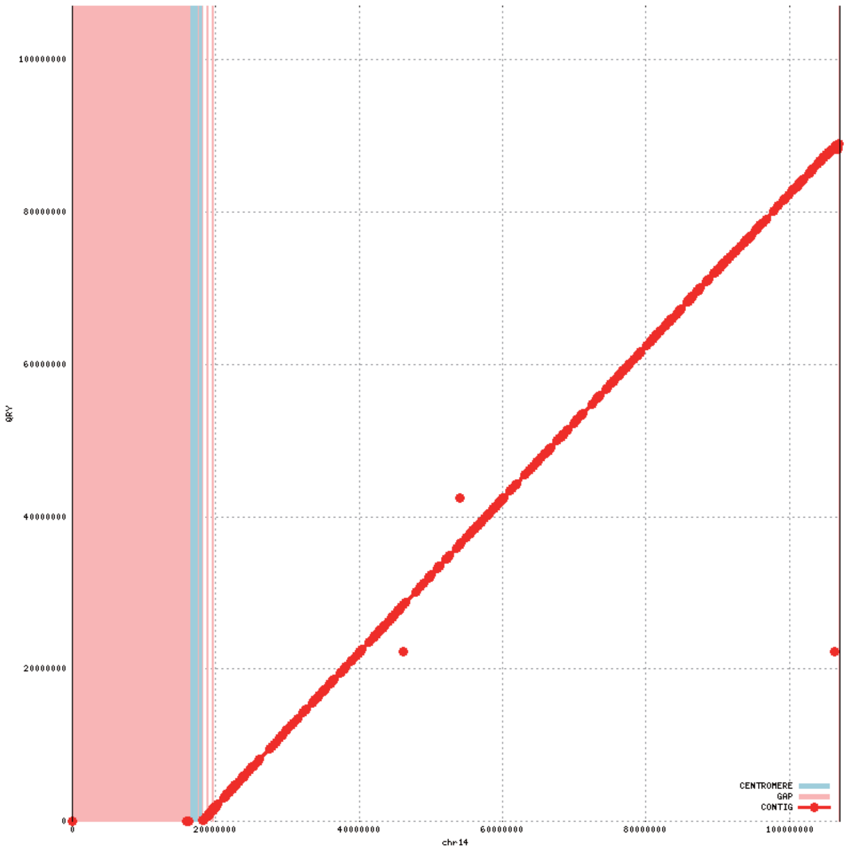

0 40M 80M

Chr 14

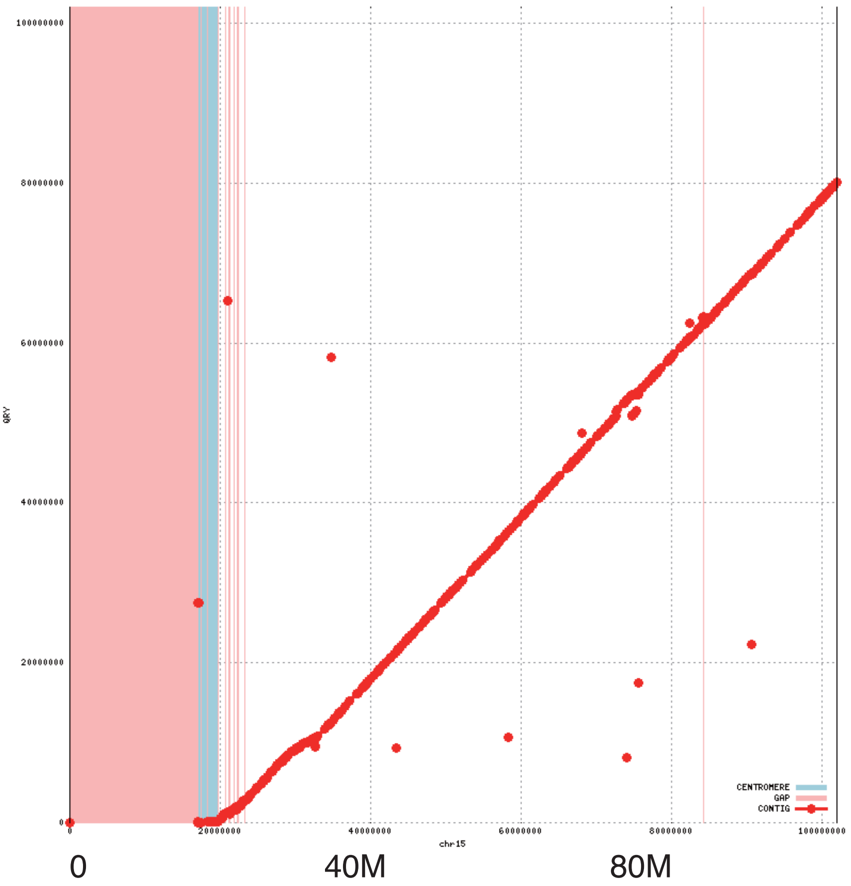

CENTROMERE
GAP
CONTIG
QRY
chr15
0
40M
80M

Chr 15

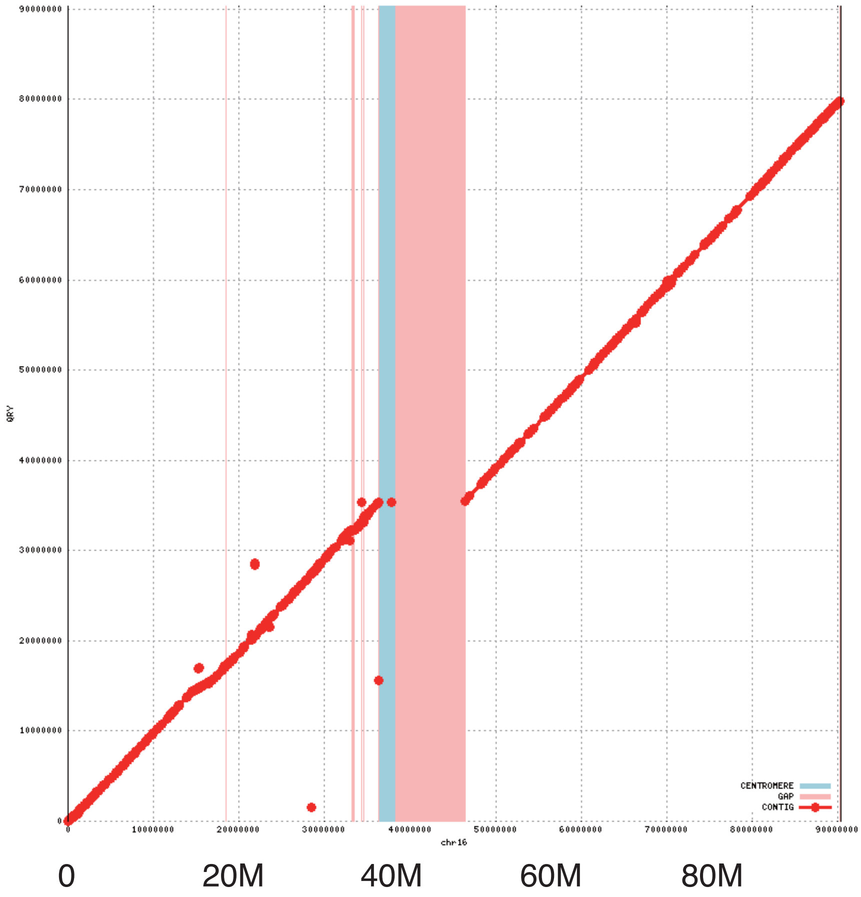

CENTROMERE
GAP
CONTIG
QRY
chr16
0
20M
40M
60M
80M

Chr 16

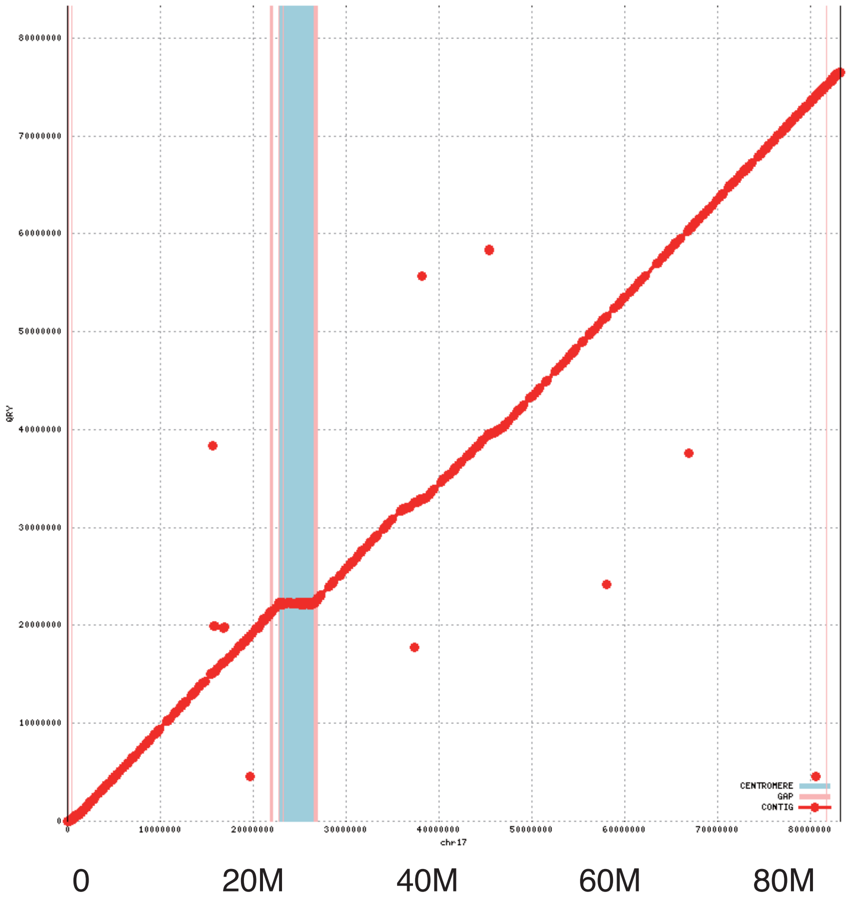
CENTROMERE
GAP
CONTIG
QRY
chr17
0
20M
40M
60M
80M

Chr 17

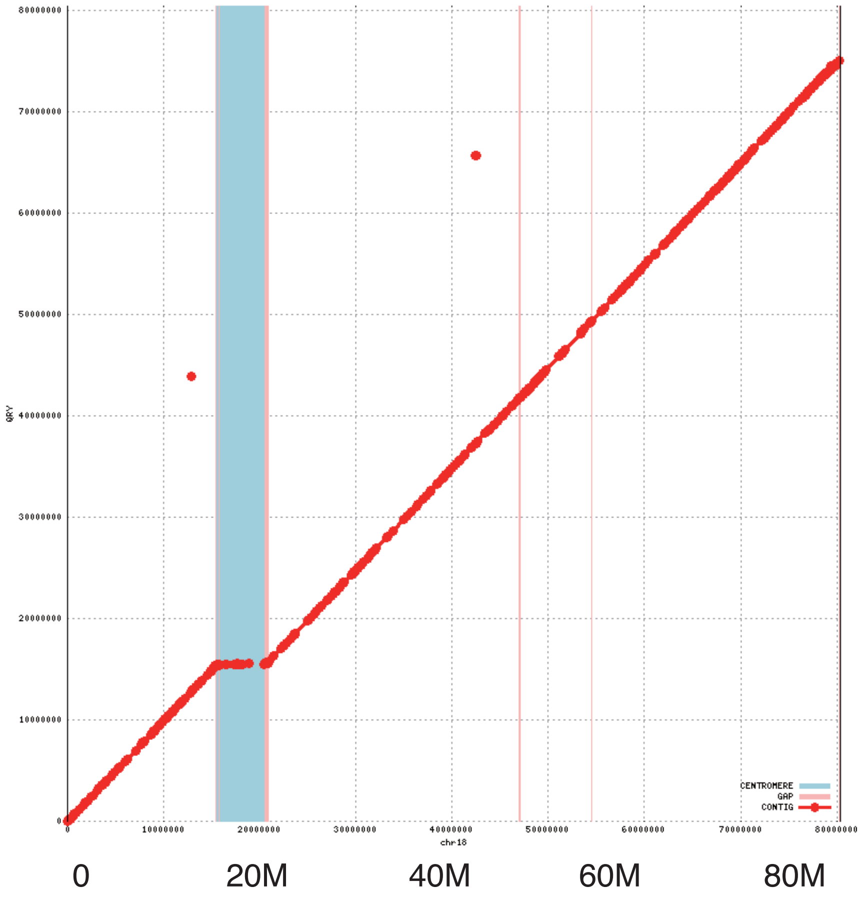
CENTROMERE
GAP
CONTIG
QRY
chr18
0
20M
40M
60M
80M

Chr 18

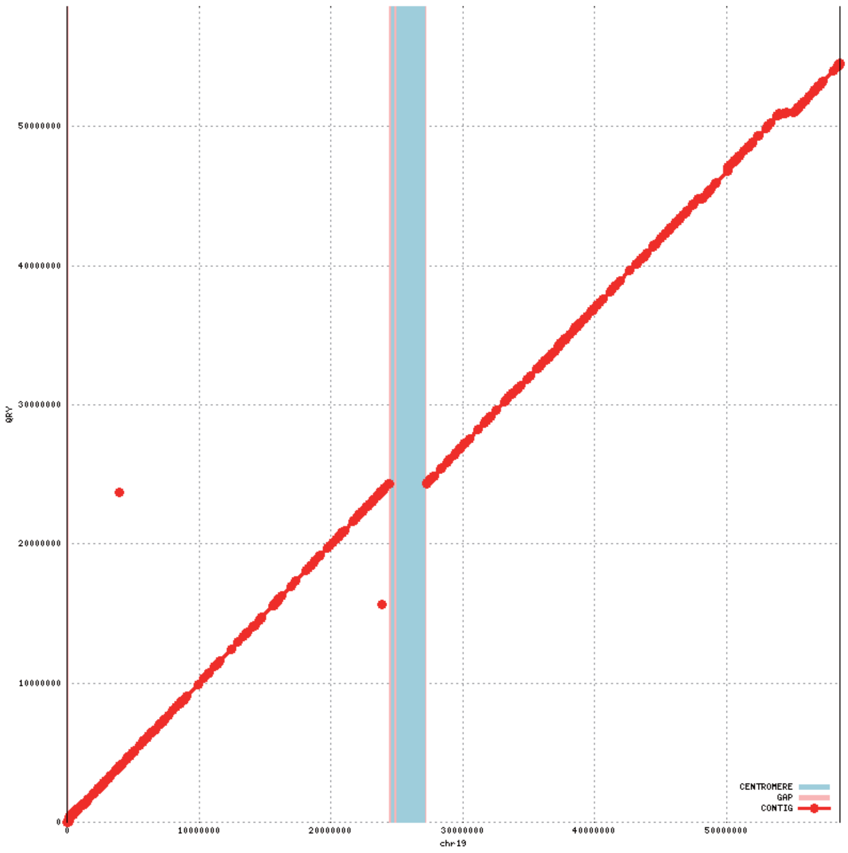

0 20M 40M 60M

Chr 19

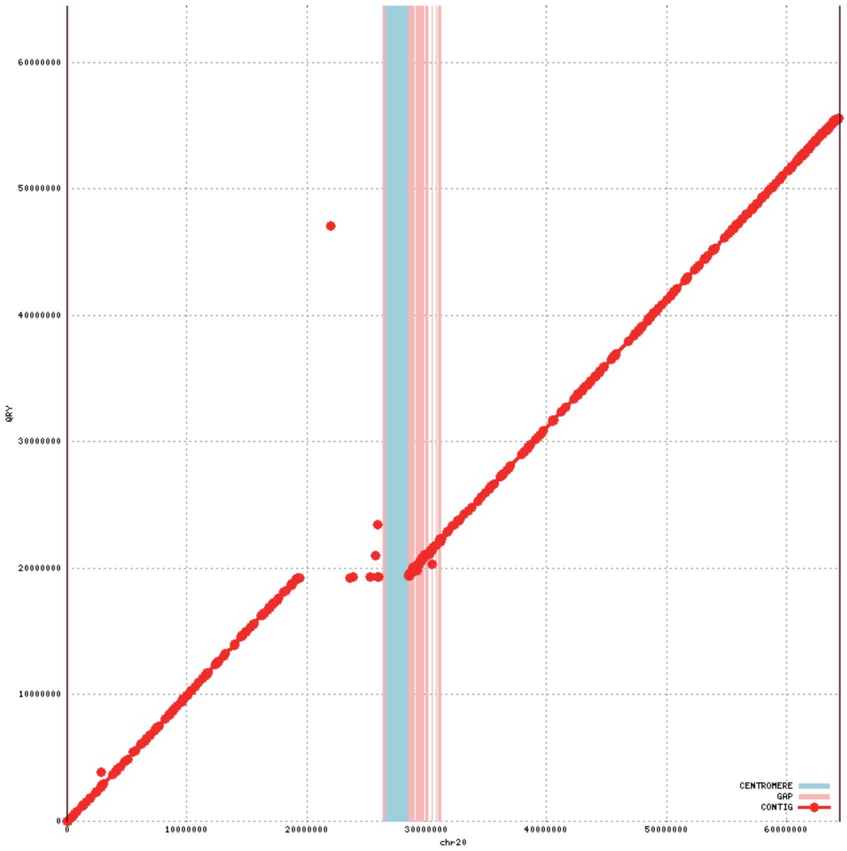

0 20M 40M 60M

Chr 20

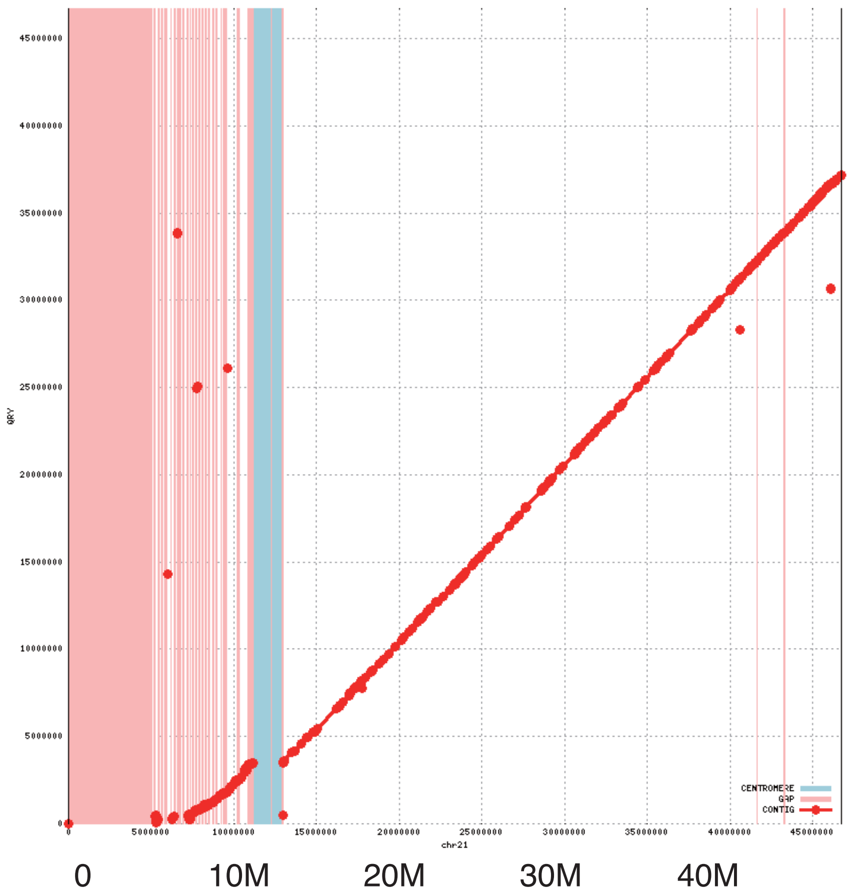

QRY
chr21
CENTROMERE
GAP
CONTIG
0
10M
20M
30M
40M

Chr 21

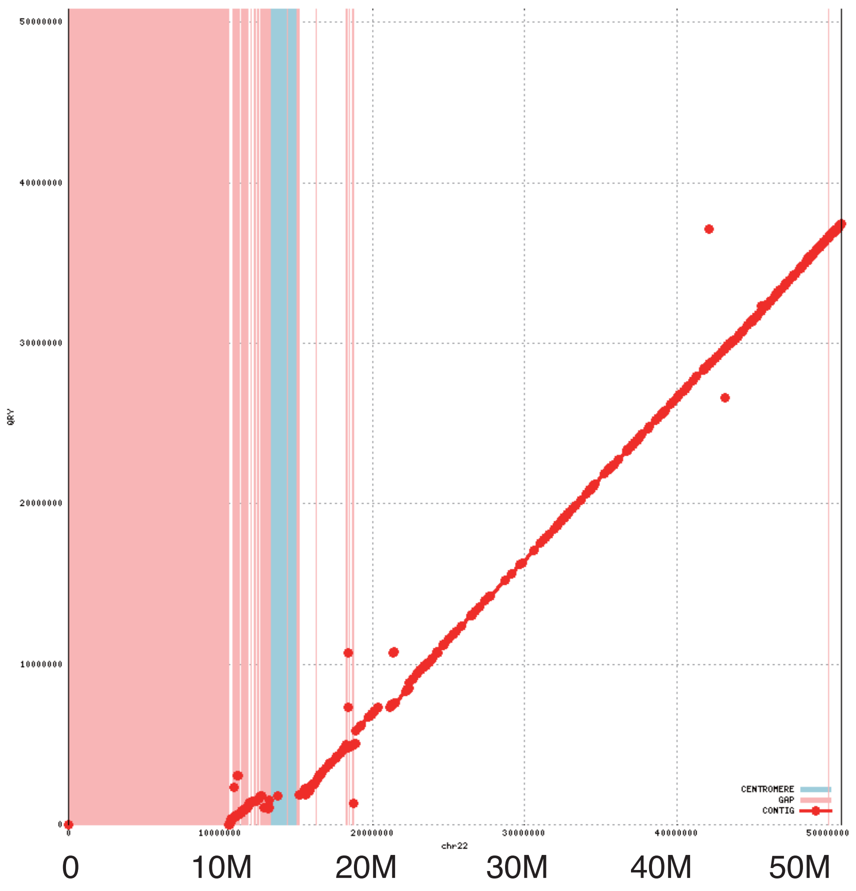

QRY
chr22
CENTROMERE
GAP
CONTIG
0
10M
20M
30M
40M
50M

Chr 22

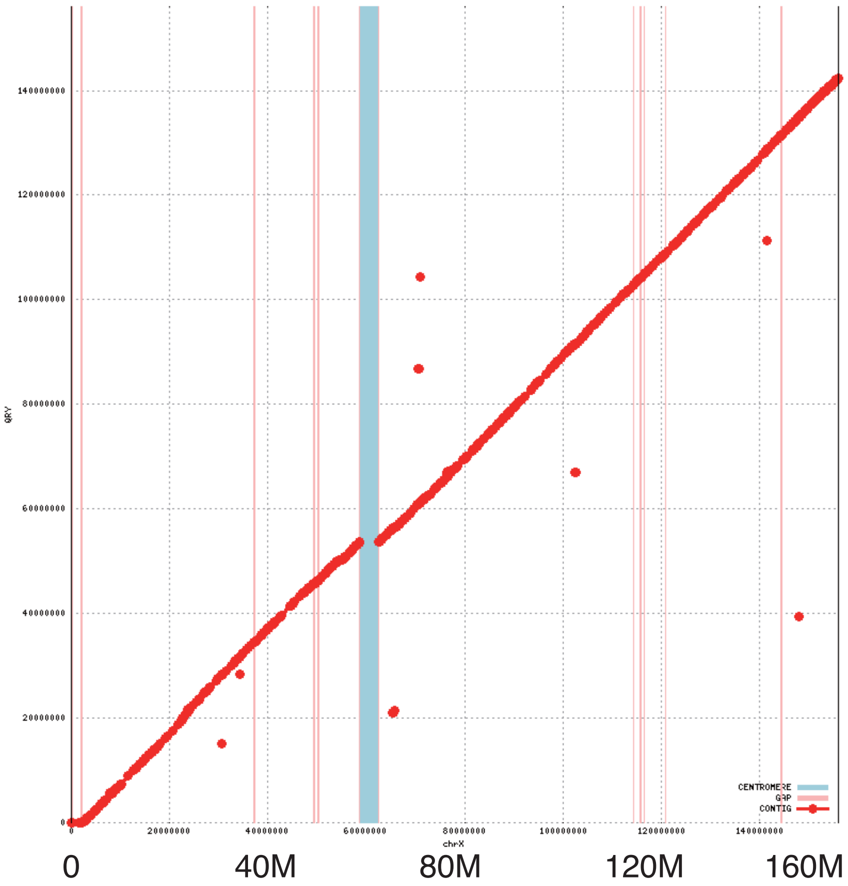

QRY
chrX
CENTROMERE
GAP
CONTIG
0
40M
80M
120M
160M

Chr X

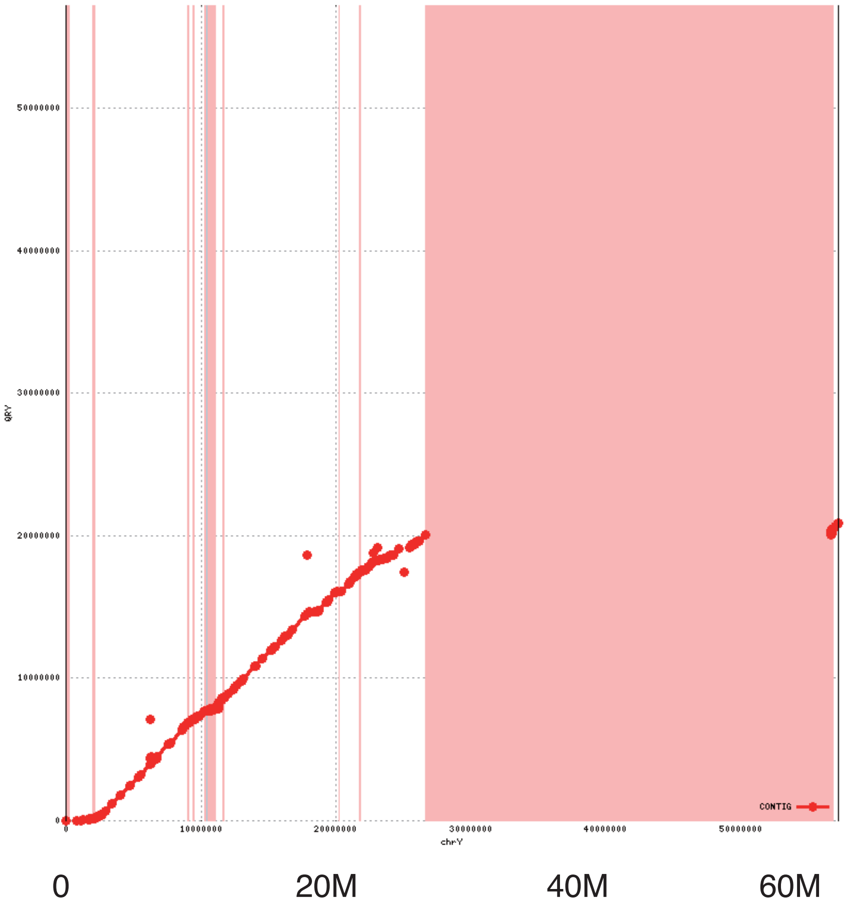

QRY
chrY
CONTIG
0
20M
40M
60M

Chr Y

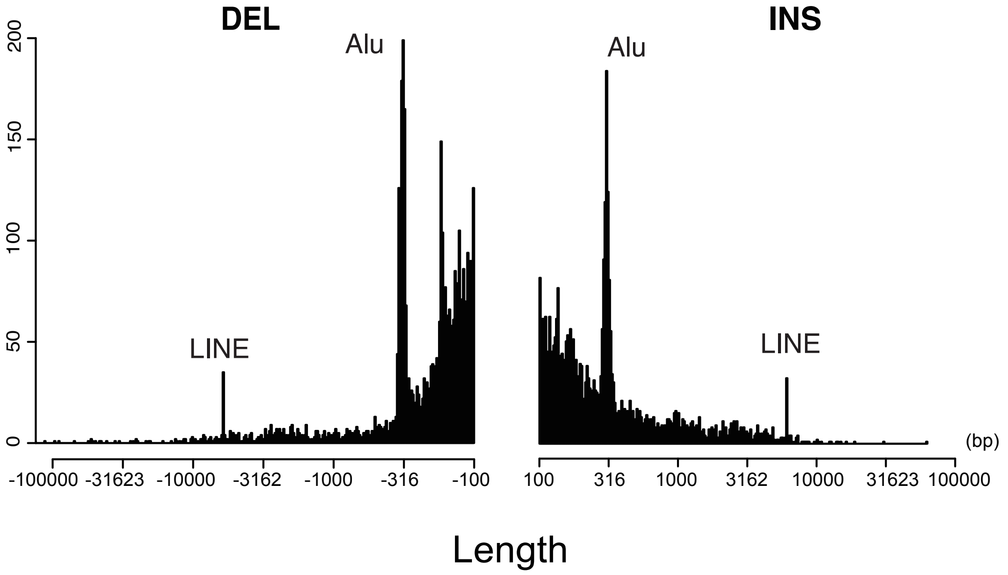

Supplementary Fig. 8. Size distribution of detected insertions and deletions.
Distribution of the lengths of 3,691 TMMINSs (right) and 4,040 deletions (left). Both showed two prominent peaks correspond to Alus and LINEs.

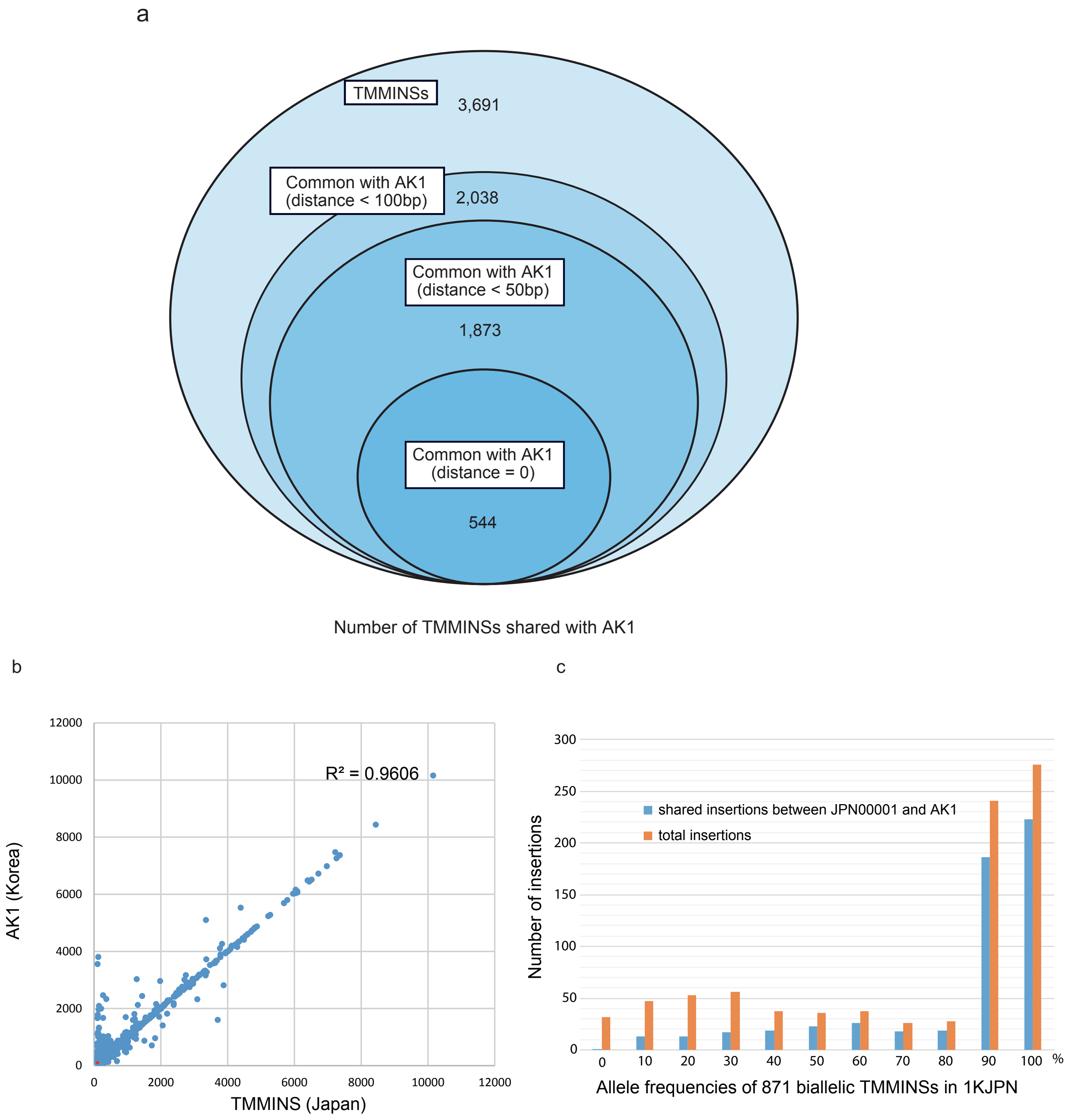

Supplementary Fig. 9. (a) Numbers of TMMINSs shared with AK1 within different distances, 0 bp, 50 bp and 100 bp each other. (b) The correlation of the insertion length to 1,873 shared insertions between TMMINSs (X-axis) and AK1 (Y-axis) (within 50bp from the insertion point). (c) The correlation of allele frequencies of 871 biallelic TMMINSs in 1KJPN (X-axis) and the number of shared insertions between JPN00001 and AK1 (within 50bp from the insertion point) (Y-axis).

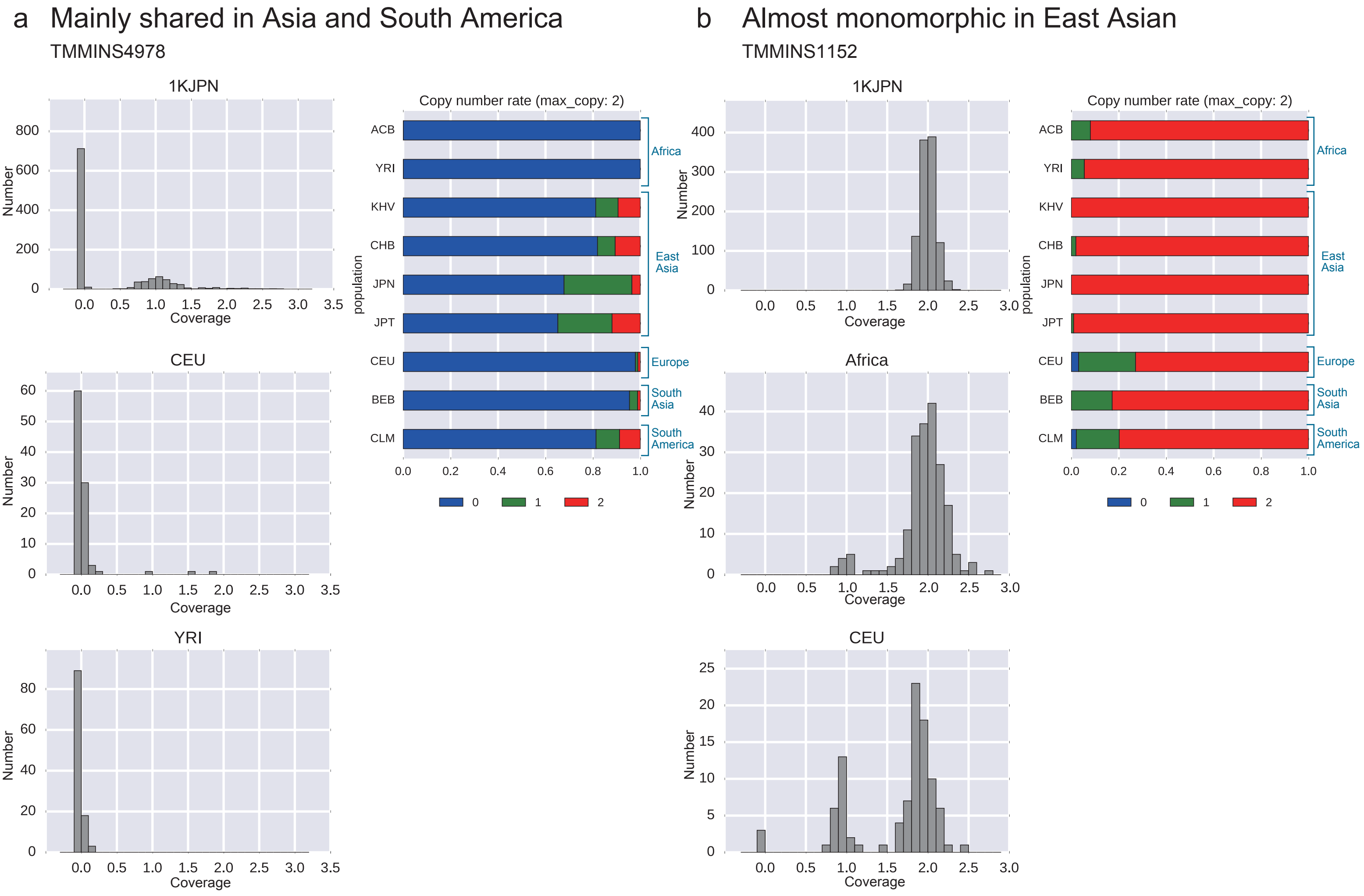

Supplementary Fig. 10. Other patterns of read coverage distributions of TMMINSs in 1KJPN and international populations. (a) A less common sequence, mainly shared in East Asian and CLM populations, compared with (a) in Fig. 3. (b) An almost monomorphic sequence in the East Asian population, but not in other populations.

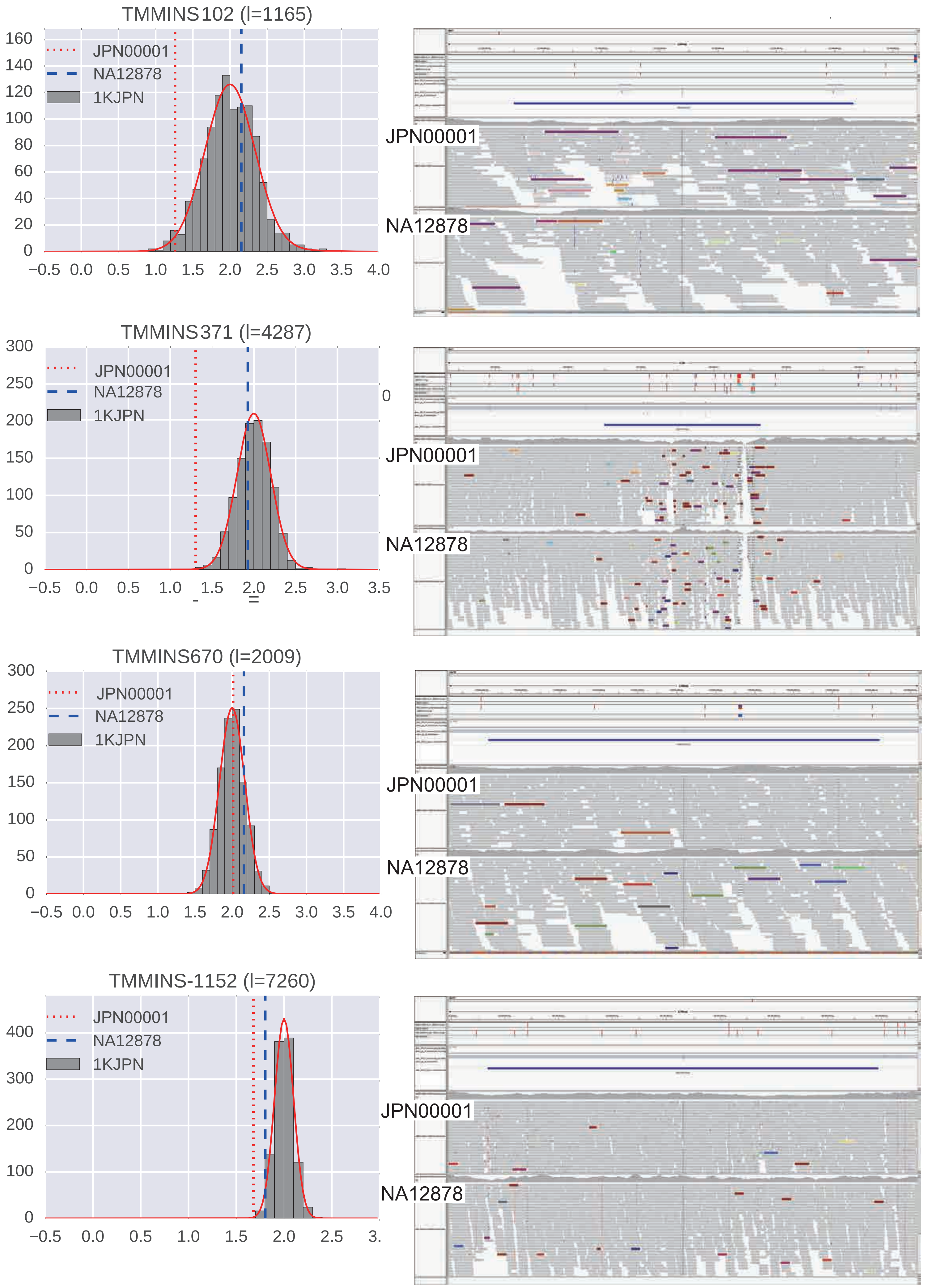

Supplementary Fig. 11. Genotype frequencies of PCR validated 10 TMMINSs in 1KJPN and an alignment snapshot in JPN00001 and NA12878. Left panel: genotype frequencies of 1KJPN (gray), JPN00001 (red dot) and NA12878 (blue dot). Right panel: iGV snapshot of JPN00001 (top) and NA12878 (bottom) to each PCR validated insertion.

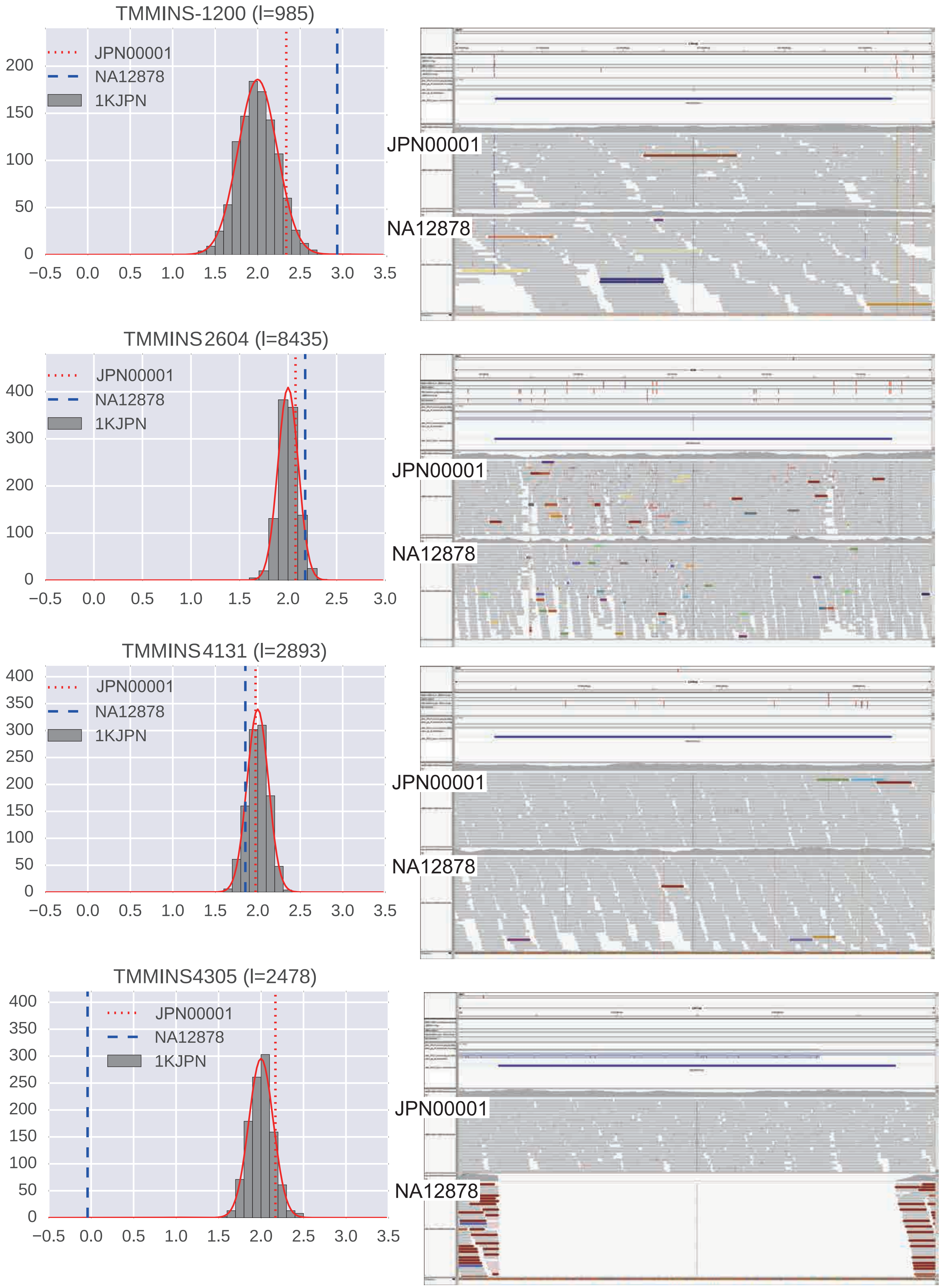
TMMINS-1200 (l=985)
JPN00001
NA12878
1KJPN
200
150
100
50
0
−0.5 0.0 0.5 1.0 1.5 2.0 2.5 3.0 3.5
JPN00001
NA12878
TMMINS2604 (l=8435)
JPN00001
NA12878
1KJPN
400
300
200
100
0
−0.5 0.0 0.5 1.0 1.5 2.0 2.5 3.0
JPN00001
NA12878
TMMINS4131 (l=2893)
JPN00001
NA12878
1KJPN
400
350
300
250
200
150
100
50
0
−0.5 0.0 0.5 1.0 1.5 2.0 2.5 3.0 3.5
JPN00001
NA12878
TMMINS4305 (l=2478)
JPN00001
NA12878
1KJPN
400
350
300
250
200
150
100
50
0
−0.5 0.0 0.5 1.0 1.5 2.0 2.5 3.0 3.5
JPN00001
NA12878

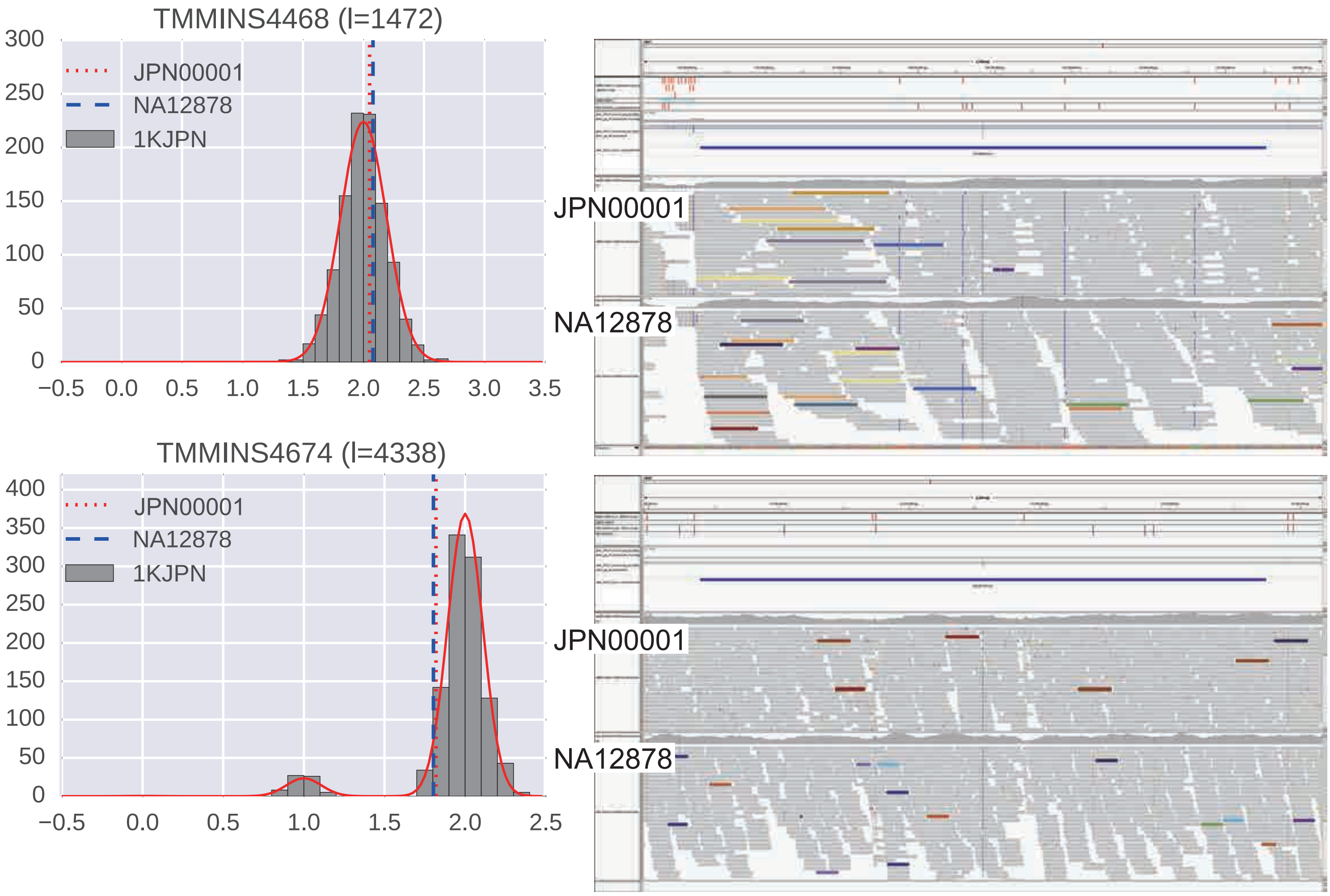

TMMINS4468 (l=1472)
JPN00001
NA12878
1KJPN
300
250
200
150
100
50
0
−0.5
0.0
0.5
1.0
1.5
2.0
2.5
3.0
3.5
JPN00001
NA12878
TMMINS4674 (l=4338)
JPN00001
NA12878
1KJPN
400
350
300
250
200
150
100
50
0
−0.5
0.0
0.5
1.0
1.5
2.0
2.5
JPN00001
NA12878

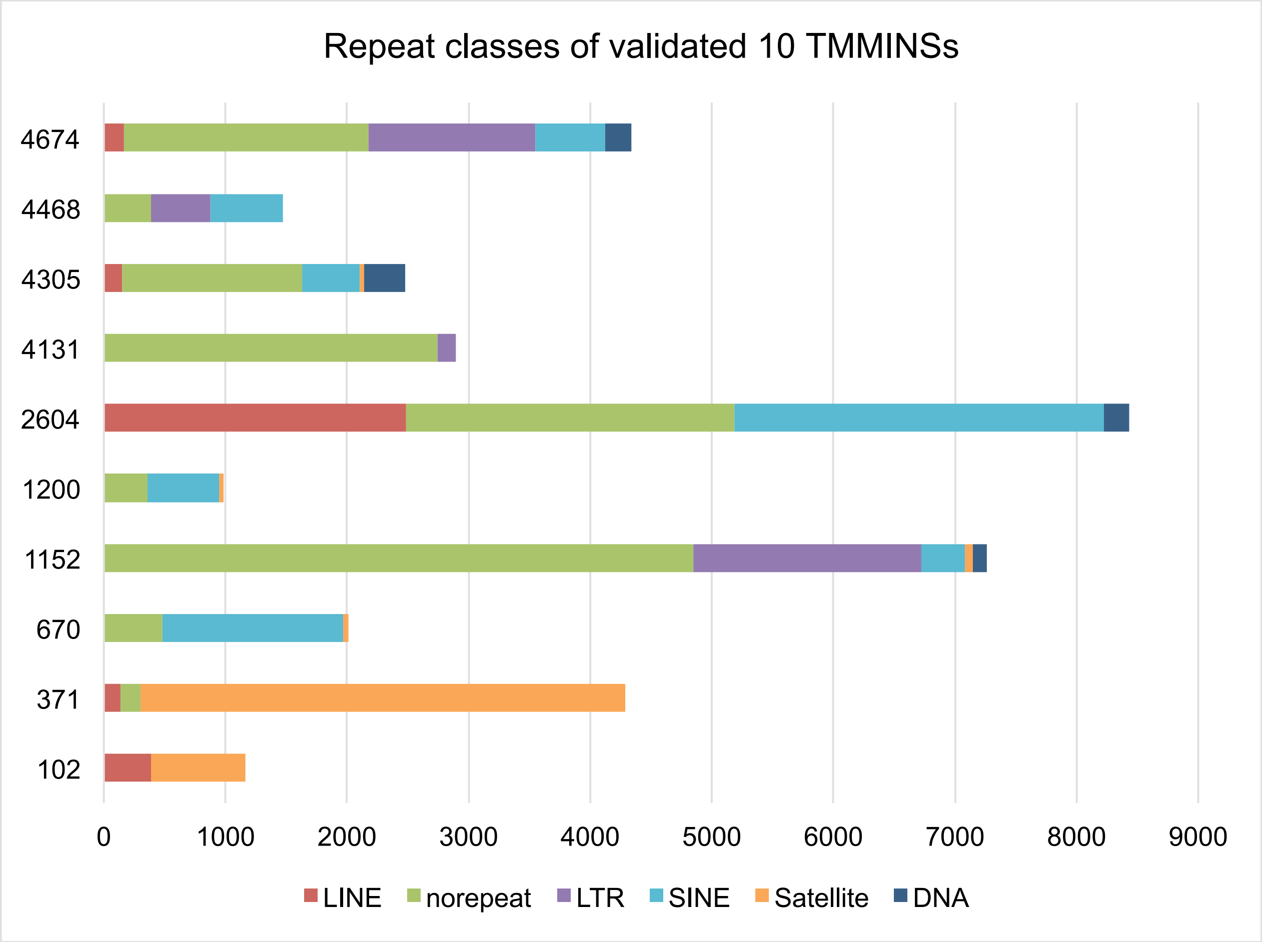

Supplementary Fig. 12. Repeat classes of PCR validated 10 TMMINSs. For each PCR validated insertion, the length for repeat classes, i.e. LINE, nonrepeat, LTR, SINE, Satellite and DNA, and their ratio to the total insertion length are shown.

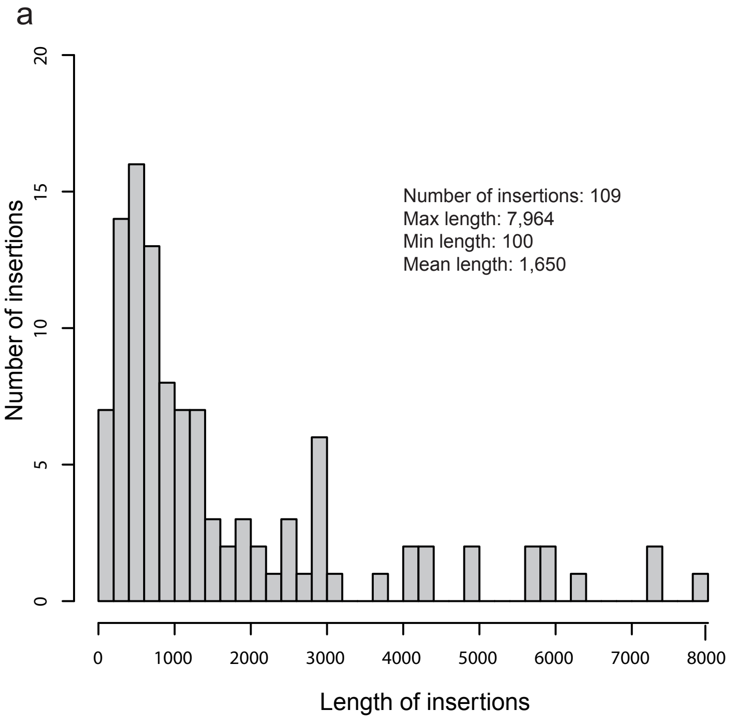

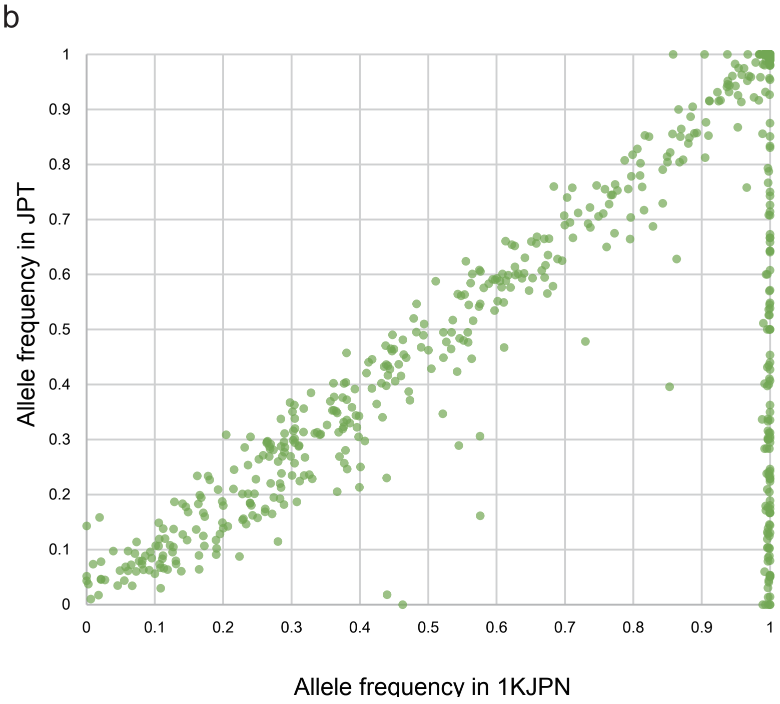

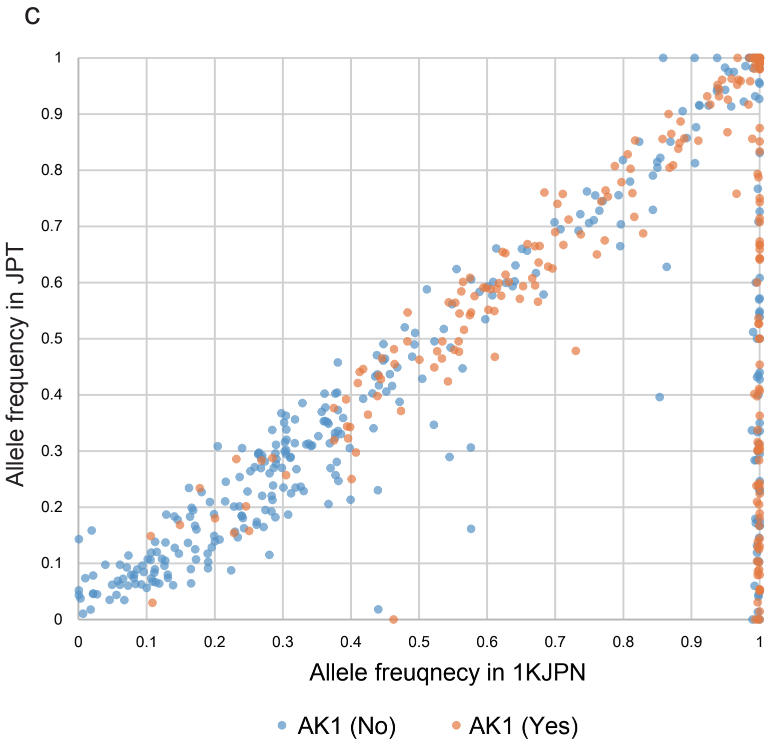

Supplementary Fig. 13. Length distribution of the shared insertions in 1KJPN and i1000g.
(a) The length distribution (X-axis) and counts (Y-axis) of shared insertions between 1KJPN and all i1000g population. (b) Scatter plots showing correlation between allele frequency in 1KJPN (X-axis) and that in JPT (Y-axis). (c) Insertions discovered in AK1 are colored in blue and insertions do not exist in AK1 are colored in orange.

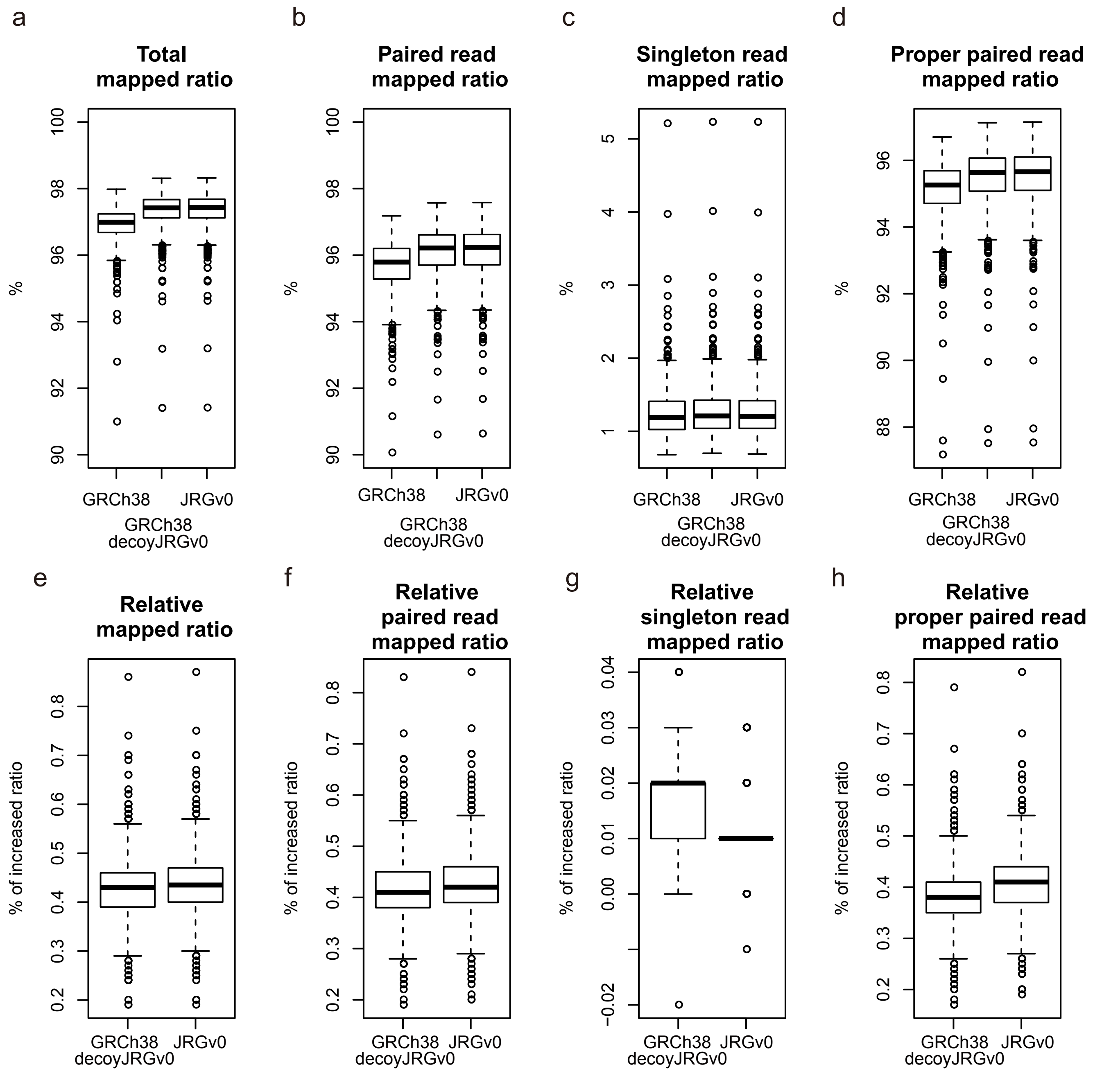

Supplementary Fig. 14 Alignment performance of decoyJRGv0 and JRGv0 using bowtie2 aligner.
(a-d) Plot the alignment ratios of short reads to the three references, GRCh38, GRCh38 + decoyJRGv0 and JRGv0 using bowtie2, respectively. (a) Total alignment ratios (b) Paired reads alignment ratios. (c) Singleton reads alignment ratios. (d) Proper read alignment ratios. (e-f) The relative alignment performance to GRCh38. (e) Total alignment ratios. (f) Paired reads alignment ratios. (g) Singleton reads alignment raitos. (h) Proper read alignment relative ratios.

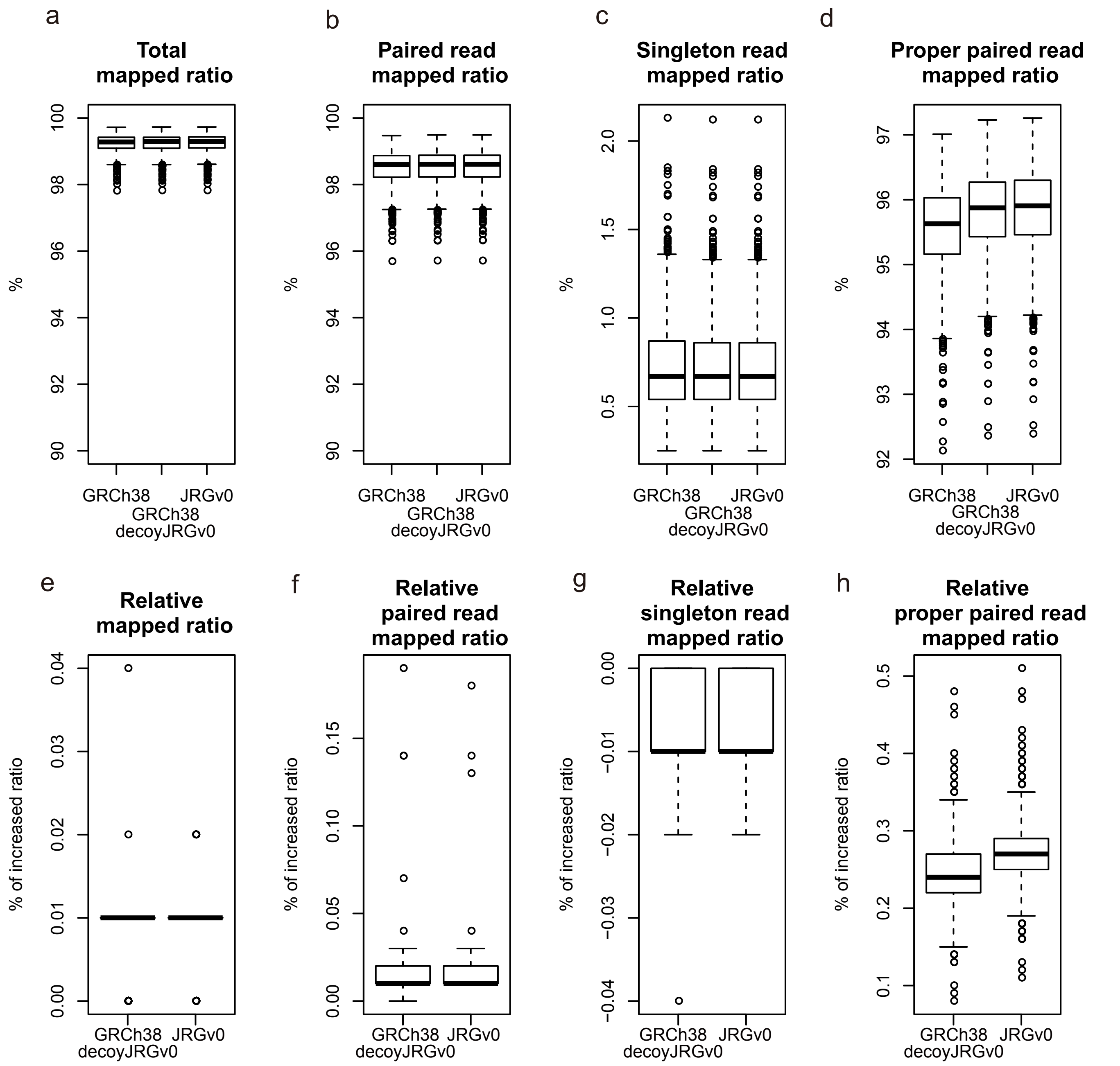

Supplementary Fig. 15 Alignment performance of decoyJRGv0 and JRGv0 using BWA aligner with “mem” option. (a-d) Plot the alignment ratios of short reads to the three references, GRCh38, GRCh38 + decoyJRGv0 and JRGv0 using the aligner, respectively. (a) Total alignment ratios. (b) Paired reads alignment ratios. (c) Singleton reads alignment ratios. (d) Proper read alignment ratios. (e-f) The relative alignment performance to GRCh38. (e) Total alignment ratios. (f) Paired reads alignment ratios. (g) Singleton reads alignment raitos. (h) Proper read alignment relative ratios.

Improved alignment of the reads around *ADRA1B* gene region by decoyJRGv0

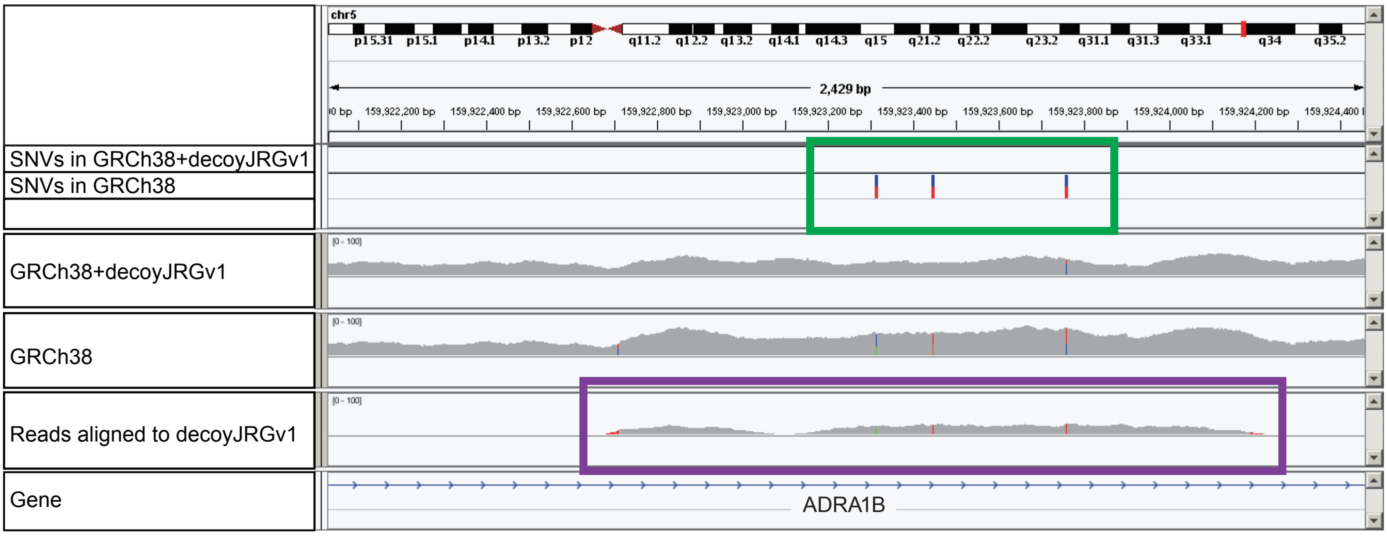

Improved alignment of the reads around *CD96* gene region by decoyJRGv0

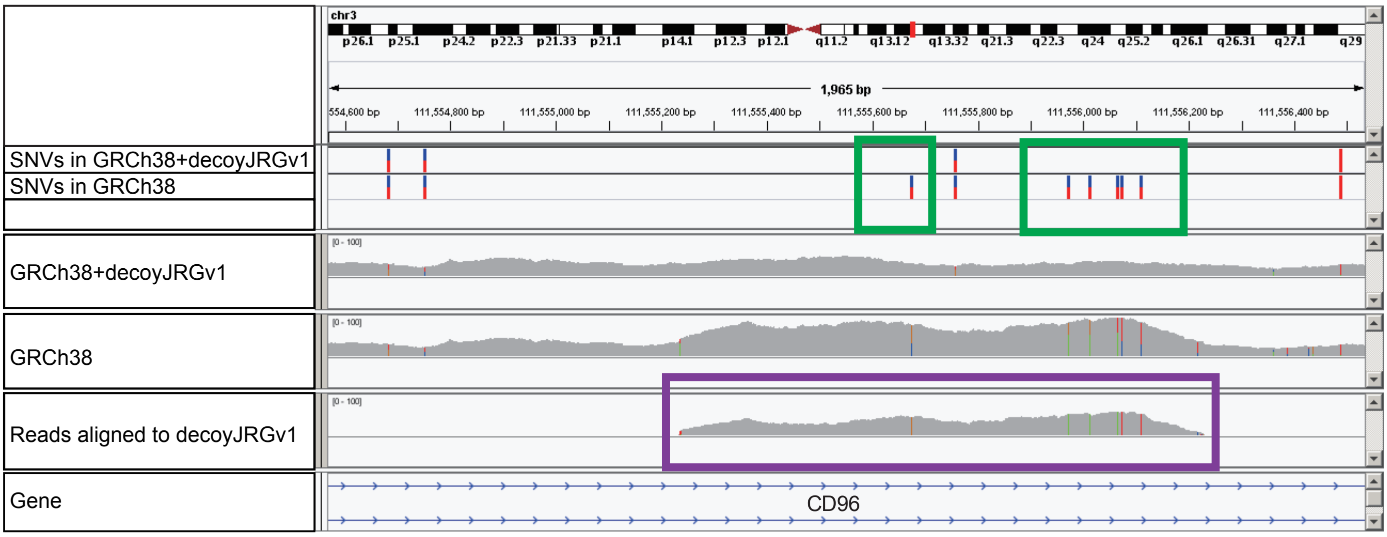

Supplementary Fig. 15. The alignment performance improvement using decoyJRGv1 in ADRA1B and CD96 regions. The result shows the comparison of the mapping results using GRCh38 and GRCh38 + decoyJRGv1 in two gene regions. A considerable number of misaligned mapped reads were aligned to decoyJRGv1 (reads in purple rectangles), i.e. avoid the misalignments to the sequence in GRCh38 chromosomes. Consequently the number of false positive SNVs was reduced (SNVs in green rectangles). (a) The alignment results to ADRA1B region, (b) The alignment results to CD96 region.

Correct alignments of the misaligned reads in *ADRA1B* to JRGv0

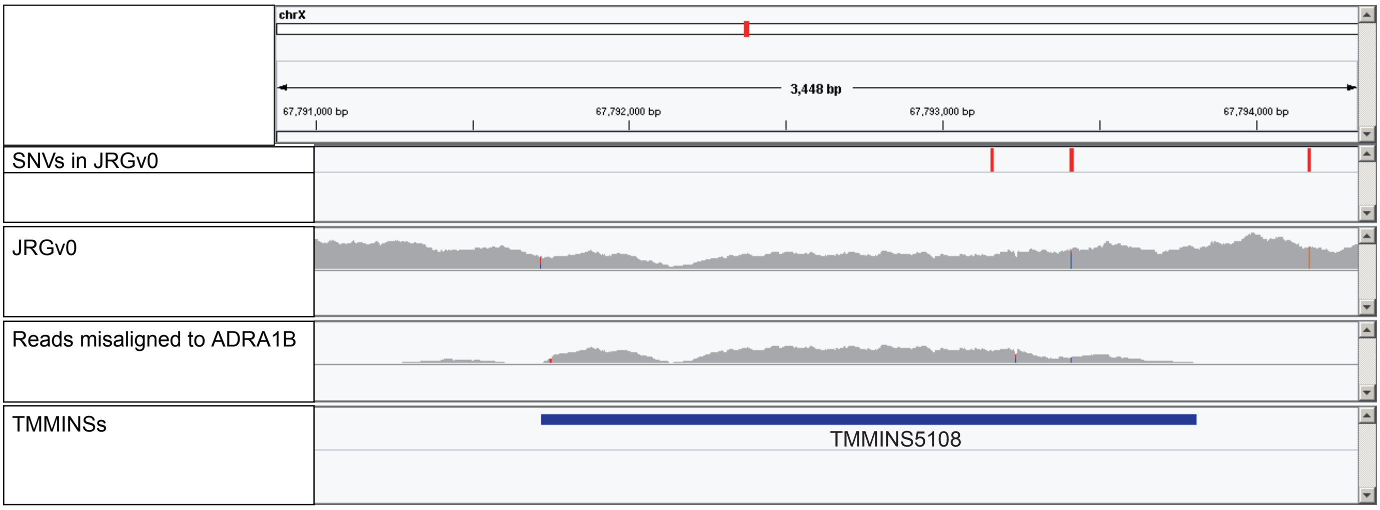

Correct alignments of the misaligned reads in *ALG1L2* to JRGv1

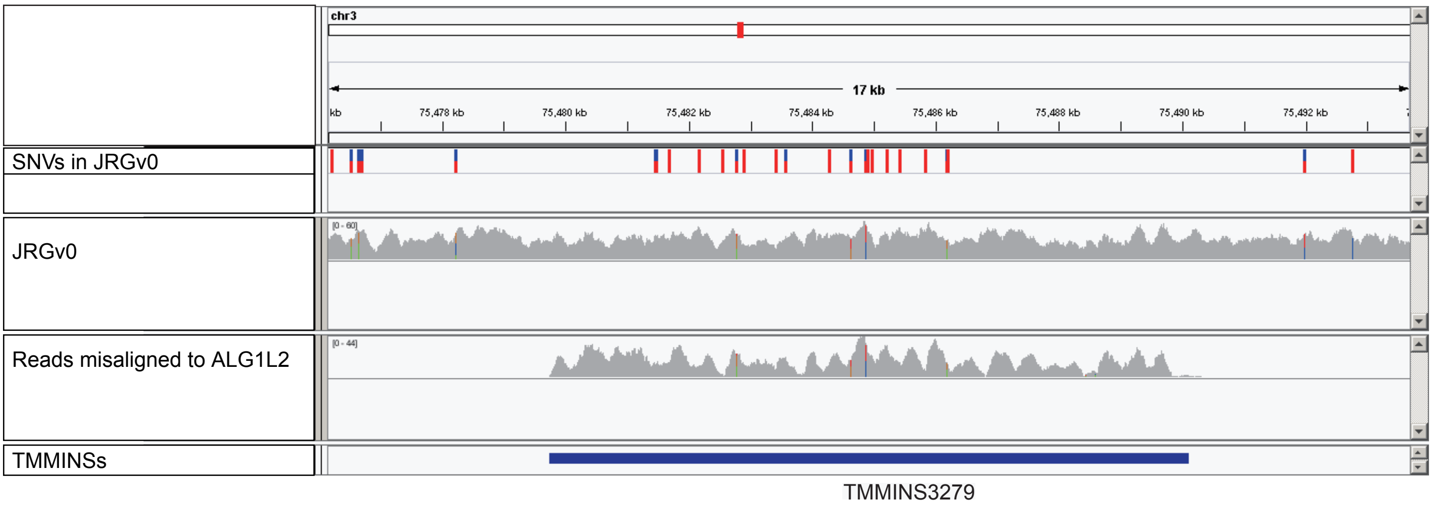

Correct alignments of the misaligned reads in *CD96* to JRGv1

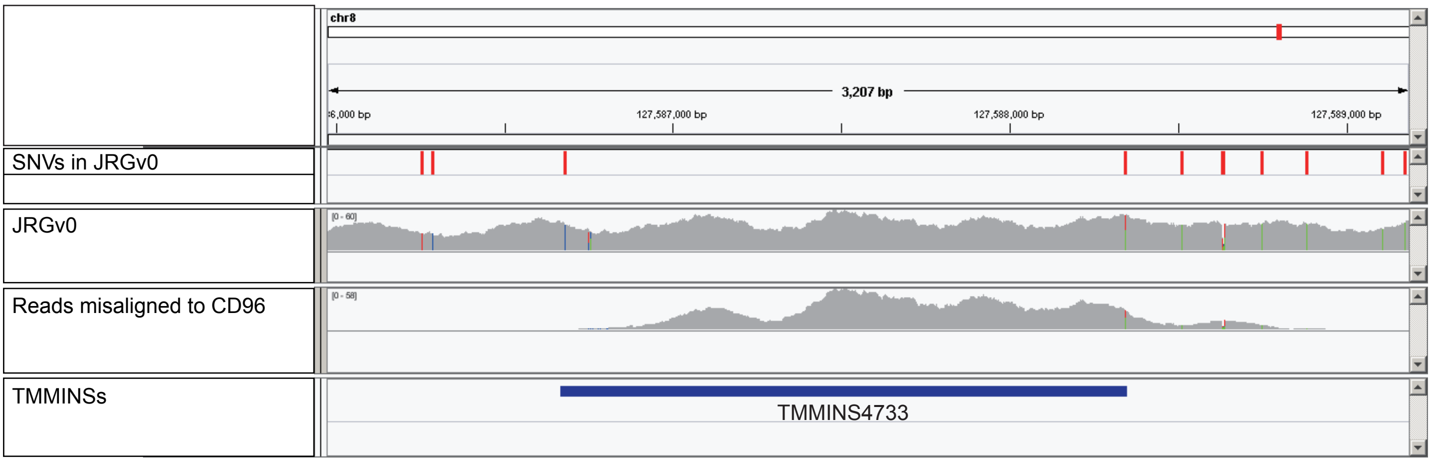

Supplementary Fig. 16. Correct alignments using JRGv0 for the misaligned reads in ADRA1B, ALG1L2 and CD96 regions. Using JRGv0 as a reference, misaligned reads when the reference was GRCh38 mapped to the TMMINS regions correctly. (a) ADRA1B region, (b) ALG1L2 region and (c) CD96 region.

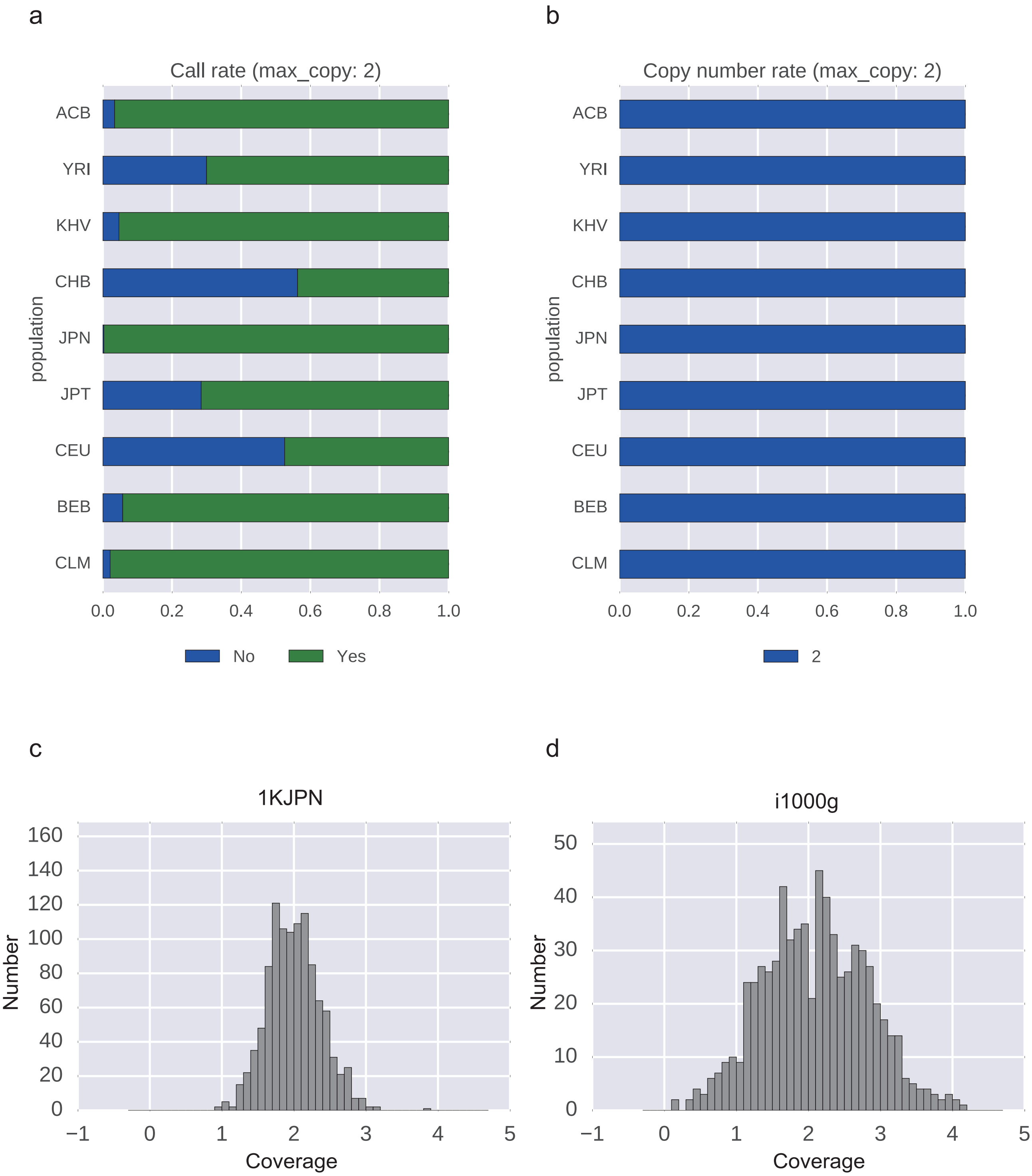

Supplementary Fig. 18. Read coverage distributions of TMMINS2292 in 1KJPN and other populations. TMMINS2292, which is located in the last exon of ZNF676, was shared between 1KJPN and i1000g populations. (a) Call rate of TMMINS2292 in each population. Green: ratio of the individuals called as "existing" or "not existing", blue: existence was not determined. (b) Copy number of TMMINS2292 in individuals for whom the existence was called in each population. All individuals had two copies. (c) Normalized coverage distribution of TMMINS2292 in 1KJPN population. It had a single peak around coverage = 2. (d) Normalized coverage distribution of TMMINS2292 in the i1000g population. It also had a single peak around coverage = 2.

```
          Chimp_ZNF676   MWSVLCLSLQPEGALTFRDVAIEFSLEEWQCLDSTQQNLYRNVMLENYRNLVFLGIAAFK
Human_ZNF676 (GRCh38)    -------------------------------------------MLENYRNLVFLGIAAFK
 Human_ZNF676 (JRGv1)    -------------------------------------------MLENYRNLVFLGIAAFK
                                                                    *****************

          Chimp_ZNF676   PDLIIFLEQGKEPWNMKRHDMVEEPPVICSHFSQEFWPEQGIEDSFQKMILRRYDKCGHE
Human_ZNF676 (GRCh38)    PDLIIFLEQGKEPWNMKRHEMVEEPPVICSHFSQEFWPEQGIEDSFQKMILRRYDKCGHE
 Human_ZNF676 (JRGv1)    PDLIIFLEQGKEPWNMKRHEMVEEPPVICSHFSQEFWPEQGIEDSFQKMILRRYDKCGHE
                         *******************:****************************************

          Chimp_ZNF676   NLHLKISCTNVDECNVHKEGYNKLNQSLTTTQSKVFQCGKYANVFHKCSNSNRHKIRHTG
Human_ZNF676 (GRCh38)    NLHLKISCTNVDECNVHKEGYNKLNQSLTTTQSKVFQCGKYANVFHKCSNSNRHKIRHTG
 Human_ZNF676 (JRGv1)    NLHLKISCTNVDECNVHKEGYNKLNQSLTTTQSKVFQCGKYANVFHKCSNSNRHKIRHTG
                         ************************************************************

          Chimp_ZNF676   EKGLKCKEYVRSFCMLSHLSQHERIYTRENSYKCEENGKAFNLSSTLTYYKSIHTGEKPY
Human_ZNF676 (GRCh38)    EKGLKCKEYVRSFCMLSHLSQHERIYTRENSYKCEENGKAFNWSSTLTYYKSIHTGEKPY
 Human_ZNF676 (JRGv1)    EKGLKCKEYVRSFCMLSHLSQHERIYTRENSYKCEENGKAFNWSSTLTYYKSIHTGEKPY
                         ****************************************** *****************

          Chimp_ZNF676   KCEECGKAFSKFSILTKHKVIHTGEKPYKCEECGKAFNRSSILTKHKIIHTGEKPYKCEE
Human_ZNF676 (GRCh38)    KCEECGKAFSKFSILTKHKVIHT-------------------------------------
 Human_ZNF676 (JRGv1)    KCEECGKAFSKFSILTKHKVIHTGEKPYKCEECGKAFNRSSILTKHKIIHTGEKPYKCEE
                         ***********************

          Chimp_ZNF676   CGKAFNSSSNLMEHKRVHTGEKPYKCEECGKAFSWSSSLTEHKRIHAGEKPYKCEECGKA
Human_ZNF676 (GRCh38)    -----------------------------------------------GEKPYKCEECGKA
 Human_ZNF676 (JRGv1)    CGKAFNSSSNLMEHKRVHTGEKPYKCEECGKAFSWSSSLTEHKRIHAGEKPYKCEECGKA
                                                                        *************

          Chimp_ZNF676   FNRSSILTKHKIIHTGEKPYKCEECGKGFSSVSTLNTHKAIHAEEKPYKCEECGKASNSS
Human_ZNF676 (GRCh38)    FNRSSILTKHKIIHTGEKPYKCEECGKGFSSVSTLNTHKAIHAEEKPYKCEECGKASNSS
 Human_ZNF676 (JRGv1)    FNRSSILTKHKIIHTGEKPYKCEECGKGFSSVSTLNTHKAIHAEEKPYKCEECGKASNSS
                         ************************************************************

          Chimp_ZNF676   SKLMEHKRIHTGEKPYKCEECGKAFSWSSSLTEHKRIHAGEKPYKCEECGKAFNRSSILT
Human_ZNF676 (GRCh38)    SKLMEHKRIHTGEKPYKCEECGKAFSWSSSLTEHKRIHAGEKPYKCEECGKAFNRSSILT
 Human_ZNF676 (JRGv1)    SKLMEHKRIHTGEKPYKCEECGKAFSWSSSLTEHKRIHAGEKPYKCEECGKAFNRSSILT
                         ************************************************************

          Chimp_ZNF676   KHKIIHTGEKPYKCEGCGKAFSKVSTLNTHKAIHAEEKPYKCEECGKASNSSSKLMEHKR
Human_ZNF676 (GRCh38)    KHKIIHTGEKPYKCEGCGKAFSKVSTLNTHKAIHAEEKPYKCEECGKASNSSSKLMEHKR
 Human_ZNF676 (JRGv1)    KHKIIHTGEKPYKCEGCGKAFSKVSTLNTHKAIHAEEKPYKCEECGKASNSSSKLMEHKR
                         ************************************************************

          Chimp_ZNF676   IHTGEKPYKCEECGKAFSWSSSLTEHKRIHAGEKPYKCEECGKAFTWSSSFTKHKRIHAA
Human_ZNF676 (GRCh38)    IHTGEKPYKCEECGKAFSWSSSLTEHKRIHAGEKPYKCEECGKAFTWSSSFTKHKRIHAA
 Human_ZNF676 (JRGv1)    IHTGEKPYKCEECGKAFSWSSSLTEHKRIHAGEKPYKCEECGKAFTWSSSFTKHKRIHAA
                         ************************************************************

          Chimp_ZNF676   EKPYKCEECGKGFSTFSILTKHKIIHTGEKRYKCEECGKAFSWSSILTEHKIIHTGEKPY
Human_ZNF676 (GRCh38)    EKPYKCEECGKGFSTFSILTKHKIIHTGEKRYKCEECGKAFSWSSILTEHKIIHTGEKPY
 Human_ZNF676 (JRGv1)    EKPYKCEECGKGFSTFSILTKHKIIHTGEKRYKCEECGKAFSWSSILTEHKIIHTGEKPY
                         ************************************************************

          Chimp_ZNF676   KCEECGKAFSRSSSLTRHKRIHTGEKPYKCKECGKAFKSSSTISYHKKIHTGENPXKCEE
Human_ZNF676 (GRCh38)    KCEECGKAFSRSSSLTRHKRIHTGEKPYKCEECGKAFKSSSTVSYHKKIHTGENP-----
 Human_ZNF676 (JRGv1)    KCEECGKAFSRSSSLTRHKRIHTGEKPYKCEECGKAFKSSSTVSYHKKIHTGENP-----
                         ******************************:***********:************

          Chimp_ZNF676   CGKAFSXFSVFSKHKKIHTGEKIYKGEECGKAFKQSSTFITHKKIHAGEKHYKREECDKG
Human_ZNF676 (GRCh38)    ------------------------------------------------------------
 Human_ZNF676 (JRGv1)    ------------------------------------------------------------

          Chimp_ZNF676   FNRSSNLVEHKRIHTGEKPYKCEECDKDFNXSSHLTTHKRIHTGEKPYKCEKRGKAFHXF
Human_ZNF676 (GRCh38)    ------------------------------------------------------------
 Human_ZNF676 (JRGv1)    ------------------------------------------------------------

          Chimp_ZNF676   STLTEHKGIYQEGNPTNVKNVASLLAVPQSLLNIHVIHTGGNSYNCVECGKAFNQSLRLT
Human_ZNF676 (GRCh38)    ------------------------------------------------------------
 Human_ZNF676 (JRGv1)    ------------------------------------------------------------

          Chimp_ZNF676   TYKTTHTGEKPCMCEECGKASNRSSILKKHKLIHT
Human_ZNF676 (GRCh38)    -----------------------------------
 Human_ZNF676 (JRGv1)    -----------------------------------
```

Supplementary Fig. 19. Comparison of the ZNF676 gene among chimpanzee, GRCh38 and JRGv1. Multiple alignments of all amino acids in the ZNF676 protein of the chimpanzee reference assembly, GRCh38 and JRGv1. Asterisks indicate the conserved amino acids.

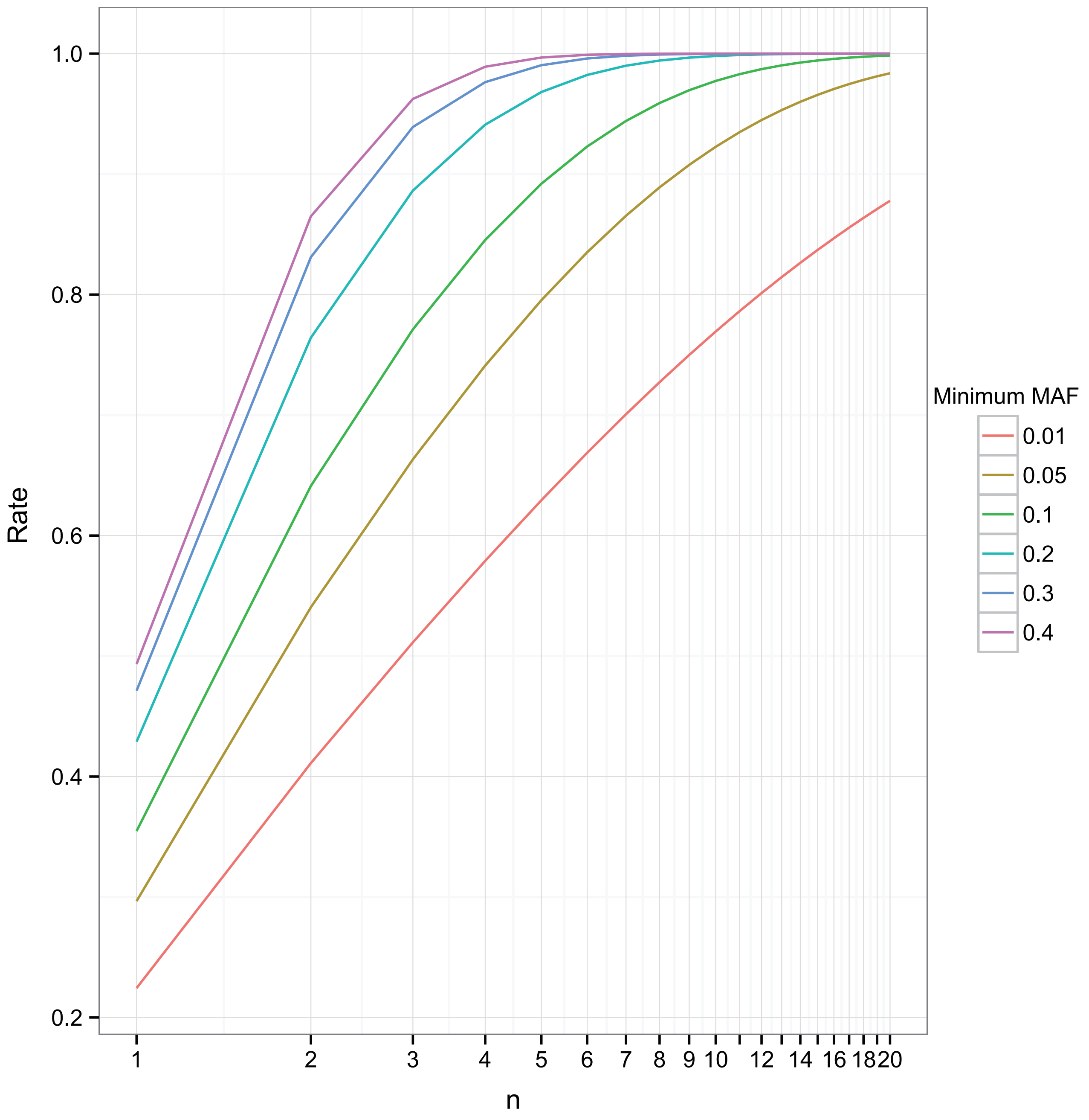

Supplementary Fig. 20. Estimated discovery rates of novel sequences. The rates of novel sequence discovery (Y-axis) are plotted against the number of samples for the discovery study (X-axis). These rates are plotted using different colors according to the minimum minor allele frequency of undiscovered sequences among a population.
