# Supplementary material for: Construction of JRG (Japanese reference genome) with single-molecule real-time sequencing
Source: Hum Genome Var. 2019 Jun 7;6:27. doi: 10.1038/s41439-019-0057-7 (PMC6555796; doi:10.1038/s41439-019-0057-7)
Supplement: Supplementary file 3 — Supplementary Tables1-7 [file 41439_2019_57_MOESM3_ESM.pdf]

Supplementary Table 1. Analyzed individuals and populations in the international 1000 genomes.

Population	Mainland	Mailand detail	Sex	Total
ACB	AFR	Africa	male	43
ACB	AFR	Africa	female	45
BEB	SAN	South Asia	male	43
BEB	SAN	South Asia	female	44
CEU	EUR	Europe	male	47
CEU	EUR	Europe	female	49
CHB	ASN	East Asia	male	46
CHB	ASN	East Asia	female	57
CLM	AMR	America	male	44
CLM	AMR	America	female	51
JPT	ASN	East Asia	male	55
JPT	ASN	East Asia	female	47
KHV	ASN	East Asia	male	41
KHV	ASN	East Asia	female	45
YRI	AFR	Africa	male	53
YRI	AFR	Africa	female	57
Total				767

Supplementary Table 2. Primer list for PCR validation.

a. Primers for the 1st PCR

Chr	Identity	Direction	Sequence	TMMINS length	TMMINS positions in JRGv1		Primer-designed region (JRGv1 coordinate)		Primer-designed region (GRCh38 coordinate)		Predicted PCR product size in JRGv1	Predicted PCR product size in GRCh38	Gene
					start	end	start	end	start	end			
1	TMMINS102	Forward	*GCAGTCGAACATGTAGCTGACTCAGGTCAC* GTCTCTCATGGTACCTTATTCCCTTG	1,165	27,142,299	27,143,464	27,138,396	27,146,350	27,082,597	27,089,381	7,954	6,784	
		Reverse	*TGGATCACTTGTGCAAGCATCACATCGTAG* ATTTGAGATGTAGTCCCTATCACCCC										
1	TMMINS371	Forward	*GCAGTCGAACATGTAGCTGACTCAGGTCAC* CTAGCATATTCCAAAGTTCTCTCTGCCTCC	4,287	223,283,005	223,287,292	223,282,703	223,288,100	223,080,887	223,081,996	5,397	1,109	
		Reverse	*TGGATCACTTGTGCAAGCATCACATCGTAG* CAGGCTGGATATTCATTCTAAGGCC										
2	TMMINS2604	Forward	*GCAGTCGAACATGTAGCTGACTCAGGTCAC* CAGTTCCTGGTATCTGCTCCTATATAGTCC	8,435	172,106,229	172,114,664	172,102,865	172,115,261	171,972,705	171,976,663	12,396	3,958	SLC25A12, intron
		Reverse	*TGGATCACTTGTGCAAGCATCACATCGTAG* CATTCTTCCCACCAATAATGTCTCCAGG										
6	TMMINS4131	Forward	*GCAGTCGAACATGTAGCTGACTCAGGTCAC* GCTCCTTCCTCTATACAACTAAGGG	2,893	79,965,345	79,968,238	79,965,040	79,969,230	79,901,250	79,902,547	4,190	1,297	
		Reverse	*TGGATCACTTGTGCAAGCATCACATCGTAG* CTGGCTTGTAGTGTAATCTCTAGTGTGTCC										
7	TMMINS4305	Forward	*GCAGTCGAACATGTAGCTGACTCAGGTCAC* CTGCTCCTAGGTCTAAGAGTAAGTAAGG	2,478	8,336,260	8,338,738	8,331,695	8,339,386	8,328,117	8,333,328	7,691	5,211	AC007128.1, intron
		Reverse	*TGGATCACTTGTGCAAGCATCACATCGTAG* GGAAGTGCACACATAGTCAAAAGCC										
7	TMMINS4467	Forward	*GCAGTCGAACATGTAGCTGACTCAGGTCAC* CACCATCTCCTAAAACGGTTTCTGTCTAGG	560	107,834,272	107,834,832	107,832,147	107,841,239	107,725,333	107,732,236	9,092	6,903	
	TMMINS4468	Reverse	*TGGATCACTTGTGCAAGCATCACATCGTAG* TTTTCTTGGGACGGGGTAAACATCTG	1,472	107,837,633	107,839,105							
8	TMMINS4674	Forward	*GCAGTCGAACATGTAGCTGACTCAGGTCAC* CTTCCAGCTCATCCTAGACCTACATTTTGG	4,338	61,255,397	61,259,735	61,255,073	61,262,035	61,212,999	61,215,620	6,962	2,621	
		Reverse	*TGGATCACTTGTGCAAGCATCACATCGTAG* GGCTTCAAAGTTCTAGCTAGTAGGATCC										
10	TMMINS670	Forward	*GCAGTCGAACATGTAGCTGACTCAGGTCAC* CTTAAGACATGCTATGAATCCCTCCTGAGC	2,009	119,690,433	119,692,442	119,687,627	119,693,772	119,544,517	119,548,662	6,145	4,145	
		Reverse	*TGGATCACTTGTGCAAGCATCACATCGTAG* CTCAGCCAGTAGTTCATAAGTCCAGAGC										
12	TMMINS1152	Forward	*GCAGTCGAACATGTAGCTGACTCAGGTCAC* TCAGTCTGCACATGTATCTGGGTTAAGG	7,260	86,346,684	86,353,944	86,346,190	86,354,815	86,259,298	86,260,666	8,625	1,368	MGAT4C, intron
		Reverse	*TGGATCACTTGTGCAAGCATCACATCGTAG* CTCTGACTTACACTCTATGCTCTGATCC										
12	TMMINS1200	Forward	*GCAGTCGAACATGTAGCTGACTCAGGTCAC* CGTACGCACATAGATTTCAAGAGAGG	985	121,169,082	121,170,067	121,169,019	121,170,407	121,052,709	121,053,089	1,388	380	
		Reverse	*TGGATCACTTGTGCAAGCATCACATCGTAG* GATACCGTACAATGTTAGGGATAGCC										

Italic characters indicate universal sequences for the 1st and the 2nd PCR.

TMMINS4467 and TMMINS 4468 were included in one PCR region. Existence of both two were confirmed by PCR product length and sequencing.

Chr: Chromosome

b. Primers for the 2nd PCR

Index 1	Forward	GGTAGGCGCTCTGTGTGCAGC *GCAGTCGAACATGTAGCTGACTCAGGTCAC*
	Reverse	CCATCTCATATGTAGTACTCT *TGGATCACTTGTGCAAGCATCACATCGTAG*
Index 2	Forward	GGTAGTCATGAGTCGACACTA *GCAGTCGAACATGTAGCTGACTCAGGTCAC*
	Reverse	CCATCGCGATCTATGCACACG *TGGATCACTTGTGCAAGCATCACATCGTAG*

b. Index 1 was used for the sample JPN00001 and index 2 was for NA12878.

Supplementary Table 3. Statistics of the assembly.

Chr*	Sequence data coverage (x)	Number of contigs	Total contig length (bp)	Max contig length (bp)	N50 contig length (bp)	Average contig length (bp)	GRCh38 chromosome length excluding Ns (bp)	Total contig length /GRCh38 chromosome length excluding Ns	Number of detected insertions
All	-	8,963	3,005,226,201	8,980,851	2,063,069	335,292	2,937,639,113	1.023	3,691
1	103.4	768	239,804,808	7,334,856	2,203,576	312,246	230,481,012	1.040	309
2	110.0	695	252,230,444	8,327,381	2,403,608	362,922	240,548,228	1.049	243
3	111.3	482	206,085,831	8,140,595	2,214,460	427,564	198,100,135	1.040	216
4	116.6	609	201,224,338	6,752,894	1,935,266	330,418	189,752,667	1.060	195
5	111.2	489	186,408,127	6,487,799	2,336,772	381,203	181,265,378	1.028	168
6	108.6	465	173,537,994	8,033,860	1,965,487	373,200	170,078,522	1.020	209
7	108.4	571	165,367,764	5,244,112	1,522,997	289,611	158,970,131	1.040	218
8	110.1	398	148,756,701	8,980,851	2,523,972	373,761	144,768,136	1.028	152
9	94.5	589	127,928,285	7,402,535	2,334,573	217,196	121,790,550	1.050	189
10	116.7	390	141,109,772	8,934,635	3,002,521	361,820	133,262,962	1.059	173
11	107.6	355	137,756,170	8,719,303	2,797,989	388,046	134,533,742	1.024	208
12	107.6	340	137,595,943	4,944,683	1,959,042	404,694	133,137,816	1.033	178
13	97.6	245	101,166,176	6,299,428	2,221,377	412,923	97,983,125	1.032	172
14	92.9	220	93,095,444	4,991,752	2,792,678	423,161	90,568,149	1.028	97
15	86.0	309	84,400,768	5,869,895	1,496,418	273,142	84,641,325	0.997	87
16	98.2	448	87,845,890	3,660,737	1,336,756	196,085	81,805,943	1.074	96
17	95.8	239	80,001,206	5,478,828	1,716,060	334,733	82,920,204	0.965	130
18	107.3	176	78,496,616	7,574,015	2,653,706	446,004	80,089,605	0.980	93
19	94.3	137	56,448,220	4,921,697	1,718,140	412,031	58,440,758	0.966	118
20	92.8	215	58,970,503	5,182,149	1,914,098	274,281	63,944,257	0.922	136
21	113.0	202	40,677,242	4,226,681	1,135,595	201,372	40,088,619	1.015	83
22	87.1	201	40,710,478	4,424,484	1,563,289	202,540	39,159,777	1.040	83
X	54.4	308	144,129,712	4,538,696	1,416,623	467,954	154,893,029	0.931	112
Y	41.3	112	21,477,769	2,048,718	627,963	191,766	26,415,043	0.813	26

* Chromosome

Supplementary Table 4. Comparison of the assembled contigs with published datasets.

	PRJNA253696	CHM1_1.1	JPN00001_1.0	X1_1.1	AK1_v2
Sample	NA12878	CHM1	JPN00001	HX1	AK1
Ploidy	Diploid	Haploid	Diploid	Diploid	Diploid
Sequencer	PacBio	Illumina	PacBio	PacBio	PacBio
Scaffolding	BioNano	BAC clones	–	BioNano	BioNano<br>10x Genomics
PacBio chemistry	XL-C2, P5-C3	-	P6-C4	P6-C4	P6-C4
Coverage	44.0x	100x	101x	103x	101x
Total scaffold length	3,176,574,379	3,037,866,619	-	2,934,082,568	2,904,207,228
Total contg length	3,030,222,093	2,827,653,301	3,005,226,201	2,894,741,085	2,866,867,749
# contigs	18,903	40,828	8,963	9,348	3,096
N50 contig length	1,557,716	143,936	2,063,069	8,325,004	18,080,262
Average contig length	142,699	69,148	335,292	309,664	925,991
Maximum contig length	10,883,701	7,163,879	8,980,851	38,177,111	76,477,139
References	Pendleton et al. 2015	Steinberg et al. 2015	Our study	Shi et al. 2016	Seo et al. 2016
Accession number	GCA_001013985.1	GCA_000306695.2	–	GCA_001708065.2	GCA_001750385.2

Supplementary Table 5. Estimated allele frequency of 871 biallelic TMMINSs in international populations.

TMMINS	i1000g_af	ACB	BEB	CEU	CHB	CLM	1KJPN	JPT	KHV	YRI
13	0.1706	0.1032	0.18	0.25	0.1571	0.1917	1	0.2237	0.1316	0.2041
18	0.9981	1	1	1	1	1	1	1	1	1
57	0.9974	1	1	1	1	1	1	1	1	1
62	0.6755	1	1	1	1	1	1	1	1	1
63	0.844	1	1	1	1	1	1	1	1	1
64	0.4588	1	1	1	1	1	1	1	1	1
66	0.1419	0.1127	0.3519	0.1078	0.0702	0.1059	0.0861	0.0887	0.1923	0.0634
67	0.2567	0.3	0.1964	0.2553	0.2755	0.1593	0.265	0.2969	0.3642	0.2407
71	0.1039	0.0784	0.1442	0.1939	0.0301	0.1103	0.9991	0.1029	0.0556	0.1563
74	0.6365	1	1	1	1	1	1	1	1	1
81	0.9599	0.8182	1	1	1	0.9945	1	1	1	0.9
84	0.6639	1	1	1	1	1	1	1	1	1
87	0.6904	1	1	1	1	1	1	1	1	1
88	0.7004	1	1	1	1	1	0.9972	1	1	1
89	0.8438	1	1	1	1	1	1	1	1	1
93	0.25	0.2302	0.2857	0.4182	0.1136	0.1887	1	0.2426	0.1406	0.3944
94	0.9987	1	1	1	1	1	1	1	1	1
100	0.5288	1	1	1	1	1	1	1	1	1
101	0.5098	1	1	1	1	1	1	1	1	1
102	0.9803	1	1	1	1	1	0.9981	1	1	1
104	0.9809	1	1	1	1	1	1	1	1	1
115	0.9847	0.9451	1	1	1	0.989	1	1	1	0.9537
125	0.79	1	1	1	1	1	0.9961	1	1	1
128	0.9115	0.9217	0.8824	0.9421	0.88	0.9444	0.9495	0.9427	0.9096	0.9087
131	0.0172	0.013	0.0321	0.0441	0.0055	0.0195	0.9933	0	0.0278	0.0057
140	0.8947	0.9535	0.8675	0.9737	0.83	0.9579	0.8231	0.8505	0.8072	0.9266
149	0.7767	1	1	1	1	1	1	1	1	1
156	0.0349	0.0176	0.0375	0.0139	0.0172	0.0625	0.0005	0.0517	0.0741	0.0096
157	0.7685	1	1	1	1	1	1	1	1	1
163	0.6328	0.8667	0.75	0.7857	0.7222	0.8	1	0.6429	0.7778	0.8684
164	0.0941	0.0987	0.0663	0.2031	0.1	0.0843	0.0815	0.0795	0.0128	0.1603
169	0.8196	0.9048	0.6744	0.5053	0.9608	0.6389	0.9542	0.9752	0.9012	0.9861
172	0.1926	0.0479	0.2231	0.2857	0.25	0.307	1	0.2295	0.2328	0.0526
187	0.258	0.1207	0.4176	0.2737	0.285	0.2151	0.3927	0.3918	0.247	0.1435
192	0.4272	0.6071	0.4275	0.7672	0.3407	0.6667	0.4316	0.4023	0.3333	0.5519
193	0.0698	0.0577	0.0405	0.0933	0.0797	0.1	0.2241	0.0875	0.0625	0.0267
200	0.9241	1	1	1	1	1	1	0.9895	1	0.99
201	0.1042	0.0407	0.0513	0.0873	0.1753	0.0966	0.207	0.142	0.1753	0.0813
203	0.9896	1	1	1	1	1	0.9981	1	1	1
211	0.9853	0.9941	0.9941	0.9516	1	0.956	1	0.995	1	1
220	0.9505	0.8352	1	1	1	0.9894	1	1	1	0.8045
221	0.209	0.3288	0.05	0.0655	0.3012	0.1728	0.305	0.338	0.2121	0.3264
224	0.7766	0.7651	0.7824	0.9842	0.7842	0.9529	0.7339	0.6923	0.6603	0.6733
227	0.3163	0.4348	0.48	0.2778	0.4	0.2969	0.9991	0.125	0.4706	0.2558
229	0.3963	0.5556	0.6	0.8	0.6667	0.6	1	0.75	0.8333	0.7778
231	0.3895	0.3333	0.4091	0.4762	0.4231	0.4559	0.3942	0.3438	0.5403	0.4167
236	0.6983	0.8373	0.5581	0.6198	0.6919	0.625	0.6989	0.7071	0.6914	0.8619
279	1	1	1	1	1	1	1	1	1	1
281	0.5857	1	1	1	1	1	1	1	1	1
285	0.9704	1	1	1	1	1	0.9991	1	1	1
298	0.886	0.6494	0.9826	0.9583	1	0.9783	0.9986	0.995	0.9702	0.6009
300	0.951	0.9935	0.8176	0.9195	1	0.9425	0.9977	0.9949	1	0.9804
315	0.9127	1	1	1	1	1	1	1	1	1
319	0.9789	1	1	0.9895	1	0.994	1	1	1	1
324	0.044	0.0057	0.0294	0.0427	0.0957	0.0109	0.1001	0.0561	0.0864	0.0266
330	0.4095	0.4394	0.5488	0.5577	0.5313	0.6538	1	0.4535	0.5833	0.4697
339	0.1452	0.1786	0.2	0.2561	0.0643	0.0776	0.9962	0.119	0.1702	0.1915
348	0.7358	1	1	1	1	1	0.9991	1	1	1
357	0.25	0.4609	0.1781	0.0968	0.1823	0.2711	0.1621	0.234	0.2438	0.5625
361	0.5923	NULL	0.8571	0.8333	1	0.9	1	0.875	0.9	0.9167
363	0.9786	1	1	1	1	1	0.9981	1	1	1

369	0.3	0.4855	0.2313	0.2895	0.321	0.3165	0.289	0.1818	0.25	0.5181
371	0.7417	1	1	1	1	1	0.9991	1	1	1
372	0.3231	0.3908	0.2184	0.2604	0.3529	0.3022	0.3053	0.32	0.3882	0.367
375	0.0671	0.0181	0.125	0.1304	0.0301	0.0625	0.0393	0.0974	0.0361	0.0821
377	0.0909	0.0542	0.0513	0.1842	0.0753	0.1379	1	0.0798	0.0556	0.1
378	0.8191	0.9667	0.9744	0.9537	0.9138	0.9756	0.9991	0.9844	0.939	0.9746
390	0.2333	0.1707	0.3088	0.3542	0.2566	0.253	0.2695	0.2202	0.2192	0.2078
395	0.7252	0.9253	0.6092	0.6042	0.7087	0.6421	0.5887	0.5833	0.7151	0.9909
403	0.0931	0.0813	0.0549	0.2024	0.087	0.0714	0.0005	0.1429	0.0353	0.1557
418	0.959	1	1	1	1	1	1	1	1	1
426	0.1142	0.2	0.0671	0.0924	0.1279	0.0852	0.1928	0.2089	0.0533	0.2895
442	0.2723	0.3882	0.3038	0.25	0.3526	0.1941	0.2505	0.1576	0.2905	0.3656
450	0.2716	0.2805	0.4082	0.4608	0.2606	0.3571	0.5758	0.3061	0.1212	0.2319
459	0.4779	0.6452	0.5172	0.6452	0.6857	0.7206	1	0.7262	0.6429	0.5833
466	0.2806	0.1667	0.3256	0.369	0.3558	0.3462	0.998	0.2404	0.2826	0.2344
473	1	1	1	1	1	1	1	1	1	1
482	0.1937	0.1429	0.2143	0	0.1	0.1481	1	0.1667	0.4615	0.1818
483	0.9394	NULL	1	1	1	1	1	1	1	1
488	0.3002	0.5083	0.3462	0.22	0.2927	0.4257	0.1276	0.1371	0.1688	0.4762
489	0.2626	0.2778	0.3065	0.5091	0.2246	0.2193	1	0.3627	0.13	0.2583
490	0.7791	0.8167	0.75	0.675	0.9753	0.6522	0.9995	0.9268	0.9453	0.759
494	0.4345	0.5823	0.3072	0.3737	0.3614	0.4253	0.493	0.4896	0.2952	0.7216
508	0.6344	0.6149	0.6386	0.7528	0.5909	0.6935	0.5645	0.601	0.6446	0.5841
518	0.9498	0.9434	0.9609	0.9867	0.9625	0.9923	0.9371	0.9408	0.9915	0.9733
519	1	1	1	1	1	1	0.9376	1	1	1
572	0.1759	0.1867	0.0833	0.1607	0.3415	0.1176	0.2697	0.2917	0.2231	0.1854
575	0.2224	0.1914	0.4538	0.2933	0.3704	0.1402	0.3792	0.2805	0.3818	0.0425
576	0.7475	1	1	1	1	1	1	1	1	1
581	0.9742	1	1	1	1	1	1	1	1	1
585	0.1102	0.0714	0.128	0.1121	0.0872	0.0964	0.0192	0.1585	0.0455	0.1908
588	0.4029	0.7045	0.3448	0.276	0.2039	0.3737	0.3025	0.299	0.2558	0.7409
592	0.2702	0.2174	0.3889	0.3971	0.3387	0.3162	0.3701	0.2692	0.2643	0.2119
596	0.9333	0.9615	0.8701	0.8427	0.9946	0.859	1	0.9889	1	0.9899
605	0.3001	0.1897	0.2765	0.3353	0.4271	0.2903	0.352	0.3265	0.4568	0.1422
616	0.988	1	1	1	1	1	1	1	1	1
621	0.9895	1	1	1	1	1	1	1	1	1
623	0.498	0.6648	0.4023	0.2604	0.5049	0.4053	0.4477	0.4902	0.4767	0.7455
632	0.2361	0.24	0.1842	0.2963	0.2945	0.2632	0.3767	0.3235	0.2597	0.1953
644	0.9855	0.9477	1	1	1	0.9891	1	1	1	0.9591
646	0.9659	1	1	1	1	1	1	1	1	1
651	0.6168	0.5563	0.7171	0.7753	0.485	0.8086	0.5743	0.5417	0.6474	0.5594
652	1	1	1	1	1	1	1	1	1	1
658	0.4057	0.2727	0.4667	0.5217	0.4848	0.4487	0.9991	0.4107	0.375	0.3523
666	0.888	1	1	1	1	1	0.999	1	1	1
669	0.1659	0.1474	0.2698	0.2308	0.1585	0.2237	0.1711	0.1667	0.127	0.14
670	0.9987	1	1	1	1	1	1	1	1	1
700	0.5804	1	1	1	1	1	0.9953	1	1	1
710	0.6849	1	1	1	1	1	0.9991	1	1	1
713	0.651	0.8182	0.5422	0.5533	0.6047	0.5372	0.6131	0.6607	0.6118	0.8882
719	0.93	1	1	1	1	1	1	1	1	1
721	1	1	1	1	1	1	1	1	1	1
728	0.9576	1	1	1	1	1	1	1	1	1
738	0.3445	0.3288	0.3264	0.2882	0.4471	0.321	0.418	0.4458	0.3214	0.4655
739	1	1	1	1	1	1	1	1	1	1
744	0.6558	0.4886	0.7184	0.9202	0.6602	0.8548	0.6373	0.5931	0.6059	0.4682
755	0.8996	1	1	1	1	1	0.9991	1	1	1
758	0.6486	0.7151	0.5904	0.6011	0.6429	0.7167	0.6415	0.6302	0.7202	0.6143
763	0.9947	1	1	1	1	1	1	1	1	1
765	0.9763	1	1	1	1	1	1	1	0.9933	1
770	0.9944	1	1	1	1	1	0.9972	1	1	1
771	0.2848	0.3485	0.2857	0.3913	0.2761	0.2388	0.243	0.1623	0.3067	0.442
772	0.9428	0.881	0.9593	0.9948	0.965	0.9725	0.9402	0.9316	0.9464	0.9455
775	0.2942	0.3378	0.3583	0.3917	0.2937	0.4091	0.999	0.2045	0.2581	0.2917
778	0.1377	0.0714	0.0774	0.1705	0.2278	0.0739	0.2855	0.2386	0.1538	0.1262
786	0.7112	1	1	1	1	1	0.9981	1	1	1
789	0.2135	0.303	0.2671	0.2414	0.1579	0.1358	0.1806	0.2267	0.1267	0.4154

791	0.7337	1	1	1	1	1	1	1	1	1
795	0.0774	0.037	0.0902	0.1667	0.0385	0.04	0.9962	0.1316	0.0915	0.0758
808	0.9847	1	1	1	1	1	0.9932	1	1	1
810	0.9932	1	1	1	1	1	1	1	1	1
811	0.0238	0.0075	0.06	0.0341	0.0137	0.02	1	0.0143	0.0089	0.0476
812	0.9881	0.9828	1	1	1	1	1	0.995	0.994	0.9861
814	0.99	0.9699	1	1	1	0.9947	1	0.995	1	0.9626
816	0.9985	1	1	1	1	1	0.9995	1	1	1
821	0.9814	1	1	1	1	1	1	1	1	0.9934
823	0.183	0.1951	0.1324	0.2246	0.2468	0.1866	0.2801	0.1148	0.1304	0.2857
830	0.1527	0.277	0.1543	0.1136	0.0423	0.1488	0.1474	0.1173	0.0949	0.4196
831	0.9671	1	0.9868	0.9121	0.9947	0.9568	0.9991	0.9889	0.9861	0.9757
841	0.4071	0.1186	0.3962	0.6081	0.5377	0.5741	0.9991	0.5698	0.5667	0.1032
846	0.2774	0.233	0.1494	0.0469	0.4903	0.2228	0.5621	0.5842	0.314	0.1591
848	1	1	1	1	1	1	0.9967	1	1	1
865	0.4561	0.1628	0.5625	0.4267	0.6479	0.4831	0.7962	0.7037	0.6467	0.1141
869	0.0894	0.1136	0.0698	0.1737	0.034	0.1421	0.0458	0.0347	0.0291	0.1182
876	0.5985	1	1	1	1	1	1	1	1	1
880	0.9712	1	1	1	1	1	1	1	1	1
886	0.9935	1	1	1	1	1	0.9991	1	1	1
887	0.5016	0.58	0.8111	0.7955	0.5926	0.75	1	0.5484	0.75	0.5571
893	0.1781	0.1125	0.1583	0.226	0.1324	0.1667	0.4009	0.25	0.1957	0.2143
897	0.9915	1	1	1	1	1	1	1	1	1
902	0.0436	0.0059	0.037	0.098	0.1145	0.0167	0.0492	0.0617	0.0183	0.0197
909	0.3835	1	1	0.8667	0.75	0.8889	1	0.8333	1	0.6818
910	0.3293	0.3182	0.3571	0.4	0.34	0.3977	0.9985	0.3125	0.375	0.2935
913	0.9987	1	1	1	1	1	1	1	1	1
916	0.3677	0.5959	0.3765	0.5495	0.1373	0.4416	0.2414	0.1804	0.1845	0.6146
918	0.4232	0.5643	0.623	0.625	0.2121	0.5	0.4388	0.3977	0.3038	0.592
919	0.1245	0.0964	0.0688	0.2848	0.086	0.1294	0.9972	0.1386	0.0974	0.1559
923	1	1	1	1	1	1	1	1	1	1
931	0.1034	0.0479	0.1038	0.2286	0.0882	0.0968	0.9991	0.1455	0.1667	0.0323
943	0.9876	1	1	1	1	1	1	1	1	1
944	0.3467	0.3412	0.1786	0.2556	0.4175	0.3736	0.5048	0.4286	0.4	0.3846
946	0.9893	1	1	1	1	1	1	1	1	1
948	0.8422	1	1	1	1	1	1	1	1	1
956	0.3025	0.2229	0.2976	0.2368	0.4175	0.2935	0.3669	0.3485	0.3198	0.285
966	0.8695	1	1	1	1	1	1	1	1	1
971	0.1805	0.2222	0.1538	0.1176	0.197	0.1818	0.1638	0.1831	0.16	0.3088
978	0.7679	1	1	1	1	1	1	1	1	1
979	0.7146	1	1	1	1	1	1	1	1	1
986	0.9959	1	1	1	1	1	0.9676	1	1	1
1002	0.9986	1	1	1	1	1	1	1	1	1
1012	0.6304	1	1	1	1	1	0.9981	1	1	1
1013	0.3583	0.5246	0.4	0.2404	0.37	0.4692	0.9923	0.2833	0.4804	0.4245
1015	0.9056	0.9884	0.9762	0.9211	0.8495	0.8846	0.8929	0.8571	0.8049	0.9905
1016	0.9826	1	1	1	1	1	1	1	1	1
1017	0.9986	1	1	1	1	1	0.9991	1	1	1
1019	0.9787	1	1	0.9202	1	0.954	1	1	1	0.9953
1029	0.1608	0.0588	0.2817	0.1316	0.189	0.1867	0.3121	0.2244	0.2292	0.0294
1031	0.6667	0.6567	0.6293	0.6892	0.7113	0.8095	0.7611	0.65	0.7018	0.6438
1032	0.9483	0.9128	0.9588	0.974	0.9412	0.9628	0.979	0.9853	0.9477	0.9318
1039	0.2901	0.1506	0.3228	0.3478	0.3838	0.3571	0.4411	0.4341	0.2848	0.1019
1049	0.2374	0.1712	0.1056	0.4357	0.244	0.1118	0.8531	0.3958	0.1905	0.3961
1053	0.0949	0.0114	0.0647	0.25	0.0632	0.125	0.1648	0.0895	0.0938	0.0857
1055	0.4677	0.4726	0.3933	0.4156	0.6382	0.4384	0.6827	0.5786	0.619	0.4451
1085	0.9288	0.8831	1	0.9615	0.9677	0.95	0.9767	0.9219	0.9625	0.8868
1086	0.2431	0.3506	0.1034	0.0729	0.3058	0.0798	0.3175	0.3137	0.3605	0.3426
1094	0.7784	0.6294	0.8354	0.6117	0.9947	0.7253	0.9588	0.9628	0.9857	0.6748
1095	0.2336	0.121	0.4167	0.1818	0.1961	0.2466	1	0.2946	0.3302	0.125
1102	0.6867	1	1	1	1	1	1	0.9902	1	1
1103	0.9566	1	1	1	1	1	1	1	1	1
1106	0.9953	1	1	1	1	1	1	1	1	1
1108	0.9913	1	1	1	0.9951	1	1	1	1	1
1110	0.4411	0.5422	0.408	0.4796	0.4333	0.5526	0.3413	0.3086	0.3547	0.4671
1113	0.9692	1	1	1	1	1	0.9991	1	1	1

1116	0.2348	0.2529	0.2262	0.1	0.3529	0.0924	0.3982	0.305	0.2927	0.271
1117	0.7223	1	1	1	1	1	1	1	1	1
1119	0.3931	0.1307	0.5756	0.3723	0.5859	0.5368	0.4127	0.4406	0.3895	0.1402
1123	0.2971	0.3966	0.2384	0.2447	0.201	0.4355	0.2638	0.2941	0.2151	0.3624
1124	0.9848	1	1	1	1	1	1	1	1	1
1130	0.2789	0.4178	0.2683	0.339	0.2011	0.3232	0.2617	0.1739	0.2099	0.4231
1139	0.2858	0.5526	0.2071	0.1712	0.2563	0.3148	0.2152	0.2101	0.4214	0.3889
1143	0.2209	0.125	0.1389	0.3944	0.1692	0.2241	0.9991	0.2833	0.1757	0.254
1152	0.9444	0.9598	0.9138	0.8474	0.9903	0.8883	1	0.995	1	0.9725
1154	0.1346	0.0547	0.1182	0.2432	0.1296	0.1803	0.9995	0.1667	0.181	0.0556
1156	0.1299	0.1119	0.1066	0.1364	0.1133	0.2143	0.9971	0.1039	0.1452	0.1282
1160	0.2673	0.069	0.2439	0.3387	0.4355	0.3506	0.5005	0.4624	0.2222	0.0688
1161	0.1158	0.025	0.1186	0.2209	0.1231	0.1	0.999	0.1019	0.0333	0.2239
1163	0.1857	0.1154	0.1216	0.1845	0.4417	0.1875	0.3816	0.2465	0.3548	0.1348
1167	0.7016	0.7153	0.6467	0.5215	0.8708	0.488	0.8573	0.8554	0.8354	0.7732
1175	0.2459	0.5556	0.2647	0.5862	0.044	0.4107	0.1312	0.0739	0.2153	0.6016
1176	0.7182	0.9368	0.5581	0.6771	0.6716	0.6702	0.605	0.5882	0.7035	0.9227
1182	0.9735	1	1	1	1	0.9942	1	1	1	1
1191	0.1658	0.12	0.1486	0.1111	0.2313	0.1221	0.2899	0.3108	0.1813	0.1216
1196	0.0265	0	0.0064	0	0.0974	0.0057	0.061	0.0615	0.0316	0
1197	0.0946	0.2384	0.023	0.1848	0.0049	0.0824	0.0574	0.0686	0.0058	0.1651
1198	0.1245	0.0172	0.1	0.0723	0.325	0.0266	0.2716	0.1647	0.338	0.0464
1199	0.1227	0.0284	0.0977	0.0052	0.301	0.0053	0.2162	0.2451	0.2882	0.0136
1200	0.9914	1	1	1	1	1	0.9991	1	1	1
1203	0.996	1	1	1	1	1	0.9991	1	1	1
1211	0.9894	1	1	1	1	1	0.9995	1	1	1
1214	0.777	0.9207	0.82	0.4314	0.7971	0.7093	0.8431	0.7292	0.8253	0.9014
1218	0.7901	0.6364	0.8046	0.6146	0.9903	0.6649	0.9972	0.9951	1	0.6273
1219	0.1211	0.0811	0.0643	0.2742	0.0305	0.1014	1	0.1742	0.0563	0.2254
1226	0.9866	1	1	1	1	1	1	1	1	1
1230	0.4211	0.3487	0.5	0.5133	0.4511	0.5373	0.5222	0.4489	0.5769	0.3564
1245	0.9986	1	1	1	1	1	1	1	1	1
1260	0.9969	1	1	1	1	1	0.9991	1	1	1
1261	0.5959	0.4253	0.5575	0.7031	0.7621	0.6398	0.7031	0.74	0.5765	0.3591
1267	0.2381	0.3162	0.1688	0.1397	0.3415	0.0989	0.2979	0.3671	0.1806	0.4706
1274	0.7426	1	1	1	1	1	0.9958	1	1	1
1279	0.9885	1	1	1	1	1	0.9991	1	1	0.9856
1310	0.9961	1	1	1	1	1	1	1	1	1
1314	0.2215	0.2574	0.2986	0.2432	0.1633	0.2846	0.9937	0.1792	0.2955	0.2787
1318	0.9968	1	0.9925	0.9942	1	0.9848	1	1	1	1
1334	0.4377	0.3158	0.4928	0.5	0.5688	0.4474	0.6704	0.5946	0.5441	0.3466
1336	0.4774	0.451	0.4196	0.5385	0.6379	0.4231	1	0.6731	0.6122	0.5583
1343	0.7204	0.5435	0.8265	0.8833	0.8981	0.8019	0.989	0.8558	0.8214	0.5714
1351	0.365	0.4219	0.4407	0.4556	0.2676	0.4531	0.441	0.4167	0.3226	0.3651
1354	1	1	1	1	1	1	0.9991	1	1	1
1359	0.985	0.9828	0.9943	0.9526	1	0.9579	1	1	1	0.9909
1365	0.2948	0.1429	0.3214	0.2083	0.5694	0.2662	0.5638	0.4468	0.6071	0.1727
1368	0.0667	0.0542	0.0926	0.1071	0.0112	0.0659	0.0098	0.0733	0.0309	0.1286
1373	0.1619	0.1857	0.1594	0.3448	0.093	0.1299	0.5761	0.1613	0.1392	0.3714
1377	0.3854	0.2215	0.4359	0.3936	0.5108	0.3412	0.6527	0.5934	0.3684	0.2571
1378	0.0898	0.1618	0.0319	0.1364	0	0.1034	0.0813	0.0735	0.0288	0.25
1387	0.2348	0.226	0.1389	0.3367	0.4097	0.2403	0.3047	0.3017	0.3485	0.1667
1390	0.3883	0.4219	0.4	0.4	0.3571	0.4063	0.9981	0.4348	0.4375	0.371
1394	0.2102	0.25	0.2963	0.3913	0.1094	0.1959	0.9971	0.1286	0.1373	0.2797
1406	0.9112	0.9828	0.8391	0.8211	0.9559	0.7947	0.923	0.9314	0.9588	0.9953
1408	1	1	1	1	1	1	0.9991	1	1	1
1409	0.2031	NULL	1	0.9167	1	1	1	0.5	NULL	1
1410	0.9966	1	1	1	1	1	1	1	1	1
1413	0.9675	1	1	1	1	1	0.9903	1	1	1
1417	0.5482	0.5	0.592	0.3385	0.6893	0.5319	0.6106	0.549	0.6105	0.5727
1419	0.2477	0.1231	0.254	0.1949	0.3469	0.2742	1	0.4273	0.5098	0.1824
1423	0.4435	1	1	1	1	1	0.999	1	1	0.98
1443	0.8462	1	1	1	1	1	0.9991	1	1	1
1482	0.5195	0.0739	0.7654	0.6383	0.7323	0.4886	0.7991	0.8177	0.8092	0.0182
1487	0.4434	0.3793	0.4023	0.3936	0.4949	0.4326	0.5336	0.4646	0.4882	0.4954
1492	0.0952	0.1024	0.0402	0.0323	0.0978	0.043	0.2284	0.1538	0.1688	0.14

1497	0.0829	0.037	0.0779	0.0932	0.0787	0.1456	0.0654	0.0723	0.0438	0.1467
1503	0.2211	0.142	0.2239	0.4028	0.2958	0.3165	0.2642	0.2955	0.2	0.0797
1504	0.2564	0.1807	0.2803	0.4921	0.2688	0.3421	0.2688	0.2831	0.2697	0.1563
1506	0.1244	0.0479	0.0855	0.0536	0.1962	0.0655	0.3341	0.3115	0.2059	0.0956
1512	0.6276	0.8551	0.6875	0.593	0.5729	0.6096	0.5586	0.4767	0.625	0.8988
1516	0.8892	0.8011	0.9302	0.7526	0.9691	0.8967	0.9921	0.9802	1	0.8303
1528	0.3845	0.3457	0.5247	0.4844	0.4158	0.5179	0.375	0.3191	0.3671	0.1972
1530	0.2873	0.3161	0.1609	0.0521	0.4412	0.1526	0.4519	0.4059	0.4405	0.3227
1536	0.5456	0.4375	0.7356	0.6667	0.4951	0.6526	0.4472	0.4608	0.4593	0.4773
1537	1	1	1	1	1	1	1	1	1	1
1539	0.072	0.0833	0.0123	0.1058	0.0291	0.125	0.0609	0.0974	0.0183	0.1316
1540	0.466	0.6705	0.3953	0.151	0.4505	0.3789	0.4454	0.465	0.4765	0.7318
1543	0.6055	0.3563	0.5893	0.3385	0.9069	0.5707	0.887	0.905	0.8631	0.3455
1547	0.6395	0.5774	0.5235	0.4787	0.6818	0.6033	0.7972	0.7784	0.7143	0.729
1549	0.9861	1	1	1	1	1	1	1	1	1
1556	0.681	0.4156	0.75	0.7885	0.8537	0.8333	0.9276	0.9167	0.7722	0.3672
1558	0.5667	0.7256	0.6173	0.484	0.3737	0.6707	0.4788	0.5202	0.4063	0.8182
1566	0.986	0.994	0.9765	0.9565	1	0.9892	1	1	1	1
1594	0.9579	0.9922	0.9918	0.9841	0.9863	0.9737	1	0.9923	0.9732	1
1626	0.7959	0.7824	0.7321	0.5815	0.9541	0.6978	0.9117	0.915	0.9878	0.7952
1638	0.202	0.2419	0.2612	0.2	0.1667	0.1753	0.2402	0.3049	0.0821	0.2179
1639	1	1	1	1	1	1	0.9849	1	1	1
1640	0.8763	0.625	1	1	1	0.9415	0.9995	1	0.9941	0.5091
1642	0.5335	0.6087	0.6226	0.4746	0.7542	0.4462	1	0.7073	0.6548	0.6455
1650	0.0949	0.0111	0.1103	0.189	0.0688	0.1447	1	0.0508	0.0889	0.064
1659	0.0526	0.0872	0.1059	0.0214	0.0213	0.0106	0.0215	0.0455	0.0235	0.1084
1664	0.9656	0.9561	0.947	1	0.9941	1	1	0.9797	0.9828	0.9747
1668	0.0091	0	0.0615	0	0	0	1	0	0.0159	0
1673	0.08	0.0588	0.0645	0.1111	0.0161	0.0833	0.9991	0.0887	0.0616	0.175
1675	0.6135	0.7065	0.619	0.4615	0.7879	0.4333	0.966	0.7581	0.8804	0.5417
1676	0.9986	1	1	1	1	1	1	1	1	1
1680	0.9182	0.9659	0.8824	0.7273	0.9943	0.8138	1	1	1	0.9831
1686	0.2435	0.1733	0.3077	0.3936	0.2353	0.3662	0.2676	0.2692	0.2095	0.1806
1704	0.215	0.2469	0.2785	0.256	0.1094	0.2586	0.1904	0.1023	0.2342	0.3316
1706	0.8804	0.9423	0.86	0.8	0.9758	0.8333	0.9995	0.9831	0.8913	0.9583
1716	0.5624	0.7083	0.6667	0.7604	0.6557	0.6889	1	0.6585	0.5463	0.678
1718	1	NULL	1	1	1	1	1	1	NULL	1
1737	0.9597	1	1	1	1	1	1	1	1	1
1738	0.9946	1	1	1	1	1	1	1	1	1
1744	0.6315	0.75	0.6744	0.8194	0.6618	0.5	1	0.6667	0.575	0.6286
1756	0.9869	1	1	1	1	1	1	1	1	1
1766	0.8904	0.8607	0.9844	0.8813	0.9875	0.863	1	0.9539	0.9815	0.8594
1769	0.2781	0.2768	0.2698	0.4063	0.2035	0.2439	1	0.189	0.2857	0.4198
1772	0.6237	0.8312	0.4792	0.4835	0.6647	0.5733	0.7374	0.6854	0.4803	0.8693
1774	0.7763	1	1	1	1	1	0.9972	1	1	1
1782	0.6321	NULL	1	0.675	0.8333	0.625	0.9962	0.6	1	0.6364
1785	0.8233	0.7857	0.9178	0.9529	0.7753	0.925	0.7193	0.712	0.7805	0.7959
1786	0.1133	0.0658	0.1087	0.0902	0.179	0.0976	0.2299	0.1563	0.1324	0.1197
1789	0.3476	0.3276	0.3966	0.3777	0.3529	0.5217	0.2479	0.2277	0.3023	0.3056
1792	0.9893	1	1	1	1	1	0.9986	1	1	1
1793	0.5882	0.8059	0.5862	0.5158	0.5343	0.4894	0.5434	0.5644	0.3941	0.8227
1797	0.9741	1	1	1	1	1	1	1	1	1
1800	0.8389	1	1	1	1	1	0.9952	1	1	1
1835	0.3025	0.3026	0.4203	0.575	0.1684	0.5071	0.2898	0.2771	0.2303	0.2262
1838	0.1686	0.3861	0.0422	0.0349	0.1368	0.0435	0.1289	0.1868	0.1824	0.4514
1847	0.3852	0.1852	0.4091	0.49	0.4375	0.3684	0.9981	0.5364	0.5732	0.1985
1848	0.2444	0.2101	0.1554	0.47	0.2945	0.1731	0.3987	0.3425	0.2029	0.2803
1850	0.9985	1	1	1	1	1	0.9981	1	1	1
1860	0.6852	1	1	1	1	1	1	1	1	1
1863	0.9809	0.9675	1	1	1	1	1	1	1	0.9643
1865	0.8273	1	1	1	1	1	0.9981	1	1	1
1898	0.9967	1	1	1	1	1	0.9981	1	0.9942	1
1900	0.9516	1	1	1	1	1	0.9962	1	1	1
1904	0.9946	1	1	1	1	1	1	1	1	1
1907	0.9404	1	1	1	1	1	1	1	1	1
1908	0.4985	0.4408	0.6429	0.6929	0.4402	0.5897	0.5266	0.4773	0.4527	0.4897

1909	0.989	1	1	0.994	1	0.9921	0.9981	1	1	1
1918	0.9987	1	1	1	1	1	1	1	1	1
1920	1	1	1	1	1	1	1	1	1	1
1921	0.3793	0.5059	0.3333	0.4053	0.265	0.2926	0.3763	0.3317	0.3353	0.5545
1923	0.1038	0.0902	0.1771	0.2333	0.0809	0.0571	0.9971	0.0789	0.0909	0.1016
1926	0.0782	0.0227	0.046	0.2604	0.0631	0.1632	0.0672	0.0343	0.0291	0.0091
1936	0.2705	0.4	0.2467	0.1981	0.2759	0.1829	0.3028	0.3158	0.2857	0.3514
1942	0.0422	0.0057	0.0116	0.0172	0.1117	0	0.0935	0.0957	0.0882	0.014
1951	0.9951	1	1	1	1	1	0.9957	1	1	1
1953	0.5585	1	1	1	1	1	0.9991	0.9565	1	0.9688
1954	0.9955	1	1	1	1	1	1	1	1	1
1955	0.9091	1	1	1	1	1	0.9981	1	1	1
1963	1	1	1	1	1	1	1	1	1	1
1966	0.9457	1	1	1	1	0.9921	0.9981	1	1	1
1972	0.9797	1	1	1	1	1	0.9991	1	1	1
1975	0.2831	0.2118	0.3735	0.343	0.4235	0.2074	0.3884	0.3586	0.2235	0.1636
1979	0.8999	0.8272	0.9815	0.8539	0.9559	0.8663	0.962	0.9747	0.9533	0.8396
1981	1	1	1	1	1	1	1	1	1	1
1985	0.2027	0.1351	0.2016	0.3452	0.1733	0.3433	0.2002	0.1389	0.1761	0.254
1989	0.1563	0.0929	0.2232	0.3056	0.0696	0.1232	1	0.1439	0.1216	0.2417
1992	1	1	1	1	1	1	0.9991	1	1	1
1994	0.3684	0.2941	0.5294	0.3425	0.7717	0.4919	0.8636	0.6279	0.859	0.25
1997	0.1498	0.1951	0.1827	0.1111	0.1346	0.13	0.9941	0.1714	0.14	0.1061
1999	1	1	1	1	1	1	0.9972	1	1	1
2001	0.2665	0.4603	0.22	0.2344	0.2407	0.1761	0.2677	0.2877	0.1692	0.5667
2006	0.9726	1	1	1	1	1	1	1	1	1
2009	0.9406	1	1	1	1	1	1.001	1	1	1
2010	0.9953	0.9942	1	1	1	1	1	1	1	1
2013	0.8487	0.9527	0.7647	0.7765	0.8198	0.82	0.9382	0.9518	0.7826	0.9778
2025	0.9774	1	1	1	1	1	1	1	1	1
2030	0.6166	0.8704	0.4884	0.5677	0.5294	0.5805	0.5579	0.495	0.5357	0.9167
2035	0.9881	1	1	1	1	1	0.9991	1	1	1
2036	0.7959	0.7132	0.8406	0.9056	0.8022	0.8286	0.854	0.8218	0.7222	0.7553
2056	0.612	0.6301	0.4286	0.4602	0.7102	0.6938	0.6894	0.628	0.8176	0.6461
2057	0.9802	1	1	1	1	1	0.9991	1	1	1
2058	0.1001	0.1386	0.1027	0.1781	0.1319	0.1205	0.1262	0.0955	0.0183	0.0645
2059	0.789	1	1	1	1	0.9906	1	0.9906	0.9848	0.9732
2060	0.9906	1	1	1	1	1	1	1	1	1
2071	0.993	1	1	1	1	1	1	1	1	0.9901
2106	0.8257	0.875	0.7816	0.9632	0.8252	0.8158	0.817	0.8529	0.686	0.8028
2108	0.4222	0.6935	0.7051	0.4545	0.82	0.6053	0.9971	0.7931	0.7143	0.8036
2111	0.529	0.6371	0.575	0.6558	0.5313	0.6667	0.5518	0.48	0.5726	0.5805
2117	0.0975	0.0952	0.1139	0.1746	0.0756	0.0591	0.0771	0.0793	0.1588	0.0461
2122	0.1547	0.3795	0.0698	0.0729	0.1324	0.1022	0.112	0.0891	0.0988	0.3204
2124	0.8364	1	1	1	1	1	0.9991	1	1	1
2126	1	1	1	1	1	1	1	1	1	1
2131	0.9305	0.8161	0.9943	0.9263	1	0.9355	1	1	1	0.7955
2135	0.7757	0.7089	0.7037	0.7234	0.89	0.744	0.8701	0.8646	0.9122	0.699
2144	0.3952	0.7244	0.1494	0.1737	0.4192	0.2181	0.3377	0.3131	0.4198	0.83
2152	0.1765	0.2281	0.1429	0.087	0.1842	0.1901	0.3186	0.2347	0.2097	0.3019
2153	0.5613	0.7464	0.5724	0.6534	0.449	0.5783	0.444	0.4278	0.3974	0.7268
2158	0.983	0.9921	1	1	1	0.9922	0.9981	1	0.9851	0.9888
2164	0.2151	0.1071	0.186	0.32	0.2838	0.2556	0.9972	0.3	0.2442	0.1333
2172	0.4483	0.7	0.6429	0.875	1	0.8333	1	0.6667	1	0.7917
2192	0.2936	0.3981	0.3333	0.15	0.2308	0.3558	0.9971	0.3375	0.3939	0.2636
2230	0.2312	0.1154	0.306	0.4508	0.0706	0.2203	0.9953	0.2838	0.1164	0.4154
2237	0.9934	1	1	1	1	1	1	1	1	1
2241	0.9901	1	1	1	1	1	1	1	1	1
2243	0.9895	1	1	1	1	1	0.9944	1	1	1
2249	1	1	1	1	1	1	0.8582	1	1	1
2254	0.5964	0.6325	0.3902	0.4421	0.8112	0.4086	0.85	0.8041	0.7183	0.6
2256	0.2236	0.3451	0.1447	0.2867	0.2013	0.1059	0.2459	0.2014	0.1481	0.5081
2258	0.1714	0.0741	0.2326	0.2429	0.225	0.1596	1	0.234	0.1413	0.1053
2261	0.8061	1	1	1	1	1	1	1	1	1
2263	0.9026	1	1	1	1	1	0.9963	1	1	1
2264	0.9882	1	1	1	1	1	0.9991	1	1	1

2268	1	1	1	1	1	1	0.9981	1	1	1
2278	0.2526	0.2045	0.4138	0.3229	0.2136	0.3351	0.2848	0.2129	0.2151	0.1364
2280	0.4993	0.5176	0.6369	0.5895	0.4175	0.5978	0.3768	0.402	0.4405	0.4626
2281	0.9986	1	1	1	1	1	1	1	1	1
2282	0.3192	0.3413	0.3806	0.4597	0.1623	0.2877	0.9971	0.3025	0.25	0.4241
2285	0.3831	0.4182	0.4554	0.5	0.1905	0.4206	1	0.3356	0.2705	0.5
2292	0.9983	1	1	1	1	1	0.9991	1	1	1
2295	0.8981	1	1	1	1	1	0.9991	1	1	0.9947
2297	0.9889	1	1	1	1	1	1	1	1	1
2299	1	1	1	1	1	1	0.9991	1	1	1
2301	1	1	1	1	1	1	1	1	1	1
2307	0.4389	0.5	0.5846	0.454	0.4277	0.3779	0.4356	0.4326	0.4706	0.5947
2310	0.362	0.3621	0.3162	0.5385	0.6379	0.3571	0.4831	0.5465	0.5192	0.3837
2314	0.6299	1	1	1	1	1	0.9991	1	1	1
2320	0.5531	0.5759	0.4605	0.3516	0.6648	0.4941	0.6276	0.599	0.6268	0.6684
2321	0.9869	1	1	1	1	1	1	1	1	1
2324	0.8416	1	1	1	1	1	1	1	1	1
2325	0.9711	1	1	1	1	1	0.9991	1	1	1
2330	0.9646	1	1	1	1	1	1	1	1	1
2332	0.9987	1	1	1	1	1	1	1	1	1
2333	0.9466	1	1	1	1	1	0.9981	1	1	1
2336	0.9707	1	1	1	1	1	1	1	1	1
2339	0.9455	1	1	1	1	1	1	1	1	1
2342	0.3081	0.4425	0.3895	0.349	0.2282	0.2527	0.1223	0.1078	0.2706	0.4404
2355	1	1	1	1	1	1	0.9971	1	1	1
2363	0.3761	0.602	0.58	0.5781	0.319	0.4643	0.3008	0.2347	0.2222	0.6719
2366	0.3519	0.5	0.4688	0.2778	0.4444	0.537	1	0.3056	0.56	0.4474
2368	0.7496	0.7	0.5972	0.821	0.8554	0.6857	0.8689	0.8506	0.8788	0.7561
2388	0.9704	1	1	1	1	1	0.9937	1	1	1
2390	0.6035	1	1	1	1	1	1	1	1	1
2398	0.9555	1	1	1	1	1	0.9942	1	1	1
2402	0.8466	0.733	0.907	0.8936	0.896	0.9309	0.8679	0.8041	0.9302	0.7176
2419	0.604	0.6146	0.7667	0.8085	0.8804	0.7625	0.9981	0.7875	0.7609	0.7759
2423	0.6964	0.7317	0.557	0.5889	0.7113	0.6875	0.7875	0.8073	0.7099	0.7961
2427	0.3151	0.3125	0.2652	0.0615	0.6491	0.1667	0.6074	0.5769	0.5	0.4141
2435	0.5048	0.6728	0.3554	0.2292	0.6364	0.3118	0.5765	0.6053	0.488	0.73
2437	0.9974	0.9943	1	1	1	1	1	1	1	0.9955
2442	0.3554	0.2813	0.3627	0.4375	0.4592	0.3469	0.9886	0.3367	0.4091	0.2917
2443	0.9748	1	1	0.9894	1	0.9941	1	1	1	1
2444	0.9822	0.9593	1	0.9896	1	0.9945	1	1	1	0.95
2450	0.7486	0.6	0.7561	0.5895	0.865	0.7528	0.9112	0.9158	0.9167	0.6587
2461	0.9929	1	1	1	1	1	1	1	1	1
2472	0.7194	0.9195	0.6105	0.6094	0.7059	0.6935	0.6769	0.665	0.5465	0.9861
2474	0.2657	0.2532	0.2171	0.3667	0.3092	0.3269	0.2845	0.3372	0.403	0.2434
2490	0.0876	0.1638	0.0617	0.1078	0.0169	0.0955	0.0836	0.0633	0.0357	0.2661
2491	0.2211	0.1164	0.3203	0.4167	0.2143	0.3462	0.2364	0.2535	0.2266	0.0962
2492	0.2738	0.3607	0.4894	0.3452	0.2338	0.2431	0.2315	0.2857	0.3103	0.3679
2497	0.3122	0.2857	0.241	0.0885	0.5	0.1978	0.5814	0.5756	0.4932	0.2913
2498	0.414	0.4167	0.43	0.4714	0.3824	0.4831	0.9971	0.3333	0.5	0.3333
2502	0.1649	0.0682	0.1149	0.0365	0.2598	0.0368	0.36	0.3529	0.3529	0.1
2513	1	1	1	1	1	1	0.9044	1	1	1
2531	0.9522	1	1	1	1	1	0.9971	1	1	1
2536	0.5273	0.5345	0.5057	0.4368	0.596	0.3681	0.7367	0.7216	0.5783	0.5094
2544	0.5145	0.7616	0.408	0.2448	0.5147	0.2158	0.6204	0.5765	0.506	0.8692
2556	0.3378	0.3333	0.4275	0.3059	0.3941	0.3	0.4248	0.3647	0.4189	0.3133
2561	0.4195	0.6333	0.5556	0.3022	0.3421	0.3276	0.3855	0.3299	0.2595	0.6875
2563	0.7681	0.5575	0.907	0.8723	0.8235	0.7857	0.8838	0.8866	0.8588	0.5505
2564	0.0068	0	0	0	0.0556	0.0072	1	0	0.0091	0
2570	0.6739	0.8333	0.8043	0.8839	0.7069	0.7935	0.9972	0.7667	0.82	0.7719
2571	0.9926	1	1	1	1	1	0.99	1	1	1
2573	0.6712	0.6081	0.6304	0.6722	0.7582	0.6131	0.7769	0.7527	0.8224	0.6667
2575	0.588	0.7024	0.6627	0.1842	0.665	0.5169	0.5971	0.5343	0.6098	0.8786
2583	0.3304	0.35	0.3768	0.3291	0.3065	0.4412	0.4603	0.4155	0.3986	0.3895
2586	0.4118	0.3452	0.3193	0.3594	0.5573	0.45	0.598	0.5904	0.4313	0.3019
2593	0.9954	1	0.9941	1	1	1	1	1	1	1
2596	0.9957	1	1	1	1	1	0.9981	1	1	1

2602	0.0365	0.023	0.0174	0.029	0.0309	0.022	0.0957	0.0843	0.0488	0.0353
2604	0.9732	0.892	1	1	1	0.9947	1	1	1	0.9045
2608	0.2982	0.3562	0.2792	0.3108	0.3118	0.1628	0.3621	0.3533	0.4071	0.3765
2611	0.7762	1	1	1	1	1	1	1	1	1
2623	0.9742	0.9667	1	1	1	0.9881	1	0.9942	1	0.9167
2624	0.9984	1	1	1	1	1	0.9972	1	1	1
2631	0.9277	1	1	1	1	1	1	1	1	1
2633	0.5648	0.2184	0.7059	0.7842	0.6814	0.75	0.7563	0.7108	0.6265	0.0963
2635	0.7392	0.9261	0.6207	0.6406	0.6748	0.6737	0.6265	0.652	0.7733	0.9409
2637	0.8642	0.7159	0.954	0.901	0.9118	0.9263	0.9449	0.9608	0.9128	0.6545
2641	0.9973	1	1	1	1	1	0.9991	1	1	1
2643	0.1854	0.1554	0.2639	0.1615	0.241	0.142	0.2394	0.1842	0.2595	0.1438
2644	0.1691	0.0063	0.2192	0.0761	0.3446	0.1627	0.2736	0.2813	0.22	0.0682
2647	0.9922	1	1	1	1	1	1	1	1	0.9727
2655	0.7243	1	1	1	1	1	1	1	1	1
2674	0.1639	0	0.25	0.2	0.75	0.0625	1	0.1667	0.1875	0.1923
2676	0.811	1	1	1	1	1	1	1	1	1
2677	0.9778	1	1	1	1	1	1	1	1	1
2682	0.3566	0.3125	0.4302	0.3125	0.301	0.3947	0.2988	0.2696	0.4535	0.4
2701	0.996	1	1	1	1	1	1	1	1	1
2718	0.9882	1	0.9598	0.974	1	0.9684	1	1	1	1
2723	0.2579	0.1552	0.2	0.224	0.3786	0.2926	0.3796	0.404	0.2831	0.1343
2775	0.0524	0.0253	0.053	0.1556	0.0441	0.0227	0.9923	0.0597	0.0462	0.0507
2783	1	1	1	1	1	1	0.9981	1	1	1
2798	0.5	0.5345	0.6591	0.5	0.5357	0.525	0.9981	0.5263	0.5952	0.5952
2805	0.1225	0.0814	0.1563	0.2606	0.099	0.2442	0.1133	0.0659	0.0952	0.0385
2811	0.9941	1	1	0.974	1	0.9946	0.9991	0.9951	1	1
2813	0.5791	1	1	1	1	1	0.9991	1	1	1
2822	0.3738	0.9643	1	0.9783	1	0.975	1	0.9815	1	1
2828	0.9886	0.9918	1	1	1	1	1	1	1	1
2831	0.1714	0.0926	0.2	0.1917	0.2688	0.1685	0.1954	0.1282	0.1884	0.1899
2834	0.9862	1	1	1	1	1	1	1	1	1
2835	0.9857	1	1	1	1	1	0.9991	1	1	1
2838	0.1932	0.4435	0.1852	0.3276	0.0545	0.1453	0.1014	0.1071	0.1111	0.4157
2841	1	1	1	NULL	1	1	0.9991	1	1	1
2842	0.0748	0.125	0.0634	0.1	0.0662	0.0577	0.0215	0.0781	0.0385	0.1071
2847	0.279	0.2545	0.3	0.5	0.1644	0.1471	0.9971	0.3158	0.1702	0.4295
2854	0.951	1	1	1	1	1	0.9991	1	1	1
2857	0.6261	1	1	1	1	1	0.9991	1	1	1
2860	0.9768	1	1	1	1	1	1	1	1	1
2864	0.9226	1	1	1	1	1	1	1	1	1
2876	0.5927	1	1	1	1	1	1	1	1	1
2882	0.8619	1	1	1	1	1	0.9951	1	1	1
2954	0.55	0.566	0.6176	0.661	0.6667	0.6204	0.9991	0.5263	0.6154	0.6087
2956	0.077	0.0542	0.0432	0.1369	0.0202	0.118	0.9981	0.044	0.061	0.1436
2957	0.6421	0.3642	0.6145	0.7849	0.7889	0.7941	0.8105	0.8023	0.7727	0.375
2969	0.3389	0.1127	0.425	0.5	0.4649	0.442	1	0.44	0.4375	0.0704
2971	0.0835	0.026	0.0764	0.1081	0.0272	0.131	1	0.0519	0.125	0.125
2976	0.9071	1	1	1	1	1	0.9991	1	1	1
2977	0.9961	1	1	1	1	1	1	1	1	1
2985	0.212	0.1452	0.1739	0.1857	0.3173	0.2742	1	0.2283	0.2609	0.1207
2986	0.5445	1	1	1	1	1	1	1	1	1
2987	0.988	1	1	1	1	1	1	1	1	1
2988	0.5273	1	1	1	1	1	1	1	1	0.9889
2989	0.5992	1	1	1	1	1	0.9991	1	1	1
2994	0.9846	1	1	1	1	1	1	1	1	1
2997	0.9874	1	1	1	0.9851	1	0.9491	0.9826	0.9877	1
3003	0.5893	NULL	1	1	NULL	1	1	1	1	1
3026	0.4375	0.34	0.5588	0.7955	0.6923	0.4722	1	0.5385	0.54	0.6757
3039	0.9875	0.993	1	1	1	1	0.9892	1	1	0.9938
3050	0.0145	0	0.0059	0.0169	0.0161	0.0105	0.0028	0.0372	0.0058	0.0206
3067	0.9705	1	1	1	1	1	0.9981	1	1	1
3068	0.6364	1	1	1	1	1	0.9934	1	1	1
3079	0.2189	0.0465	0.2933	0.4313	0.2912	0.2558	0.367	0.2053	0.3182	0.0324
3087	0.9901	1	1	1	1	1	1	1	1	1
3089	1	1	1	1	1	1	1	1	1	1

3095	0.0987	0.0774	0.0563	0.0789	0.1453	0.0598	0.1731	0.16	0.0802	0.1486
3098	0.0986	0.0549	0.0529	0.0974	0.1304	0.087	0.1272	0.1047	0.141	0.1386
3106	0.1968	0.0952	0.1818	0.3361	0.1333	0.1321	0.9971	0.3	0.2027	0.2813
3110	0.7803	1	1	1	1	1	0.9962	1	1	1
3113	0.721	0.5988	0.8512	0.7935	0.802	0.8488	0.8822	0.8485	0.679	0.4722
3135	0.723	1	1	1	1	1	1	1	1	1
3137	1	1	1	1	1	1	0.9962	1	1	1
3140	0.5309	0.7246	0.4074	0.5611	0.5625	0.4805	0.5361	0.5172	0.5806	0.7874
3148	0.9221	0.9759	0.821	0.8626	0.99	0.7558	0.9972	0.9949	0.9873	0.9747
3161	0.1404	NULL	1	0.0952	0	0.6667	1	0.05	0	0.1786
3180	0.1975	0.3165	0.1918	0.1698	0.1905	0.1519	0.2615	0.1688	0.1691	0.2534
3181	0.5114	0.4267	0.4861	0.5577	0.6576	0.487	0.6958	0.625	0.6045	0.3557
3187	0.7506	1	1	1	1	1	1	1	1	1
3190	0.0502	0.0325	0.0357	0.1204	0.0407	0.0641	0.9972	0.0133	0.0526	0.0625
3201	0.1078	0.0574	0.1058	0.2143	0.0775	0.049	0.9962	0.1034	0.0714	0.2063
3208	0.1109	0.06	0.0253	0.1385	0.1558	0.0843	0.1455	0.1776	0.1346	0.1622
3216	1	1	1	1	1	1	1	1	1	1
3219	1	1	1	1	1	1	1	1	1	1
3224	0.1074	0.0435	0.1429	0.2143	0.0486	0.1159	1	0.1304	0.1176	0.0676
3233	0.2959	0.2273	0.2849	0.2813	0.4175	0.2632	0.3287	0.385	0.2988	0.2091
3236	0.1733	0.1014	0.1452	0.2222	0.2154	0.1232	1	0.2463	0.2308	0.123
3237	0.9987	1	1	1	1	1	0.9981	1	1	1
3239	0.9622	1	1	1	1	1	1	1	1	1
3240	0.9487	0.9186	1	1	1	0.9677	1	1	1	0.7636
3242	0.9921	1	1	1	1	1	1	1	1	1
3243	0.3318	0.5	0.875	0.675	0.3333	0.6875	0.9981	0.4	0.6875	0.2188
3249	0.9783	1	1	1	1	1	1	1	1	1
3255	0.2265	0.1566	0.3971	0.1133	0.3851	0.1522	0.3048	0.2945	0.2571	0.2405
3268	0.3618	0.3868	0.725	0.4605	0.5192	0.5488	1	0.6429	0.8409	0.2358
3279	0.9509	0.9828	0.9195	0.9219	0.95	0.9574	0.9379	0.9455	0.9535	1
3286	0.6909	0.9342	0.6081	0.6105	0.6337	0.62	0.6715	0.6167	0.6188	0.9368
3287	0.9275	1	1	1	1	1	1	1	1	1
3292	0.5215	0.625	0.454	0.4688	0.4515	0.5263	0.4379	0.4706	0.4884	0.6727
3294	0.5271	0.1627	0.4702	0.5842	0.905	0.5625	0.8432	0.7903	0.7826	0.1182
3296	0.7123	0.5756	0.7831	0.7742	0.7602	0.8161	0.8494	0.8144	0.7563	0.5
3297	0.0847	0.0633	0.0976	0.0723	0.1389	0.0739	0.1112	0.0833	0.0671	0.1023
3304	0.9844	0.9851	1	0.9937	1	0.994	0.9897	0.9817	1	0.9942
3314	0.9982	1	1	1	1	1	0.9929	1	1	1
3329	0.9817	1	1	1	1	1	1	1	1	1
3332	0.48	0.6172	0.5299	0.4699	0.4157	0.4662	0.6173	0.5987	0.3786	0.6353
3333	0.5489	0.7244	0.5641	0.5109	0.433	0.4419	0.6225	0.6543	0.4125	0.732
3334	0.3465	0.3678	0.3391	0.1719	0.432	0.1436	0.5943	0.5909	0.3663	0.3455
3338	0.1466	0.1307	0.1628	0.2011	0.1188	0.1444	0.106	0.1485	0.1824	0.1065
3339	0.2471	0.2075	0.1949	0.3	0.2589	0.1875	1	0.35	0.2131	0.3333
3345	0.7148	0.7245	0.8365	0.7722	0.8051	0.7281	0.9051	0.8125	0.7857	0.7434
3347	0.4138	0.6111	0.4214	0.264	0.4261	0.4329	0.3958	0.3222	0.5267	0.625
3368	1	1	1	1	1	1	1	1	1	1
3369	0.8356	0.7529	0.9277	0.6489	0.9293	0.7384	0.986	0.9583	0.9437	0.8271
3381	0.992	1	1	1	1	1	1	1	1	1
3388	0.9617	0.8922	1	0.962	1	0.9638	1	0.9941	0.9929	0.8545
3389	0.1655	0.1938	0.1646	0.2215	0.1889	0.0647	0.2832	0.1923	0.223	0.1392
3392	0.6688	0.6477	0.6264	0.7344	0.6845	0.6064	0.7586	0.7549	0.6919	0.6091
3399	0.8034	0.6707	0.8049	0.8263	0.895	0.8412	0.8061	0.8283	0.9337	0.6651
3406	0.1894	0.2465	0.1139	0.0909	0.2284	0.1304	0.2906	0.2857	0.2333	0.2538
3422	0.7603	0.939	0.8605	0.8085	0.5874	0.7903	0.4939	0.5101	0.7083	0.9398
3423	0.3542	0.4821	0.2464	0.4136	0.3333	0.3767	0.4631	0.4813	0.363	0.6234
3424	0.5144	0.3523	0.5057	0.3947	0.5922	0.5263	0.6592	0.6683	0.657	0.4227
3428	0.3932	0.3451	0.4167	0.3766	0.539	0.4452	0.5482	0.5614	0.5159	0.4867
3429	0.4954	0.3807	0.4826	0.3646	0.5833	0.4632	0.5551	0.6238	0.593	0.4773
3433	0.4647	0.3701	0.5769	0.6	0.4242	0.6159	0.6317	0.6011	0.3397	0.3287
3439	0.355	0.4253	0.1839	0.2396	0.455	0.2872	0.4734	0.3713	0.3353	0.5333
3440	0.3596	0.4483	0.1839	0.2396	0.451	0.2766	0.4714	0.3873	0.3235	0.5377
3446	0.1182	0.1894	0.0725	0	0.1231	0.0407	0.1607	0.1364	0.1765	0.1771
3448	0.2559	0.375	0.1379	0.2316	0.1471	0.2394	0.2806	0.26	0.2093	0.4312
3468	0.0856	0.0608	0.2273	0.1613	0.0365	0.0588	0.9971	0.0305	0.0563	0.1
3483	0.9914	1	1	1	1	1	0.9981	1	1	1

3493	0.2078	0.2118	0.2134	0.1793	0.1111	0.1556	0.2044	0.3085	0.2805	0.2374
3497	0.0614	0.0484	0.0522	0.0804	0.0779	0.1154	0.4399	0.0179	0.0833	0.037
3499	0.6328	0.6131	0.6706	0.7181	0.63	0.6263	0.6583	0.6563	0.619	0.5654
3504	1	1	1	1	1	1	0.9971	1	1	1
3509	0.1038	0.1358	0.1849	0.0538	0.1278	0.0769	0.1387	0.0608	0.0962	0.1118
3513	0.0857	0.0886	0.0443	0.0444	0.1111	0.1159	0.1308	0.0795	0.1013	0.1755
3518	0.5884	0.6556	0.6875	0.4688	0.6944	0.5727	0.9981	0.7083	0.7755	0.6279
3524	0.0796	0.046	0.05	0.0614	0.2467	0.0562	0.0727	0.0602	0.0741	0.0667
3526	0.5159	1	1	1	1	1	0.9991	1	1	1
3531	0.7652	0.7215	0.8782	0.8614	0.6818	0.8596	0.7736	0.7637	0.6375	0.7704
3574	0.3704	0.266	0.5	0.3462	0.3519	0.5104	1	0.5	0.4516	0.1383
3578	0.5934	0.5529	0.7658	0.5729	0.549	0.5667	0.5748	0.6082	0.5875	0.5888
3584	0.2899	0.4261	0.1494	0.2105	0.2574	0.3564	0.2283	0.201	0.1802	0.5045
3588	0.1425	0.0407	0.2667	0.0588	0.3198	0.0852	0.1681	0.1944	0.209	0.0762
3593	0.2622	0.25	0.3558	0.4722	0.1525	0.2685	1	0.1803	0.2456	0.2719
3596	0.9577	0.8837	1	1	1	0.9837	0.9977	1	1	0.8551
3605	0.5125	0.5227	0.5233	0.6263	0.5097	0.4255	0.4641	0.4545	0.6235	0.4583
3612	0.9486	1	1	1	1	1	0.9972	1	1	1
3618	0.9846	0.9805	1	1	1	0.9942	1	1	1	0.9684
3620	0.2772	0.3623	0.1884	0.5213	0.2603	0.2929	0.2394	0.1849	0.2	0.375
3623	0.3675	0.5306	0.4362	0.4545	0.4875	0.4259	0.9961	0.4314	0.45	0.2763
3626	0.2383	0.0824	0.3082	0.3951	0.2931	0.2938	0.3178	0.3563	0.4545	0.019
3627	0.8795	0.9419	0.6494	0.9043	0.9091	0.9362	0.9248	0.915	0.8735	0.9128
3631	0.1324	0.4015	0.0823	0.0636	0.0407	0.1099	0.071	0.0926	0.0476	0.3413
3633	0.4706	0.4091	0.477	0.2656	0.6942	0.3158	0.5593	0.5446	0.6265	0.4409
3636	0.6086	0.5482	0.628	0.3789	0.78	0.4944	0.7927	0.7552	0.7439	0.5962
3640	0.2705	0.5172	0.18	0.2045	0.2969	0.2727	0.3078	0.1866	0.2429	0.5408
3649	0.9986	1	1	1	1	1	0.9981	1	1	1
3650	0.2031	0.2356	0.2765	0.125	0.199	0.2394	0.1405	0.1832	0.131	0.2409
3663	0.6524	0.6447	0.609	0.266	0.9318	0.4471	0.9934	0.9315	0.973	0.6538
3664	0.5422	0.4667	0.5093	0.4737	0.8169	0.4706	1	0.8306	0.75	0.4348
3666	0.1747	0.0581	0.2368	0.2088	0.2386	0.1839	0.2586	0.2713	0.2571	0.0467
3673	0.3961	0.5357	0.338	0.4386	0.4103	0.3561	0.4569	0.4366	0.4907	0.6439
3679	0.128	0.1481	0.0696	0.127	0.1534	0.1517	0.19	0.0909	0.2039	0.125
3698	0.5286	0.55	0.6136	0.5833	0.5385	0.5385	1	0.6406	0.6	0.5179
3700	0.995	1	1	0.9875	1	0.9938	0.9848	1	1	1
3705	0.1213	0.2836	0.057	0.0635	0.0407	0.0756	0.0734	0.1139	0.1063	0.3492
3709	0.997	1	1	1	1	1	1	1	1	1
3713	0.3291	0.375	0.4262	0.5921	0.2697	0.5242	0.2834	0.22	0.25	0.3852
3714	0.1981	0.223	0.1597	0.25	0.1667	0.1757	0.166	0.1987	0.1603	0.3361
3718	1	1	1	1	1	1	1	1	1	1
3719	0.5387	0.5123	0.4545	0.6854	0.4263	0.7857	0.4897	0.4677	0.487	0.5357
3724	0.5562	0.5185	0.5802	0.2632	0.7424	0.4494	0.7493	0.7056	0.6757	0.6436
3726	0.5804	0.4873	0.4012	0.4121	0.7632	0.4778	0.8158	0.7167	0.7533	0.6364
3741	0.574	0.2941	0.72	0.617	0.75	0.6203	0.8099	0.7798	0.7794	0.3221
3743	0.2169	0.2836	0.2	0.2407	0.2273	0.2034	0.4074	0.2973	0.2213	0.2078
3780	1	1	1	1	1	1	1	1	1	1
3781	0.2869	0.4516	0.2131	0.2024	0.3162	0.2321	0.2889	0.2955	0.3986	0.3571
3783	0.9832	0.9688	1	1	1	1	1	1	1	0.9722
3784	1	1	1	1	1	1	1	1	1	1
3785	0.8107	1	1	1	1	1	0.9981	1	1	1
3786	0.2488	0.2985	0.1493	0.4151	0.2115	0.3923	0.1988	0.1875	0.2192	0.3699
3787	0.1819	0.1477	0.4059	0.1667	0.1505	0.266	0.1186	0.0637	0.1686	0.1296
3791	0.3047	0.2857	0.3492	0.3261	0.4127	0.3108	0.3689	0.3134	0.2727	0.3154
3802	1	1	1	1	1	1	1	1	1	1
3804	0.6609	0.7188	0.7134	0.7033	0.55	0.7048	0.7117	0.6667	0.5563	0.7356
3811	0.5355	0.5455	0.5714	0.8288	0.3736	0.6458	0.4679	0.4489	0.4324	0.6882
3812	NULL	NULL	NULL	NULL	NULL	NULL	0.4626	NULL	NULL	NULL
3815	0.2593	0.1216	0.4286	0.4118	0.2568	0.2529	0.2847	0.2874	0.137	0.2188
3818	1	1	1	1	1	1	1	1	1	1
3819	0.7286	0.8038	0.7821	0.7857	0.672	0.7901	0.7075	0.6947	0.6375	0.8177
3824	0.9974	1	1	1	1	1	0.9981	1	1	1
3826	0.1171	0.2701	0.023	0.0156	0.0842	0.0316	0.1071	0.0735	0.1395	0.2917
3827	0.2387	0.15	0.1909	0.4683	0.0844	0.2109	0.9981	0.2348	0.1579	0.4179
3834	0.8052	0.8372	0.8103	0.7979	0.8186	0.8152	0.711	0.7574	0.7733	0.838
3855	0.1603	0.1131	0.0633	0.2119	0.223	0.1105	0.3304	0.2286	0.2958	0.1494

3856	0.5659	0.8793	0.5287	0.3737	0.5146	0.3632	0.4827	0.4951	0.407	0.9318
3863	0.262	0.1575	0.2845	0.1707	0.4694	0.1914	0.3805	0.4576	0.4018	0.2222
3867	0.5397	0.4051	0.6712	0.4677	0.6667	0.4318	0.7464	0.7619	0.6319	0.4279
3872	0.0366	0.025	0.0476	0.0625	0.0556	0.0167	0.0177	0.0174	0.012	0.0743
3874	1	1	1	1	1	1	1	1	1	1
3884	0.4626	0.5253	0.4706	0.5538	0.3929	0.5867	0.3749	0.3763	0.4451	0.4769
3889	0.3214	0.1534	0.2874	0.4316	0.3932	0.4894	0.3623	0.3775	0.3605	0.1
3890	0.3266	0.6509	0.1835	0.2556	0.25	0.3693	0.343	0.3098	0.2375	0.6481
3892	0.7934	0.8194	0.8052	0.956	0.7713	0.8356	0.6699	0.6647	0.7424	0.8495
3899	0.9803	0.9432	1	1	1	0.9789	1	0.9901	1	0.9541
3920	0.9829	1	1	1	1	1	1	1	1	1
3923	0.1935	0.0183	0.2533	0.1639	0.2941	0.1919	0.3107	0.2877	0.3704	0.026
3930	0.485	0.5943	0.4063	0.5625	0.57	0.5096	0.611	0.4674	0.6404	0.4048
3932	0.1528	0.1867	0.0875	0.2463	0.0952	0.2662	0.1086	0.0298	0.1646	0.1962
3941	0.9934	1	1	1	1	1	1	1	1	1
3943	0.2718	0.2055	0.3828	0.3804	0.3403	0.3333	0.3773	0.2568	0.4219	0.1056
3961	0.9462	1	1	1	1	1	0.9972	1	1	1
3978	0.2428	0.0398	0.4195	0.3125	0.2379	0.2766	0.381	0.3725	0.314	0.0045
3982	0.9865	1	1	1	1	1	1	1	1	1
4007	0.6551	1	1	1	1	1	1	1	1	1
4017	0.8668	0.7931	0.9709	0.9948	0.8578	0.9731	0.8894	0.8564	0.8471	0.6909
4022	0.63	NULL	0.5	0.6905	0.75	0.5	1	0.7143	1	0.7273
4023	0.6102	0.7126	0.6919	0.3594	0.7136	0.3842	0.5532	0.5637	0.6453	0.8091
4024	0.3514	1	NULL	NULL	NULL	0.9	0.9981	1	0.9	1
4026	0.0688	0.027	0.0588	0.0789	0.0357	0.1149	0.9972	0.0854	0.0643	0.0923
4029	0.1967	0.3446	0.3681	0.2593	0.0588	0.25	0.1057	0.1063	0.0309	0.2794
4035	0.3829	0.6967	0.5847	0.4884	0.2595	0.4384	0.1651	0.0641	0.3116	0.6071
4041	0.7846	0.88	0.6582	0.6154	0.8605	0.7024	0.9065	0.8765	0.6875	0.9745
4060	0.634	1	1	1	1	1	0.9995	1	1	1
4068	0.9779	1	1	1	1	1	0.9971	1	1	1
4075	0.9071	0.7985	0.9621	0.9659	0.9941	0.9634	1	0.9947	0.9931	0.8316
4083	0.9492	0.8239	1	0.9948	1	0.9731	1	1	1	0.8318
4089	0.9791	0.911	1	1	1	1	0.9995	0.9947	1	0.9356
4106	0.6276	0.8205	0.6284	0.7188	0.396	0.7241	0.5112	0.5876	0.4345	0.8317
4109	0.167	0.0649	0.2639	0.275	0.2246	0.1625	0.2363	0.2014	0.1286	0.1
4130	0.2819	0.4569	0.2643	0.2278	0.3963	0.2877	0.3809	0.3355	0.2857	0.325
4131	0.9961	1	1	1	1	1	1	1	1	1
4134	0.0795	0.0747	0.0632	0	0.1378	0.0532	0.1411	0.1276	0.1012	0.0802
4142	0.2634	0.1392	0.36	0.3063	0.375	0.3929	0.5215	0.3467	0.4052	0.0701
4146	0.5511	0.4709	0.5488	0.4624	0.6324	0.7312	0.6088	0.601	0.5882	0.4128
4150	0.0934	0.0671	0.1908	0.14	0.0313	0.1324	0.1123	0.1382	0.0235	0.1014
4151	0.107	0.0563	0.1957	0.129	0.0519	0.1218	1	0.0533	0.1103	0.1486
4154	0.4674	0.6667	0.5	0.5455	0.3333	0.4643	1	0.3125	0.5714	0.5556
4158	0.2053	0.25	0.0517	0.1198	0.2476	0.1579	0.3114	0.2892	0.1802	0.3091
4159	0.6946	0.6964	0.657	0.5753	0.6931	0.6782	0.7694	0.7448	0.8176	0.802
4162	0.2915	0.4138	0.1782	0.2809	0.2784	0.4043	0.2003	0.18	0.2151	0.3878
4167	0.1676	0.128	0.1667	0.2937	0.4196	0.2536	0.4388	0.4364	0.2222	0.0275
4169	0.9917	1	1	1	1	1	1	1	1	1
4176	0.5611	0.44	0.5833	0.4524	0.6818	0.58	0.9995	0.5455	0.55	0.6731
4179	1	1	1	1	1	1	1	1	1	1
4215	0.5112	0.6333	0.7927	0.5536	0.4727	0.38	0.9922	0.4015	0.5357	0.4789
4216	0.7228	1	1	1	1	1	1	1	1	1
4224	0.8472	1	1	1	1	1	1.0009	0.98	1	1
4228	0.6068	1	1	1	1	1	0.9935	1	1	1
4229	0.8513	0.7212	0.8889	0.8525	0.9315	0.8852	0.9528	0.8676	0.9196	0.7434
4230	0.9375	1	1	1	1	1	0.9922	1	1	1
4232	0.5878	1	1	1	1	1	0.9953	1	1	1
4233	0.8946	1	1	1	1	1	0.9991	1	1	1
4234	1	1	1	1	1	1	1	1	1	1
4235	0.7247	0.5833	0.75	0.4694	0.949	0.6688	0.9836	0.9167	0.9861	0.5741
4238	0.951	1	1	1	1	1	0.9972	1	1	1
4245	0.3998	0.2422	0.4194	0.3269	0.5726	0.3889	1	0.5714	0.5082	0.2119
4247	0.9275	1	1	1	1	1	1	1	1	1
4258	0.7636	1	1	1	1	1	0.9991	1	1	1
4259	0.6543	1	1	1	1	1	0.9981	1	1	1
4295	0.6798	1	1	1	1	1	1	1	1	1

4305	0.8206	0.875	0.6353	0.5158	0.99	0.6489	1	1	0.9375	0.9682
4310	0.2531	0.6032	0.1951	0.1813	0.1804	0.2247	0.115	0.12	0.1329	0.6
4312	0.1537	0.1084	0.1	0.1264	0.314	0.142	0.2524	0.264	0.0679	0.1359
4316	0.4358	0.8194	0.3688	0.5056	0.2526	0.4573	0.3199	0.2677	0.1548	0.85
4317	0.9547	0.9138	0.9353	0.9521	0.9845	0.9734	0.972	0.9588	0.9941	0.963
4321	1	1	1	1	1	1	1	1	1	1
4329	0.9661	1	1	1	1	1	0.9991	1	1	1
4332	0.1422	0.1753	0.1623	0.1442	0.169	0.1294	0.1084	0.0671	0.1098	0.25
4334	0.9707	1	1	1	1	1	0.9991	1	1	1
4336	0.9986	1	1	1	1	1	1	1	1	1
4340	0.2532	0.3333	0.4462	0.1067	0.4024	0.0538	0.3653	0.3514	0.4397	0.25
4343	0.0547	0.0183	0.1133	0.0976	0.0291	0.1037	0.0556	0.0438	0.0235	0.0423
4352	0.1174	0.1125	0.1111	0.1349	0.1279	0.0988	0.1067	0.119	0.1118	0.1646
4358	0.8485	1	1	1	1	1	1	1	1	1
4359	0.4109	0.4634	0.3861	0.3351	0.4783	0.2901	0.6139	0.5889	0.4026	0.4109
4368	0.4192	0.5882	0.3529	0.2765	0.6139	0.3311	0.5423	0.4236	0.4841	0.5987
4373	0.9888	1	1	1	1	1	1	1	1	1
4394	0.9908	0.9803	1	1	1	1	1	0.9946	0.9936	0.9891
4401	0.1873	0.2672	0.1635	0.3077	0.0294	0.1319	0.9971	0.1765	0.3545	0.2083
4402	0.9895	1	1	1	1	1	0.9981	1	1	1
4407	0.2158	0.2557	0.1221	0.0625	0.233	0.1632	0.3044	0.3627	0.2209	0.2818
4414	0.8603	0.8916	0.7176	0.7737	0.896	0.8353	0.9668	0.9521	0.8951	0.9231
4415	0.9971	1	1	1	1	1	0.9991	1	1	1
4416	0.9971	1	1	1	1	1	1	1	1	1
4422	0.6947	0.5855	0.6875	0.4624	0.9022	0.6379	0.958	0.9136	0.88	0.5784
4423	0.2844	0.2892	0.3913	0.4762	0.2041	0.3875	0.3263	0.2363	0.2089	0.2143
4428	0.2066	0.1667	0.2532	0.2647	0.1889	0.1705	0.1991	0.1489	0.3269	0.195
4429	0.9482	1	1	1	1	1	1	1	1	1
4430	0.9986	1	1	1	1	1	0.9991	1	1	1
4434	0.2065	0.3151	0.34	0.3333	0.0824	0.3	0.1712	0.125	0.0536	0.2639
4435	0.9851	1	1	1	1	1	0.9981	0.9947	1	1
4436	0.617	0.5556	0.679	0.3021	0.8535	0.3901	0.9099	0.8523	0.7963	0.5841
4437	0.1869	0.3	0.1667	0.022	0.3072	0.0787	0.3046	0.2569	0.2357	0.3397
4442	0.2521	0.3052	0.0855	0.2069	0.3397	0.2346	0.3613	0.4022	0.2857	0.2908
4443	0.2282	0.2113	0.2361	0.25	0.2763	0.3047	0.4393	0.2302	0.2016	0.25
4447	0.8957	1	1	1	1	1	1	1	1	1
4448	0.855	1	1	1	1	1	0.9991	1	1	1
4453	0.3355	0.3673	0.4107	0.4912	0.1324	0.3125	1	0.3017	0.2308	0.4667
4456	0.8784	0.871	0.94	0.9247	0.9527	0.9306	0.9986	1	0.9344	0.8056
4459	0.988	0.9589	1	1	1	0.9945	1	1	1	0.9894
4465	1	1	1	1	1	1	0.9843	1	1	1
4468	0.996	1	1	1	1	1	1	1	1	1
4470	0.7056	0.6125	0.7	0.8605	0.7647	0.725	1	0.7429	0.6351	0.7955
4473	0.3829	0.241	0.3056	0.3547	0.6196	0.3375	0.666	0.6075	0.4571	0.265
4484	0.8136	0.7239	0.8881	0.9345	0.8782	0.8169	0.8131	0.759	0.8651	0.7386
4487	0.5768	0.6776	0.6341	0.383	0.586	0.5765	0.6477	0.5707	0.628	0.6735
4488	0.0485	0.0188	0.0779	0.0893	0.0875	0.0349	0.0271	0.0449	0.0179	0.0368
4489	0.4776	0.22	0.6538	0.6563	0.5104	0.6	0.9902	0.5116	0.6429	0.2209
4498	0.9894	1	1	1	1	1	0.9942	1	1	1
4502	0.9226	0.8782	0.8014	0.967	0.9894	0.962	0.9939	0.988	0.9675	0.8763
4506	0.9934	1	1	1	1	1	1	1	1	1
4509	0.9948	1	1	1	1	0.96	0.9981	1	1	0.9857
4512	0.3805	0.1875	0.4231	0.3696	0.4615	0.373	1	0.6078	0.6224	0.1698
4514	0.803	0.9206	0.8731	0.7222	0.821	0.7095	0.7647	0.7278	0.8167	0.9765
4521	0.2212	0.1313	0.3491	0.1071	0.5	0.1831	0.4178	0.3929	0.4286	0.1296
4525	1	1	1	1	1	1	0.9933	1	1	1
4530	0.7304	1	1	1	1	1	0.9991	1	1	1
4531	0.3059	0.3	0.4032	0.3548	0.3929	0.2966	0.4331	0.3403	0.5	0.2113
4536	0.8381	1	1	1	1	1	0.9991	1	1	1
4578	0.0027	0	0	0.006	0	0.0053	0.0065	0.0101	0	0
4593	0.3575	0.1953	0.3241	0.3409	0.6286	0.375	0.73	0.4783	0.5776	0.1148
4601	0.8571	1	1	1	1	1	1	1	1	1
4603	0.8894	0.7785	0.878	0.9222	0.9694	0.9375	0.8662	0.9	1	0.7576
4604	0.7824	0.7091	0.902	0.7639	0.9058	0.803	0.8804	0.838	0.9355	0.6027
4605	0.0278	0.0122	0.026	0	0.0385	0.0281	0.0215	0.0461	0.0179	0.0455
4610	0.1533	0.0685	0.0833	0.3205	0.1575	0.2901	0.2338	0.1462	0.1753	0.0606

4612	1	1	1	1	1	1	0.9995	1	1	1
4619	0.9854	1	1	1	1	1	1	1	1	1
4622	0.8342	1	1	1	1	1	0.9981	1	1	1
4623	0.6985	1	1	1	1	1	1	1	1	1
4630	0.9856	1	0.9906	1	0.9861	1	1	1	1	0.9932
4634	0.702	0.9648	0.7639	0.7697	0.4783	0.7039	0.5658	0.5158	0.58	0.9659
4636	0.6744	1	1	1	1	1	0.9991	1	1	1
4637	0.9921	1	1	1	1	1	1	1	1	1
4645	0.2358	0.2927	0.25	0.099	0.2549	0.1333	0.2743	0.1947	0.2901	0.3857
4649	0.8975	1	1	1	1	1	1	1	1	1
4652	0.7315	1	1	1	1	1	0.9991	1	1	1
4654	0.2488	0.0208	0.4	0.2241	0.4894	0.1905	0.9961	0.3974	0.4886	0.0439
4660	0.9878	1	1	1	1	1	1	1	1	1
4661	0.7651	1	1	1	1	1	1	1	1	1
4662	0.8309	0.5357	0.9938	0.9323	0.965	0.9516	0.9533	0.9255	0.9176	0.4861
4664	0.5355	0.1919	0.7892	0.8723	0.5882	0.7989	0.5761	0.5464	0.6346	0.0364
4666	0.7289	1	1	1	1	1	1	1	1	1
4670	0.9987	1	1	1	1	1	1	1	1	1
4671	0.2249	0.3243	0.3526	0.3333	0.1348	0.2716	0.1895	0.1173	0.1563	0.2179
4674	0.8312	0.9205	0.8161	0.4583	0.9466	0.5474	0.9692	0.9608	0.9942	0.9864
4675	0.9711	1	1	1	1	1	1	1	1	1
4677	0.505	0.7576	0.538	0.2857	0.5426	0.3869	0.6745	0.5655	0.4675	0.8896
4678	0.6906	0.8429	0.7639	0.337	0.8011	0.4725	0.7673	0.7444	0.8169	0.8978
4683	0.9681	1	1	1	1	1	0.9991	1	1	1
4684	0.5454	0.5291	0.4765	0.4479	0.6429	0.5914	0.6008	0.5928	0.6235	0.4811
4690	0.1741	0.125	0.1232	0.3615	0.1386	0.1338	0.9951	0.2381	0.0942	0.2688
4695	0.9985	1	1	1	1	1	1	1	1	1
4696	0.8314	0.8523	0.8654	0.9057	0.8362	0.8617	1	0.8509	0.8723	0.9032
4712	0.1069	0.125	0.0988	0.0365	0.1505	0.0158	0.1489	0.1683	0.064	0.1773
4714	0.9986	1	1	1	1	1	1	1	1	1
4717	0.0625	0.0556	0.0522	0.0724	0.0333	0.0676	0.9981	0.0407	0.1508	0.0521
4733	0.7285	0.7319	0.7899	0.8241	0.72	0.8194	0.7953	0.6645	0.7267	0.7581
4737	0.7079	1	1	1	1	1	1	1	1	1
4738	0.9817	1	1	1	1	1	0.9981	1	1	1
4742	0.972	0.9329	1	1	1	0.9944	1	1	1	0.919
4749	0.3333	0.4907	0.5111	0.6596	0.1618	0.3534	0.3994	0.2132	0.2544	0.3051
4751	0.755	0.9744	0.7244	0.9167	0.6071	0.9268	0.6018	0.5515	0.5128	0.989
4770	0.7444	1	1	1	1	1	1	1	1	1
4771	0.9185	1	1	1	1	1	0.9981	1	1	1
4783	0.1337	0.0636	0.0976	0.1667	0.375	0.2264	0.357	0.3696	0.2326	0.0172
4788	0.4045	0.2765	0.3095	0.1968	0.6327	0.3908	0.6402	0.6022	0.5875	0.3029
4795	0.6176	0.6948	0.7563	0.6559	0.5372	0.5714	0.6753	0.6354	0.45	0.7602
4802	0.5061	0.5976	0.4345	0.484	0.365	0.6294	0.45	0.4643	0.3631	0.7103
4805	0.0063	0.0122	0.0063	0	0.0156	0	0.9895	0	0.0065	0.0097
4808	0.5775	0.4602	0.5115	0.8021	0.5485	0.7609	0.5224	0.495	0.5465	0.5
4810	0.4455	0.4034	0.4186	0.1875	0.6287	0.3226	0.627	0.6139	0.6131	0.3909
4814	0.3495	0.3	0.35	0.5	0.375	0.5556	0.9961	0.5	0.5625	0.2273
4826	0.4682	0.142	0.54	0.5269	0.8315	0.5114	0.873	0.8086	0.7464	0.0682
4842	0.4706	0.3409	0.2989	0.5938	0.6019	0.4734	0.6839	0.76	0.4529	0.2364
4843	0.4682	0.5446	0.6574	0.4054	0.5809	0.4552	0.7725	0.675	0.6739	0.6111
4845	0.5608	0.3294	0.6418	0.6845	0.7738	0.5602	0.8289	0.6875	0.8649	0.2619
4846	0.3949	0.4583	0.3014	0.28	0.3571	0.3194	0.5461	0.4839	0.56	0.5244
4924	0.1874	0.0806	0.1724	0.2845	0.1786	0.1319	0.9962	0.2095	0.1574	0.277
4926	0.4187	0.408	0.4713	0.4271	0.4604	0.4368	0.4099	0.4208	0.5	0.2591
4927	0.1298	0.3355	0.0759	0.0926	0.1	0.1337	0.0921	0.0625	0.0443	0.2254
4928	0.3463	0.2797	0.3868	0.3488	0.5	0.3305	1	0.407	0.3542	0.3365
4929	0.6233	1	1	1	1	1	1	1	1	1
4935	0.9851	1	1	1	1	1	0.9991	1	1	1
4936	0.2444	0.4651	0.1322	0.0781	0.2524	0.1702	0.2866	0.2696	0.1588	0.4128
4938	0.9938	1	1	1	1	1	1	1	1	0.995
4940	0.8523	0.8353	0.741	0.7211	0.95	0.7935	0.9407	0.9439	0.9695	0.8853
4946	0.1182	0.0733	0.1053	0.1813	0.1105	0.1346	0.1725	0.1067	0.1338	0.1563
4948	0.9948	1	1	1	1	1	1	1	1	1
4949	0.6974	1	1	1	1	1	0.9991	1	1	1
4950	0.9318	1	1	1	1	1	1	1	1	1
4953	0.2796	0.1067	0.3828	0.4825	0.3016	0.3712	0.5447	0.2891	0.3509	0.1059

4958	0.5845	0.9773	0.9853	1	1	1	1	1	1	1
4959	0.9289	1	1	1	1	1	1	1	1	1
4966	0.5569	0.5602	0.4226	0.3854	0.6786	0.5341	0.6998	0.6895	0.5833	0.6193
4972	0.4049	0.431	0.3908	0.2	0.4747	0.3211	0.5341	0.495	0.4709	0.4545
4977	0.4706	0.5	0.5	0.375	0.5	0.5417	0.9942	0.6	0.3333	0.5882
4978	0.0831	0	0.0287	0.0158	0.1436	0.1374	0.178	0.2337	0.1412	0
4987	0.7011	1	1	1	1	1	0.9971	1	1	1
4991	0.9751	1	1	1	1	1	0.9981	1	1	1
4993	0.8623	1	1	1	1	1	1	1	1	1
4995	0.2588	0.1667	0.6875	0.3333	0	0.2105	0.999	0.5	0	0.24
4996	0.9782	1	1	1	1	1	1	1	1	1
5000	0.9961	1	1	1	1	1	1	1	1	1
5006	0.0384	0.0061	0.0298	0.0816	0.0563	0.0333	0.0005	0.0432	0.0235	0.0571
5008	0.9983	1	1	1	1	1	1	1	1	1
5011	0.2564	0.5978	0.2143	0.0649	0.4221	0.0893	0.3016	0.3507	0.425	0.65
5017	0.0696	0.1231	0.1515	0.0462	0.0422	0.0541	1	0.0536	0.0614	0.0471
5026	0.055	0.0484	0.1273	0.069	0.0385	0.0063	0.9972	0	0.0887	0.0862
5038	0.748	0.6548	0.8647	0.8	0.7772	0.8523	0.6508	0.6598	0.8025	0.6636

Supplementary Table 6. Results of validation for 10 TMMINSs by short read mapping and PCR.

Chr	Identity	INS length (bp)	Results	
			JPN00001	NA12878
1	TMMINS102	1,165	+ (+/+)*	+ (+/+)
1	TMMINS371	4,287	+ (+/+)	+ (+/+)
2	TMMINS2604	8,435	+ (+/+)	+ (+/+)
6	TMMINS4131	2,893	+ (+/+)	+ (+/+)
7	TMMINS4305	2,478	+ (+/+)	+ (-/-)**
7	TMMINS4468	1,472	+ (+/+)	+ (+/+)
8	TMMINS4674	4,338	+ (+/+)	+ (+/+)
10	TMMINS670	2,009	+ (+/+)	+ (+/+)
12	TMMINS1152	7,260	+ (+/+)	+ (+/+)
12	TMMINS1200	985	+ (+/+)	+ (+/+)

Plus and minus signs inside the parentheses indicate the result of short read mapping (left) and PCR (right), respectively. Plus means that the existence of the TMMINS was confirmed while minus means not confirmed. The plus (+) outside the parentheses denotes that the result of short read mapping and PCR are consistent.

* Both results were positive and they were consistent.

** Both results were negative and they were consistent.

Supplementary Table 7. Features of 871 biallelic TMMINSs for 1KJPN, JPT and Korean (AK1).

TMMINS	Chr	Start	End	i1000g_af	1KJPN	JPT	Korean (AK1)
13	chr1	1477854	1477854	0.1706	1	0.2237	1
18	chr1	1993704	1993704	0.9981	1	1	1
57	chr1	9624025	9624025	0.9974	1	1	0
62	chr1	10971095	10971095	0.6755	1	1	0
63	chr1	11041749	11041749	0.844	1	1	0
64	chr1	11373885	11373885	0.4588	1	1	1
66	chr1	11770159	11770159	0.1419	0.0861	0.0887	0
67	chr1	12565679	12565717	0.2567	0.265	0.2969	0
71	chr1	13470170	13470170	0.1039	0.9991	0.1029	0
74	chr1	14492689	14492711	0.6365	1	1	0
81	chr1	17289330	17289330	0.9599	1	1	0
84	chr1	18049642	18049642	0.6639	1	1	0
87	chr1	19932945	19932945	0.6904	1	1	0
88	chr1	20382469	20382469	0.7004	0.9972	1	1
89	chr1	20463556	20463556	0.8438	1	1	0
93	chr1	22950454	22950454	0.25	1	0.2426	1
94	chr1	23277312	23277312	0.9987	1	1	0
100	chr1	25529503	25529503	0.5288	1	1	1
101	chr1	27030606	27030606	0.5098	1	1	0
102	chr1	27086500	27086500	0.9803	0.9981	1	1
104	chr1	27878472	27878472	0.9809	1	1	0
115	chr1	33186800	33186817	0.9847	1	1	0
125	chr1	37446187	37446187	0.79	0.9961	1	1
128	chr1	38945870	38945870	0.9115	0.9495	0.9427	0
131	chr1	40050405	40050405	0.0172	0.9933	0	1
140	chr1	45710639	45710846	0.8947	0.8231	0.8505	0
149	chr1	50369365	50369365	0.7767	1	1	0
156	chr1	54510666	54510666	0.0349	0.0005	0.0517	0
157	chr1	55003057	55003057	0.7685	1	1	0
163	chr1	58729593	58729593	0.6328	1	0.6429	1
164	chr1	58739645	58739645	0.0941	0.0815	0.0795	0
169	chr1	61393285	61393285	0.8196	0.9542	0.9752	0
172	chr1	64492395	64492395	0.1926	1	0.2295	0
187	chr1	77472789	77472789	0.258	0.3927	0.3918	1
192	chr1	80079572	80079572	0.4272	0.4316	0.4023	0
193	chr1	83123083	83123083	0.0698	0.2241	0.0875	0
200	chr1	87046939	87046939	0.9241	1	0.9895	0
201	chr1	88347942	88347942	0.1042	0.207	0.142	0
203	chr1	88457679	88457679	0.9896	0.9981	1	1
211	chr1	93876121	93876138	0.9853	1	0.995	0
220	chr1	103938233	103938233	0.9505	1	1	1
221	chr1	104389532	104389532	0.209	0.305	0.338	0
224	chr1	105571334	105571334	0.7766	0.7339	0.6923	0
227	chr1	105963338	105963338	0.3163	0.9991	0.125	1
229	chr1	108675987	108675987	0.3963	1	0.75	1
231	chr1	111259975	111259975	0.3895	0.3942	0.3438	1
236	chr1	112999691	112999691	0.6983	0.6989	0.7071	0
279	chr1	156004627	156004627	1	1	1	0
281	chr1	156735557	156735557	0.5857	1	1	0
285	chr1	161035894	161035894	0.9704	0.9991	1	1
298	chr1	167205990	167205990	0.886	0.9986	0.995	1
300	chr1	168395498	168395498	0.951	0.9977	0.9949	1

315	chr1	179681968	179681968	0.9127	1	1	1
319	chr1	182138477	182138477	0.9789	1	1	1
324	chr1	185187063	185187063	0.044	0.1001	0.0561	0
330	chr1	191119509	191119509	0.4095	1	0.4535	1
339	chr1	198397492	198397492	0.1452	0.9962	0.119	0
348	chr1	204195224	204195224	0.7358	0.9991	1	1
357	chr1	213791000	213791000	0.25	0.1621	0.234	0
361	chr1	218988269	218988269	0.5923	1	0.875	1
363	chr1	219074032	219074032	0.9786	0.9981	1	1
369	chr1	221654479	221654479	0.3	0.289	0.1818	0
371	chr1	223081189	223081189	0.7417	0.9991	1	1
372	chr1	223347734	223347745	0.3231	0.3053	0.32	0
375	chr1	225372931	225372931	0.0671	0.0393	0.0974	0
377	chr1	225964538	225964538	0.0909	1	0.0798	1
378	chr1	226227771	226227771	0.8191	0.9991	0.9844	1
390	chr1	233934818	233934818	0.2333	0.2695	0.2202	0
395	chr1	235410023	235410023	0.7252	0.5887	0.5833	0
403	chr1	237743989	237743989	0.0931	0.0005	0.1429	0
418	chr1	246009982	246009982	0.959	1	1	1
426	chr1	247222870	247222873	0.1142	0.1928	0.2089	0
442	chr10	1483710	1483710	0.2723	0.2505	0.1576	1
450	chr10	2562647	2562647	0.2716	0.5758	0.3061	0
459	chr10	5916704	5916704	0.4779	1	0.7262	0
466	chr10	9469018	9469018	0.2806	0.998	0.2404	1
473	chr10	12825144	12825144	1	1	1	1
482	chr10	18566017	18566017	0.1937	1	0.1667	0
483	chr10	19238068	19238068	0.9394	1	1	1
488	chr10	23508108	23508108	0.3002	0.1276	0.1371	0
489	chr10	24557950	24557950	0.2626	1	0.3627	1
490	chr10	25454270	25454293	0.7791	0.9995	0.9268	0
494	chr10	28043991	28043991	0.4345	0.493	0.4896	0
508	chr10	35610828	35610836	0.6344	0.5645	0.601	1
518	chr10	39216800	39216800	0.9498	0.9371	0.9408	0
519	chr10	39254773	39254793	1	0.9376	1	0
572	chr10	44825673	44825673	0.1759	0.2697	0.2917	0
575	chr10	46876556	46876556	0.2224	0.3792	0.2805	0
576	chr10	47023844	47023844	0.7475	1	1	0
581	chr10	49742433	49742433	0.9742	1	1	1
585	chr10	54210218	54210218	0.1102	0.0192	0.1585	0
588	chr10	56576045	56576045	0.4029	0.3025	0.299	0
592	chr10	60365100	60365100	0.2702	0.3701	0.2692	0
596	chr10	63783076	63783076	0.9333	1	0.9889	1
605	chr10	69784614	69784618	0.3001	0.352	0.3265	0
616	chr10	74819359	74819359	0.988	1	1	0
621	chr10	81212445	81212445	0.9895	1	1	1
623	chr10	83219155	83219155	0.498	0.4477	0.4902	0
632	chr10	90455315	90455315	0.2361	0.3767	0.3235	0
644	chr10	98735245	98735250	0.9855	1	1	1
646	chr10	99580569	99580569	0.9659	1	1	0
651	chr10	101288328	101288330	0.6168	0.5743	0.5417	1
652	chr10	101728733	101728733	1	1	1	0
658	chr10	103992654	103992654	0.4057	0.9991	0.4107	1
666	chr10	116390787	116390787	0.888	0.999	1	0
669	chr10	119009618	119009618	0.1659	0.1711	0.1667	0
670	chr10	119547323	119547332	0.9987	1	1	1
700	chr10	131512438	131512438	0.5804	0.9953	1	1

710	chr10	132514641	132514641	0.6849	0.9991	1	1
713	chr10	133004613	133004613	0.651	0.6131	0.6607	0
719	chr11	356450	356450	0.93	1	1	1
721	chr11	428014	428014	1	1	1	1
728	chr11	2187925	2187925	0.9576	1	1	1
738	chr11	5114183	5114183	0.3445	0.418	0.4458	1
739	chr11	5549455	5549455	1	1	1	0
744	chr11	7815556	7815556	0.6558	0.6373	0.5931	0
755	chr11	13016508	13016508	0.8996	0.9991	1	1
758	chr11	14841729	14841729	0.6486	0.6415	0.6302	0
763	chr11	18378369	18378369	0.9947	1	1	0
765	chr11	19618916	19618916	0.9763	1	1	1
770	chr11	23016591	23016591	0.9944	0.9972	1	1
771	chr11	23336971	23336971	0.2848	0.243	0.1623	0
772	chr11	23347666	23347666	0.9428	0.9402	0.9316	1
775	chr11	25044085	25044085	0.2942	0.999	0.2045	1
778	chr11	26580099	26580102	0.1377	0.2855	0.2386	0
786	chr11	33403303	33403303	0.7112	0.9981	1	0
789	chr11	34375839	34375839	0.2135	0.1806	0.2267	0
791	chr11	36055104	36055113	0.7337	1	1	0
795	chr11	37684745	37684745	0.0774	0.9962	0.1316	0
808	chr11	44942023	44942023	0.9847	0.9932	1	1
810	chr11	47371383	47371383	0.9932	1	1	1
811	chr11	47557575	47557575	0.0238	1	0.0143	1
812	chr11	47638553	47638553	0.9881	1	0.995	0
814	chr11	47893238	47893238	0.99	1	0.995	0
816	chr11	48727163	48727163	0.9985	0.9995	1	0
821	chr11	48970399	48970399	0.9814	1	1	1
823	chr11	49695838	49695838	0.183	0.2801	0.1148	0
830	chr11	54527189	54527189	0.1527	0.1474	0.1173	0
831	chr11	54926201	54926201	0.9671	0.9991	0.9889	0
841	chr11	61377742	61377742	0.4071	0.9991	0.5698	0
846	chr11	63559621	63559621	0.2774	0.5621	0.5842	1
848	chr11	63931437	63931437	1	0.9967	1	1
865	chr11	67837701	67837701	0.4561	0.7962	0.7037	0
869	chr11	68443505	68443505	0.0894	0.0458	0.0347	0
876	chr11	69292049	69292049	0.5985	1	1	0
880	chr11	70803384	70803384	0.9712	1	1	0
886	chr11	75763446	75763446	0.9935	0.9991	1	0
887	chr11	76619322	76619322	0.5016	1	0.5484	0
893	chr11	81761979	81761979	0.1781	0.4009	0.25	1
897	chr11	86272201	86272201	0.9915	1	1	0
902	chr11	88646606	88646606	0.0436	0.0492	0.0617	0
909	chr11	91044224	91044224	0.3835	1	0.8333	1
910	chr11	91147223	91147223	0.3293	0.9985	0.3125	0
913	chr11	92329739	92329739	0.9987	1	1	1
916	chr11	96089966	96089968	0.3677	0.2414	0.1804	0
918	chr11	96794927	96794927	0.4232	0.4388	0.3977	1
919	chr11	97317908	97317908	0.1245	0.9972	0.1386	1
923	chr11	101925712	101925712	1	1	1	1
931	chr11	106299754	106299754	0.1034	0.9991	0.1455	0
943	chr11	118421755	118421755	0.9876	1	1	0
944	chr11	118683016	118683016	0.3467	0.5048	0.4286	0
946	chr11	118807470	118807470	0.9893	1	1	1
948	chr11	119253539	119253539	0.8422	1	1	1
956	chr11	121260819	121260819	0.3025	0.3669	0.3485	0

966	chr11	126632699	126632699	0.8695	1	1	0
971	chr11	129127585	129127585	0.1805	0.1638	0.1831	0
978	chr11	132125738	132125738	0.7679	1	1	1
979	chr11	133027804	133027804	0.7146	1	1	0
986	chr11	134435748	134435753	0.9959	0.9676	1	1
1002	chr12	1620991	1620991	0.9986	1	1	0
1012	chr12	5481487	5481487	0.6304	0.9981	1	1
1013	chr12	5617183	5617183	0.3583	0.9923	0.2833	0
1015	chr12	6305635	6305635	0.9056	0.8929	0.8571	0
1016	chr12	6362962	6362962	0.9826	1	1	0
1017	chr12	6403579	6403579	0.9986	0.9991	1	0
1019	chr12	6950074	6950074	0.9787	1	1	0
1029	chr12	9718566	9718566	0.1608	0.3121	0.2244	0
1031	chr12	11189300	11189300	0.6667	0.7611	0.65	1
1032	chr12	12653569	12653569	0.9483	0.979	0.9853	0
1039	chr12	17674882	17674882	0.2901	0.4411	0.4341	1
1049	chr12	25106528	25106559	0.2374	0.8531	0.3958	0
1053	chr12	26775933	26775933	0.0949	0.1648	0.0895	0
1055	chr12	26805727	26805727	0.4677	0.6827	0.5786	0
1085	chr12	37973123	37973123	0.9288	0.9767	0.9219	0
1086	chr12	38854389	38854391	0.2431	0.3175	0.3137	0
1094	chr12	43524179	43524179	0.7784	0.9588	0.9628	1
1095	chr12	45025772	45025772	0.2336	1	0.2946	0
1102	chr12	49611831	49611831	0.6867	1	0.9902	0
1103	chr12	51498895	51498895	0.9566	1	1	0
1106	chr12	53290716	53290716	0.9953	1	1	1
1108	chr12	54209274	54209274	0.9913	1	1	0
1110	chr12	55334399	55334399	0.4411	0.3413	0.3086	0
1113	chr12	57191973	57191973	0.9692	0.9991	1	0
1116	chr12	58726315	58726315	0.2348	0.3982	0.305	0
1117	chr12	59832056	59832056	0.7223	1	1	1
1119	chr12	61061267	61061267	0.3931	0.4127	0.4406	1
1123	chr12	63305014	63305014	0.2971	0.2638	0.2941	0
1124	chr12	64783848	64783848	0.9848	1	1	1
1130	chr12	71139972	71139974	0.2789	0.2617	0.1739	0
1139	chr12	77990098	77990108	0.2858	0.2152	0.2101	0
1143	chr12	78777421	78777421	0.2209	0.9991	0.2833	1
1152	chr12	86259792	86259795	0.9444	1	0.995	1
1154	chr12	87477046	87477046	0.1346	0.9995	0.1667	1
1156	chr12	88343484	88343484	0.1299	0.9971	0.1039	0
1160	chr12	94868534	94868534	0.2673	0.5005	0.4624	1
1161	chr12	95195689	95195689	0.1158	0.999	0.1019	1
1163	chr12	96119943	96119943	0.1857	0.3816	0.2465	0
1167	chr12	101747636	101747643	0.7016	0.8573	0.8554	1
1175	chr12	107809487	107809487	0.2459	0.1312	0.0739	0
1176	chr12	107914290	107914290	0.7182	0.605	0.5882	1
1182	chr12	110178828	110178828	0.9735	1	1	0
1191	chr12	115293288	115293288	0.1658	0.2899	0.3108	0
1196	chr12	117152017	117152017	0.0265	0.061	0.0615	0
1197	chr12	118295184	118295184	0.0946	0.0574	0.0686	0
1198	chr12	120621961	120621961	0.1245	0.2716	0.1647	0
1199	chr12	120746038	120746038	0.1227	0.2162	0.2451	0
1200	chr12	121052772	121052772	0.9914	0.9991	1	1
1203	chr12	122902424	122902424	0.996	0.9991	1	0
1211	chr12	124999904	124999904	0.9894	0.9995	1	0
1214	chr12	125316618	125316618	0.777	0.8431	0.7292	0

1218	chr12	127153620	127153657	0.7901	0.9972	0.9951	0
1219	chr12	127229747	127229766	0.1211	1	0.1742	0
1226	chr12	130055052	130055052	0.9866	1	1	0
1230	chr12	130660325	130660325	0.4211	0.5222	0.4489	1
1245	chr13	18653028	18653028	0.9986	1	1	1
1260	chr13	22679414	22679414	0.9969	0.9991	1	1
1261	chr13	23006720	23006721	0.5959	0.7031	0.74	1
1267	chr13	24937876	24937876	0.2381	0.2979	0.3671	0
1274	chr13	26972770	26972770	0.7426	0.9958	1	1
1279	chr13	28584809	28584809	0.9885	0.9991	1	1
1310	chr13	48912442	48912444	0.9961	1	1	1
1314	chr13	50963171	50963171	0.2215	0.9937	0.1792	0
1318	chr13	52893262	52893262	0.9968	1	1	1
1334	chr13	64251734	64251734	0.4377	0.6704	0.5946	1
1336	chr13	67329322	67329322	0.4774	1	0.6731	1
1343	chr13	72653253	72653253	0.7204	0.989	0.8558	1
1351	chr13	78077552	78077552	0.365	0.441	0.4167	0
1354	chr13	78697466	78697466	1	0.9991	1	0
1359	chr13	84345473	84345484	0.985	1	1	1
1365	chr13	85766014	85766017	0.2948	0.5638	0.4468	0
1368	chr13	86426898	86426898	0.0667	0.0098	0.0733	0
1373	chr13	88458706	88458706	0.1619	0.5761	0.1613	0
1377	chr13	89467768	89467768	0.3854	0.6527	0.5934	1
1378	chr13	90090935	90090935	0.0898	0.0813	0.0735	0
1387	chr13	93453566	93453566	0.2348	0.3047	0.3017	0
1390	chr13	95195647	95195647	0.3883	0.9981	0.4348	0
1394	chr13	97852874	97852874	0.2102	0.9971	0.1286	1
1406	chr13	102694504	102694507	0.9112	0.923	0.9314	1
1408	chr13	103643888	103643888	1	0.9991	1	0
1409	chr13	104244971	104244992	0.2031	1	0.5	0
1410	chr13	104564770	104564770	0.9966	1	1	0
1413	chr13	105644785	105644785	0.9675	0.9903	1	1
1417	chr13	108690378	108690378	0.5482	0.6106	0.549	1
1419	chr13	109048964	109048964	0.2477	1	0.4273	0
1423	chr13	110043600	110043600	0.4435	0.999	1	1
1443	chr13	112972801	112972801	0.8462	0.9991	1	1
1482	chr14	22944942	22944956	0.5195	0.7991	0.8177	0
1487	chr14	25272708	25272708	0.4434	0.5336	0.4646	1
1492	chr14	34374260	34374260	0.0952	0.2284	0.1538	1
1497	chr14	36173595	36173595	0.0829	0.0654	0.0723	0
1503	chr14	40110481	40110481	0.2211	0.2642	0.2955	0
1504	chr14	40123224	40123224	0.2564	0.2688	0.2831	1
1506	chr14	41371069	41371069	0.1244	0.3341	0.3115	0
1512	chr14	44417492	44417493	0.6276	0.5586	0.4767	1
1516	chr14	49761132	49761132	0.8892	0.9921	0.9802	1
1528	chr14	56999617	56999620	0.3845	0.375	0.3191	1
1530	chr14	57945429	57945448	0.2873	0.4519	0.4059	0
1536	chr14	63802351	63802351	0.5456	0.4472	0.4608	0
1537	chr14	64822374	64822374	1	1	1	1
1539	chr14	65796449	65796449	0.072	0.0609	0.0974	0
1540	chr14	66616294	66616294	0.466	0.4454	0.465	1
1543	chr14	69028324	69028324	0.6055	0.887	0.905	0
1547	chr14	73332740	73332742	0.6395	0.7972	0.7784	1
1549	chr14	74462088	74462088	0.9861	1	1	0
1556	chr14	80488215	80488215	0.681	0.9276	0.9167	1
1558	chr14	81649622	81649721	0.5667	0.4788	0.5202	0

1566	chr14	88684236	88684236	0.986	1	1	1
1594	chr14	105096071	105096071	0.9579	1	0.9923	1
1626	chr15	24626703	24626713	0.7959	0.9117	0.915	0
1638	chr15	33739651	33739651	0.202	0.2402	0.3049	0
1639	chr15	33894822	33894822	1	0.9849	1	0
1640	chr15	34376474	34376474	0.8763	0.9995	1	0
1642	chr15	36132570	36132570	0.5335	1	0.7073	1
1650	chr15	42712111	42712111	0.0949	1	0.0508	1
1659	chr15	47470416	47470416	0.0526	0.0215	0.0455	0
1664	chr15	53873744	53873744	0.9656	1	0.9797	1
1668	chr15	56541739	56541739	0.0091	1	0	0
1673	chr15	61348448	61348448	0.08	0.9991	0.0887	1
1675	chr15	63082400	63082400	0.6135	0.966	0.7581	1
1676	chr15	66212004	66212004	0.9986	1	1	1
1680	chr15	68395672	68395672	0.9182	1	1	1
1686	chr15	70861418	70861418	0.2435	0.2676	0.2692	0
1704	chr15	78993301	78993301	0.215	0.1904	0.1023	0
1706	chr15	84398524	84398524	0.8804	0.9995	0.9831	1
1716	chr15	90551822	90551822	0.5624	1	0.6585	1
1718	chr15	90871542	90871542	1	1	1	1
1737	chr16	182788	182788	0.9597	1	1	1
1738	chr16	239293	239293	0.9946	1	1	0
1744	chr16	760468	760468	0.6315	1	0.6667	1
1756	chr16	2863523	2863523	0.9869	1	1	0
1766	chr16	7147060	7147060	0.8904	1	0.9539	0
1769	chr16	7875178	7875178	0.2781	1	0.189	1
1772	chr16	9140607	9140607	0.6237	0.7374	0.6854	1
1774	chr16	10040009	10040009	0.7763	0.9972	1	1
1782	chr16	17470922	17470922	0.6321	0.9962	0.6	1
1785	chr16	19321671	19321671	0.8233	0.7193	0.712	1
1786	chr16	20162142	20162142	0.1133	0.2299	0.1563	0
1789	chr16	23776031	23776031	0.3476	0.2479	0.2277	0
1792	chr16	24872998	24872998	0.9893	0.9986	1	0
1793	chr16	26172099	26172106	0.5882	0.5434	0.5644	1
1797	chr16	27941650	27941650	0.9741	1	1	0
1800	chr16	30752247	30752247	0.8389	0.9952	1	0
1835	chr16	69728986	69728992	0.3025	0.2898	0.2771	0
1838	chr16	70087896	70087939	0.1686	0.1289	0.1868	0
1847	chr16	79770131	79770131	0.3852	0.9981	0.5364	1
1848	chr16	80183527	80183527	0.2444	0.3987	0.3425	1
1850	chr16	80763272	80763272	0.9985	0.9981	1	1
1860	chr16	86013934	86013934	0.6852	1	1	1
1863	chr16	86986874	86986874	0.9809	1	1	1
1865	chr16	87502299	87502299	0.8273	0.9981	1	1
1898	chr17	1219664	1219664	0.9967	0.9981	1	1
1900	chr17	1276786	1276786	0.9516	0.9962	1	1
1904	chr17	2719770	2719770	0.9946	1	1	1
1907	chr17	3899261	3899261	0.9404	1	1	0
1908	chr17	4384079	4384079	0.4985	0.5266	0.4773	1
1909	chr17	4790733	4790733	0.989	0.9981	1	1
1918	chr17	7302557	7302557	0.9987	1	1	0
1920	chr17	8166153	8166153	1	1	1	1
1921	chr17	8409775	8409775	0.3793	0.3763	0.3317	0
1923	chr17	9414088	9414088	0.1038	0.9971	0.0789	1
1926	chr17	9901444	9901448	0.0782	0.0672	0.0343	0
1936	chr17	14624337	14624337	0.2705	0.3028	0.3158	0

1942	chr17	19910327	19910327	0.0422	0.0935	0.0957	0
1951	chr17	26683715	26683715	0.9951	0.9957	1	0
1953	chr17	26821849	26821849	0.5585	0.9991	0.9565	0
1954	chr17	26848282	26848282	0.9955	1	1	0
1955	chr17	26857717	26857717	0.9091	0.9981	1	0
1963	chr17	33357030	33357030	1	1	1	1
1966	chr17	34346395	34346395	0.9457	0.9981	1	1
1972	chr17	40523190	40523190	0.9797	0.9991	1	1
1975	chr17	43413675	43413675	0.2831	0.3884	0.3586	0
1979	chr17	48089820	48089820	0.8999	0.962	0.9747	0
1981	chr17	50041127	50041127	1	1	1	1
1985	chr17	52953848	52953848	0.2027	0.2002	0.1389	0
1989	chr17	59551584	59551584	0.1563	1	0.1439	1
1992	chr17	63547438	63547442	1	0.9991	1	1
1994	chr17	65970817	65970817	0.3684	0.8636	0.6279	0
1997	chr17	66931725	66931725	0.1498	0.9941	0.1714	0
1999	chr17	68005992	68005992	1	0.9972	1	0
2001	chr17	69879221	69879221	0.2665	0.2677	0.2877	0
2006	chr17	73051761	73051761	0.9726	1	1	1
2009	chr17	75298386	75298386	0.9406	1	1	1
2010	chr17	76073593	76073593	0.9953	1	1	0
2013	chr17	77103709	77103709	0.8487	0.9382	0.9518	1
2025	chr17	80082249	80082249	0.9774	1	1	1
2030	chr17	81007861	81007861	0.6166	0.5579	0.495	1
2035	chr17	81336422	81336422	0.9881	0.9991	1	1
2036	chr17	81352882	81352882	0.7959	0.854	0.8218	0
2056	chr18	2066603	2066603	0.612	0.6894	0.628	1
2057	chr18	3618707	3618707	0.9802	0.9991	1	0
2058	chr18	3669122	3669122	0.1001	0.1262	0.0955	0
2059	chr18	4874672	4874672	0.789	1	0.9906	1
2060	chr18	8963713	8963713	0.9906	1	1	0
2071	chr18	13982057	13982057	0.993	1	1	1
2106	chr18	22550728	22550728	0.8257	0.817	0.8529	1
2108	chr18	23577367	23577367	0.4222	0.9971	0.7931	1
2111	chr18	25887655	25887655	0.529	0.5518	0.48	1
2117	chr18	29951909	29951909	0.0975	0.0771	0.0793	0
2122	chr18	33116802	33116811	0.1547	0.112	0.0891	0
2124	chr18	37283952	37283952	0.8364	0.9991	1	1
2126	chr18	40598044	40598044	1	1	1	1
2131	chr18	48671984	48671984	0.9305	1	1	1
2135	chr18	51423423	51423423	0.7757	0.8701	0.8646	1
2144	chr18	57062203	57062203	0.3952	0.3377	0.3131	0
2152	chr18	61107763	61107763	0.1765	0.3186	0.2347	0
2153	chr18	61644856	61644865	0.5613	0.444	0.4278	1
2158	chr18	63264905	63264905	0.983	0.9981	1	1
2164	chr18	65820051	65820051	0.2151	0.9972	0.3	0
2172	chr18	73171962	73171962	0.4483	1	0.6667	1
2192	chr18	78645474	78645474	0.2936	0.9971	0.3375	1
2230	chr19	1328034	1328034	0.2312	0.9953	0.2838	0
2237	chr19	3128203	3128203	0.9934	1	1	0
2241	chr19	3742712	3742712	0.9901	1	1	0
2243	chr19	3990071	3990071	0.9895	0.9944	1	0
2249	chr19	4739271	4739271	1	0.8582	1	0
2254	chr19	6626296	6626296	0.5964	0.85	0.8041	0
2256	chr19	6991964	6991965	0.2236	0.2459	0.2014	1
2258	chr19	7102803	7102803	0.1714	1	0.234	1

2261	chr19	8621718	8621718	0.8061	1	1	0
2263	chr19	8889008	8889008	0.9026	0.9963	1	0
2264	chr19	9066911	9066911	0.9882	0.9991	1	0
2268	chr19	10715007	10715007	1	0.9981	1	1
2278	chr19	15866491	15866491	0.2526	0.2848	0.2129	0
2280	chr19	16057641	16057785	0.4993	0.3768	0.402	0
2281	chr19	16256453	16256453	0.9986	1	1	0
2282	chr19	18116846	18116846	0.3192	0.9971	0.3025	1
2285	chr19	19116471	19116471	0.3831	1	0.3356	0
2292	chr19	22180935	22180935	0.9983	0.9991	1	1
2295	chr19	23466452	23466452	0.8981	0.9991	1	0
2297	chr19	23828498	23828498	0.9889	1	1	0
2299	chr19	23850382	23850382	1	0.9991	1	0
2301	chr19	24082669	24082669	1	1	1	0
2307	chr19	28732922	28732922	0.4389	0.4356	0.4326	0
2310	chr19	29364877	29364877	0.362	0.4831	0.5465	1
2314	chr19	33458810	33458810	0.6299	0.9991	1	1
2320	chr19	38131836	38131836	0.5531	0.6276	0.599	0
2321	chr19	38546808	38546808	0.9869	1	1	0
2324	chr19	39795753	39795753	0.8416	1	1	0
2325	chr19	39905478	39905478	0.9711	0.9991	1	0
2330	chr19	41510088	41510088	0.9646	1	1	1
2332	chr19	43517413	43517413	0.9987	1	1	0
2333	chr19	43665725	43665725	0.9466	0.9981	1	0
2336	chr19	45325154	45325154	0.9707	1	1	0
2339	chr19	47419530	47419530	0.9455	1	1	1
2342	chr19	49169513	49169513	0.3081	0.1223	0.1078	0
2355	chr19	55488872	55488872	1	0.9971	1	1
2363	chr19	56914164	56914164	0.3761	0.3008	0.2347	0
2366	chr19	58488284	58488284	0.3519	1	0.3056	1
2368	chr2	158188	158188	0.7496	0.8689	0.8506	0
2388	chr2	2750490	2750490	0.9704	0.9937	1	0
2390	chr2	3046162	3046162	0.6035	1	1	0
2398	chr2	4452406	4452406	0.9555	0.9942	1	1
2402	chr2	5488559	5488559	0.8466	0.8679	0.8041	1
2419	chr2	12358210	12358210	0.604	0.9981	0.7875	1
2423	chr2	14274483	14274483	0.6964	0.7875	0.8073	1
2427	chr2	19548655	19548655	0.3151	0.6074	0.5769	0
2435	chr2	25399817	25399817	0.5048	0.5765	0.6053	0
2437	chr2	26714469	26714469	0.9974	1	1	1
2442	chr2	31727141	31727141	0.3554	0.9886	0.3367	0
2443	chr2	31821093	31821093	0.9748	1	1	1
2444	chr2	31823417	31823417	0.9822	1	1	1
2450	chr2	41505928	41505928	0.7486	0.9112	0.9158	0
2461	chr2	47768960	47768960	0.9929	1	1	0
2472	chr2	57832933	57832933	0.7194	0.6769	0.665	1
2474	chr2	61473618	61473631	0.2657	0.2845	0.3372	0
2490	chr2	78024500	78024500	0.0876	0.0836	0.0633	0
2491	chr2	78738300	78738300	0.2211	0.2364	0.2535	0
2492	chr2	80541885	80541885	0.2738	0.2315	0.2857	1
2497	chr2	83932721	83932729	0.3122	0.5814	0.5756	1
2498	chr2	84501631	84501631	0.414	0.9971	0.3333	0
2502	chr2	86056429	86056429	0.1649	0.36	0.3529	0
2513	chr2	90371363	90371363	1	0.9044	1	0
2531	chr2	99121457	99121457	0.9522	0.9971	1	1
2536	chr2	105015796	105015796	0.5273	0.7367	0.7216	0

2544	chr2	113948973	113948974	0.5145	0.6204	0.5765	1
2556	chr2	124294179	124294182	0.3378	0.4248	0.3647	1
2561	chr2	127892113	127892115	0.4195	0.3855	0.3299	0
2563	chr2	129171859	129171860	0.7681	0.8838	0.8866	1
2564	chr2	129685909	129685909	0.0068	1	0	1
2570	chr2	132026027	132026027	0.6739	0.9972	0.7667	0
2571	chr2	132278675	132278675	0.9926	0.99	1	0
2573	chr2	133504330	133504333	0.6712	0.7769	0.7527	1
2575	chr2	135833725	135833725	0.588	0.5971	0.5343	0
2583	chr2	146765597	146765597	0.3304	0.4603	0.4155	0
2586	chr2	150966223	150966223	0.4118	0.598	0.5904	1
2593	chr2	157917517	157917517	0.9954	1	1	0
2596	chr2	161095821	161095821	0.9957	0.9981	1	0
2602	chr2	169496786	169496786	0.0365	0.0957	0.0843	0
2604	chr2	171976069	171976069	0.9732	1	1	1
2608	chr2	177784228	177784228	0.2982	0.3621	0.3533	0
2611	chr2	179475727	179475727	0.7762	1	1	1
2623	chr2	194058380	194058380	0.9742	1	0.9942	1
2624	chr2	194362306	194362306	0.9984	0.9972	1	1
2631	chr2	204274158	204274158	0.9277	1	1	1
2633	chr2	205345784	205345898	0.5648	0.7563	0.7108	0
2635	chr2	206225736	206225741	0.7392	0.6265	0.652	1
2637	chr2	208098602	208098602	0.8642	0.9449	0.9608	1
2641	chr2	209956309	209956309	0.9973	0.9991	1	1
2643	chr2	211843631	211843631	0.1854	0.2394	0.1842	0
2644	chr2	211981039	211981074	0.1691	0.2736	0.2813	0
2647	chr2	213306364	213306364	0.9922	1	1	1
2655	chr2	218082962	218082962	0.7243	1	1	1
2674	chr2	232993181	232993181	0.1639	1	0.1667	0
2676	chr2	233176494	233176606	0.811	1	1	0
2677	chr2	234514840	234514840	0.9778	1	1	0
2682	chr2	237655103	237655103	0.3566	0.2988	0.2696	0
2701	chr2	241178755	241178755	0.996	1	1	0
2718	chr20	4446660	4446665	0.9882	1	1	1
2723	chr20	6063349	6063364	0.2579	0.3796	0.404	0
2775	chr20	29504193	29504195	0.0524	0.9923	0.0597	0
2783	chr20	30153493	30153493	1	0.9981	1	0
2798	chr20	35564372	35564372	0.5	0.9981	0.5263	1
2805	chr20	39582039	39582039	0.1225	0.1133	0.0659	0
2811	chr20	44025528	44025528	0.9941	0.9991	0.9951	1
2813	chr20	44404780	44404780	0.5791	0.9991	1	1
2822	chr20	48010935	48010935	0.3738	1	0.9815	0
2828	chr20	50258483	50258483	0.9886	1	1	1
2831	chr20	50685681	50685681	0.1714	0.1954	0.1282	0
2834	chr20	51740560	51740560	0.9862	1	1	1
2835	chr20	53415694	53415694	0.9857	0.9991	1	1
2838	chr20	54942783	54942783	0.1932	0.1014	0.1071	0
2841	chr20	55625486	55625486	1	0.9991	1	1
2842	chr20	55738278	55738278	0.0748	0.0215	0.0781	0
2847	chr20	58520304	58520304	0.279	0.9971	0.3158	1
2854	chr20	60764341	60764341	0.951	0.9991	1	1
2857	chr20	61337312	61337312	0.6261	0.9991	1	0
2860	chr20	61444270	61444270	0.9768	1	1	1
2864	chr20	61795137	61795137	0.9226	1	1	0
2876	chr20	62504419	62504419	0.5927	1	1	0
2882	chr20	63158462	63158462	0.8619	0.9951	1	1

2954	chr21	19024015	19024015	0.55	0.9991	0.5263	1
2956	chr21	20588483	20588483	0.077	0.9981	0.044	0
2957	chr21	21097962	21097962	0.6421	0.8105	0.8023	1
2969	chr21	24981925	24981925	0.3389	1	0.44	0
2971	chr21	25880083	25880083	0.0835	1	0.0519	1
2976	chr21	29010510	29010510	0.9071	0.9991	1	0
2977	chr21	29118275	29118275	0.9961	1	1	0
2985	chr21	37685535	37685535	0.212	1	0.2283	0
2986	chr21	37707735	37707735	0.5445	1	1	0
2987	chr21	38059655	38059655	0.988	1	1	1
2988	chr21	38508035	38508035	0.5273	1	1	0
2989	chr21	38914020	38914030	0.5992	0.9991	1	0
2994	chr21	41672845	41672845	0.9846	1	1	0
2997	chr21	42994336	42994336	0.9874	0.9491	0.9826	0
3003	chr21	44817899	44817899	0.5893	1	1	1
3026	chr21	46645268	46645268	0.4375	1	0.5385	0
3039	chr22	12568666	12568666	0.9875	0.9892	1	0
3050	chr22	16399802	16399802	0.0145	0.0028	0.0372	0
3067	chr22	19919251	19919251	0.9705	0.9981	1	0
3068	chr22	20252106	20252106	0.6364	0.9934	1	0
3079	chr22	23530492	23530492	0.2189	0.367	0.2053	0
3087	chr22	30597423	30597423	0.9901	1	1	1
3089	chr22	31355889	31355889	1	1	1	0
3095	chr22	34784759	34784759	0.0987	0.1731	0.16	0
3098	chr22	35397424	35397424	0.0986	0.1272	0.1047	0
3106	chr22	38613414	38613414	0.1968	0.9971	0.3	1
3110	chr22	40007384	40007384	0.7803	0.9962	1	0
3113	chr22	41050348	41050348	0.721	0.8822	0.8485	1
3135	chr22	47654609	47654609	0.723	1	1	1
3137	chr22	48117392	48117392	1	0.9962	1	1
3140	chr22	48698690	48698690	0.5309	0.5361	0.5172	0
3148	chr22	49276647	49276652	0.9221	0.9972	0.9949	1
3161	chr22	50697540	50697563	0.1404	1	0.05	0
3180	chr3	7128504	7128504	0.1975	0.2615	0.1688	0
3181	chr3	7779575	7779575	0.5114	0.6958	0.625	1
3187	chr3	10365837	10365837	0.7506	1	1	1
3190	chr3	11599655	11599655	0.0502	0.9972	0.0133	0
3201	chr3	17504195	17504195	0.1078	0.9962	0.1034	1
3208	chr3	23256668	23256668	0.1109	0.1455	0.1776	0
3216	chr3	32674998	32674998	1	1	1	0
3219	chr3	35367773	35367773	1	1	1	1
3224	chr3	38358206	38358206	0.1074	1	0.1304	1
3233	chr3	42346961	42346964	0.2959	0.3287	0.385	0
3236	chr3	44298812	44298812	0.1733	1	0.2463	0
3237	chr3	45133733	45133739	0.9987	0.9981	1	1
3239	chr3	46840340	46840340	0.9622	1	1	0
3240	chr3	47431271	47431271	0.9487	1	1	1
3242	chr3	47784464	47784464	0.9921	1	1	0
3243	chr3	48486260	48486260	0.3318	0.9981	0.4	0
3249	chr3	51596100	51596100	0.9783	1	1	0
3255	chr3	56731610	56731610	0.2265	0.3048	0.2945	0
3268	chr3	66917048	66917048	0.3618	1	0.6429	1
3279	chr3	75414207	75414274	0.9509	0.9379	0.9455	0
3286	chr3	79527977	79527977	0.6909	0.6715	0.6167	0
3287	chr3	79612872	79612872	0.9275	1	1	1
3292	chr3	84900611	84900611	0.5215	0.4379	0.4706	0

3294	chr3	85548222	85548235	0.5271	0.8432	0.7903	0
3296	chr3	87570690	87570729	0.7123	0.8494	0.8144	0
3297	chr3	87601783	87601783	0.0847	0.1112	0.0833	0
3304	chr3	90577058	90577058	0.9844	0.9897	0.9817	0
3314	chr3	91438798	91438815	0.9982	0.9929	1	0
3329	chr3	94929820	94929820	0.9817	1	1	1
3332	chr3	98183496	98183496	0.48	0.6173	0.5987	1
3333	chr3	98196230	98196233	0.5489	0.6225	0.6543	1
3334	chr3	98212354	98212354	0.3465	0.5943	0.5909	1
3338	chr3	99180310	99180310	0.1466	0.106	0.1485	1
3339	chr3	99936555	99936555	0.2471	1	0.35	0
3345	chr3	102829522	102829532	0.7148	0.9051	0.8125	0
3347	chr3	103593531	103593531	0.4138	0.3958	0.3222	1
3368	chr3	124922704	124922704	1	1	1	1
3369	chr3	125089787	125089787	0.8356	0.986	0.9583	1
3381	chr3	133129956	133129956	0.992	1	1	1
3388	chr3	139955612	139955612	0.9617	1	0.9941	1
3389	chr3	142741197	142741197	0.1655	0.2832	0.1923	0
3392	chr3	145272499	145272499	0.6688	0.7586	0.7549	0
3399	chr3	152893522	152893522	0.8034	0.8061	0.8283	1
3406	chr3	162919821	162919821	0.1894	0.2906	0.2857	0
3422	chr3	174762447	174762447	0.7603	0.4939	0.5101	0
3423	chr3	174796724	174796724	0.3542	0.4631	0.4813	1
3424	chr3	175382589	175382589	0.5144	0.6592	0.6683	1
3428	chr3	176223142	176223142	0.3932	0.5482	0.5614	0
3429	chr3	176233749	176233749	0.4954	0.5551	0.6238	0
3433	chr3	176965985	176965988	0.4647	0.6317	0.6011	1
3439	chr3	183661078	183661078	0.355	0.4734	0.3713	1
3440	chr3	183685093	183685093	0.3596	0.4714	0.3873	0
3446	chr3	188516644	188516644	0.1182	0.1607	0.1364	0
3448	chr3	190241590	190241607	0.2559	0.2806	0.26	0
3468	chr4	660975	660975	0.0856	0.9971	0.0305	1
3483	chr4	3594093	3594093	0.9914	0.9981	1	1
3493	chr4	7947942	7947942	0.2078	0.2044	0.3085	0
3497	chr4	9601620	9601620	0.0614	0.4399	0.0179	0
3499	chr4	11717449	11717449	0.6328	0.6583	0.6563	0
3504	chr4	15615473	15615473	1	0.9971	1	1
3509	chr4	21532966	21532966	0.1038	0.1387	0.0608	0
3513	chr4	25988555	25988555	0.0857	0.1308	0.0795	0
3518	chr4	27948169	27948169	0.5884	0.9981	0.7083	0
3524	chr4	37357175	37357175	0.0796	0.0727	0.0602	0
3526	chr4	38754413	38754413	0.5159	0.9991	1	0
3531	chr4	44417615	44417615	0.7652	0.7736	0.7637	1
3574	chr4	52081945	52081945	0.3704	1	0.5	1
3578	chr4	53923793	53923802	0.5934	0.5748	0.6082	1
3584	chr4	56876333	56876333	0.2899	0.2283	0.201	0
3588	chr4	60190268	60190268	0.1425	0.1681	0.1944	0
3593	chr4	67098160	67098160	0.2622	1	0.1803	1
3596	chr4	69220530	69220533	0.9577	0.9977	1	1
3605	chr4	73267225	73267225	0.5125	0.4641	0.4545	1
3612	chr4	76019526	76019526	0.9486	0.9972	1	1
3618	chr4	79783587	79783590	0.9846	1	1	1
3620	chr4	81708994	81708994	0.2772	0.2394	0.1849	0
3623	chr4	87863218	87863218	0.3675	0.9961	0.4314	0
3626	chr4	91014948	91014950	0.2383	0.3178	0.3563	0
3627	chr4	91046151	91046151	0.8795	0.9248	0.915	0

3631	chr4	94492129	94492129	0.1324	0.071	0.0926	0
3633	chr4	96502162	96502162	0.4706	0.5593	0.5446	1
3636	chr4	98761562	98761807	0.6086	0.7927	0.7552	0
3640	chr4	102212199	102212199	0.2705	0.3078	0.1866	0
3649	chr4	114231005	114231005	0.9986	0.9981	1	0
3650	chr4	114362571	114362599	0.2031	0.1405	0.1832	0
3663	chr4	134214182	134214195	0.6524	0.9934	0.9315	0
3664	chr4	134511734	134511734	0.5422	1	0.8306	0
3666	chr4	135586387	135586387	0.1747	0.2586	0.2713	0
3673	chr4	137377851	137377851	0.3961	0.4569	0.4366	0
3679	chr4	142337163	142337163	0.128	0.19	0.0909	0
3698	chr4	156660552	156660552	0.5286	1	0.6406	1
3700	chr4	157982644	157982653	0.995	0.9848	1	1
3705	chr4	164112996	164112996	0.1213	0.0734	0.1139	0
3709	chr4	167804496	167804496	0.997	1	1	0
3713	chr4	169913446	169913446	0.3291	0.2834	0.22	0
3714	chr4	171367700	171367700	0.1981	0.166	0.1987	0
3718	chr4	175048987	175048987	1	1	1	1
3719	chr4	176251280	176251302	0.5387	0.4897	0.4677	0
3724	chr4	179130756	179130805	0.5562	0.7493	0.7056	0
3726	chr4	179431995	179431995	0.5804	0.8158	0.7167	1
3741	chr4	187452645	187452645	0.574	0.8099	0.7798	0
3743	chr4	187673117	187673117	0.2169	0.4074	0.2973	1
3780	chr5	5930998	5930998	1	1	1	1
3781	chr5	6217985	6217985	0.2869	0.2889	0.2955	0
3783	chr5	6868740	6868740	0.9832	1	1	1
3784	chr5	7262724	7262724	1	1	1	0
3785	chr5	7741735	7741735	0.8107	0.9981	1	0
3786	chr5	7845940	7845940	0.2488	0.1988	0.1875	0
3787	chr5	7937089	7937089	0.1819	0.1186	0.0637	0
3791	chr5	10634882	10634882	0.3047	0.3689	0.3134	0
3802	chr5	21875592	21875592	1	1	1	1
3804	chr5	22873701	22873701	0.6609	0.7117	0.6667	1
3811	chr5	31906185	31906185	0.5355	0.4679	0.4489	0
3812	chr5	32338477	32338477	NULL	0.4626	NULL	1
3815	chr5	34459460	34459460	0.2593	0.2847	0.2874	1
3818	chr5	35752662	35752662	1	1	1	1
3819	chr5	36562617	36562617	0.7286	0.7075	0.6947	0
3824	chr5	42089532	42089533	0.9974	0.9981	1	1
3826	chr5	43416740	43416740	0.1171	0.1071	0.0735	0
3827	chr5	43541284	43541284	0.2387	0.9981	0.2348	0
3834	chr5	52531092	52531092	0.8052	0.711	0.7574	1
3855	chr5	73037329	73037329	0.1603	0.3304	0.2286	0
3856	chr5	75128425	75128425	0.5659	0.4827	0.4951	1
3863	chr5	78358363	78358363	0.262	0.3805	0.4576	0
3867	chr5	80251897	80251897	0.5397	0.7464	0.7619	0
3872	chr5	83200572	83200572	0.0366	0.0177	0.0174	0
3874	chr5	85171463	85171463	1	1	1	1
3884	chr5	96516063	96516063	0.4626	0.3749	0.3763	1
3889	chr5	99864310	99864310	0.3214	0.3623	0.3775	0
3890	chr5	100287503	100287516	0.3266	0.343	0.3098	0
3892	chr5	104590996	104590996	0.7934	0.6699	0.6647	1
3899	chr5	113146300	113146300	0.9803	1	0.9901	1
3920	chr5	135778794	135778794	0.9829	1	1	0
3923	chr5	137794470	137794470	0.1935	0.3107	0.2877	0
3930	chr5	144135488	144135488	0.485	0.611	0.4674	1

3932	chr5	147258170	147258170	0.1528	0.1086	0.0298	1
3941	chr5	157778932	157778932	0.9934	1	1	0
3943	chr5	159290164	159290164	0.2718	0.3773	0.2568	0
3961	chr5	173765950	173765950	0.9462	0.9972	1	1
3978	chr5	178999619	178999619	0.2428	0.381	0.3725	0
3982	chr5	179594778	179594778	0.9865	1	1	0
4007	chr6	3776809	3776809	0.6551	1	1	0
4017	chr6	13128170	13128174	0.8668	0.8894	0.8564	1
4022	chr6	13925069	13925069	0.63	1	0.7143	1
4023	chr6	14523508	14523508	0.6102	0.5532	0.5637	1
4024	chr6	14692917	14692917	0.3514	0.9981	1	0
4026	chr6	17249730	17249730	0.0688	0.9972	0.0854	1
4029	chr6	18346169	18346169	0.1967	0.1057	0.1063	0
4035	chr6	23978129	23978129	0.3829	0.1651	0.0641	0
4041	chr6	26043074	26043074	0.7846	0.9065	0.8765	0
4060	chr6	39845371	39845371	0.634	0.9995	1	1
4068	chr6	42919815	42919815	0.9779	0.9971	1	0
4075	chr6	44319587	44319587	0.9071	1	0.9947	0
4083	chr6	47466497	47466538	0.9492	1	1	0
4089	chr6	52826658	52826660	0.9791	0.9995	0.9947	1
4106	chr6	64290970	64290970	0.6276	0.5112	0.5876	0
4109	chr6	65454089	65454089	0.167	0.2363	0.2014	0
4130	chr6	78133467	78133467	0.2819	0.3809	0.3355	0
4131	chr6	79901555	79901555	0.9961	1	1	1
4134	chr6	81048636	81048636	0.0795	0.1411	0.1276	0
4142	chr6	90416146	90416146	0.2634	0.5215	0.3467	0
4146	chr6	94031356	94031694	0.5511	0.6088	0.601	0
4150	chr6	96569807	96569807	0.0934	0.1123	0.1382	0
4151	chr6	97238852	97238860	0.107	1	0.0533	1
4154	chr6	103384150	103384150	0.4674	1	0.3125	1
4158	chr6	106482352	106482352	0.2053	0.3114	0.2892	0
4159	chr6	107000721	107000721	0.6946	0.7694	0.7448	0
4162	chr6	110600712	110600714	0.2915	0.2003	0.18	1
4167	chr6	115585561	115585561	0.1676	0.4388	0.4364	0
4169	chr6	116047604	116047604	0.9917	1	1	1
4176	chr6	120443670	120443670	0.5611	0.9995	0.5455	0
4179	chr6	121840524	121840524	1	1	1	0
4215	chr6	157213166	157213166	0.5112	0.9922	0.4015	1
4216	chr6	157238333	157238333	0.7228	1	1	1
4224	chr6	159604868	159604868	0.8472	1	0.98	1
4228	chr6	160219092	160219096	0.6068	0.9935	1	0
4229	chr6	160235495	160235501	0.8513	0.9528	0.8676	1
4230	chr6	160299197	160299201	0.9375	0.9922	1	0
4232	chr6	160535209	160535209	0.5878	0.9953	1	1
4233	chr6	160669052	160669052	0.8946	0.9991	1	1
4234	chr6	160728335	160728335	1	1	1	1
4235	chr6	160849872	160849872	0.7247	0.9836	0.9167	1
4238	chr6	161374605	161374605	0.951	0.9972	1	1
4245	chr6	163393198	163393198	0.3998	1	0.5714	0
4247	chr6	165840008	165840008	0.9275	1	1	0
4258	chr6	168119996	168119996	0.7636	0.9991	1	1
4259	chr6	168270260	168270260	0.6543	0.9981	1	1
4295	chr7	3120272	3120272	0.6798	1	1	1
4305	chr7	8332682	8332682	0.8206	1	1	1
4310	chr7	12253802	12253802	0.2531	0.115	0.12	0
4312	chr7	13210642	13210642	0.1537	0.2524	0.264	0

4316	chr7	16708103	16708103	0.4358	0.3199	0.2677	0
4317	chr7	19478699	19478704	0.9547	0.972	0.9588	1
4321	chr7	23503699	23503699	1	1	1	0
4329	chr7	31657166	31657166	0.9661	0.9991	1	1
4332	chr7	32791629	32791629	0.1422	0.1084	0.0671	0
4334	chr7	33749078	33749078	0.9707	0.9991	1	1
4336	chr7	34800842	34800861	0.9986	1	1	0
4340	chr7	38720115	38720115	0.2532	0.3653	0.3514	0
4343	chr7	41271202	41271202	0.0547	0.0556	0.0438	0
4352	chr7	46062579	46062579	0.1174	0.1067	0.119	0
4358	chr7	48194058	48194100	0.8485	1	1	0
4359	chr7	48481582	48481582	0.4109	0.6139	0.5889	1
4368	chr7	52703778	52703778	0.4192	0.5423	0.4236	1
4373	chr7	57493118	57493118	0.9888	1	1	0
4394	chr7	64829559	64829559	0.9908	1	0.9946	1
4401	chr7	68462839	68462839	0.1873	0.9971	0.1765	0
4402	chr7	68761514	68761514	0.9895	0.9981	1	1
4407	chr7	72844274	72844298	0.2158	0.3044	0.3627	0
4414	chr7	75058095	75058098	0.8603	0.9668	0.9521	1
4415	chr7	75906887	75906887	0.9971	0.9991	1	0
4416	chr7	76414827	76414827	0.9971	1	1	0
4422	chr7	81066090	81066090	0.6947	0.958	0.9136	0
4423	chr7	81255227	81255227	0.2844	0.3263	0.2363	0
4428	chr7	83222440	83222440	0.2066	0.1991	0.1489	0
4429	chr7	88110882	88110882	0.9482	1	1	0
4430	chr7	88444262	88444262	0.9986	0.9991	1	0
4434	chr7	92122238	92122238	0.2065	0.1712	0.125	0
4435	chr7	92552153	92552153	0.9851	0.9981	0.9947	0
4436	chr7	93405381	93405381	0.617	0.9099	0.8523	1
4437	chr7	93517305	93517305	0.1869	0.3046	0.2569	1
4442	chr7	95741489	95741489	0.2521	0.3613	0.4022	0
4443	chr7	97438226	97438226	0.2282	0.4393	0.2302	0
4447	chr7	99219897	99219897	0.8957	1	1	1
4448	chr7	99549043	99549043	0.855	0.9991	1	1
4453	chr7	101191965	101191965	0.3355	1	0.3017	1
4456	chr7	102260388	102260388	0.8784	0.9986	1	0
4459	chr7	102932561	102932561	0.988	1	1	1
4465	chr7	106981710	106981710	1	0.9843	1	0
4468	chr7	107730235	107730235	0.996	1	1	0
4470	chr7	111195767	111195767	0.7056	1	0.7429	1
4473	chr7	113339572	113339572	0.3829	0.666	0.6075	1
4484	chr7	124426023	124426023	0.8136	0.8131	0.759	1
4487	chr7	125239057	125239057	0.5768	0.6477	0.5707	1
4488	chr7	127230174	127230174	0.0485	0.0271	0.0449	0
4489	chr7	128289277	128289277	0.4776	0.9902	0.5116	0
4498	chr7	132608124	132608124	0.9894	0.9942	1	0
4502	chr7	136498573	136498575	0.9226	0.9939	0.988	1
4506	chr7	138650801	138650801	0.9934	1	1	0
4509	chr7	139101511	139101511	0.9948	0.9981	1	0
4512	chr7	142659903	142659903	0.3805	1	0.6078	0
4514	chr7	148936255	148936255	0.803	0.7647	0.7278	0
4521	chr7	151315017	151315019	0.2212	0.4178	0.3929	0
4525	chr7	152540442	152540442	1	0.9933	1	1
4530	chr7	154264991	154264991	0.7304	0.9991	1	1
4531	chr7	154268965	154268965	0.3059	0.4331	0.3403	0
4536	chr7	156101312	156101312	0.8381	0.9991	1	0

4578	chr8	439575	439575	0.0027	0.0065	0.0101	0
4593	chr8	5971760	5971765	0.3575	0.73	0.4783	1
4601	chr8	9771920	9771920	0.8571	1	1	1
4603	chr8	10149871	10149871	0.8894	0.8662	0.9	1
4604	chr8	10271520	10271524	0.7824	0.8804	0.838	1
4605	chr8	11169220	11169220	0.0278	0.0215	0.0461	0
4610	chr8	15691011	15691011	0.1533	0.2338	0.1462	0
4612	chr8	16674062	16674063	1	0.9995	1	1
4619	chr8	20621985	20621985	0.9854	1	1	0
4622	chr8	21766797	21766797	0.8342	0.9981	1	1
4623	chr8	22709296	22709296	0.6985	1	1	1
4630	chr8	26027550	26027550	0.9856	1	1	1
4634	chr8	27438465	27438465	0.702	0.5658	0.5158	1
4636	chr8	29820459	29820461	0.6744	0.9991	1	1
4637	chr8	30740468	30740468	0.9921	1	1	0
4645	chr8	36525567	36525582	0.2358	0.2743	0.1947	0
4649	chr8	38918553	38918553	0.8975	1	1	1
4652	chr8	40820766	40820766	0.7315	0.9991	1	1
4654	chr8	41412939	41412939	0.2488	0.9961	0.3974	1
4660	chr8	47764037	47764037	0.9878	1	1	0
4661	chr8	48174023	48174023	0.7651	1	1	0
4662	chr8	49713404	49713404	0.8309	0.9533	0.9255	1
4664	chr8	51790814	51790814	0.5355	0.5761	0.5464	1
4666	chr8	53594013	53594013	0.7289	1	1	1
4670	chr8	57072061	57072061	0.9987	1	1	0
4671	chr8	58703431	58703431	0.2249	0.1895	0.1173	0
4674	chr8	61213323	61213323	0.8312	0.9692	0.9608	1
4675	chr8	63092056	63092060	0.9711	1	1	1
4677	chr8	64549215	64549217	0.505	0.6745	0.5655	1
4678	chr8	65040539	65040542	0.6906	0.7673	0.7444	1
4683	chr8	69160400	69160400	0.9681	0.9991	1	0
4684	chr8	72111387	72111629	0.5454	0.6008	0.5928	0
4690	chr8	77036103	77036103	0.1741	0.9951	0.2381	1
4695	chr8	84962077	84962077	0.9985	1	1	1
4696	chr8	85262121	85262121	0.8314	1	0.8509	1
4712	chr8	108791896	108791896	0.1069	0.1489	0.1683	1
4714	chr8	115398732	115398732	0.9986	1	1	1
4717	chr8	117608519	117608519	0.0625	0.9981	0.0407	0
4733	chr8	127521605	127521605	0.7285	0.7953	0.6645	0
4737	chr8	130897702	130897702	0.7079	1	1	0
4738	chr8	132933298	132933298	0.9817	0.9981	1	1
4742	chr8	136026912	136026912	0.972	1	1	1
4749	chr8	138313722	138313722	0.3333	0.3994	0.2132	0
4751	chr8	139157950	139157950	0.755	0.6018	0.5515	1
4770	chr8	143988337	143988337	0.7444	1	1	0
4771	chr8	144021102	144021263	0.9185	0.9981	1	0
4783	chr9	129766	129766	0.1337	0.357	0.3696	0
4788	chr9	817722	817722	0.4045	0.6402	0.6022	0
4795	chr9	2781617	2781617	0.6176	0.6753	0.6354	1
4802	chr9	6727606	6727606	0.5061	0.45	0.4643	0
4805	chr9	6978413	6978413	0.0063	0.9895	0	0
4808	chr9	9008030	9008030	0.5775	0.5224	0.495	0
4810	chr9	10798390	10798394	0.4455	0.627	0.6139	1
4814	chr9	12026316	12026316	0.3495	0.9961	0.5	0
4826	chr9	19671056	19671059	0.4682	0.873	0.8086	1
4842	chr9	29877652	29877652	0.4706	0.6839	0.76	1

4843	chr9	31978475	31978475	0.4682	0.7725	0.675	1
4845	chr9	32883288	32883291	0.5608	0.8289	0.6875	1
4846	chr9	33130561	33130561	0.3949	0.5461	0.4839	0
4924	chr9	72029976	72029976	0.1874	0.9962	0.2095	1
4926	chr9	74972504	74972504	0.4187	0.4099	0.4208	1
4927	chr9	76280876	76280876	0.1298	0.0921	0.0625	0
4928	chr9	76539568	76539568	0.3463	1	0.407	1
4929	chr9	77452611	77452611	0.6233	1	1	1
4935	chr9	81197428	81197428	0.9851	0.9991	1	1
4936	chr9	81431675	81431675	0.2444	0.2866	0.2696	0
4938	chr9	83888038	83888038	0.9938	1	1	0
4940	chr9	86999017	86999017	0.8523	0.9407	0.9439	1
4946	chr9	88096330	88096330	0.1182	0.1725	0.1067	0
4948	chr9	88659629	88659629	0.9948	1	1	0
4949	chr9	88974430	88974430	0.6974	0.9991	1	1
4950	chr9	89040233	89040233	0.9318	1	1	1
4953	chr9	90432262	90432262	0.2796	0.5447	0.2891	0
4958	chr9	93208752	93208752	0.5845	1	1	1
4959	chr9	93211257	93211257	0.9289	1	1	1
4966	chr9	98595559	98595559	0.5569	0.6998	0.6895	1
4972	chr9	105087642	105087646	0.4049	0.5341	0.495	1
4977	chr9	108455199	108455199	0.4706	0.9942	0.6	0
4978	chr9	109350879	109350879	0.0831	0.178	0.2337	1
4987	chr9	116936097	116936097	0.7011	0.9971	1	1
4991	chr9	121895762	121895762	0.9751	0.9981	1	1
4993	chr9	123347685	123347685	0.8623	1	1	1
4995	chr9	124445060	124445060	0.2588	0.999	0.5	1
4996	chr9	125265677	125265677	0.9782	1	1	0
5000	chr9	125814179	125814179	0.9961	1	1	0
5006	chr9	127368427	127368427	0.0384	0.0005	0.0432	0
5008	chr9	127882602	127882602	0.9983	1	1	0
5011	chr9	129689821	129689821	0.2564	0.3016	0.3507	0
5017	chr9	131789213	131789213	0.0696	1	0.0536	1
5026	chr9	134301279	134301279	0.055	0.9972	0	1
5038	chr9	135350252	135350252	0.748	0.6508	0.6598	0
