# Supplementary material for: Construction of JRG (Japanese reference genome) with single-molecule real-time sequencing
Source: Hum Genome Var. 2019 Jun 7;6:27. doi: 10.1038/s41439-019-0057-7 (PMC6555796; doi:10.1038/s41439-019-0057-7)
Supplement: Supplementary file 6 — Supplementary Material 1 [file 41439_2019_57_MOESM6_ESM.pdf]

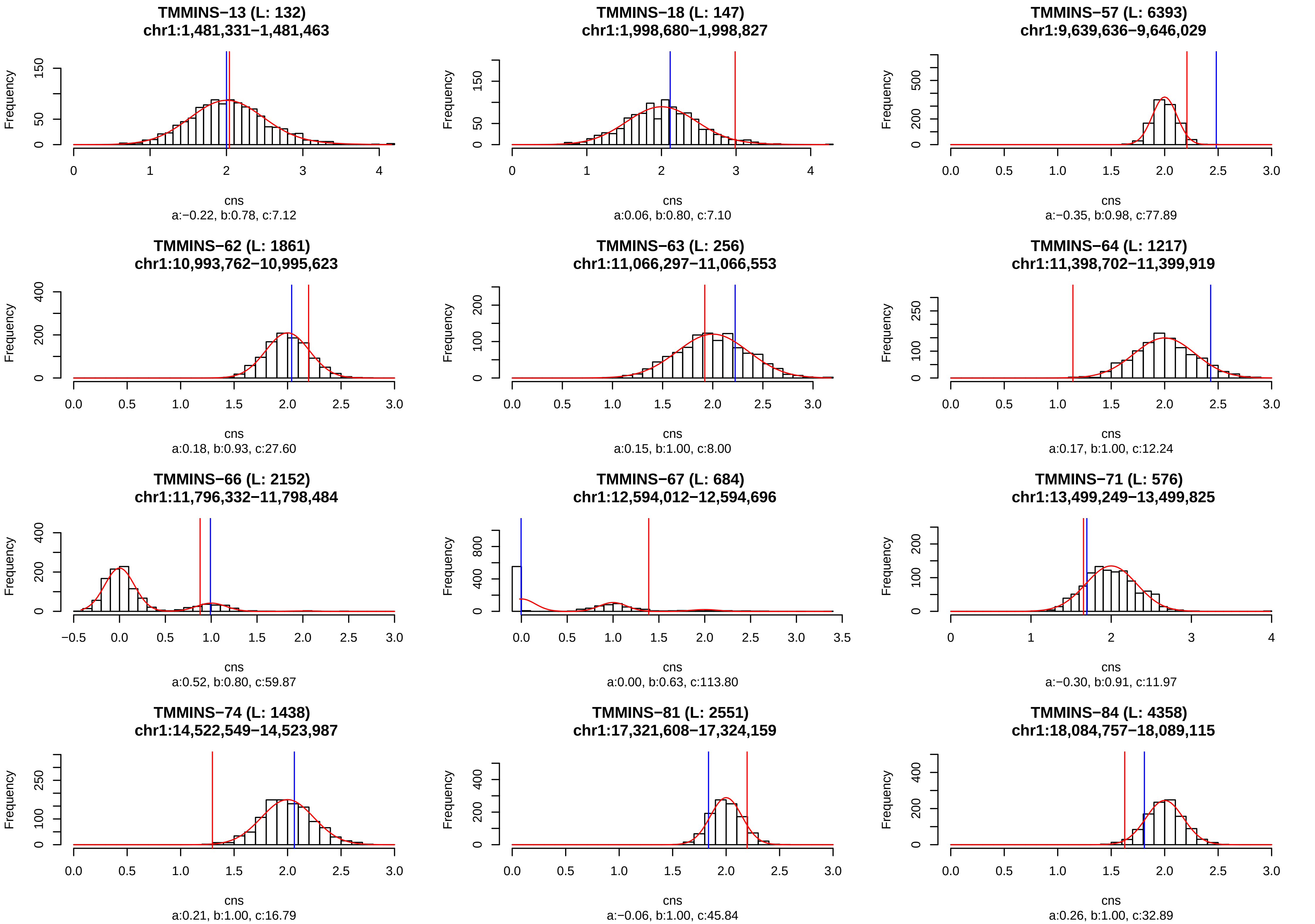
TMMINS−13 (L: 132)
chr1:1,481,331−1,481,463
Frequency
cns
a:−0.22, b:0.78, c:7.12
TMMINS−18 (L: 147)
chr1:1,998,680−1,998,827
Frequency
cns
a:0.06, b:0.80, c:7.10
TMMINS−57 (L: 6393)
chr1:9,639,636−9,646,029
Frequency
cns
a:−0.35, b:0.98, c:77.89
TMMINS−62 (L: 1861)
chr1:10,993,762−10,995,623
Frequency
cns
a:0.18, b:0.93, c:27.60
TMMINS−63 (L: 256)
chr1:11,066,297−11,066,553
Frequency
cns
a:0.15, b:1.00, c:8.00
TMMINS−64 (L: 1217)
chr1:11,398,702−11,399,919
Frequency
cns
a:0.17, b:1.00, c:12.24
TMMINS−66 (L: 2152)
chr1:11,796,332−11,798,484
Frequency
cns
a:0.52, b:0.80, c:59.87
TMMINS−67 (L: 684)
chr1:12,594,012−12,594,696
Frequency
cns
a:0.00, b:0.63, c:113.80
TMMINS−71 (L: 576)
chr1:13,499,249−13,499,825
Frequency
cns
a:−0.30, b:0.91, c:11.97
TMMINS−74 (L: 1438)
chr1:14,522,549−14,523,987
Frequency
cns
a:0.21, b:1.00, c:16.79
TMMINS−81 (L: 2551)
chr1:17,321,608−17,324,159
Frequency
cns
a:−0.06, b:1.00, c:45.84
TMMINS−84 (L: 4358)
chr1:18,084,757−18,089,115
Frequency
cns
a:0.26, b:1.00, c:32.89

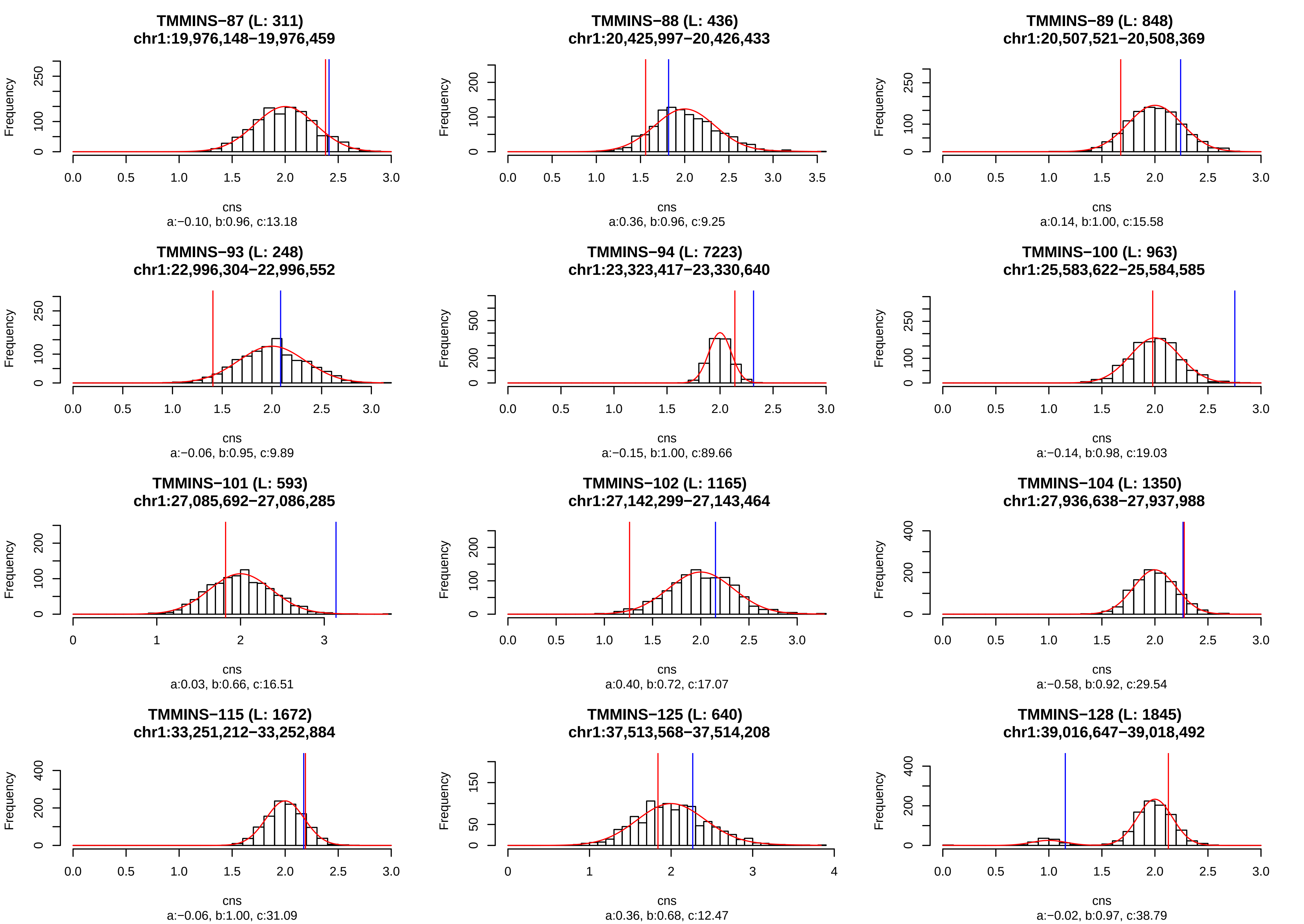
TMMINS−87 (L: 311)
chr1:19,976,148−19,976,459
Frequency
cns
a:−0.10, b:0.96, c:13.18
TMMINS−88 (L: 436)
chr1:20,425,997−20,426,433
Frequency
cns
a:0.36, b:0.96, c:9.25
TMMINS−89 (L: 848)
chr1:20,507,521−20,508,369
Frequency
cns
a:0.14, b:1.00, c:15.58
TMMINS−93 (L: 248)
chr1:22,996,304−22,996,552
Frequency
cns
a:−0.06, b:0.95, c:9.89
TMMINS−94 (L: 7223)
chr1:23,323,417−23,330,640
Frequency
cns
a:−0.15, b:1.00, c:89.66
TMMINS−100 (L: 963)
chr1:25,583,622−25,584,585
Frequency
cns
a:−0.14, b:0.98, c:19.03
TMMINS−101 (L: 593)
chr1:27,085,692−27,086,285
Frequency
cns
a:0.03, b:0.66, c:16.51
TMMINS−102 (L: 1165)
chr1:27,142,299−27,143,464
Frequency
cns
a:0.40, b:0.72, c:17.07
TMMINS−104 (L: 1350)
chr1:27,936,638−27,937,988
Frequency
cns
a:−0.58, b:0.92, c:29.54
TMMINS−115 (L: 1672)
chr1:33,251,212−33,252,884
Frequency
cns
a:−0.06, b:1.00, c:31.09
TMMINS−125 (L: 640)
chr1:37,513,568−37,514,208
Frequency
cns
a:0.36, b:0.68, c:12.47
TMMINS−128 (L: 1845)
chr1:39,016,647−39,018,492
Frequency
cns
a:−0.02, b:0.97, c:38.79

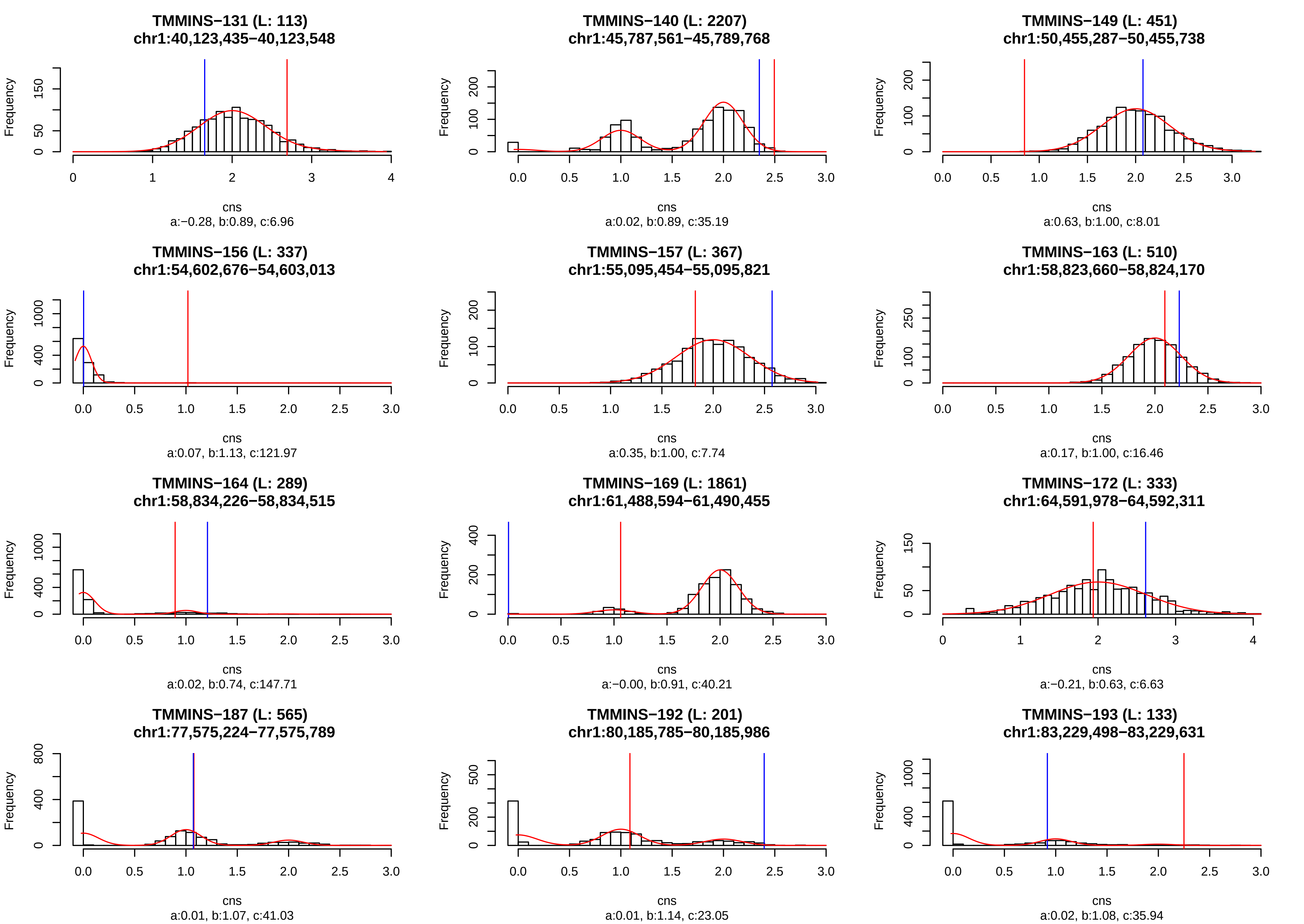
TMMINS−131 (L: 113)
chr1:40,123,435−40,123,548
Frequency
cns
a:−0.28, b:0.89, c:6.96
TMMINS−140 (L: 2207)
chr1:45,787,561−45,789,768
Frequency
cns
a:0.02, b:0.89, c:35.19
TMMINS−149 (L: 451)
chr1:50,455,287−50,455,738
Frequency
cns
a:0.63, b:1.00, c:8.01
TMMINS−156 (L: 337)
chr1:54,602,676−54,603,013
Frequency
cns
a:0.07, b:1.13, c:121.97
TMMINS−157 (L: 367)
chr1:55,095,454−55,095,821
Frequency
cns
a:0.35, b:1.00, c:7.74
TMMINS−163 (L: 510)
chr1:58,823,660−58,824,170
Frequency
cns
a:0.17, b:1.00, c:16.46
TMMINS−164 (L: 289)
chr1:58,834,226−58,834,515
Frequency
cns
a:0.02, b:0.74, c:147.71
TMMINS−169 (L: 1861)
chr1:61,488,594−61,490,455
Frequency
cns
a:−0.00, b:0.91, c:40.21
TMMINS−172 (L: 333)
chr1:64,591,978−64,592,311
Frequency
cns
a:−0.21, b:0.63, c:6.63
TMMINS−187 (L: 565)
chr1:77,575,224−77,575,789
Frequency
cns
a:0.01, b:1.07, c:41.03
TMMINS−192 (L: 201)
chr1:80,185,785−80,185,986
Frequency
cns
a:0.01, b:1.14, c:23.05
TMMINS−193 (L: 133)
chr1:83,229,498−83,229,631
Frequency
cns
a:0.02, b:1.08, c:35.94

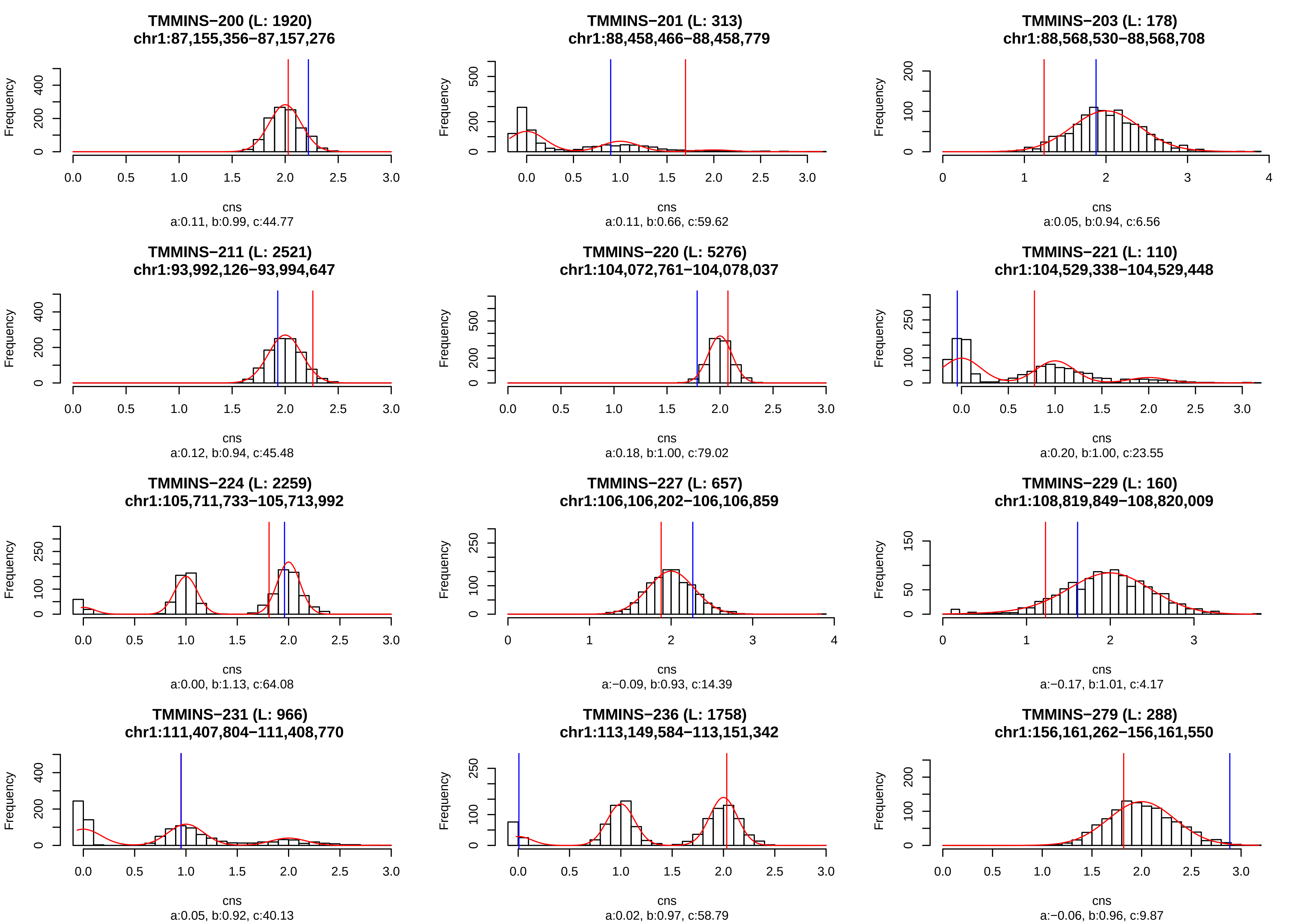
TMMINS−200 (L: 1920)
chr1:87,155,356−87,157,276
Frequency
cns
a:0.11, b:0.99, c:44.77
TMMINS−201 (L: 313)
chr1:88,458,466−88,458,779
Frequency
cns
a:0.11, b:0.66, c:59.62
TMMINS−203 (L: 178)
chr1:88,568,530−88,568,708
Frequency
cns
a:0.05, b:0.94, c:6.56
TMMINS−211 (L: 2521)
chr1:93,992,126−93,994,647
Frequency
cns
a:0.12, b:0.94, c:45.48
TMMINS−220 (L: 5276)
chr1:104,072,761−104,078,037
Frequency
cns
a:0.18, b:1.00, c:79.02
TMMINS−221 (L: 110)
chr1:104,529,338−104,529,448
Frequency
cns
a:0.20, b:1.00, c:23.55
TMMINS−224 (L: 2259)
chr1:105,711,733−105,713,992
Frequency
cns
a:0.00, b:1.13, c:64.08
TMMINS−227 (L: 657)
chr1:106,106,202−106,106,859
Frequency
cns
a:−0.09, b:0.93, c:14.39
TMMINS−229 (L: 160)
chr1:108,819,849−108,820,009
Frequency
cns
a:−0.17, b:1.01, c:4.17
TMMINS−231 (L: 966)
chr1:111,407,804−111,408,770
Frequency
cns
a:0.05, b:0.92, c:40.13
TMMINS−236 (L: 1758)
chr1:113,149,584−113,151,342
Frequency
cns
a:0.02, b:0.97, c:58.79
TMMINS−279 (L: 288)
chr1:156,161,262−156,161,550
Frequency
cns
a:−0.06, b:0.96, c:9.87

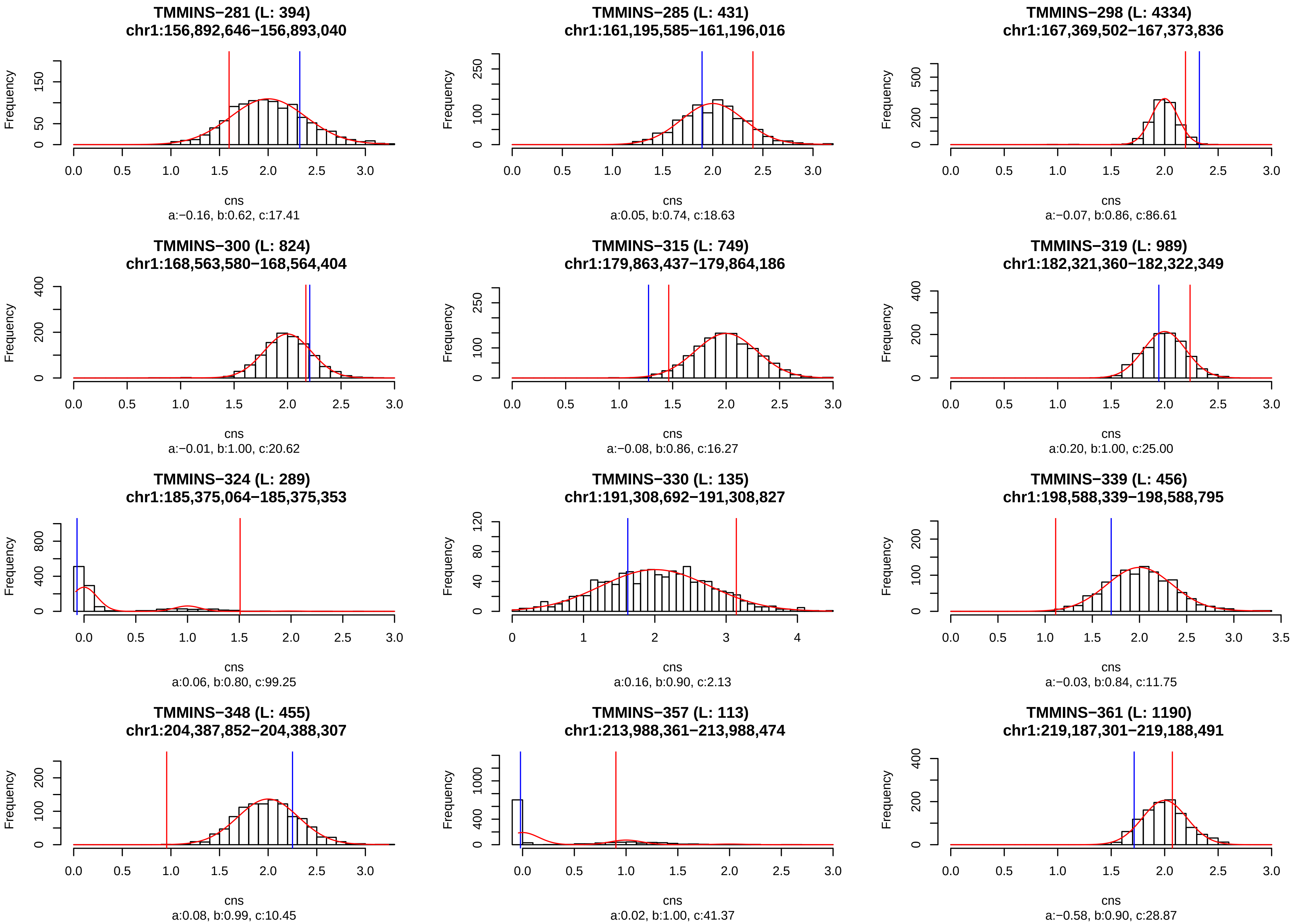
TMMINS−281 (L: 394)
chr1:156,892,646−156,893,040
Frequency
cns
a:−0.16, b:0.62, c:17.41
TMMINS−285 (L: 431)
chr1:161,195,585−161,196,016
Frequency
cns
a:0.05, b:0.74, c:18.63
TMMINS−298 (L: 4334)
chr1:167,369,502−167,373,836
Frequency
cns
a:−0.07, b:0.86, c:86.61
TMMINS−300 (L: 824)
chr1:168,563,580−168,564,404
Frequency
cns
a:−0.01, b:1.00, c:20.62
TMMINS−315 (L: 749)
chr1:179,863,437−179,864,186
Frequency
cns
a:−0.08, b:0.86, c:16.27
TMMINS−319 (L: 989)
chr1:182,321,360−182,322,349
Frequency
cns
a:0.20, b:1.00, c:25.00
TMMINS−324 (L: 289)
chr1:185,375,064−185,375,353
Frequency
cns
a:0.06, b:0.80, c:99.25
TMMINS−330 (L: 135)
chr1:191,308,692−191,308,827
Frequency
cns
a:0.16, b:0.90, c:2.13
TMMINS−339 (L: 456)
chr1:198,588,339−198,588,795
Frequency
cns
a:−0.03, b:0.84, c:11.75
TMMINS−348 (L: 455)
chr1:204,387,852−204,388,307
Frequency
cns
a:0.08, b:0.99, c:10.45
TMMINS−357 (L: 113)
chr1:213,988,361−213,988,474
Frequency
cns
a:0.02, b:1.00, c:41.37
TMMINS−361 (L: 1190)
chr1:219,187,301−219,188,491
Frequency
cns
a:−0.58, b:0.90, c:28.87

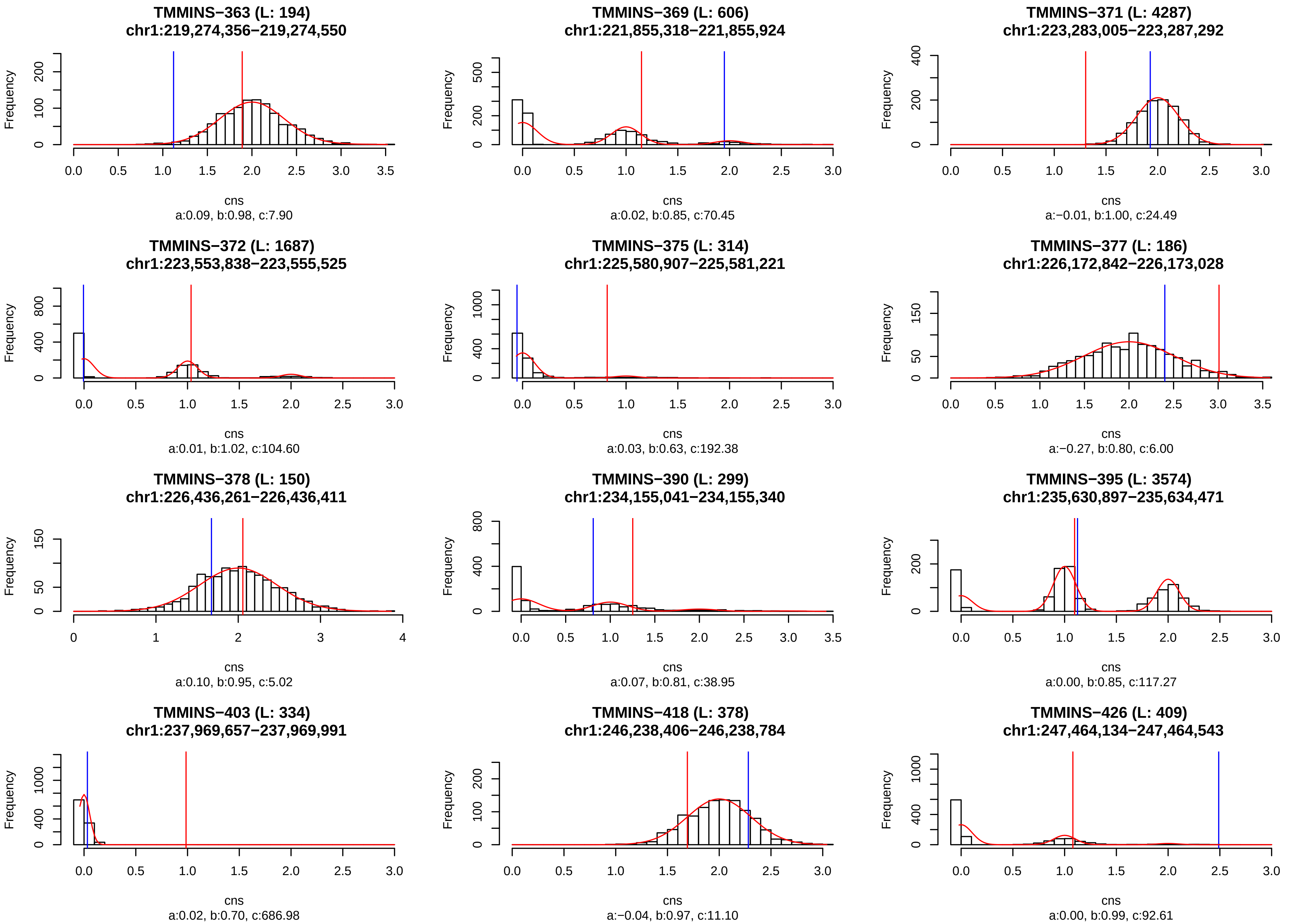
TMMINS−363 (L: 194)
chr1:219,274,356−219,274,550
Frequency
cns
a:0.09, b:0.98, c:7.90
TMMINS−369 (L: 606)
chr1:221,855,318−221,855,924
Frequency
cns
a:0.02, b:0.85, c:70.45
TMMINS−371 (L: 4287)
chr1:223,283,005−223,287,292
Frequency
cns
a:−0.01, b:1.00, c:24.49
TMMINS−372 (L: 1687)
chr1:223,553,838−223,555,525
Frequency
cns
a:0.01, b:1.02, c:104.60
TMMINS−375 (L: 314)
chr1:225,580,907−225,581,221
Frequency
cns
a:0.03, b:0.63, c:192.38
TMMINS−377 (L: 186)
chr1:226,172,842−226,173,028
Frequency
cns
a:−0.27, b:0.80, c:6.00
TMMINS−378 (L: 150)
chr1:226,436,261−226,436,411
Frequency
cns
a:0.10, b:0.95, c:5.02
TMMINS−390 (L: 299)
chr1:234,155,041−234,155,340
Frequency
cns
a:0.07, b:0.81, c:38.95
TMMINS−395 (L: 3574)
chr1:235,630,897−235,634,471
Frequency
cns
a:0.00, b:0.85, c:117.27
TMMINS−403 (L: 334)
chr1:237,969,657−237,969,991
Frequency
cns
a:0.02, b:0.70, c:686.98
TMMINS−418 (L: 378)
chr1:246,238,406−246,238,784
Frequency
cns
a:−0.04, b:0.97, c:11.10
TMMINS−426 (L: 409)
chr1:247,464,134−247,464,543
Frequency
cns
a:0.00, b:0.99, c:92.61

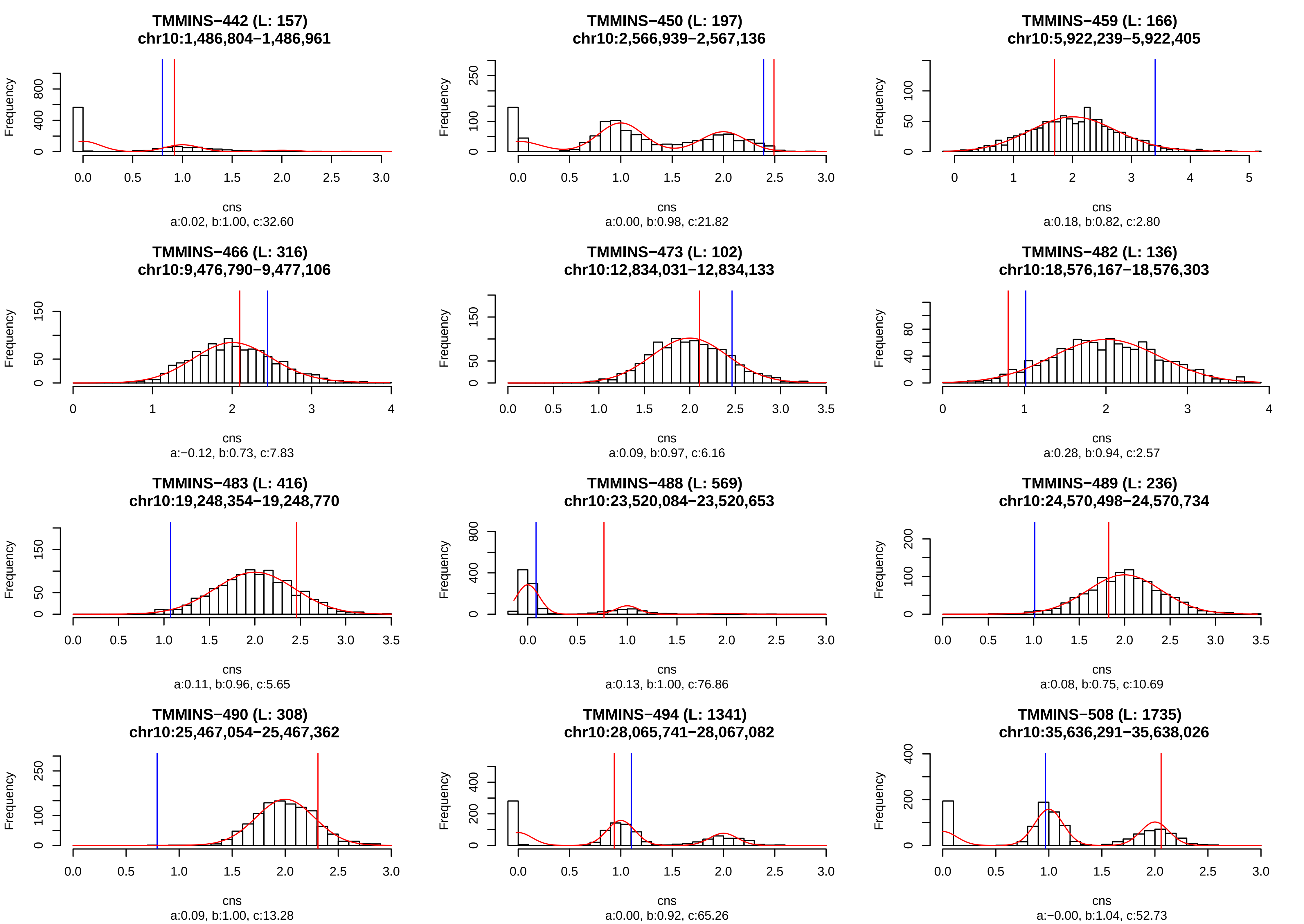
TMMINS−442 (L: 157)
chr10:1,486,804−1,486,961
Frequency
cns
a:0.02, b:1.00, c:32.60
TMMINS−450 (L: 197)
chr10:2,566,939−2,567,136
Frequency
cns
a:0.00, b:0.98, c:21.82
TMMINS−459 (L: 166)
chr10:5,922,239−5,922,405
Frequency
cns
a:0.18, b:0.82, c:2.80
TMMINS−466 (L: 316)
chr10:9,476,790−9,477,106
Frequency
cns
a:−0.12, b:0.73, c:7.83
TMMINS−473 (L: 102)
chr10:12,834,031−12,834,133
Frequency
cns
a:0.09, b:0.97, c:6.16
TMMINS−482 (L: 136)
chr10:18,576,167−18,576,303
Frequency
cns
a:0.28, b:0.94, c:2.57
TMMINS−483 (L: 416)
chr10:19,248,354−19,248,770
Frequency
cns
a:0.11, b:0.96, c:5.65
TMMINS−488 (L: 569)
chr10:23,520,084−23,520,653
Frequency
cns
a:0.13, b:1.00, c:76.86
TMMINS−489 (L: 236)
chr10:24,570,498−24,570,734
Frequency
cns
a:0.08, b:0.75, c:10.69
TMMINS−490 (L: 308)
chr10:25,467,054−25,467,362
Frequency
cns
a:0.09, b:1.00, c:13.28
TMMINS−494 (L: 1341)
chr10:28,065,741−28,067,082
Frequency
cns
a:0.00, b:0.92, c:65.26
TMMINS−508 (L: 1735)
chr10:35,636,291−35,638,026
Frequency
cns
a:−0.00, b:1.04, c:52.73

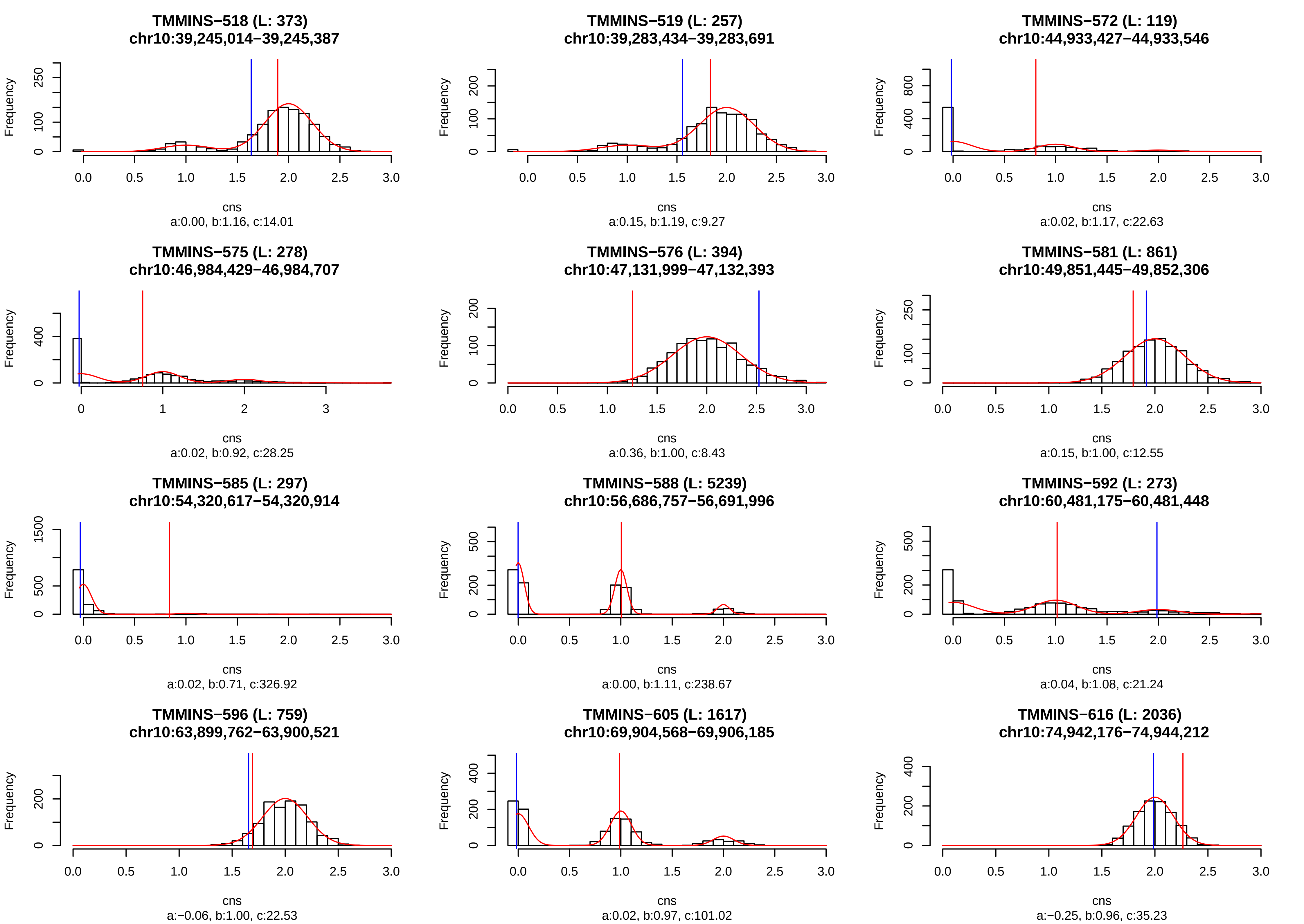

TMMINS−518 (L: 373)
chr10:39,245,014−39,245,387
a:0.00, b:1.16, c:14.01
TMMINS−519 (L: 257)
chr10:39,283,434−39,283,691
a:0.15, b:1.19, c:9.27
TMMINS−572 (L: 119)
chr10:44,933,427−44,933,546
a:0.02, b:1.17, c:22.63
TMMINS−575 (L: 278)
chr10:46,984,429−46,984,707
a:0.02, b:0.92, c:28.25
TMMINS−576 (L: 394)
chr10:47,131,999−47,132,393
a:0.36, b:1.00, c:8.43
TMMINS−581 (L: 861)
chr10:49,851,445−49,852,306
a:0.15, b:1.00, c:12.55
TMMINS−585 (L: 297)
chr10:54,320,617−54,320,914
a:0.02, b:0.71, c:326.92
TMMINS−588 (L: 5239)
chr10:56,686,757−56,691,996
a:0.00, b:1.11, c:238.67
TMMINS−592 (L: 273)
chr10:60,481,175−60,481,448
a:0.04, b:1.08, c:21.24
TMMINS−596 (L: 759)
chr10:63,899,762−63,900,521
a:−0.06, b:1.00, c:22.53
TMMINS−605 (L: 1617)
chr10:69,904,568−69,906,185
a:0.02, b:0.97, c:101.02
TMMINS−616 (L: 2036)
chr10:74,942,176−74,944,212
a:−0.25, b:0.96, c:35.23
Frequency
cns

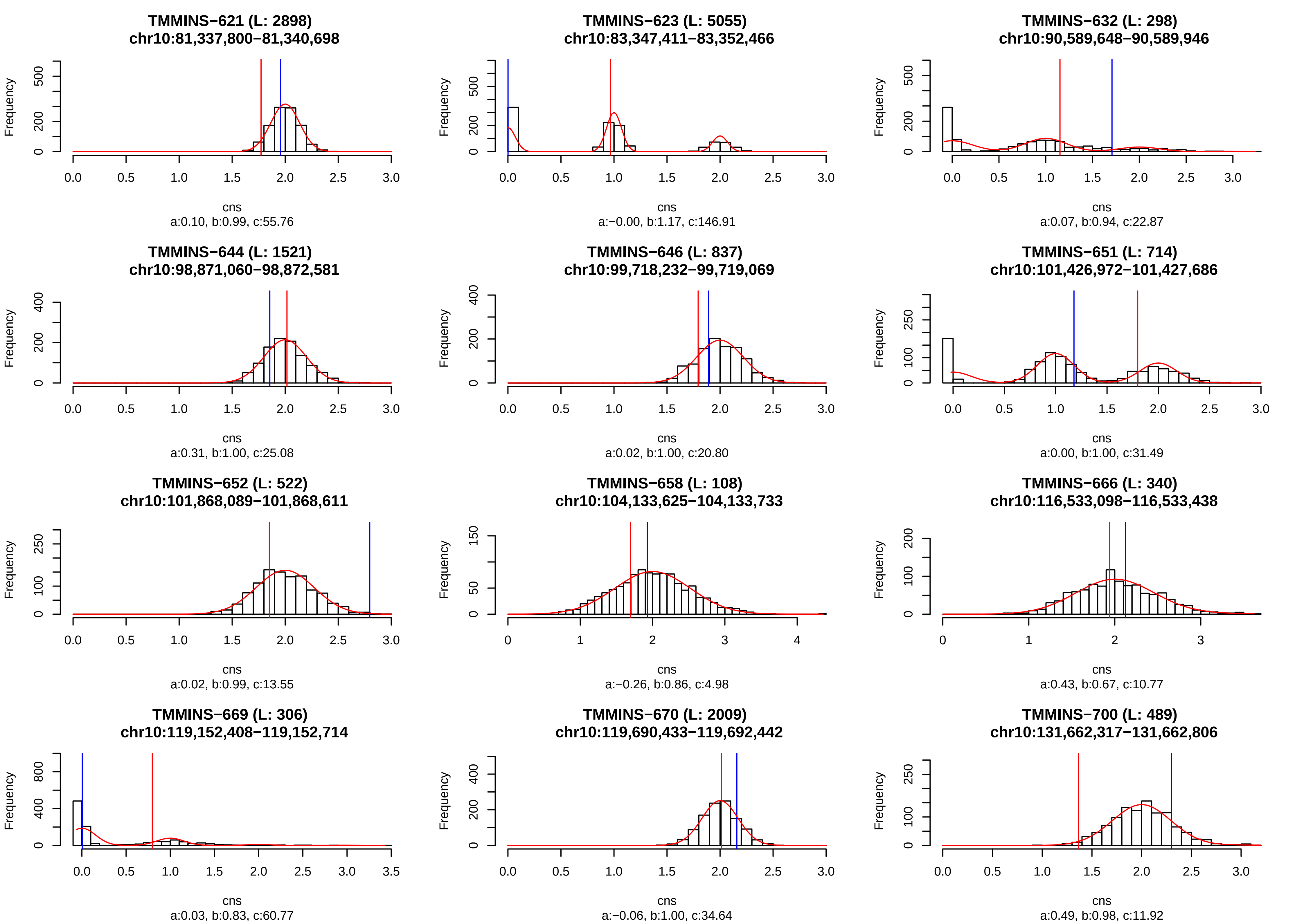
TMMINS−621 (L: 2898)
chr10:81,337,800−81,340,698
Frequency
cns
a:0.10, b:0.99, c:55.76
TMMINS−623 (L: 5055)
chr10:83,347,411−83,352,466
Frequency
cns
a:−0.00, b:1.17, c:146.91
TMMINS−632 (L: 298)
chr10:90,589,648−90,589,946
Frequency
cns
a:0.07, b:0.94, c:22.87
TMMINS−644 (L: 1521)
chr10:98,871,060−98,872,581
Frequency
cns
a:0.31, b:1.00, c:25.08
TMMINS−646 (L: 837)
chr10:99,718,232−99,719,069
Frequency
cns
a:0.02, b:1.00, c:20.80
TMMINS−651 (L: 714)
chr10:101,426,972−101,427,686
Frequency
cns
a:0.00, b:1.00, c:31.49
TMMINS−652 (L: 522)
chr10:101,868,089−101,868,611
Frequency
cns
a:0.02, b:0.99, c:13.55
TMMINS−658 (L: 108)
chr10:104,133,625−104,133,733
Frequency
cns
a:−0.26, b:0.86, c:4.98
TMMINS−666 (L: 340)
chr10:116,533,098−116,533,438
Frequency
cns
a:0.43, b:0.67, c:10.77
TMMINS−669 (L: 306)
chr10:119,152,408−119,152,714
Frequency
cns
a:0.03, b:0.83, c:60.77
TMMINS−670 (L: 2009)
chr10:119,690,433−119,692,442
Frequency
cns
a:−0.06, b:1.00, c:34.64
TMMINS−700 (L: 489)
chr10:131,662,317−131,662,806
Frequency
cns
a:0.49, b:0.98, c:11.92

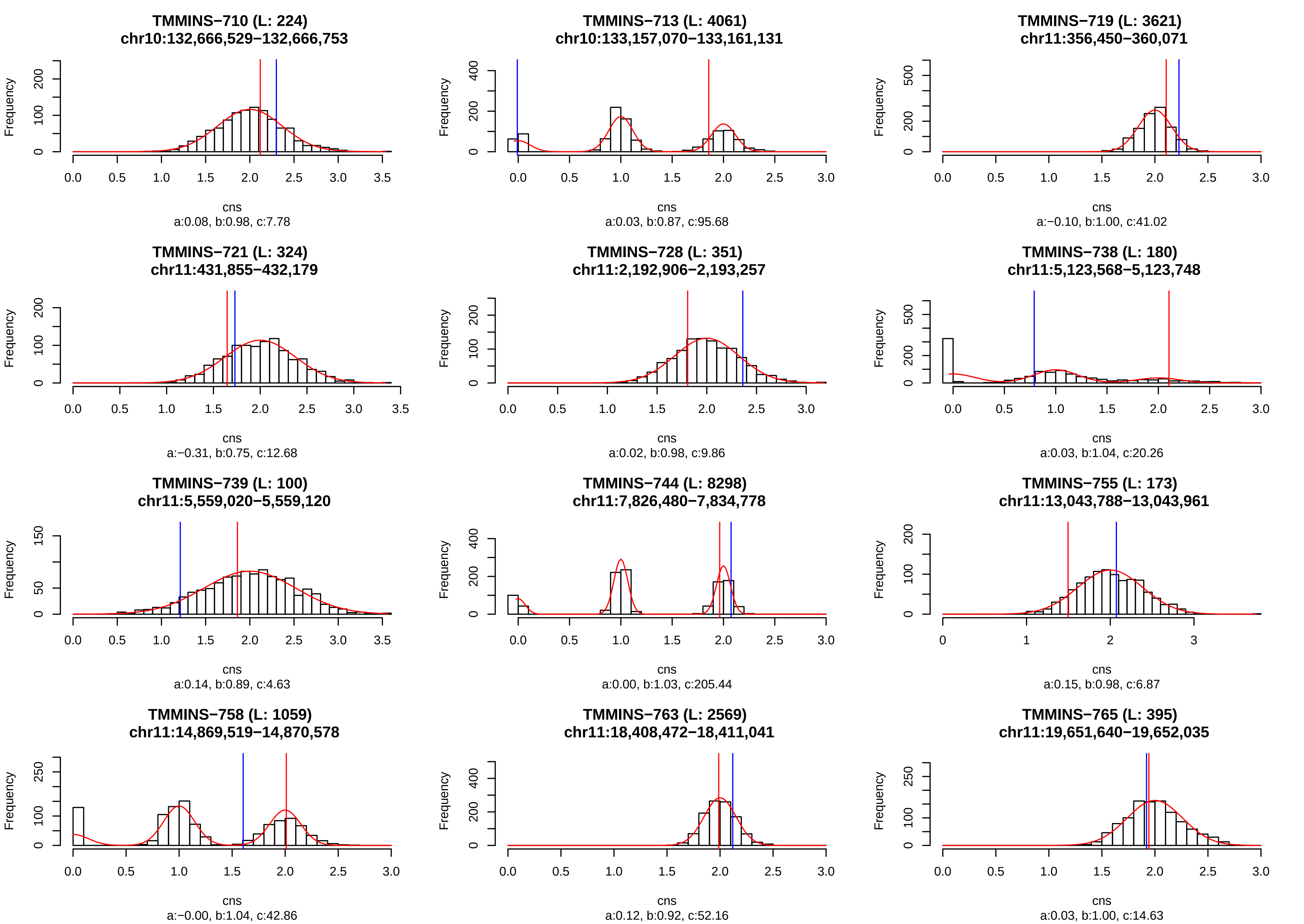
TMMINS−710 (L: 224)
chr10:132,666,529−132,666,753
Frequency
cns
a:0.08, b:0.98, c:7.78
TMMINS−713 (L: 4061)
chr10:133,157,070−133,161,131
Frequency
cns
a:0.03, b:0.87, c:95.68
TMMINS−719 (L: 3621)
chr11:356,450−360,071
Frequency
cns
a:−0.10, b:1.00, c:41.02
TMMINS−721 (L: 324)
chr11:431,855−432,179
Frequency
cns
a:−0.31, b:0.75, c:12.68
TMMINS−728 (L: 351)
chr11:2,192,906−2,193,257
Frequency
cns
a:0.02, b:0.98, c:9.86
TMMINS−738 (L: 180)
chr11:5,123,568−5,123,748
Frequency
cns
a:0.03, b:1.04, c:20.26
TMMINS−739 (L: 100)
chr11:5,559,020−5,559,120
Frequency
cns
a:0.14, b:0.89, c:4.63
TMMINS−744 (L: 8298)
chr11:7,826,480−7,834,778
Frequency
cns
a:0.00, b:1.03, c:205.44
TMMINS−755 (L: 173)
chr11:13,043,788−13,043,961
Frequency
cns
a:0.15, b:0.98, c:6.87
TMMINS−758 (L: 1059)
chr11:14,869,519−14,870,578
Frequency
cns
a:−0.00, b:1.04, c:42.86
TMMINS−763 (L: 2569)
chr11:18,408,472−18,411,041
Frequency
cns
a:0.12, b:0.92, c:52.16
TMMINS−765 (L: 395)
chr11:19,651,640−19,652,035
Frequency
cns
a:0.03, b:1.00, c:14.63

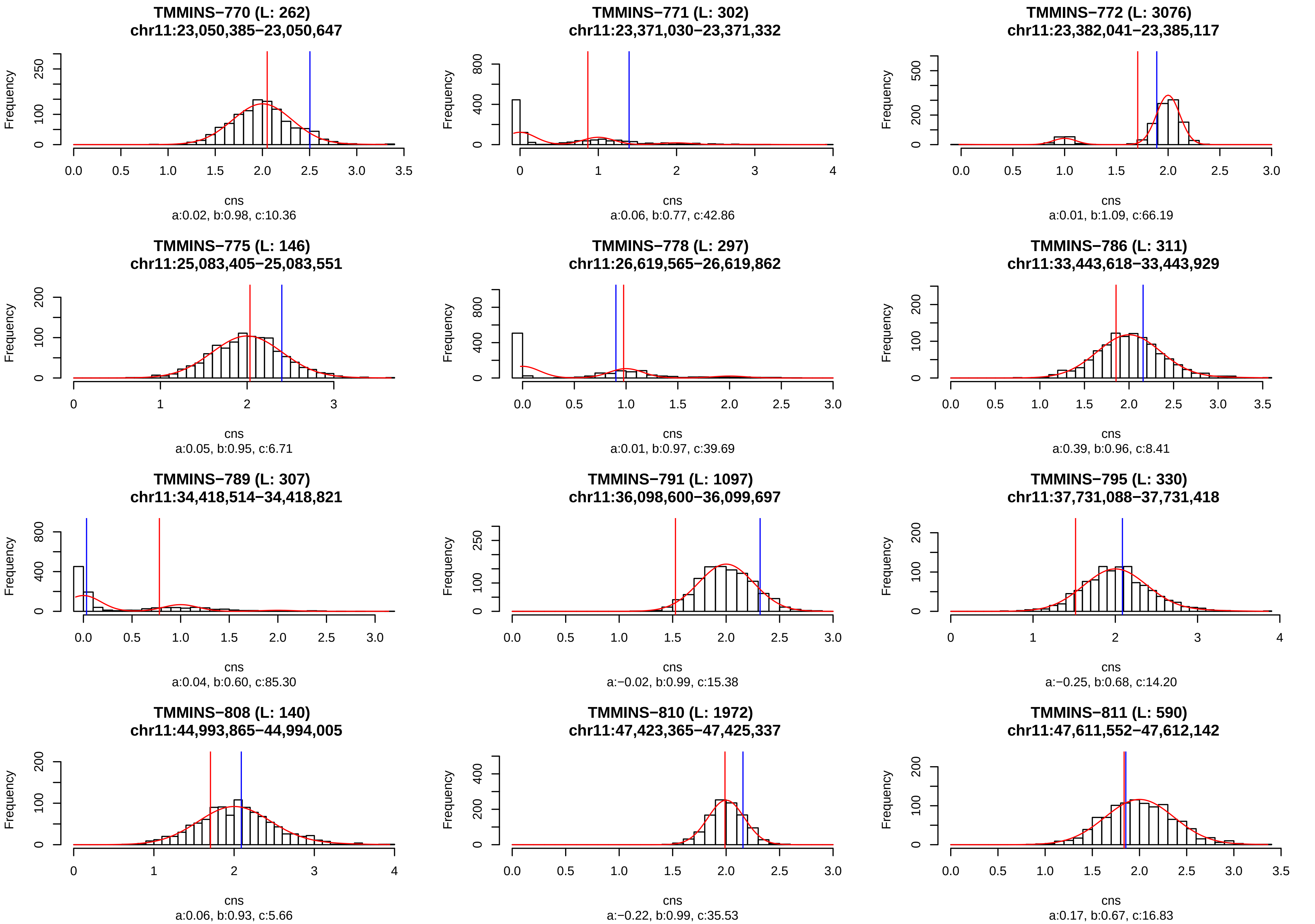
TMMINS−770 (L: 262)
chr11:23,050,385−23,050,647
Frequency
cns
a:0.02, b:0.98, c:10.36
TMMINS−771 (L: 302)
chr11:23,371,030−23,371,332
Frequency
cns
a:0.06, b:0.77, c:42.86
TMMINS−772 (L: 3076)
chr11:23,382,041−23,385,117
Frequency
cns
a:0.01, b:1.09, c:66.19
TMMINS−775 (L: 146)
chr11:25,083,405−25,083,551
Frequency
cns
a:0.05, b:0.95, c:6.71
TMMINS−778 (L: 297)
chr11:26,619,565−26,619,862
Frequency
cns
a:0.01, b:0.97, c:39.69
TMMINS−786 (L: 311)
chr11:33,443,618−33,443,929
Frequency
cns
a:0.39, b:0.96, c:8.41
TMMINS−789 (L: 307)
chr11:34,418,514−34,418,821
Frequency
cns
a:0.04, b:0.60, c:85.30
TMMINS−791 (L: 1097)
chr11:36,098,600−36,099,697
Frequency
cns
a:−0.02, b:0.99, c:15.38
TMMINS−795 (L: 330)
chr11:37,731,088−37,731,418
Frequency
cns
a:−0.25, b:0.68, c:14.20
TMMINS−808 (L: 140)
chr11:44,993,865−44,994,005
Frequency
cns
a:0.06, b:0.93, c:5.66
TMMINS−810 (L: 1972)
chr11:47,423,365−47,425,337
Frequency
cns
a:−0.22, b:0.99, c:35.53
TMMINS−811 (L: 590)
chr11:47,611,552−47,612,142
Frequency
cns
a:0.17, b:0.67, c:16.83

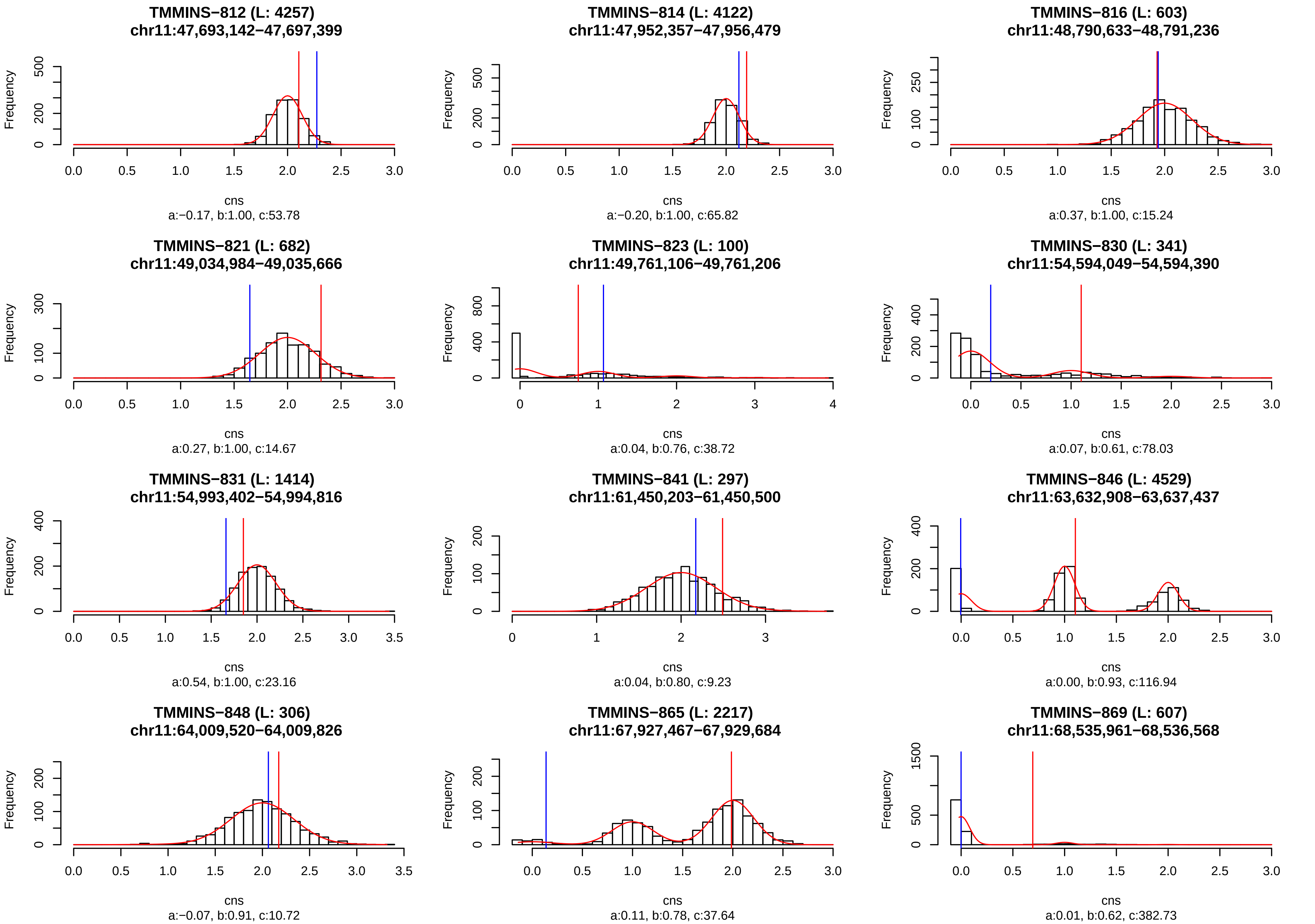
TMMINS−812 (L: 4257)
chr11:47,693,142−47,697,399
a:−0.17, b:1.00, c:53.78
TMMINS−814 (L: 4122)
chr11:47,952,357−47,956,479
a:−0.20, b:1.00, c:65.82
TMMINS−816 (L: 603)
chr11:48,790,633−48,791,236
a:0.37, b:1.00, c:15.24
TMMINS−821 (L: 682)
chr11:49,034,984−49,035,666
a:0.27, b:1.00, c:14.67
TMMINS−823 (L: 100)
chr11:49,761,106−49,761,206
a:0.04, b:0.76, c:38.72
TMMINS−830 (L: 341)
chr11:54,594,049−54,594,390
a:0.07, b:0.61, c:78.03
TMMINS−831 (L: 1414)
chr11:54,993,402−54,994,816
a:0.54, b:1.00, c:23.16
TMMINS−841 (L: 297)
chr11:61,450,203−61,450,500
a:0.04, b:0.80, c:9.23
TMMINS−846 (L: 4529)
chr11:63,632,908−63,637,437
a:0.00, b:0.93, c:116.94
TMMINS−848 (L: 306)
chr11:64,009,520−64,009,826
a:−0.07, b:0.91, c:10.72
TMMINS−865 (L: 2217)
chr11:67,927,467−67,929,684
a:0.11, b:0.78, c:37.64
TMMINS−869 (L: 607)
chr11:68,535,961−68,536,568
a:0.01, b:0.62, c:382.73
Frequency
cns

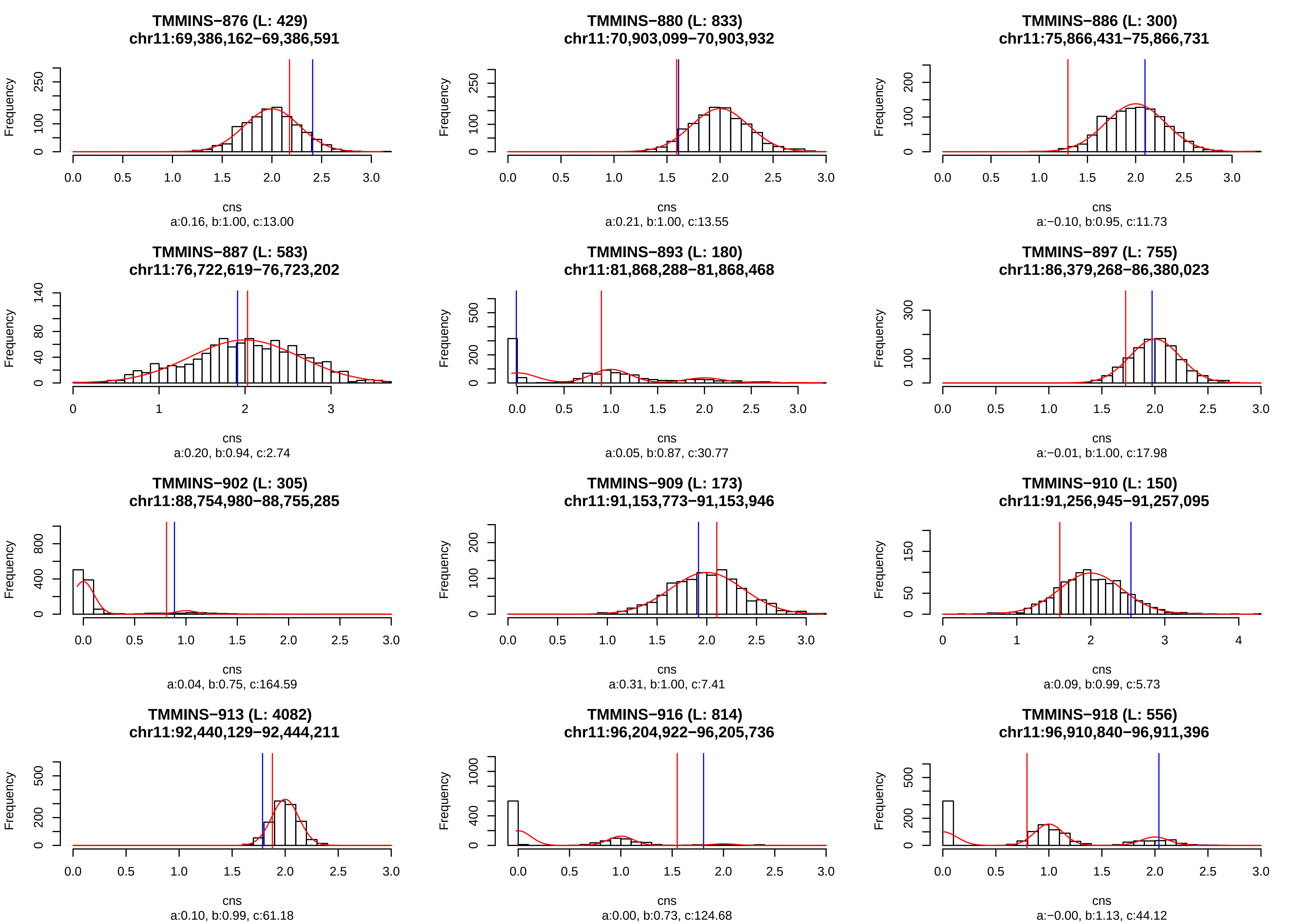

TMMINS−876 (L: 429)
chr11:69,386,162−69,386,591
Frequency
cns
a:0.16, b:1.00, c:13.00
TMMINS−880 (L: 833)
chr11:70,903,099−70,903,932
Frequency
cns
a:0.21, b:1.00, c:13.55
TMMINS−886 (L: 300)
chr11:75,866,431−75,866,731
Frequency
cns
a:−0.10, b:0.95, c:11.73
TMMINS−887 (L: 583)
chr11:76,722,619−76,723,202
Frequency
cns
a:0.20, b:0.94, c:2.74
TMMINS−893 (L: 180)
chr11:81,868,288−81,868,468
Frequency
cns
a:0.05, b:0.87, c:30.77
TMMINS−897 (L: 755)
chr11:86,379,268−86,380,023
Frequency
cns
a:−0.01, b:1.00, c:17.98
TMMINS−902 (L: 305)
chr11:88,754,980−88,755,285
Frequency
cns
a:0.04, b:0.75, c:164.59
TMMINS−909 (L: 173)
chr11:91,153,773−91,153,946
Frequency
cns
a:0.31, b:1.00, c:7.41
TMMINS−910 (L: 150)
chr11:91,256,945−91,257,095
Frequency
cns
a:0.09, b:0.99, c:5.73
TMMINS−913 (L: 4082)
chr11:92,440,129−92,444,211
Frequency
cns
a:0.10, b:0.99, c:61.18
TMMINS−916 (L: 814)
chr11:96,204,922−96,205,736
Frequency
cns
a:0.00, b:0.73, c:124.68
TMMINS−918 (L: 556)
chr11:96,910,840−96,911,396
Frequency
cns
a:−0.00, b:1.13, c:44.12

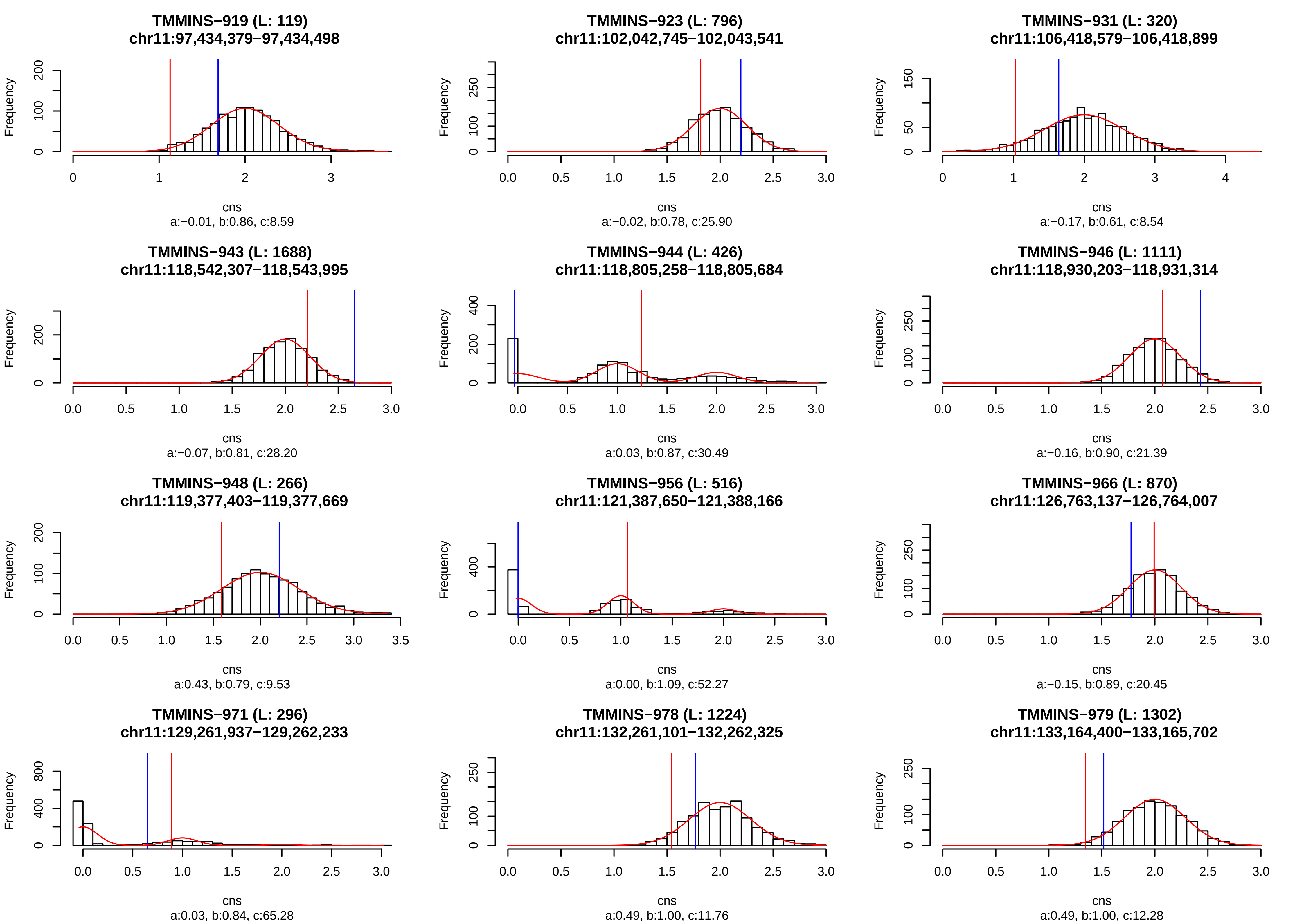
TMMINS−919 (L: 119)
chr11:97,434,379−97,434,498
Frequency
cns
a:−0.01, b:0.86, c:8.59
TMMINS−923 (L: 796)
chr11:102,042,745−102,043,541
Frequency
cns
a:−0.02, b:0.78, c:25.90
TMMINS−931 (L: 320)
chr11:106,418,579−106,418,899
Frequency
cns
a:−0.17, b:0.61, c:8.54
TMMINS−943 (L: 1688)
chr11:118,542,307−118,543,995
Frequency
cns
a:−0.07, b:0.81, c:28.20
TMMINS−944 (L: 426)
chr11:118,805,258−118,805,684
Frequency
cns
a:0.03, b:0.87, c:30.49
TMMINS−946 (L: 1111)
chr11:118,930,203−118,931,314
Frequency
cns
a:−0.16, b:0.90, c:21.39
TMMINS−948 (L: 266)
chr11:119,377,403−119,377,669
Frequency
cns
a:0.43, b:0.79, c:9.53
TMMINS−956 (L: 516)
chr11:121,387,650−121,388,166
Frequency
cns
a:0.00, b:1.09, c:52.27
TMMINS−966 (L: 870)
chr11:126,763,137−126,764,007
Frequency
cns
a:−0.15, b:0.89, c:20.45
TMMINS−971 (L: 296)
chr11:129,261,937−129,262,233
Frequency
cns
a:0.03, b:0.84, c:65.28
TMMINS−978 (L: 1224)
chr11:132,261,101−132,262,325
Frequency
cns
a:0.49, b:1.00, c:11.76
TMMINS−979 (L: 1302)
chr11:133,164,400−133,165,702
Frequency
cns
a:0.49, b:1.00, c:12.28

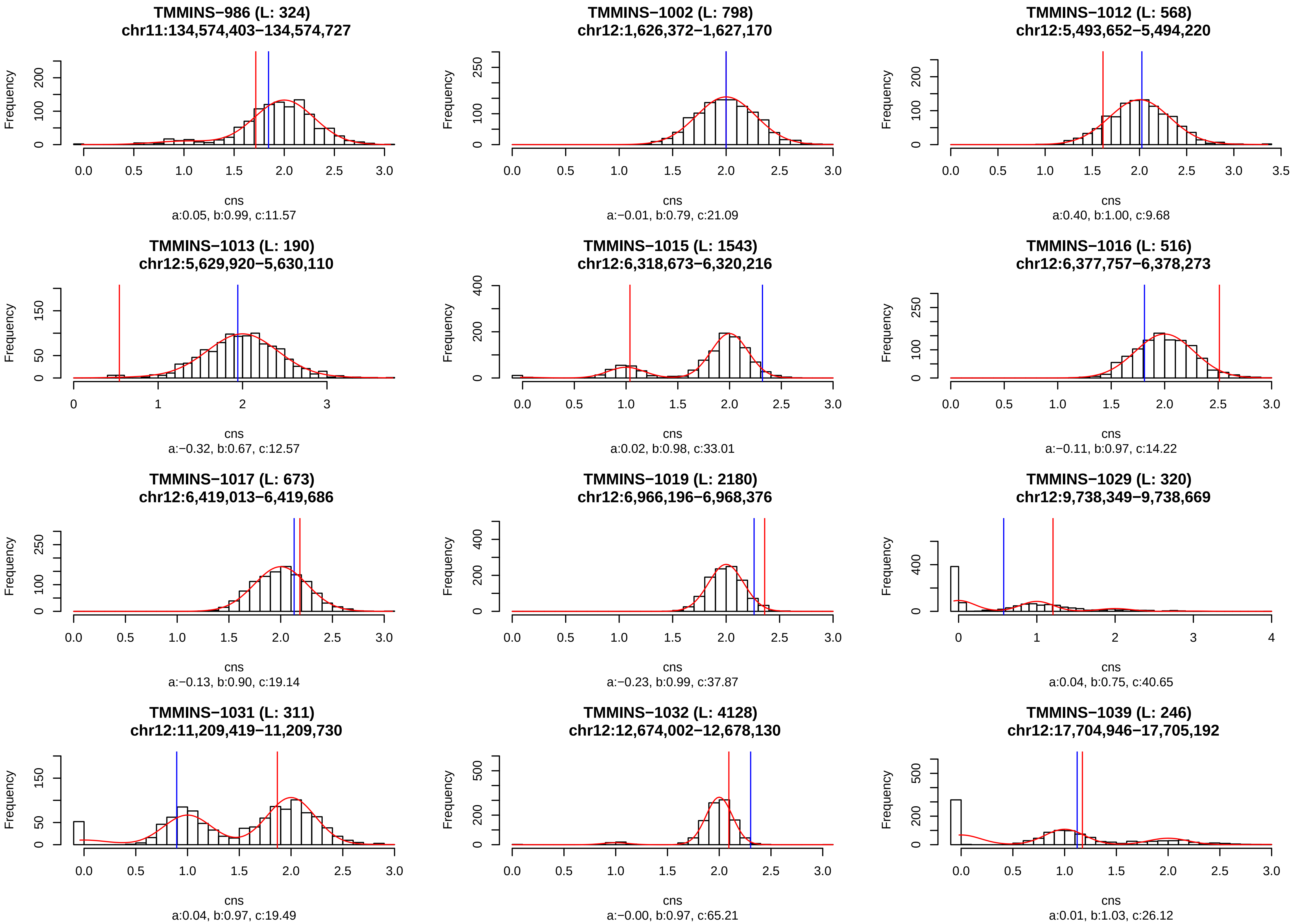
TMMINS−986 (L: 324)
chr11:134,574,403−134,574,727
a:0.05, b:0.99, c:11.57
TMMINS−1002 (L: 798)
chr12:1,626,372−1,627,170
a:−0.01, b:0.79, c:21.09
TMMINS−1012 (L: 568)
chr12:5,493,652−5,494,220
a:0.40, b:1.00, c:9.68
TMMINS−1013 (L: 190)
chr12:5,629,920−5,630,110
a:−0.32, b:0.67, c:12.57
TMMINS−1015 (L: 1543)
chr12:6,318,673−6,320,216
a:0.02, b:0.98, c:33.01
TMMINS−1016 (L: 516)
chr12:6,377,757−6,378,273
a:−0.11, b:0.97, c:14.22
TMMINS−1017 (L: 673)
chr12:6,419,013−6,419,686
a:−0.13, b:0.90, c:19.14
TMMINS−1019 (L: 2180)
chr12:6,966,196−6,968,376
a:−0.23, b:0.99, c:37.87
TMMINS−1029 (L: 320)
chr12:9,738,349−9,738,669
a:0.04, b:0.75, c:40.65
TMMINS−1031 (L: 311)
chr12:11,209,419−11,209,730
a:0.04, b:0.97, c:19.49
TMMINS−1032 (L: 4128)
chr12:12,674,002−12,678,130
a:−0.00, b:0.97, c:65.21
TMMINS−1039 (L: 246)
chr12:17,704,946−17,705,192
a:0.01, b:1.03, c:26.12
Frequency
cns

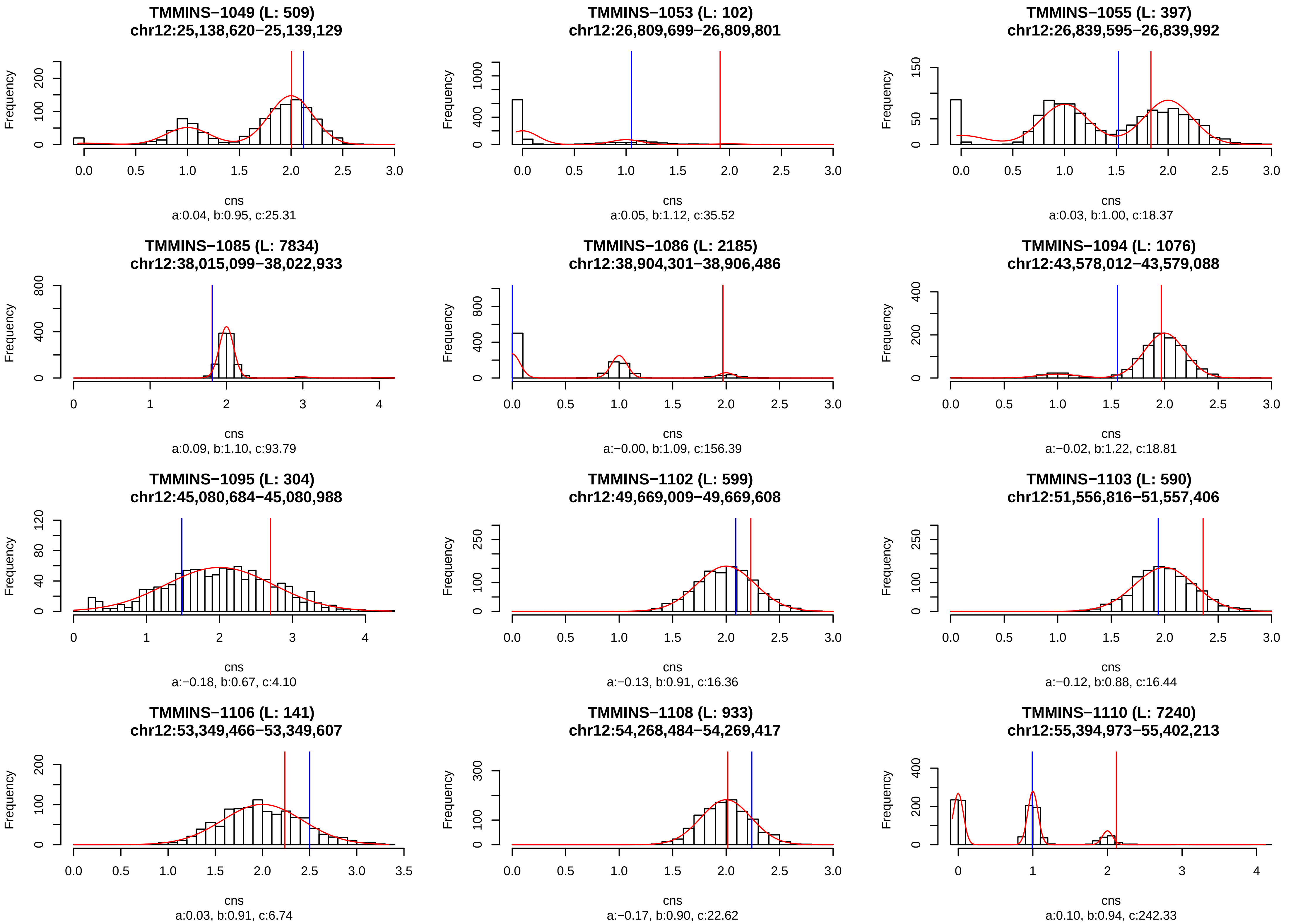
TMMINS−1049 (L: 509)
chr12:25,138,620−25,139,129
Frequency
cns
a:0.04, b:0.95, c:25.31
TMMINS−1053 (L: 102)
chr12:26,809,699−26,809,801
Frequency
cns
a:0.05, b:1.12, c:35.52
TMMINS−1055 (L: 397)
chr12:26,839,595−26,839,992
Frequency
cns
a:0.03, b:1.00, c:18.37
TMMINS−1085 (L: 7834)
chr12:38,015,099−38,022,933
Frequency
cns
a:0.09, b:1.10, c:93.79
TMMINS−1086 (L: 2185)
chr12:38,904,301−38,906,486
Frequency
cns
a:−0.00, b:1.09, c:156.39
TMMINS−1094 (L: 1076)
chr12:43,578,012−43,579,088
Frequency
cns
a:−0.02, b:1.22, c:18.81
TMMINS−1095 (L: 304)
chr12:45,080,684−45,080,988
Frequency
cns
a:−0.18, b:0.67, c:4.10
TMMINS−1102 (L: 599)
chr12:49,669,009−49,669,608
Frequency
cns
a:−0.13, b:0.91, c:16.36
TMMINS−1103 (L: 590)
chr12:51,556,816−51,557,406
Frequency
cns
a:−0.12, b:0.88, c:16.44
TMMINS−1106 (L: 141)
chr12:53,349,466−53,349,607
Frequency
cns
a:0.03, b:0.91, c:6.74
TMMINS−1108 (L: 933)
chr12:54,268,484−54,269,417
Frequency
cns
a:−0.17, b:0.90, c:22.62
TMMINS−1110 (L: 7240)
chr12:55,394,973−55,402,213
Frequency
cns
a:0.10, b:0.94, c:242.33

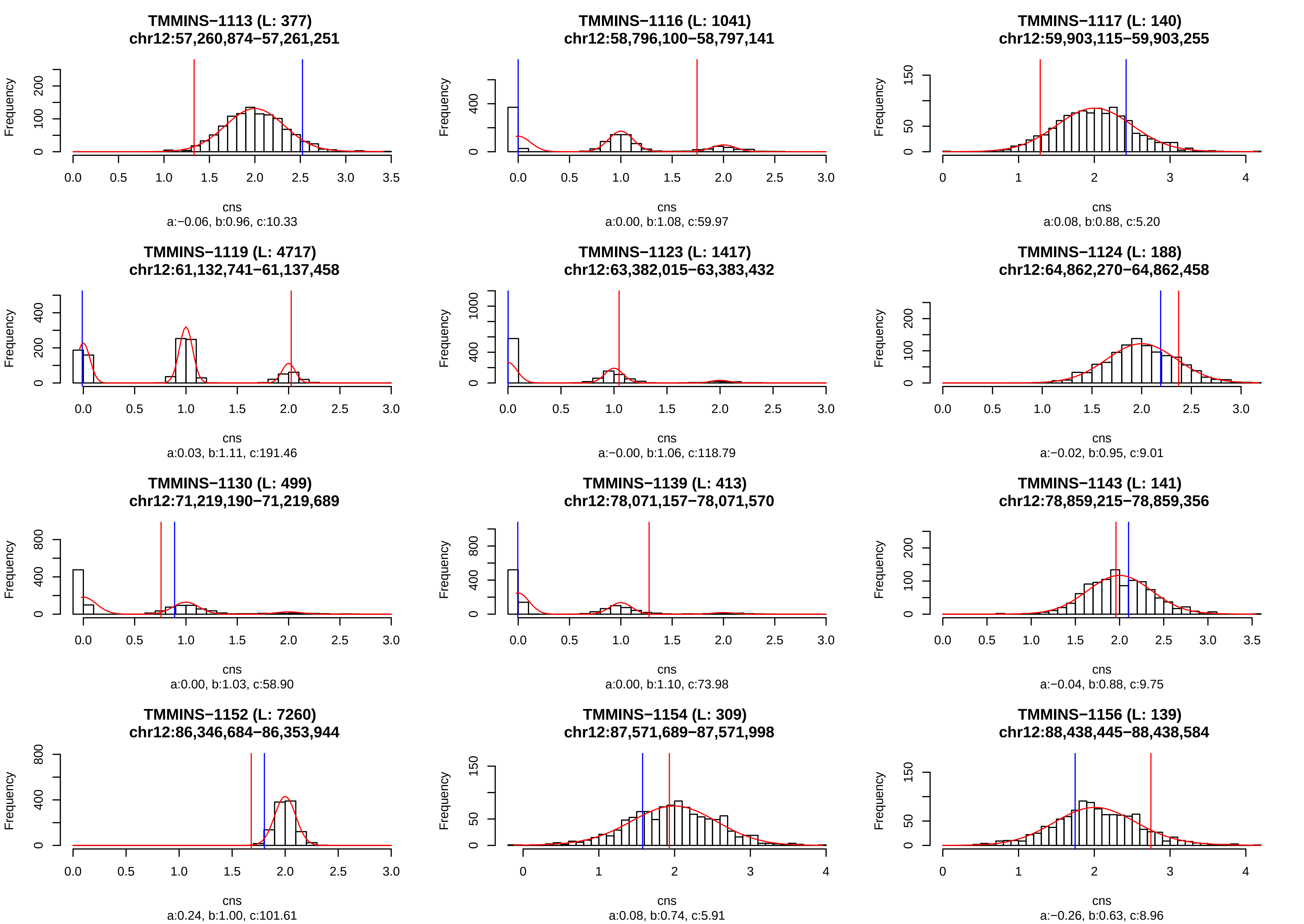
TMMINS−1113 (L: 377)
chr12:57,260,874−57,261,251
Frequency
cns
a:−0.06, b:0.96, c:10.33
TMMINS−1116 (L: 1041)
chr12:58,796,100−58,797,141
Frequency
cns
a:0.00, b:1.08, c:59.97
TMMINS−1117 (L: 140)
chr12:59,903,115−59,903,255
Frequency
cns
a:0.08, b:0.88, c:5.20
TMMINS−1119 (L: 4717)
chr12:61,132,741−61,137,458
Frequency
cns
a:0.03, b:1.11, c:191.46
TMMINS−1123 (L: 1417)
chr12:63,382,015−63,383,432
Frequency
cns
a:−0.00, b:1.06, c:118.79
TMMINS−1124 (L: 188)
chr12:64,862,270−64,862,458
Frequency
cns
a:−0.02, b:0.95, c:9.01
TMMINS−1130 (L: 499)
chr12:71,219,190−71,219,689
Frequency
cns
a:0.00, b:1.03, c:58.90
TMMINS−1139 (L: 413)
chr12:78,071,157−78,071,570
Frequency
cns
a:0.00, b:1.10, c:73.98
TMMINS−1143 (L: 141)
chr12:78,859,215−78,859,356
Frequency
cns
a:−0.04, b:0.88, c:9.75
TMMINS−1152 (L: 7260)
chr12:86,346,684−86,353,944
Frequency
cns
a:0.24, b:1.00, c:101.61
TMMINS−1154 (L: 309)
chr12:87,571,689−87,571,998
Frequency
cns
a:0.08, b:0.74, c:5.91
TMMINS−1156 (L: 139)
chr12:88,438,445−88,438,584
Frequency
cns
a:−0.26, b:0.63, c:8.96

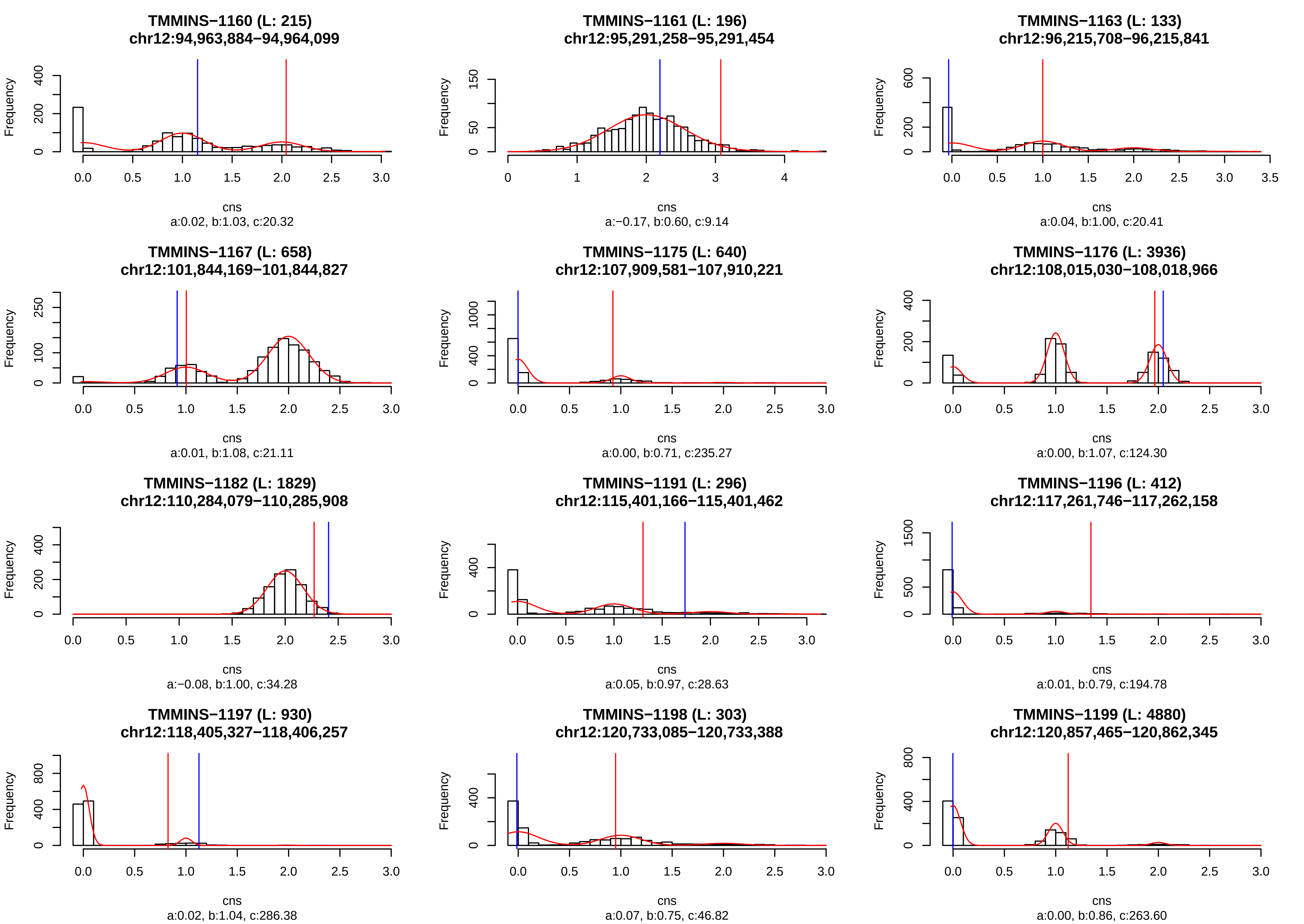
TMMINS−1160 (L: 215)
chr12:94,963,884−94,964,099
Frequency
cns
a:0.02, b:1.03, c:20.32
TMMINS−1161 (L: 196)
chr12:95,291,258−95,291,454
Frequency
cns
a:−0.17, b:0.60, c:9.14
TMMINS−1163 (L: 133)
chr12:96,215,708−96,215,841
Frequency
cns
a:0.04, b:1.00, c:20.41
TMMINS−1167 (L: 658)
chr12:101,844,169−101,844,827
Frequency
cns
a:0.01, b:1.08, c:21.11
TMMINS−1175 (L: 640)
chr12:107,909,581−107,910,221
Frequency
cns
a:0.00, b:0.71, c:235.27
TMMINS−1176 (L: 3936)
chr12:108,015,030−108,018,966
Frequency
cns
a:0.00, b:1.07, c:124.30
TMMINS−1182 (L: 1829)
chr12:110,284,079−110,285,908
Frequency
cns
a:−0.08, b:1.00, c:34.28
TMMINS−1191 (L: 296)
chr12:115,401,166−115,401,462
Frequency
cns
a:0.05, b:0.97, c:28.63
TMMINS−1196 (L: 412)
chr12:117,261,746−117,262,158
Frequency
cns
a:0.01, b:0.79, c:194.78
TMMINS−1197 (L: 930)
chr12:118,405,327−118,406,257
Frequency
cns
a:0.02, b:1.04, c:286.38
TMMINS−1198 (L: 303)
chr12:120,733,085−120,733,388
Frequency
cns
a:0.07, b:0.75, c:46.82
TMMINS−1199 (L: 4880)
chr12:120,857,465−120,862,345
Frequency
cns
a:0.00, b:0.86, c:263.60

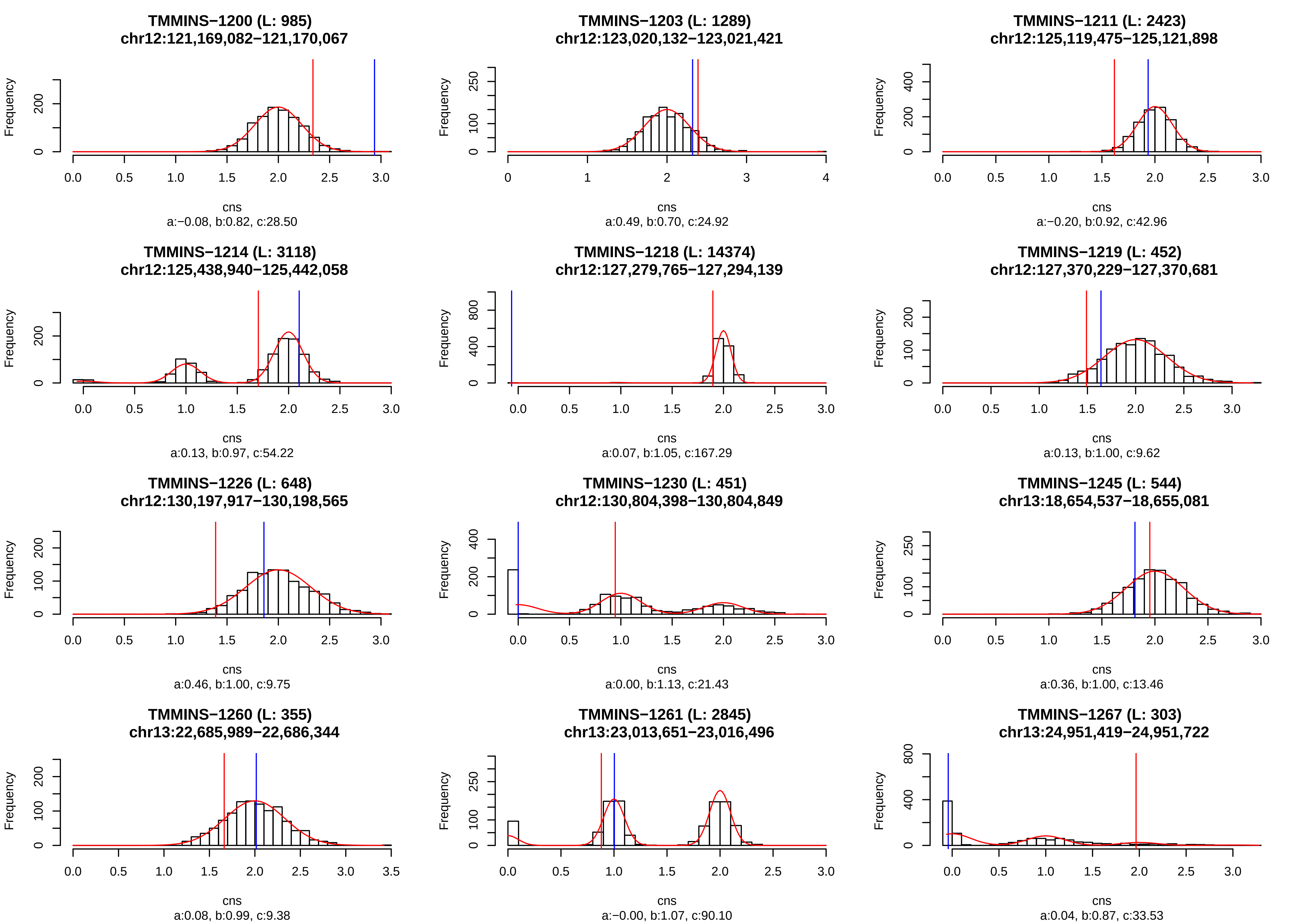
TMMINS−1200 (L: 985)
chr12:121,169,082−121,170,067
Frequency
cns
a:−0.08, b:0.82, c:28.50
TMMINS−1203 (L: 1289)
chr12:123,020,132−123,021,421
Frequency
cns
a:0.49, b:0.70, c:24.92
TMMINS−1211 (L: 2423)
chr12:125,119,475−125,121,898
Frequency
cns
a:−0.20, b:0.92, c:42.96
TMMINS−1214 (L: 3118)
chr12:125,438,940−125,442,058
Frequency
cns
a:0.13, b:0.97, c:54.22
TMMINS−1218 (L: 14374)
chr12:127,279,765−127,294,139
Frequency
cns
a:0.07, b:1.05, c:167.29
TMMINS−1219 (L: 452)
chr12:127,370,229−127,370,681
Frequency
cns
a:0.13, b:1.00, c:9.62
TMMINS−1226 (L: 648)
chr12:130,197,917−130,198,565
Frequency
cns
a:0.46, b:1.00, c:9.75
TMMINS−1230 (L: 451)
chr12:130,804,398−130,804,849
Frequency
cns
a:0.00, b:1.13, c:21.43
TMMINS−1245 (L: 544)
chr13:18,654,537−18,655,081
Frequency
cns
a:0.36, b:1.00, c:13.46
TMMINS−1260 (L: 355)
chr13:22,685,989−22,686,344
Frequency
cns
a:0.08, b:0.99, c:9.38
TMMINS−1261 (L: 2845)
chr13:23,013,651−23,016,496
Frequency
cns
a:−0.00, b:1.07, c:90.10
TMMINS−1267 (L: 303)
chr13:24,951,419−24,951,722
Frequency
cns
a:0.04, b:0.87, c:33.53

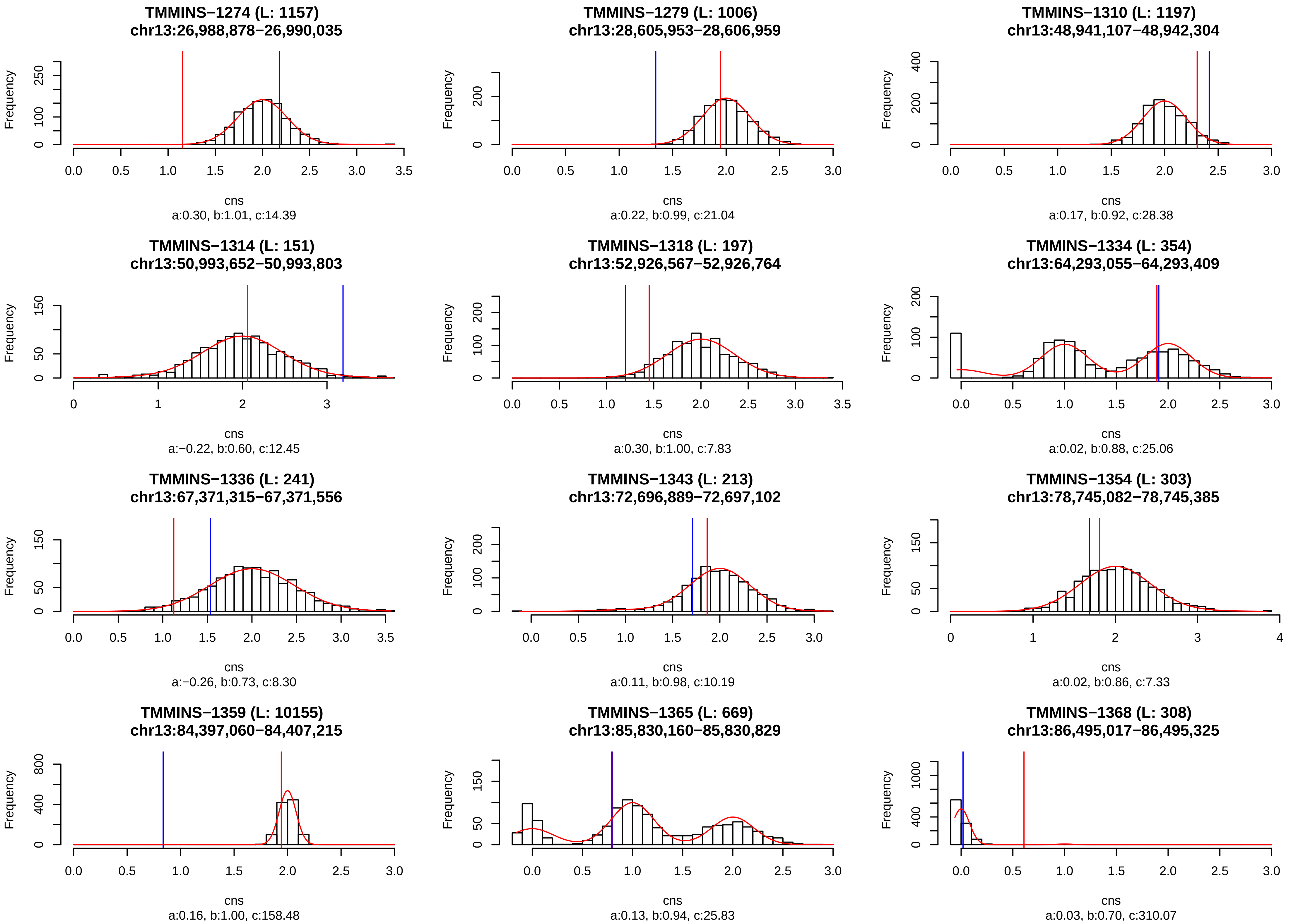
TMMINS−1274 (L: 1157)
chr13:26,988,878−26,990,035
Frequency
cns
a:0.30, b:1.01, c:14.39
TMMINS−1279 (L: 1006)
chr13:28,605,953−28,606,959
Frequency
cns
a:0.22, b:0.99, c:21.04
TMMINS−1310 (L: 1197)
chr13:48,941,107−48,942,304
Frequency
cns
a:0.17, b:0.92, c:28.38
TMMINS−1314 (L: 151)
chr13:50,993,652−50,993,803
Frequency
cns
a:−0.22, b:0.60, c:12.45
TMMINS−1318 (L: 197)
chr13:52,926,567−52,926,764
Frequency
cns
a:0.30, b:1.00, c:7.83
TMMINS−1334 (L: 354)
chr13:64,293,055−64,293,409
Frequency
cns
a:0.02, b:0.88, c:25.06
TMMINS−1336 (L: 241)
chr13:67,371,315−67,371,556
Frequency
cns
a:−0.26, b:0.73, c:8.30
TMMINS−1343 (L: 213)
chr13:72,696,889−72,697,102
Frequency
cns
a:0.11, b:0.98, c:10.19
TMMINS−1354 (L: 303)
chr13:78,745,082−78,745,385
Frequency
cns
a:0.02, b:0.86, c:7.33
TMMINS−1359 (L: 10155)
chr13:84,397,060−84,407,215
Frequency
cns
a:0.16, b:1.00, c:158.48
TMMINS−1365 (L: 669)
chr13:85,830,160−85,830,829
Frequency
cns
a:0.13, b:0.94, c:25.83
TMMINS−1368 (L: 308)
chr13:86,495,017−86,495,325
Frequency
cns
a:0.03, b:0.70, c:310.07

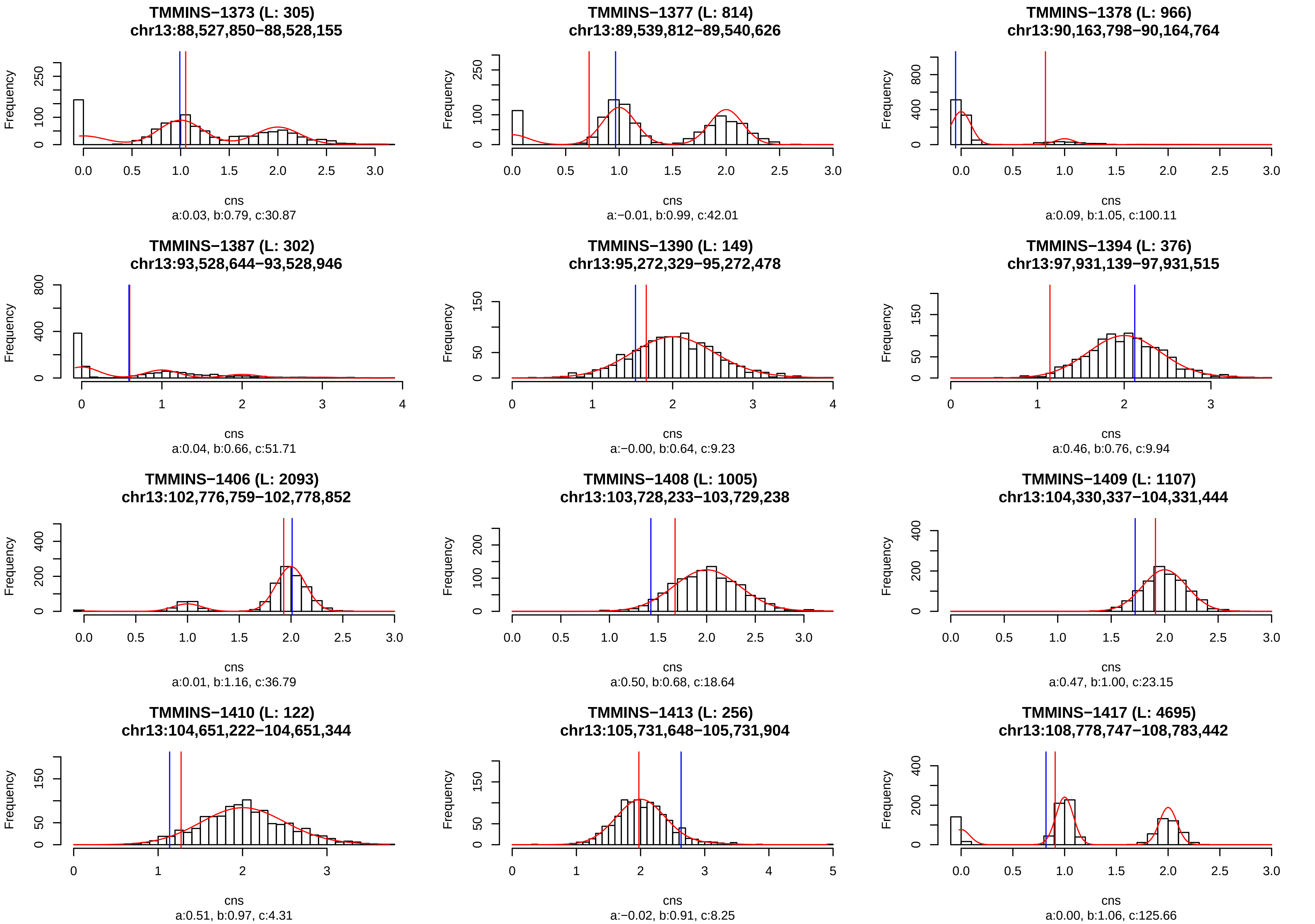
TMMINS−1373 (L: 305)
chr13:88,527,850−88,528,155
Frequency
cns
a:0.03, b:0.79, c:30.87
TMMINS−1377 (L: 814)
chr13:89,539,812−89,540,626
Frequency
cns
a:−0.01, b:0.99, c:42.01
TMMINS−1378 (L: 966)
chr13:90,163,798−90,164,764
Frequency
cns
a:0.09, b:1.05, c:100.11
TMMINS−1387 (L: 302)
chr13:93,528,644−93,528,946
Frequency
cns
a:0.04, b:0.66, c:51.71
TMMINS−1390 (L: 149)
chr13:95,272,329−95,272,478
Frequency
cns
a:−0.00, b:0.64, c:9.23
TMMINS−1394 (L: 376)
chr13:97,931,139−97,931,515
Frequency
cns
a:0.46, b:0.76, c:9.94
TMMINS−1406 (L: 2093)
chr13:102,776,759−102,778,852
Frequency
cns
a:0.01, b:1.16, c:36.79
TMMINS−1408 (L: 1005)
chr13:103,728,233−103,729,238
Frequency
cns
a:0.50, b:0.68, c:18.64
TMMINS−1409 (L: 1107)
chr13:104,330,337−104,331,444
Frequency
cns
a:0.47, b:1.00, c:23.15
TMMINS−1410 (L: 122)
chr13:104,651,222−104,651,344
Frequency
cns
a:0.51, b:0.97, c:4.31
TMMINS−1413 (L: 256)
chr13:105,731,648−105,731,904
Frequency
cns
a:−0.02, b:0.91, c:8.25
TMMINS−1417 (L: 4695)
chr13:108,778,747−108,783,442
Frequency
cns
a:0.00, b:1.06, c:125.66

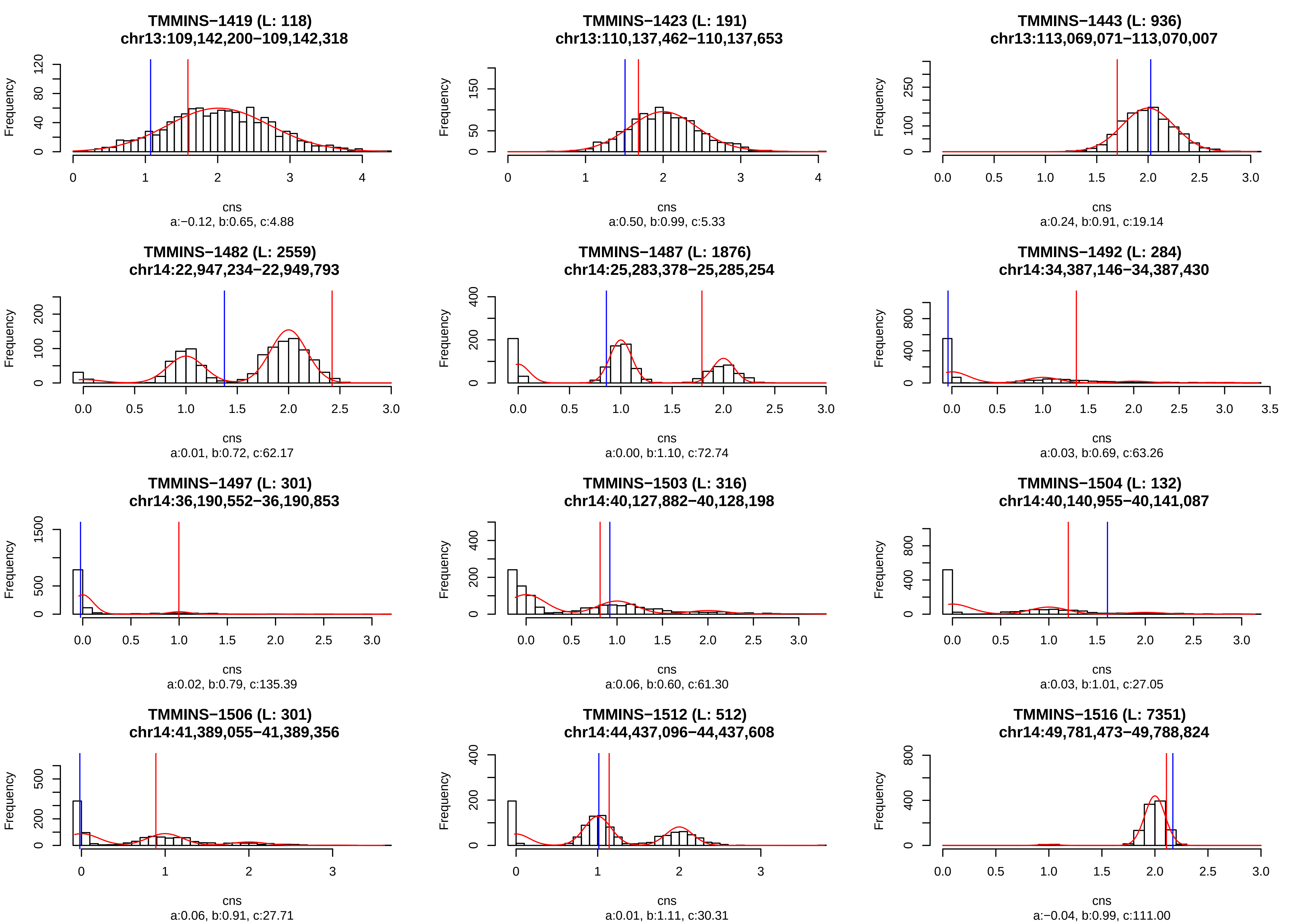
TMMINS−1419 (L: 118)
chr13:109,142,200−109,142,318
Frequency
cns
a:−0.12, b:0.65, c:4.88
TMMINS−1423 (L: 191)
chr13:110,137,462−110,137,653
Frequency
cns
a:0.50, b:0.99, c:5.33
TMMINS−1443 (L: 936)
chr13:113,069,071−113,070,007
Frequency
cns
a:0.24, b:0.91, c:19.14
TMMINS−1482 (L: 2559)
chr14:22,947,234−22,949,793
Frequency
cns
a:0.01, b:0.72, c:62.17
TMMINS−1487 (L: 1876)
chr14:25,283,378−25,285,254
Frequency
cns
a:0.00, b:1.10, c:72.74
TMMINS−1492 (L: 284)
chr14:34,387,146−34,387,430
Frequency
cns
a:0.03, b:0.69, c:63.26
TMMINS−1497 (L: 301)
chr14:36,190,552−36,190,853
Frequency
cns
a:0.02, b:0.79, c:135.39
TMMINS−1503 (L: 316)
chr14:40,127,882−40,128,198
Frequency
cns
a:0.06, b:0.60, c:61.30
TMMINS−1504 (L: 132)
chr14:40,140,955−40,141,087
Frequency
cns
a:0.03, b:1.01, c:27.05
TMMINS−1506 (L: 301)
chr14:41,389,055−41,389,356
Frequency
cns
a:0.06, b:0.91, c:27.71
TMMINS−1512 (L: 512)
chr14:44,437,096−44,437,608
Frequency
cns
a:0.01, b:1.11, c:30.31
TMMINS−1516 (L: 7351)
chr14:49,781,473−49,788,824
Frequency
cns
a:−0.04, b:0.99, c:111.00

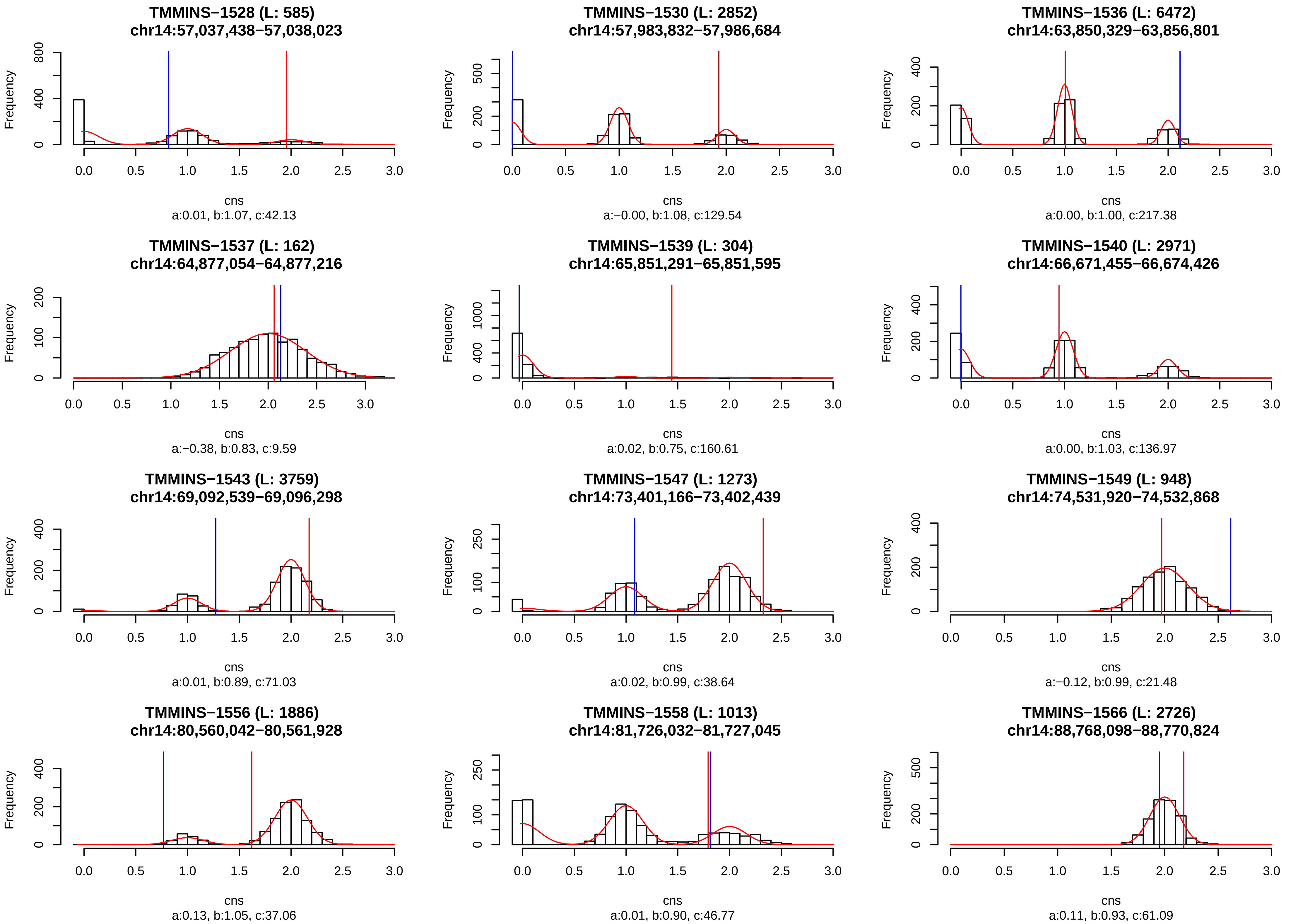

TMMINS−1528 (L: 585)
chr14:57,037,438−57,038,023
Frequency
cns
a:0.01, b:1.07, c:42.13
TMMINS−1530 (L: 2852)
chr14:57,983,832−57,986,684
Frequency
cns
a:−0.00, b:1.08, c:129.54
TMMINS−1536 (L: 6472)
chr14:63,850,329−63,856,801
Frequency
cns
a:0.00, b:1.00, c:217.38
TMMINS−1537 (L: 162)
chr14:64,877,054−64,877,216
Frequency
cns
a:−0.38, b:0.83, c:9.59
TMMINS−1539 (L: 304)
chr14:65,851,291−65,851,595
Frequency
cns
a:0.02, b:0.75, c:160.61
TMMINS−1540 (L: 2971)
chr14:66,671,455−66,674,426
Frequency
cns
a:0.00, b:1.03, c:136.97
TMMINS−1543 (L: 3759)
chr14:69,092,539−69,096,298
Frequency
cns
a:0.01, b:0.89, c:71.03
TMMINS−1547 (L: 1273)
chr14:73,401,166−73,402,439
Frequency
cns
a:0.02, b:0.99, c:38.64
TMMINS−1549 (L: 948)
chr14:74,531,920−74,532,868
Frequency
cns
a:−0.12, b:0.99, c:21.48
TMMINS−1556 (L: 1886)
chr14:80,560,042−80,561,928
Frequency
cns
a:0.13, b:1.05, c:37.06
TMMINS−1558 (L: 1013)
chr14:81,726,032−81,727,045
Frequency
cns
a:0.01, b:0.90, c:46.77
TMMINS−1566 (L: 2726)
chr14:88,768,098−88,770,824
Frequency
cns
a:0.11, b:0.93, c:61.09

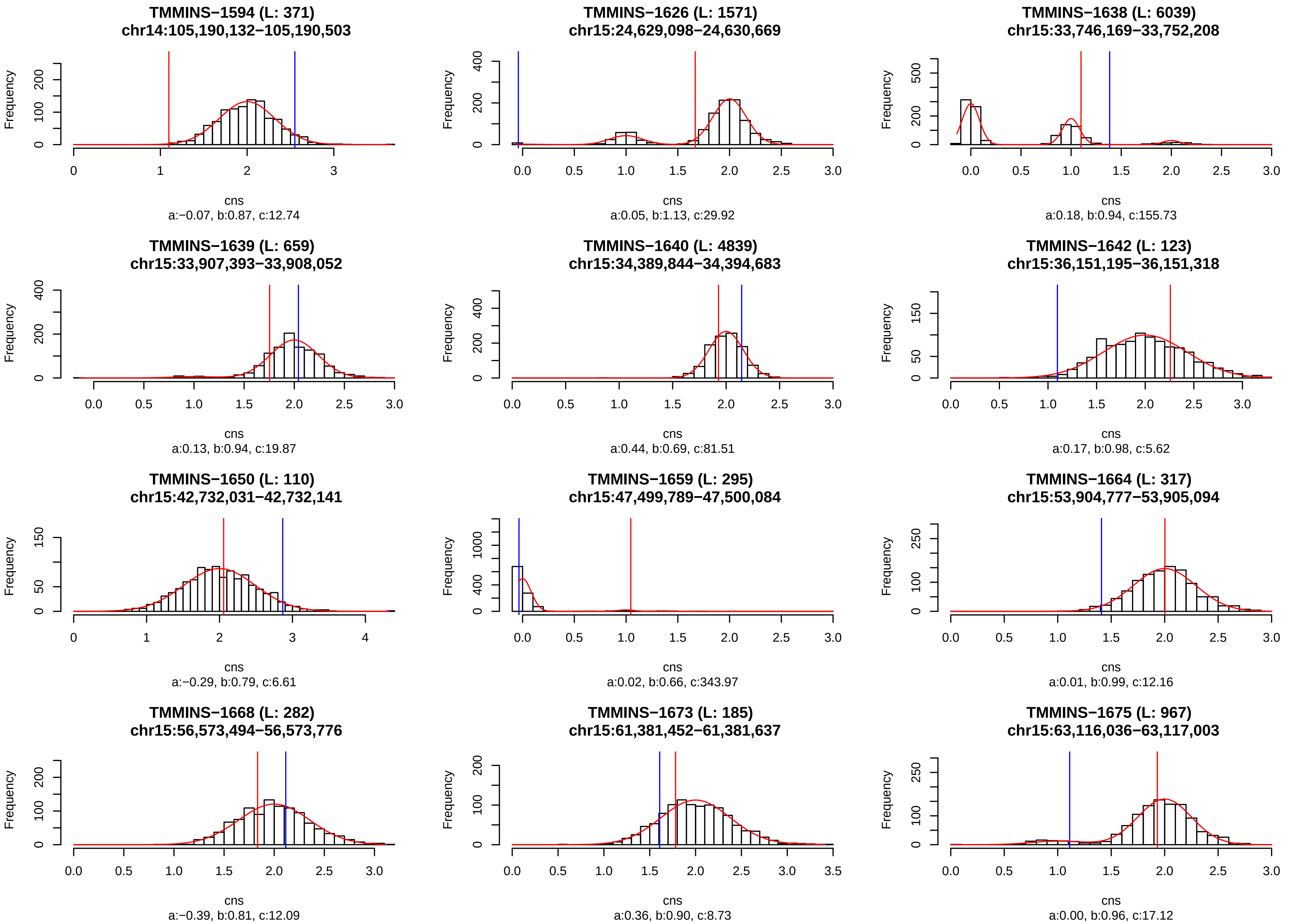
TMMINS−1594 (L: 371)
chr14:105,190,132−105,190,503
Frequency
cns
a:−0.07, b:0.87, c:12.74
TMMINS−1626 (L: 1571)
chr15:24,629,098−24,630,669
Frequency
cns
a:0.05, b:1.13, c:29.92
TMMINS−1638 (L: 6039)
chr15:33,746,169−33,752,208
Frequency
cns
a:0.18, b:0.94, c:155.73
TMMINS−1639 (L: 659)
chr15:33,907,393−33,908,052
Frequency
cns
a:0.13, b:0.94, c:19.87
TMMINS−1640 (L: 4839)
chr15:34,389,844−34,394,683
Frequency
cns
a:0.44, b:0.69, c:81.51
TMMINS−1642 (L: 123)
chr15:36,151,195−36,151,318
Frequency
cns
a:0.17, b:0.98, c:5.62
TMMINS−1650 (L: 110)
chr15:42,732,031−42,732,141
Frequency
cns
a:−0.29, b:0.79, c:6.61
TMMINS−1659 (L: 295)
chr15:47,499,789−47,500,084
Frequency
cns
a:0.02, b:0.66, c:343.97
TMMINS−1664 (L: 317)
chr15:53,904,777−53,905,094
Frequency
cns
a:0.01, b:0.99, c:12.16
TMMINS−1668 (L: 282)
chr15:56,573,494−56,573,776
Frequency
cns
a:−0.39, b:0.81, c:12.09
TMMINS−1673 (L: 185)
chr15:61,381,452−61,381,637
Frequency
cns
a:0.36, b:0.90, c:8.73
TMMINS−1675 (L: 967)
chr15:63,116,036−63,117,003
Frequency
cns
a:0.00, b:0.96, c:17.12

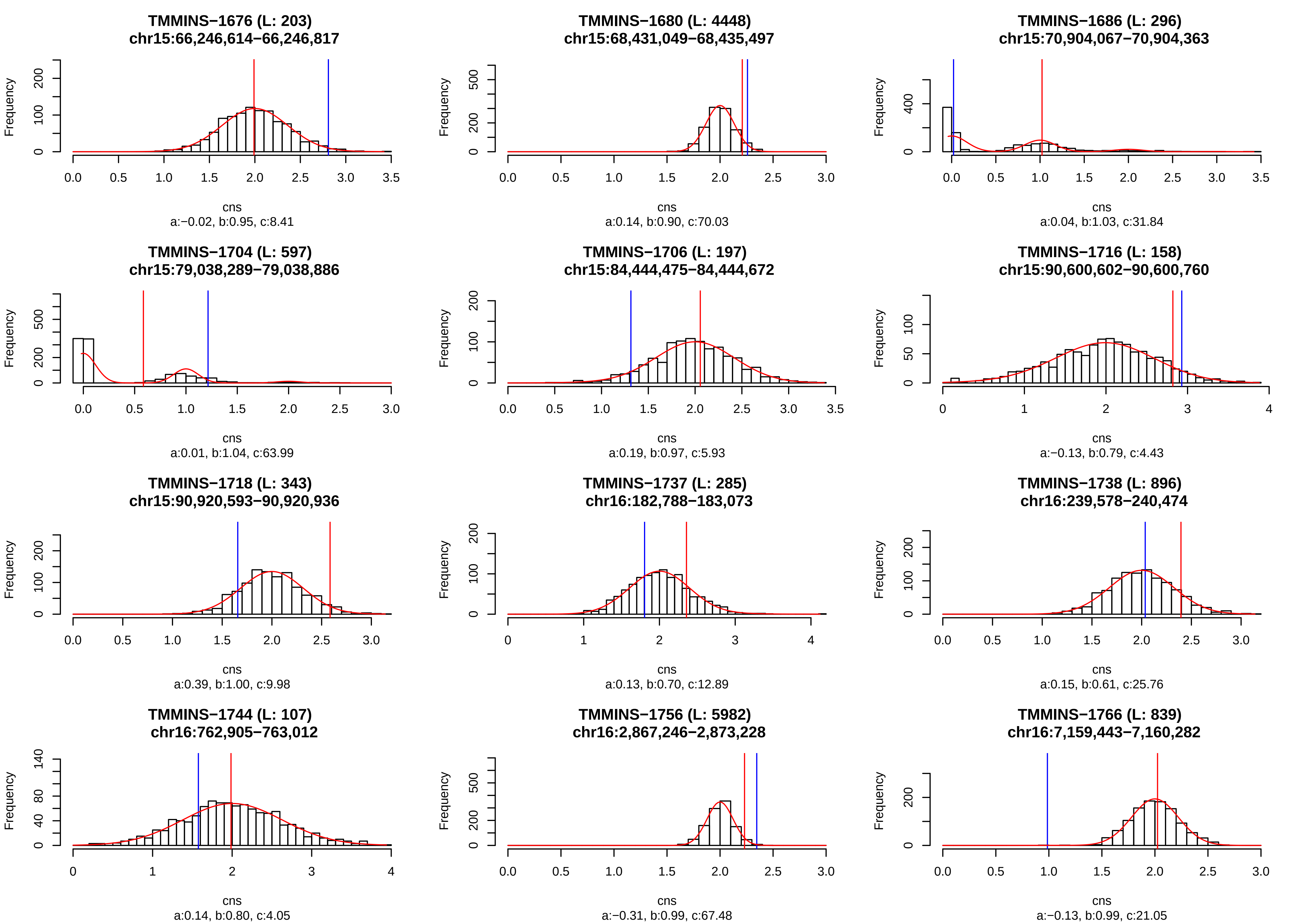
TMMINS−1676 (L: 203)
chr15:66,246,614−66,246,817
Frequency
cns
a:−0.02, b:0.95, c:8.41
TMMINS−1680 (L: 4448)
chr15:68,431,049−68,435,497
Frequency
cns
a:0.14, b:0.90, c:70.03
TMMINS−1686 (L: 296)
chr15:70,904,067−70,904,363
Frequency
cns
a:0.04, b:1.03, c:31.84
TMMINS−1704 (L: 597)
chr15:79,038,289−79,038,886
Frequency
cns
a:0.01, b:1.04, c:63.99
TMMINS−1706 (L: 197)
chr15:84,444,475−84,444,672
Frequency
cns
a:0.19, b:0.97, c:5.93
TMMINS−1716 (L: 158)
chr15:90,600,602−90,600,760
Frequency
cns
a:−0.13, b:0.79, c:4.43
TMMINS−1718 (L: 343)
chr15:90,920,593−90,920,936
Frequency
cns
a:0.39, b:1.00, c:9.98
TMMINS−1737 (L: 285)
chr16:182,788−183,073
Frequency
cns
a:0.13, b:0.70, c:12.89
TMMINS−1738 (L: 896)
chr16:239,578−240,474
Frequency
cns
a:0.15, b:0.61, c:25.76
TMMINS−1744 (L: 107)
chr16:762,905−763,012
Frequency
cns
a:0.14, b:0.80, c:4.05
TMMINS−1756 (L: 5982)
chr16:2,867,246−2,873,228
Frequency
cns
a:−0.31, b:0.99, c:67.48
TMMINS−1766 (L: 839)
chr16:7,159,443−7,160,282
Frequency
cns
a:−0.13, b:0.99, c:21.05

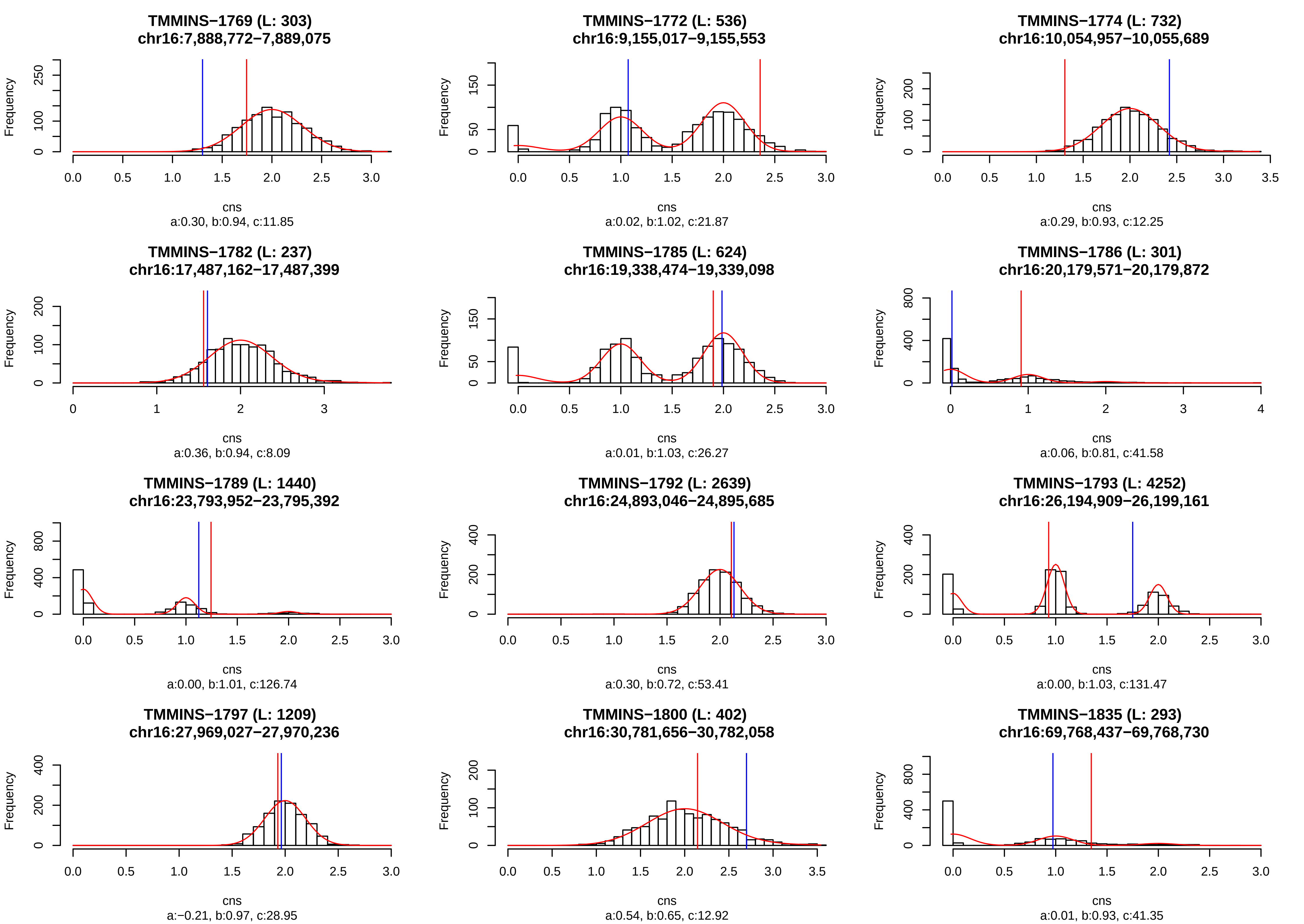
TMMINS−1769 (L: 303)
chr16:7,888,772−7,889,075
Frequency
cns
a:0.30, b:0.94, c:11.85
TMMINS−1772 (L: 536)
chr16:9,155,017−9,155,553
Frequency
cns
a:0.02, b:1.02, c:21.87
TMMINS−1774 (L: 732)
chr16:10,054,957−10,055,689
Frequency
cns
a:0.29, b:0.93, c:12.25
TMMINS−1782 (L: 237)
chr16:17,487,162−17,487,399
Frequency
cns
a:0.36, b:0.94, c:8.09
TMMINS−1785 (L: 624)
chr16:19,338,474−19,339,098
Frequency
cns
a:0.01, b:1.03, c:26.27
TMMINS−1786 (L: 301)
chr16:20,179,571−20,179,872
Frequency
cns
a:0.06, b:0.81, c:41.58
TMMINS−1789 (L: 1440)
chr16:23,793,952−23,795,392
Frequency
cns
a:0.00, b:1.01, c:126.74
TMMINS−1792 (L: 2639)
chr16:24,893,046−24,895,685
Frequency
cns
a:0.30, b:0.72, c:53.41
TMMINS−1793 (L: 4252)
chr16:26,194,909−26,199,161
Frequency
cns
a:0.00, b:1.03, c:131.47
TMMINS−1797 (L: 1209)
chr16:27,969,027−27,970,236
Frequency
cns
a:−0.21, b:0.97, c:28.95
TMMINS−1800 (L: 402)
chr16:30,781,656−30,782,058
Frequency
cns
a:0.54, b:0.65, c:12.92
TMMINS−1835 (L: 293)
chr16:69,768,437−69,768,730
Frequency
cns
a:0.01, b:0.93, c:41.35

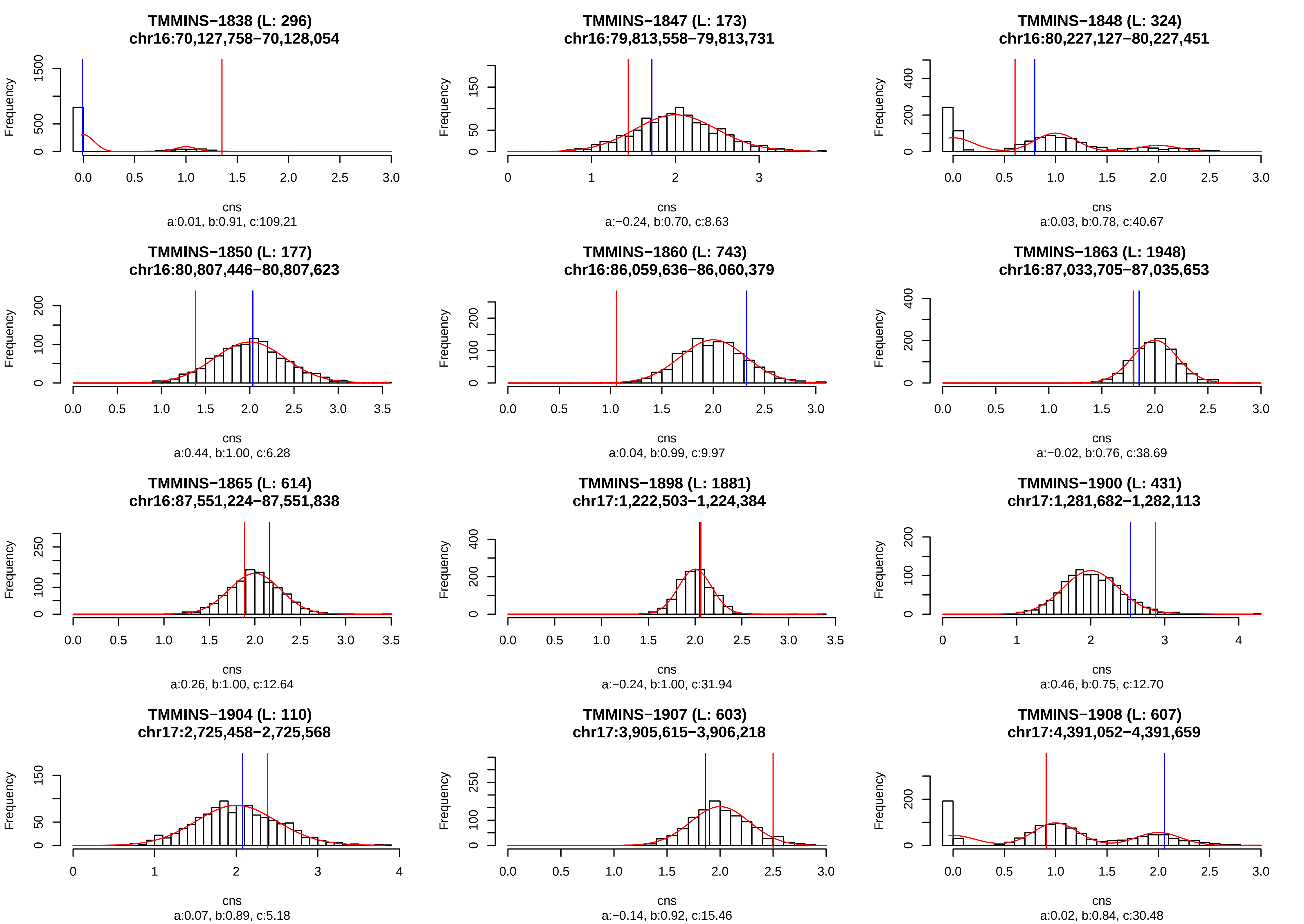
TMMINS−1838 (L: 296)
chr16:70,127,758−70,128,054
a:0.01, b:0.91, c:109.21
TMMINS−1847 (L: 173)
chr16:79,813,558−79,813,731
a:−0.24, b:0.70, c:8.63
TMMINS−1848 (L: 324)
chr16:80,227,127−80,227,451
a:0.03, b:0.78, c:40.67
TMMINS−1850 (L: 177)
chr16:80,807,446−80,807,623
a:0.44, b:1.00, c:6.28
TMMINS−1860 (L: 743)
chr16:86,059,636−86,060,379
a:0.04, b:0.99, c:9.97
TMMINS−1863 (L: 1948)
chr16:87,033,705−87,035,653
a:−0.02, b:0.76, c:38.69
TMMINS−1865 (L: 614)
chr16:87,551,224−87,551,838
a:0.26, b:1.00, c:12.64
TMMINS−1898 (L: 1881)
chr17:1,222,503−1,224,384
a:−0.24, b:1.00, c:31.94
TMMINS−1900 (L: 431)
chr17:1,281,682−1,282,113
a:0.46, b:0.75, c:12.70
TMMINS−1904 (L: 110)
chr17:2,725,458−2,725,568
a:0.07, b:0.89, c:5.18
TMMINS−1907 (L: 603)
chr17:3,905,615−3,906,218
a:−0.14, b:0.92, c:15.46
TMMINS−1908 (L: 607)
chr17:4,391,052−4,391,659
a:0.02, b:0.84, c:30.48
Frequency
cns

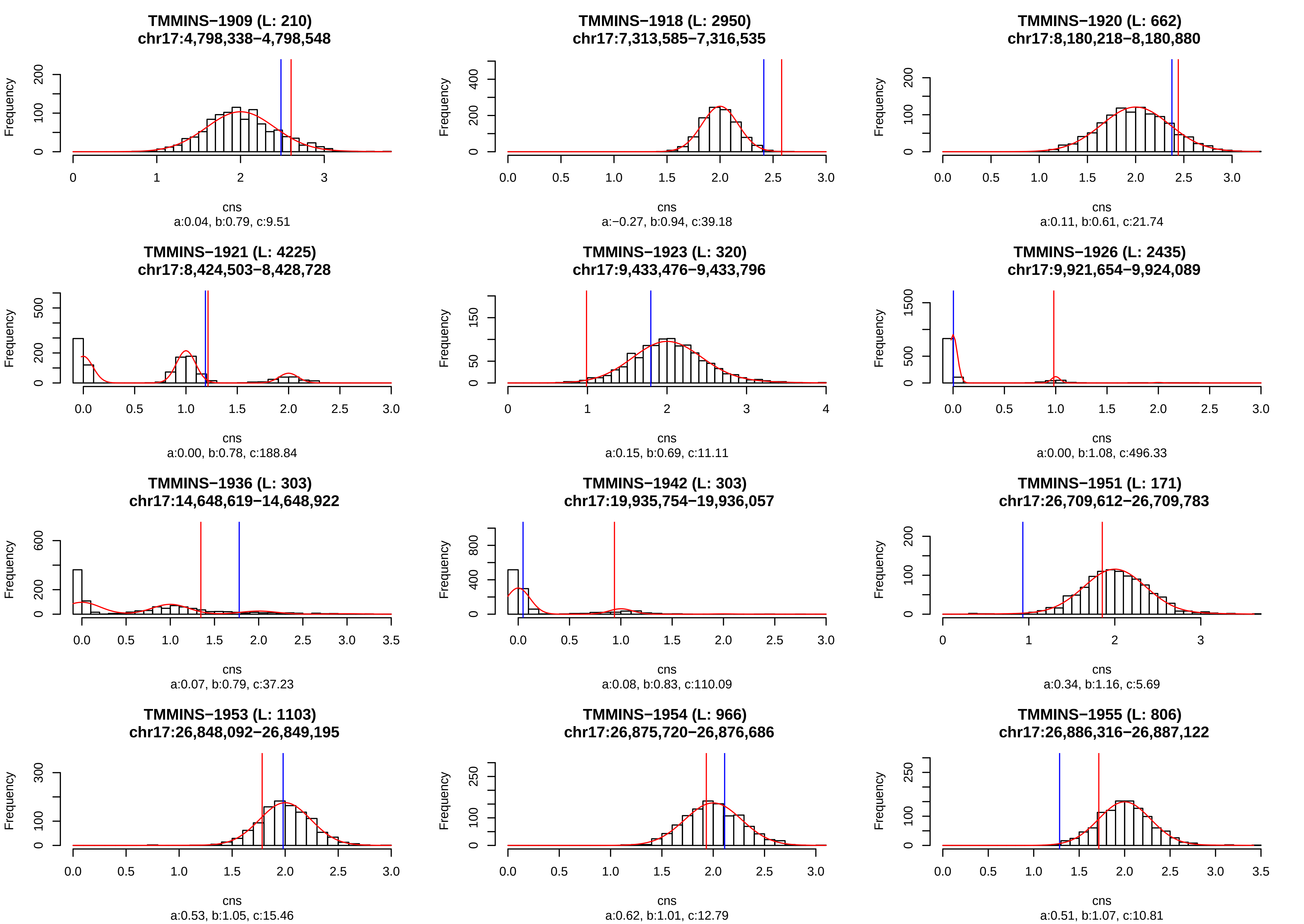
TMMINS−1909 (L: 210)
chr17:4,798,338−4,798,548
Frequency
cns
a:0.04, b:0.79, c:9.51
TMMINS−1918 (L: 2950)
chr17:7,313,585−7,316,535
Frequency
cns
a:−0.27, b:0.94, c:39.18
TMMINS−1920 (L: 662)
chr17:8,180,218−8,180,880
Frequency
cns
a:0.11, b:0.61, c:21.74
TMMINS−1921 (L: 4225)
chr17:8,424,503−8,428,728
Frequency
cns
a:0.00, b:0.78, c:188.84
TMMINS−1923 (L: 320)
chr17:9,433,476−9,433,796
Frequency
cns
a:0.15, b:0.69, c:11.11
TMMINS−1926 (L: 2435)
chr17:9,921,654−9,924,089
Frequency
cns
a:0.00, b:1.08, c:496.33
TMMINS−1936 (L: 303)
chr17:14,648,619−14,648,922
Frequency
cns
a:0.07, b:0.79, c:37.23
TMMINS−1942 (L: 303)
chr17:19,935,754−19,936,057
Frequency
cns
a:0.08, b:0.83, c:110.09
TMMINS−1951 (L: 171)
chr17:26,709,612−26,709,783
Frequency
cns
a:0.34, b:1.16, c:5.69
TMMINS−1953 (L: 1103)
chr17:26,848,092−26,849,195
Frequency
cns
a:0.53, b:1.05, c:15.46
TMMINS−1954 (L: 966)
chr17:26,875,720−26,876,686
Frequency
cns
a:0.62, b:1.01, c:12.79
TMMINS−1955 (L: 806)
chr17:26,886,316−26,887,122
Frequency
cns
a:0.51, b:1.07, c:10.81

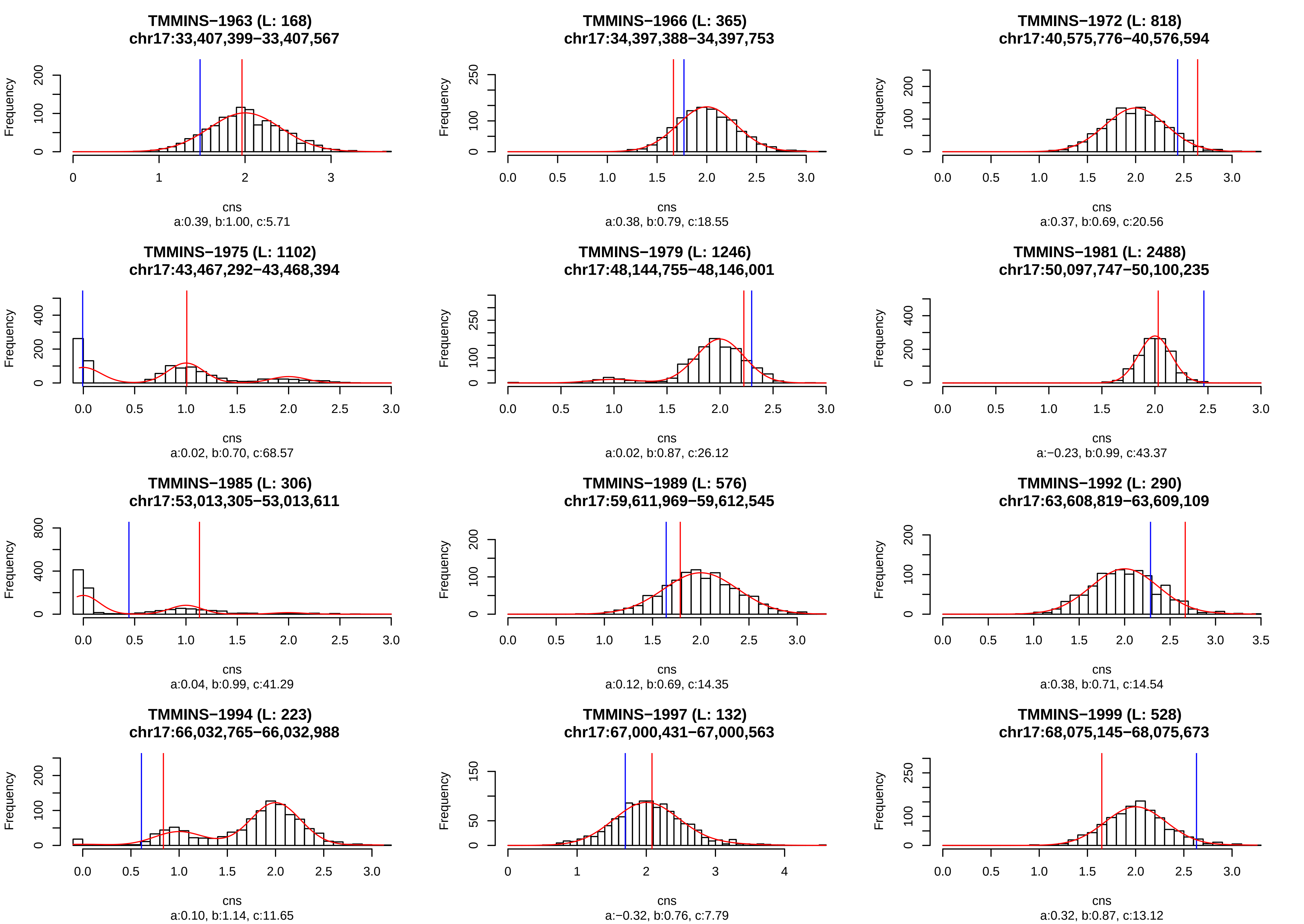

TMMINS−1963 (L: 168)
chr17:33,407,399−33,407,567
a:0.39, b:1.00, c:5.71
TMMINS−1966 (L: 365)
chr17:34,397,388−34,397,753
a:0.38, b:0.79, c:18.55
TMMINS−1972 (L: 818)
chr17:40,575,776−40,576,594
a:0.37, b:0.69, c:20.56
TMMINS−1975 (L: 1102)
chr17:43,467,292−43,468,394
a:0.02, b:0.70, c:68.57
TMMINS−1979 (L: 1246)
chr17:48,144,755−48,146,001
a:0.02, b:0.87, c:26.12
TMMINS−1981 (L: 2488)
chr17:50,097,747−50,100,235
a:−0.23, b:0.99, c:43.37
TMMINS−1985 (L: 306)
chr17:53,013,305−53,013,611
a:0.04, b:0.99, c:41.29
TMMINS−1989 (L: 576)
chr17:59,611,969−59,612,545
a:0.12, b:0.69, c:14.35
TMMINS−1992 (L: 290)
chr17:63,608,819−63,609,109
a:0.38, b:0.71, c:14.54
TMMINS−1994 (L: 223)
chr17:66,032,765−66,032,988
a:0.10, b:1.14, c:11.65
TMMINS−1997 (L: 132)
chr17:67,000,431−67,000,563
a:−0.32, b:0.76, c:7.79
TMMINS−1999 (L: 528)
chr17:68,075,145−68,075,673
a:0.32, b:0.87, c:13.12
Frequency
cns

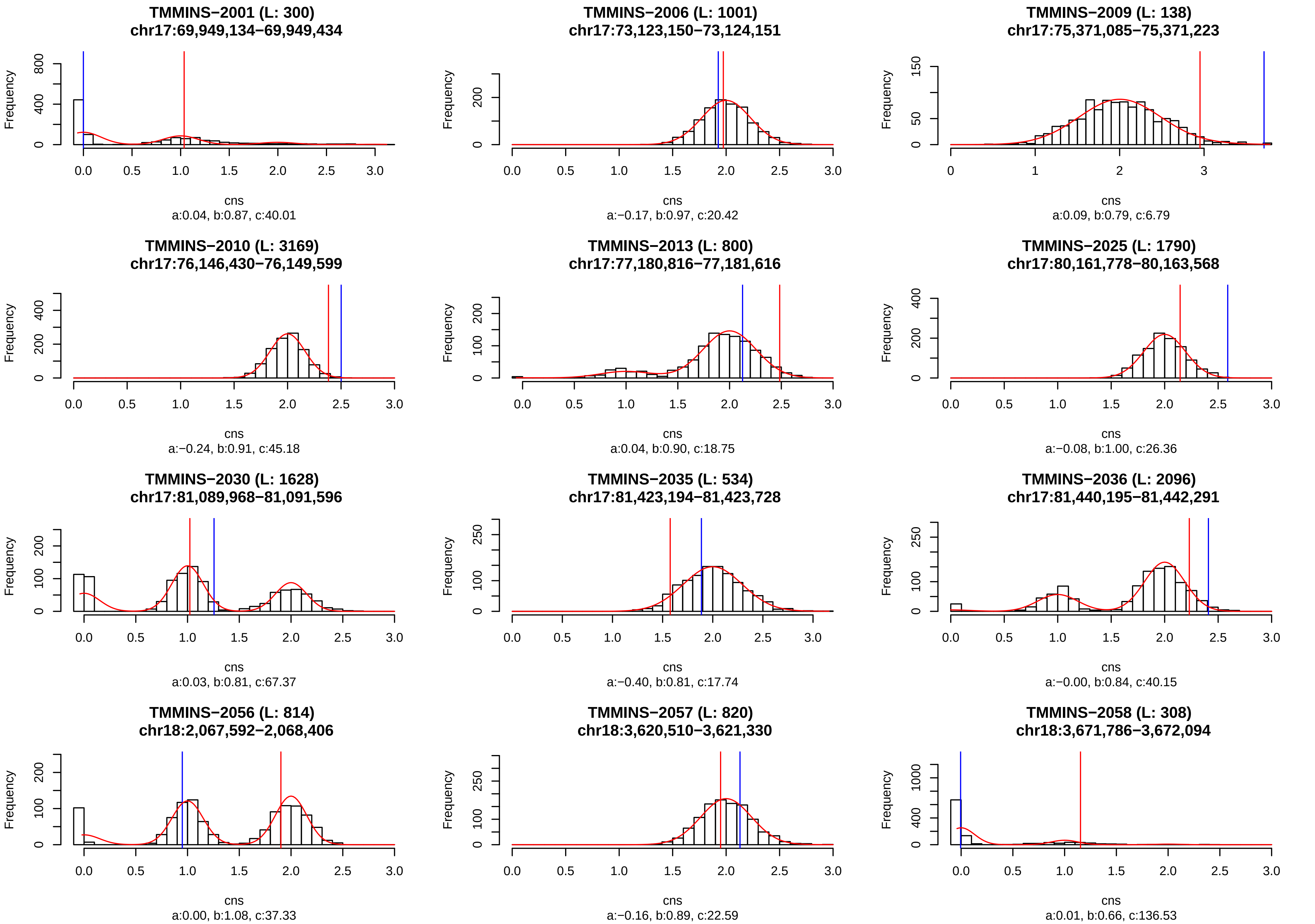
TMMINS−2001 (L: 300)
chr17:69,949,134−69,949,434
Frequency
cns
a:0.04, b:0.87, c:40.01
TMMINS−2006 (L: 1001)
chr17:73,123,150−73,124,151
Frequency
cns
a:−0.17, b:0.97, c:20.42
TMMINS−2009 (L: 138)
chr17:75,371,085−75,371,223
Frequency
cns
a:0.09, b:0.79, c:6.79
TMMINS−2010 (L: 3169)
chr17:76,146,430−76,149,599
Frequency
cns
a:−0.24, b:0.91, c:45.18
TMMINS−2013 (L: 800)
chr17:77,180,816−77,181,616
Frequency
cns
a:0.04, b:0.90, c:18.75
TMMINS−2025 (L: 1790)
chr17:80,161,778−80,163,568
Frequency
cns
a:−0.08, b:1.00, c:26.36
TMMINS−2030 (L: 1628)
chr17:81,089,968−81,091,596
Frequency
cns
a:0.03, b:0.81, c:67.37
TMMINS−2035 (L: 534)
chr17:81,423,194−81,423,728
Frequency
cns
a:−0.40, b:0.81, c:17.74
TMMINS−2036 (L: 2096)
chr17:81,440,195−81,442,291
Frequency
cns
a:−0.00, b:0.84, c:40.15
TMMINS−2056 (L: 814)
chr18:2,067,592−2,068,406
Frequency
cns
a:0.00, b:1.08, c:37.33
TMMINS−2057 (L: 820)
chr18:3,620,510−3,621,330
Frequency
cns
a:−0.16, b:0.89, c:22.59
TMMINS−2058 (L: 308)
chr18:3,671,786−3,672,094
Frequency
cns
a:0.01, b:0.66, c:136.53

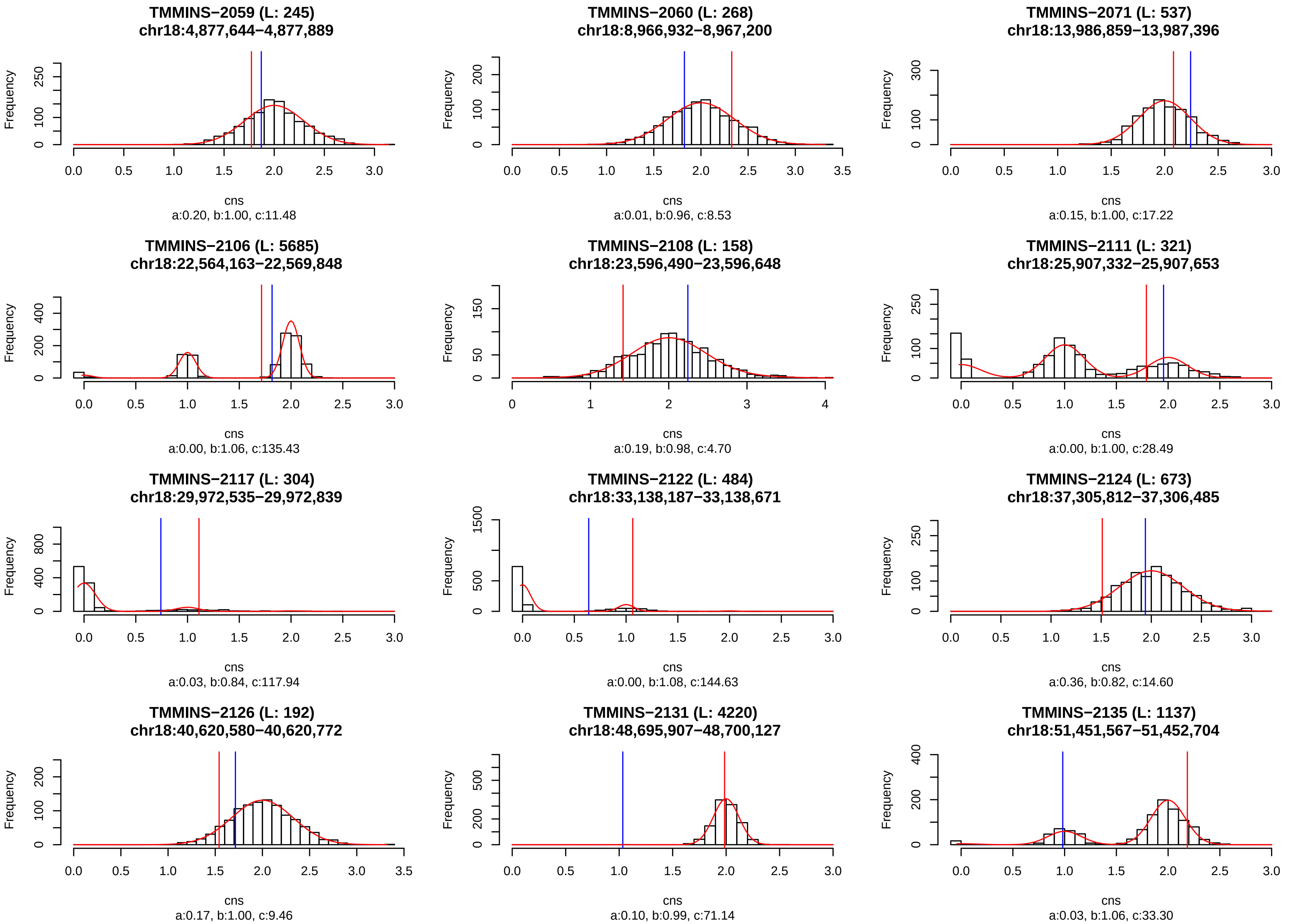
TMMINS−2059 (L: 245)
chr18:4,877,644−4,877,889
Frequency
cns
a:0.20, b:1.00, c:11.48
TMMINS−2060 (L: 268)
chr18:8,966,932−8,967,200
Frequency
cns
a:0.01, b:0.96, c:8.53
TMMINS−2071 (L: 537)
chr18:13,986,859−13,987,396
Frequency
cns
a:0.15, b:1.00, c:17.22
TMMINS−2106 (L: 5685)
chr18:22,564,163−22,569,848
Frequency
cns
a:0.00, b:1.06, c:135.43
TMMINS−2108 (L: 158)
chr18:23,596,490−23,596,648
Frequency
cns
a:0.19, b:0.98, c:4.70
TMMINS−2111 (L: 321)
chr18:25,907,332−25,907,653
Frequency
cns
a:0.00, b:1.00, c:28.49
TMMINS−2117 (L: 304)
chr18:29,972,535−29,972,839
Frequency
cns
a:0.03, b:0.84, c:117.94
TMMINS−2122 (L: 484)
chr18:33,138,187−33,138,671
Frequency
cns
a:0.00, b:1.08, c:144.63
TMMINS−2124 (L: 673)
chr18:37,305,812−37,306,485
Frequency
cns
a:0.36, b:0.82, c:14.60
TMMINS−2126 (L: 192)
chr18:40,620,580−40,620,772
Frequency
cns
a:0.17, b:1.00, c:9.46
TMMINS−2131 (L: 4220)
chr18:48,695,907−48,700,127
Frequency
cns
a:0.10, b:0.99, c:71.14
TMMINS−2135 (L: 1137)
chr18:51,451,567−51,452,704
Frequency
cns
a:0.03, b:1.06, c:33.30

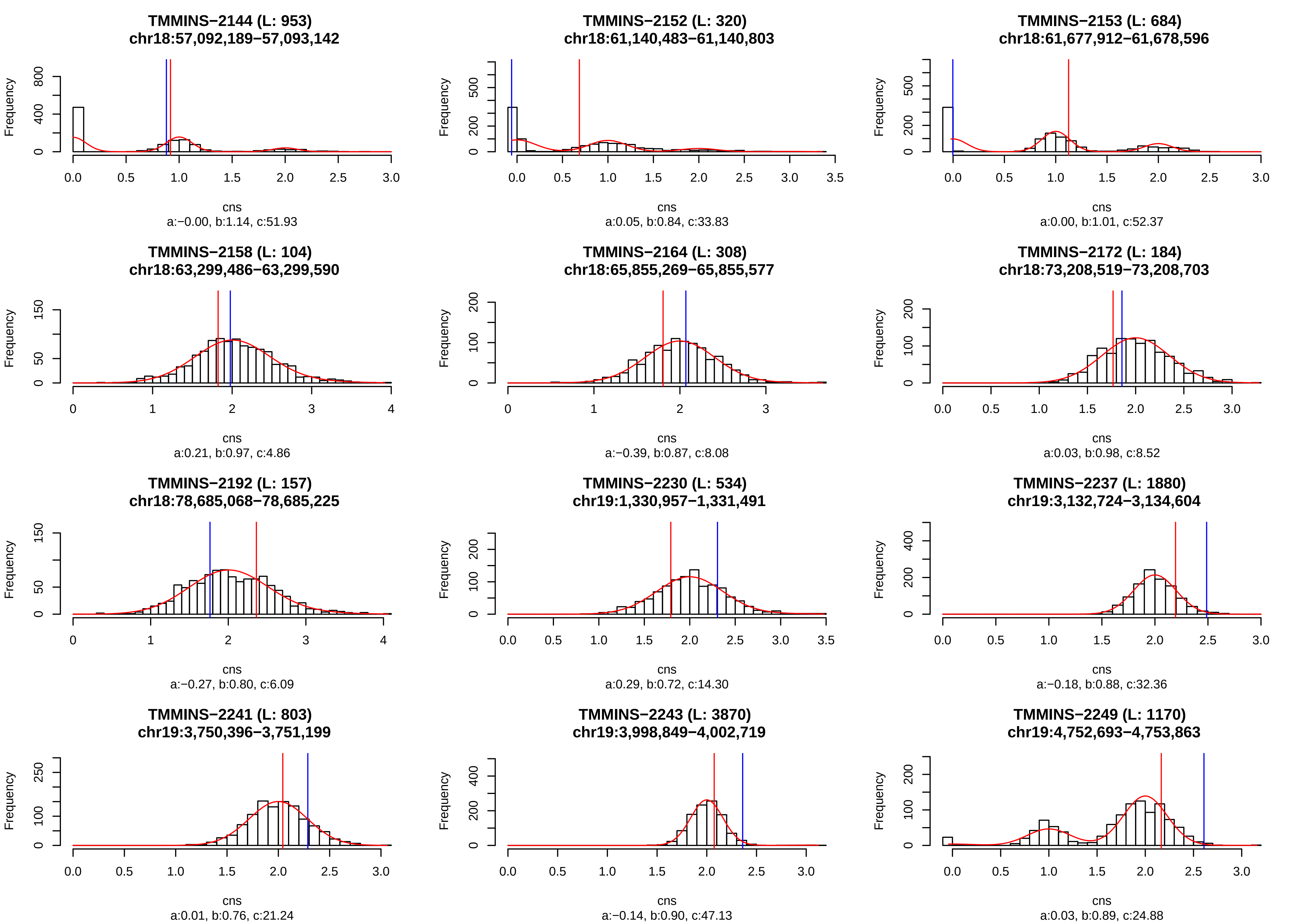
TMMINS−2144 (L: 953)
chr18:57,092,189−57,093,142
Frequency
cns
a:−0.00, b:1.14, c:51.93
TMMINS−2152 (L: 320)
chr18:61,140,483−61,140,803
Frequency
cns
a:0.05, b:0.84, c:33.83
TMMINS−2153 (L: 684)
chr18:61,677,912−61,678,596
Frequency
cns
a:0.00, b:1.01, c:52.37
TMMINS−2158 (L: 104)
chr18:63,299,486−63,299,590
Frequency
cns
a:0.21, b:0.97, c:4.86
TMMINS−2164 (L: 308)
chr18:65,855,269−65,855,577
Frequency
cns
a:−0.39, b:0.87, c:8.08
TMMINS−2172 (L: 184)
chr18:73,208,519−73,208,703
Frequency
cns
a:0.03, b:0.98, c:8.52
TMMINS−2192 (L: 157)
chr18:78,685,068−78,685,225
Frequency
cns
a:−0.27, b:0.80, c:6.09
TMMINS−2230 (L: 534)
chr19:1,330,957−1,331,491
Frequency
cns
a:0.29, b:0.72, c:14.30
TMMINS−2237 (L: 1880)
chr19:3,132,724−3,134,604
Frequency
cns
a:−0.18, b:0.88, c:32.36
TMMINS−2241 (L: 803)
chr19:3,750,396−3,751,199
Frequency
cns
a:0.01, b:0.76, c:21.24
TMMINS−2243 (L: 3870)
chr19:3,998,849−4,002,719
Frequency
cns
a:−0.14, b:0.90, c:47.13
TMMINS−2249 (L: 1170)
chr19:4,752,693−4,753,863
Frequency
cns
a:0.03, b:0.89, c:24.88

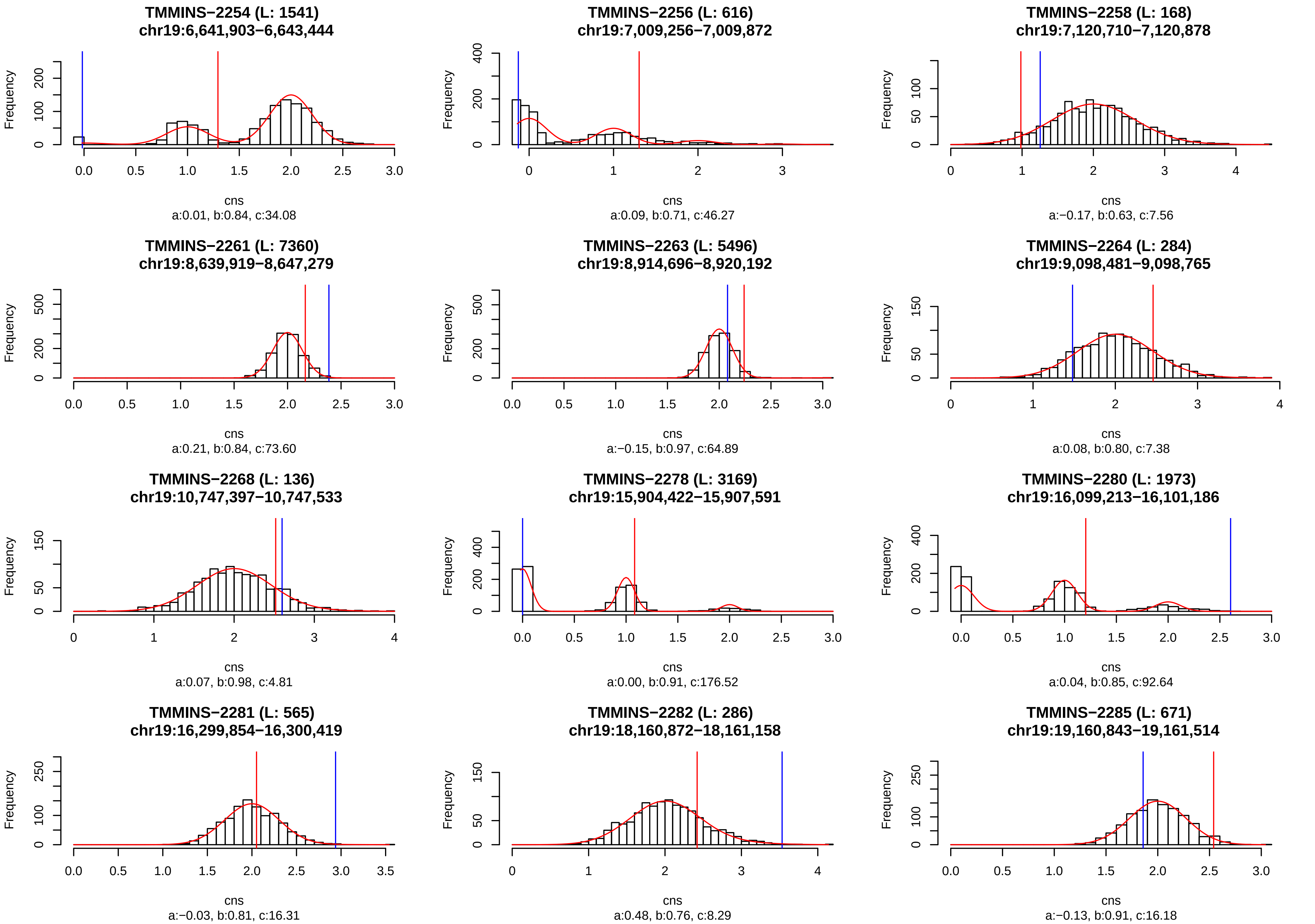
TMMINS−2254 (L: 1541)
chr19:6,641,903−6,643,444
Frequency
cns
a:0.01, b:0.84, c:34.08
TMMINS−2256 (L: 616)
chr19:7,009,256−7,009,872
Frequency
cns
a:0.09, b:0.71, c:46.27
TMMINS−2258 (L: 168)
chr19:7,120,710−7,120,878
Frequency
cns
a:−0.17, b:0.63, c:7.56
TMMINS−2261 (L: 7360)
chr19:8,639,919−8,647,279
Frequency
cns
a:0.21, b:0.84, c:73.60
TMMINS−2263 (L: 5496)
chr19:8,914,696−8,920,192
Frequency
cns
a:−0.15, b:0.97, c:64.89
TMMINS−2264 (L: 284)
chr19:9,098,481−9,098,765
Frequency
cns
a:0.08, b:0.80, c:7.38
TMMINS−2268 (L: 136)
chr19:10,747,397−10,747,533
Frequency
cns
a:0.07, b:0.98, c:4.81
TMMINS−2278 (L: 3169)
chr19:15,904,422−15,907,591
Frequency
cns
a:0.00, b:0.91, c:176.52
TMMINS−2280 (L: 1973)
chr19:16,099,213−16,101,186
Frequency
cns
a:0.04, b:0.85, c:92.64
TMMINS−2281 (L: 565)
chr19:16,299,854−16,300,419
Frequency
cns
a:−0.03, b:0.81, c:16.31
TMMINS−2282 (L: 286)
chr19:18,160,872−18,161,158
Frequency
cns
a:0.48, b:0.76, c:8.29
TMMINS−2285 (L: 671)
chr19:19,160,843−19,161,514
Frequency
cns
a:−0.13, b:0.91, c:16.18

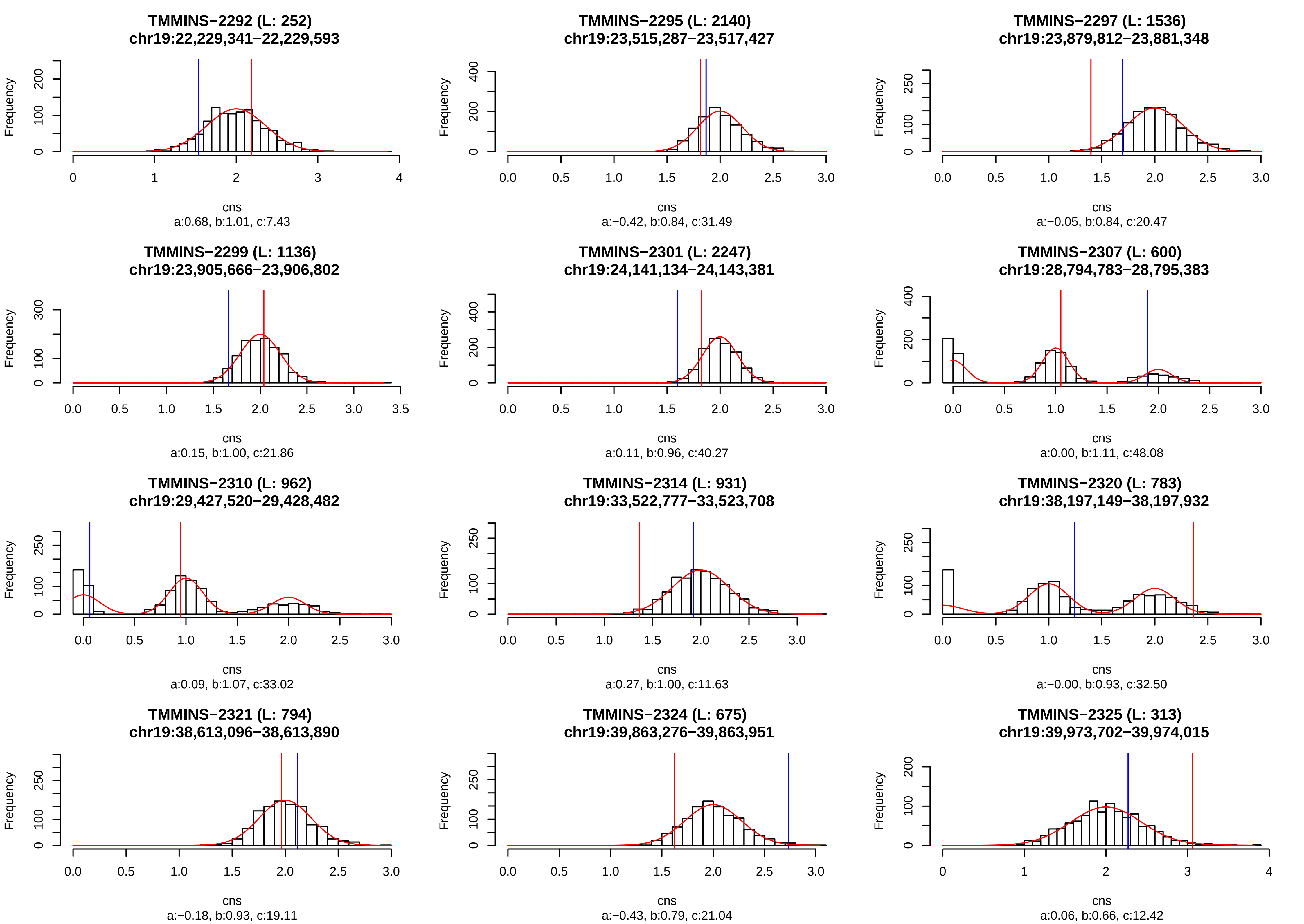
TMMINS−2292 (L: 252)
chr19:22,229,341−22,229,593
Frequency
cns
a:0.68, b:1.01, c:7.43
TMMINS−2295 (L: 2140)
chr19:23,515,287−23,517,427
Frequency
cns
a:−0.42, b:0.84, c:31.49
TMMINS−2297 (L: 1536)
chr19:23,879,812−23,881,348
Frequency
cns
a:−0.05, b:0.84, c:20.47
TMMINS−2299 (L: 1136)
chr19:23,905,666−23,906,802
Frequency
cns
a:0.15, b:1.00, c:21.86
TMMINS−2301 (L: 2247)
chr19:24,141,134−24,143,381
Frequency
cns
a:0.11, b:0.96, c:40.27
TMMINS−2307 (L: 600)
chr19:28,794,783−28,795,383
Frequency
cns
a:0.00, b:1.11, c:48.08
TMMINS−2310 (L: 962)
chr19:29,427,520−29,428,482
Frequency
cns
a:0.09, b:1.07, c:33.02
TMMINS−2314 (L: 931)
chr19:33,522,777−33,523,708
Frequency
cns
a:0.27, b:1.00, c:11.63
TMMINS−2320 (L: 783)
chr19:38,197,149−38,197,932
Frequency
cns
a:−0.00, b:0.93, c:32.50
TMMINS−2321 (L: 794)
chr19:38,613,096−38,613,890
Frequency
cns
a:−0.18, b:0.93, c:19.11
TMMINS−2324 (L: 675)
chr19:39,863,276−39,863,951
Frequency
cns
a:−0.43, b:0.79, c:21.04
TMMINS−2325 (L: 313)
chr19:39,973,702−39,974,015
Frequency
cns
a:0.06, b:0.66, c:12.42

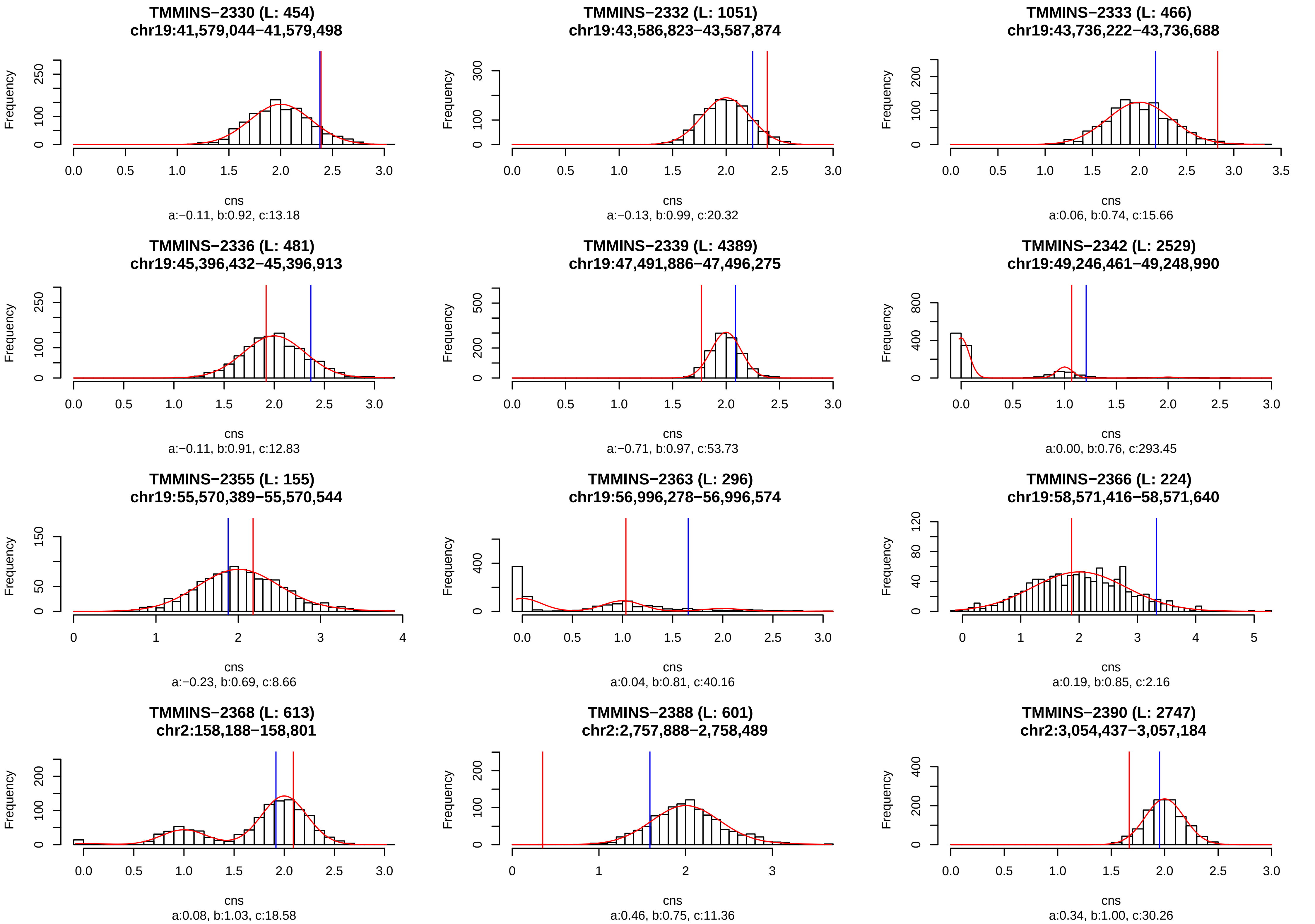
TMMINS−2330 (L: 454)
chr19:41,579,044−41,579,498
Frequency
cns
a:−0.11, b:0.92, c:13.18
TMMINS−2332 (L: 1051)
chr19:43,586,823−43,587,874
Frequency
cns
a:−0.13, b:0.99, c:20.32
TMMINS−2333 (L: 466)
chr19:43,736,222−43,736,688
Frequency
cns
a:0.06, b:0.74, c:15.66
TMMINS−2336 (L: 481)
chr19:45,396,432−45,396,913
Frequency
cns
a:−0.11, b:0.91, c:12.83
TMMINS−2339 (L: 4389)
chr19:47,491,886−47,496,275
Frequency
cns
a:−0.71, b:0.97, c:53.73
TMMINS−2342 (L: 2529)
chr19:49,246,461−49,248,990
Frequency
cns
a:0.00, b:0.76, c:293.45
TMMINS−2355 (L: 155)
chr19:55,570,389−55,570,544
Frequency
cns
a:−0.23, b:0.69, c:8.66
TMMINS−2363 (L: 296)
chr19:56,996,278−56,996,574
Frequency
cns
a:0.04, b:0.81, c:40.16
TMMINS−2366 (L: 224)
chr19:58,571,416−58,571,640
Frequency
cns
a:0.19, b:0.85, c:2.16
TMMINS−2368 (L: 613)
chr2:158,188−158,801
Frequency
cns
a:0.08, b:1.03, c:18.58
TMMINS−2388 (L: 601)
chr2:2,757,888−2,758,489
Frequency
cns
a:0.46, b:0.75, c:11.36
TMMINS−2390 (L: 2747)
chr2:3,054,437−3,057,184
Frequency
cns
a:0.34, b:1.00, c:30.26

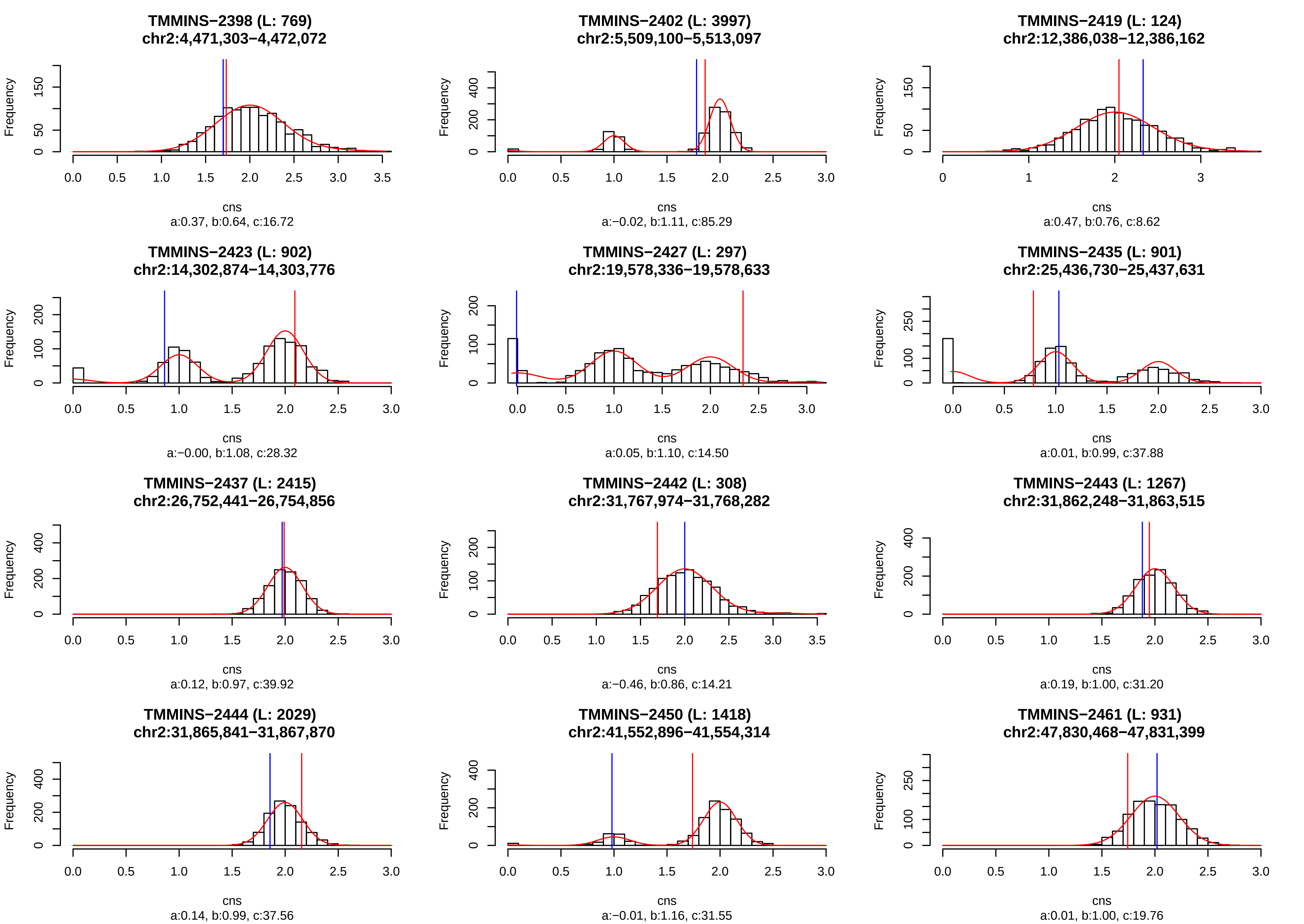
TMMINS−2398 (L: 769)
chr2:4,471,303−4,472,072
Frequency
cns
a:0.37, b:0.64, c:16.72
TMMINS−2402 (L: 3997)
chr2:5,509,100−5,513,097
Frequency
cns
a:−0.02, b:1.11, c:85.29
TMMINS−2419 (L: 124)
chr2:12,386,038−12,386,162
Frequency
cns
a:0.47, b:0.76, c:8.62
TMMINS−2423 (L: 902)
chr2:14,302,874−14,303,776
Frequency
cns
a:−0.00, b:1.08, c:28.32
TMMINS−2427 (L: 297)
chr2:19,578,336−19,578,633
Frequency
cns
a:0.05, b:1.10, c:14.50
TMMINS−2435 (L: 901)
chr2:25,436,730−25,437,631
Frequency
cns
a:0.01, b:0.99, c:37.88
TMMINS−2437 (L: 2415)
chr2:26,752,441−26,754,856
Frequency
cns
a:0.12, b:0.97, c:39.92
TMMINS−2442 (L: 308)
chr2:31,767,974−31,768,282
Frequency
cns
a:−0.46, b:0.86, c:14.21
TMMINS−2443 (L: 1267)
chr2:31,862,248−31,863,515
Frequency
cns
a:0.19, b:1.00, c:31.20
TMMINS−2444 (L: 2029)
chr2:31,865,841−31,867,870
Frequency
cns
a:0.14, b:0.99, c:37.56
TMMINS−2450 (L: 1418)
chr2:41,552,896−41,554,314
Frequency
cns
a:−0.01, b:1.16, c:31.55
TMMINS−2461 (L: 931)
chr2:47,830,468−47,831,399
Frequency
cns
a:0.01, b:1.00, c:19.76

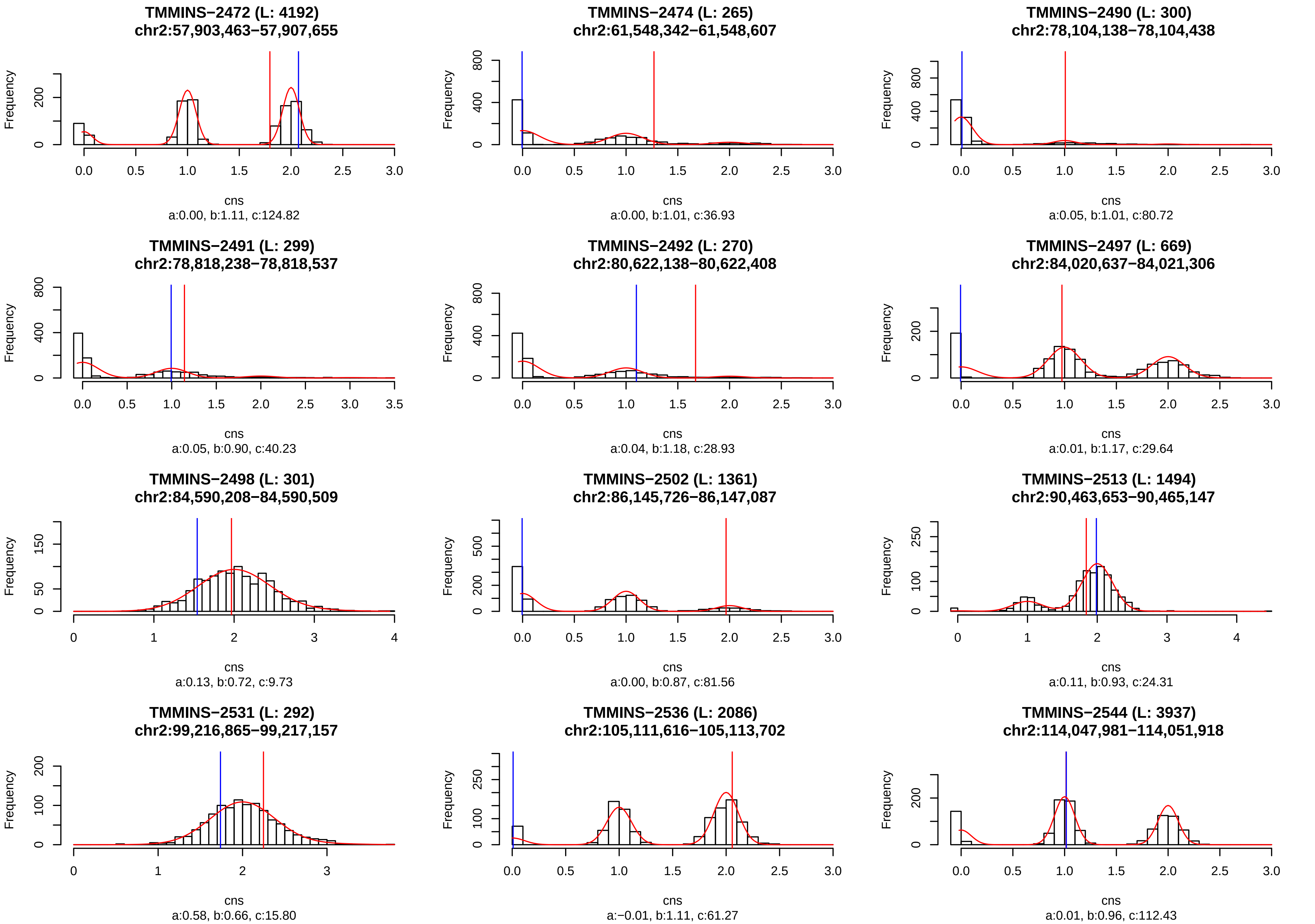

TMMINS−2472 (L: 4192)
chr2:57,903,463−57,907,655
a:0.00, b:1.11, c:124.82
TMMINS−2474 (L: 265)
chr2:61,548,342−61,548,607
a:0.00, b:1.01, c:36.93
TMMINS−2490 (L: 300)
chr2:78,104,138−78,104,438
a:0.05, b:1.01, c:80.72
TMMINS−2491 (L: 299)
chr2:78,818,238−78,818,537
a:0.05, b:0.90, c:40.23
TMMINS−2492 (L: 270)
chr2:80,622,138−80,622,408
a:0.04, b:1.18, c:28.93
TMMINS−2497 (L: 669)
chr2:84,020,637−84,021,306
a:0.01, b:1.17, c:29.64
TMMINS−2498 (L: 301)
chr2:84,590,208−84,590,509
a:0.13, b:0.72, c:9.73
TMMINS−2502 (L: 1361)
chr2:86,145,726−86,147,087
a:0.00, b:0.87, c:81.56
TMMINS−2513 (L: 1494)
chr2:90,463,653−90,465,147
a:0.11, b:0.93, c:24.31
TMMINS−2531 (L: 292)
chr2:99,216,865−99,217,157
a:0.58, b:0.66, c:15.80
TMMINS−2536 (L: 2086)
chr2:105,111,616−105,113,702
a:−0.01, b:1.11, c:61.27
TMMINS−2544 (L: 3937)
chr2:114,047,981−114,051,918
a:0.01, b:0.96, c:112.43
Frequency
cns

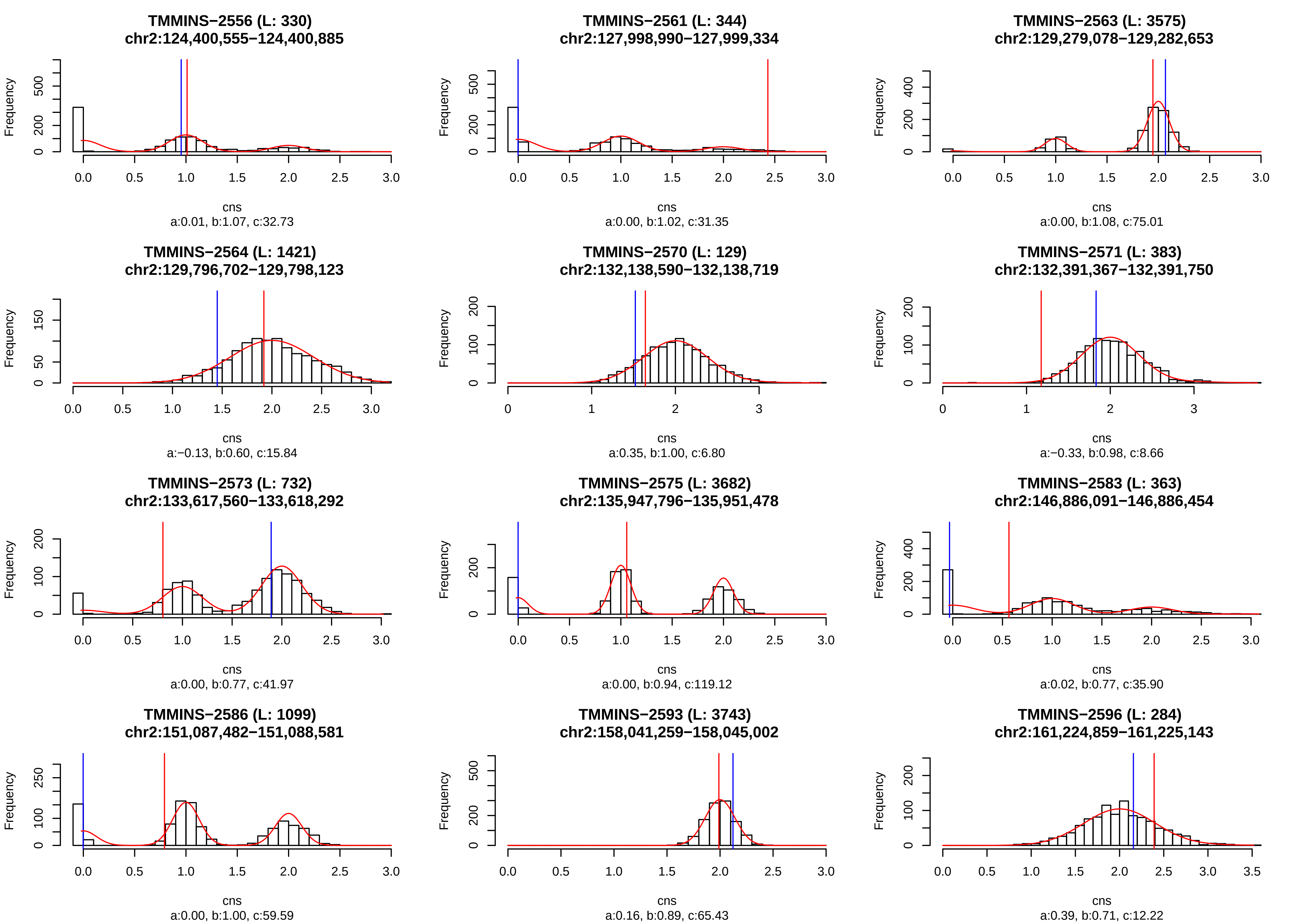
TMMINS−2556 (L: 330)
chr2:124,400,555−124,400,885
Frequency
cns
a:0.01, b:1.07, c:32.73
TMMINS−2561 (L: 344)
chr2:127,998,990−127,999,334
Frequency
cns
a:0.00, b:1.02, c:31.35
TMMINS−2563 (L: 3575)
chr2:129,279,078−129,282,653
Frequency
cns
a:0.00, b:1.08, c:75.01
TMMINS−2564 (L: 1421)
chr2:129,796,702−129,798,123
Frequency
cns
a:−0.13, b:0.60, c:15.84
TMMINS−2570 (L: 129)
chr2:132,138,590−132,138,719
Frequency
cns
a:0.35, b:1.00, c:6.80
TMMINS−2571 (L: 383)
chr2:132,391,367−132,391,750
Frequency
cns
a:−0.33, b:0.98, c:8.66
TMMINS−2573 (L: 732)
chr2:133,617,560−133,618,292
Frequency
cns
a:0.00, b:0.77, c:41.97
TMMINS−2575 (L: 3682)
chr2:135,947,796−135,951,478
Frequency
cns
a:0.00, b:0.94, c:119.12
TMMINS−2583 (L: 363)
chr2:146,886,091−146,886,454
Frequency
cns
a:0.02, b:0.77, c:35.90
TMMINS−2586 (L: 1099)
chr2:151,087,482−151,088,581
Frequency
cns
a:0.00, b:1.00, c:59.59
TMMINS−2593 (L: 3743)
chr2:158,041,259−158,045,002
Frequency
cns
a:0.16, b:0.89, c:65.43
TMMINS−2596 (L: 284)
chr2:161,224,859−161,225,143
Frequency
cns
a:0.39, b:0.71, c:12.22

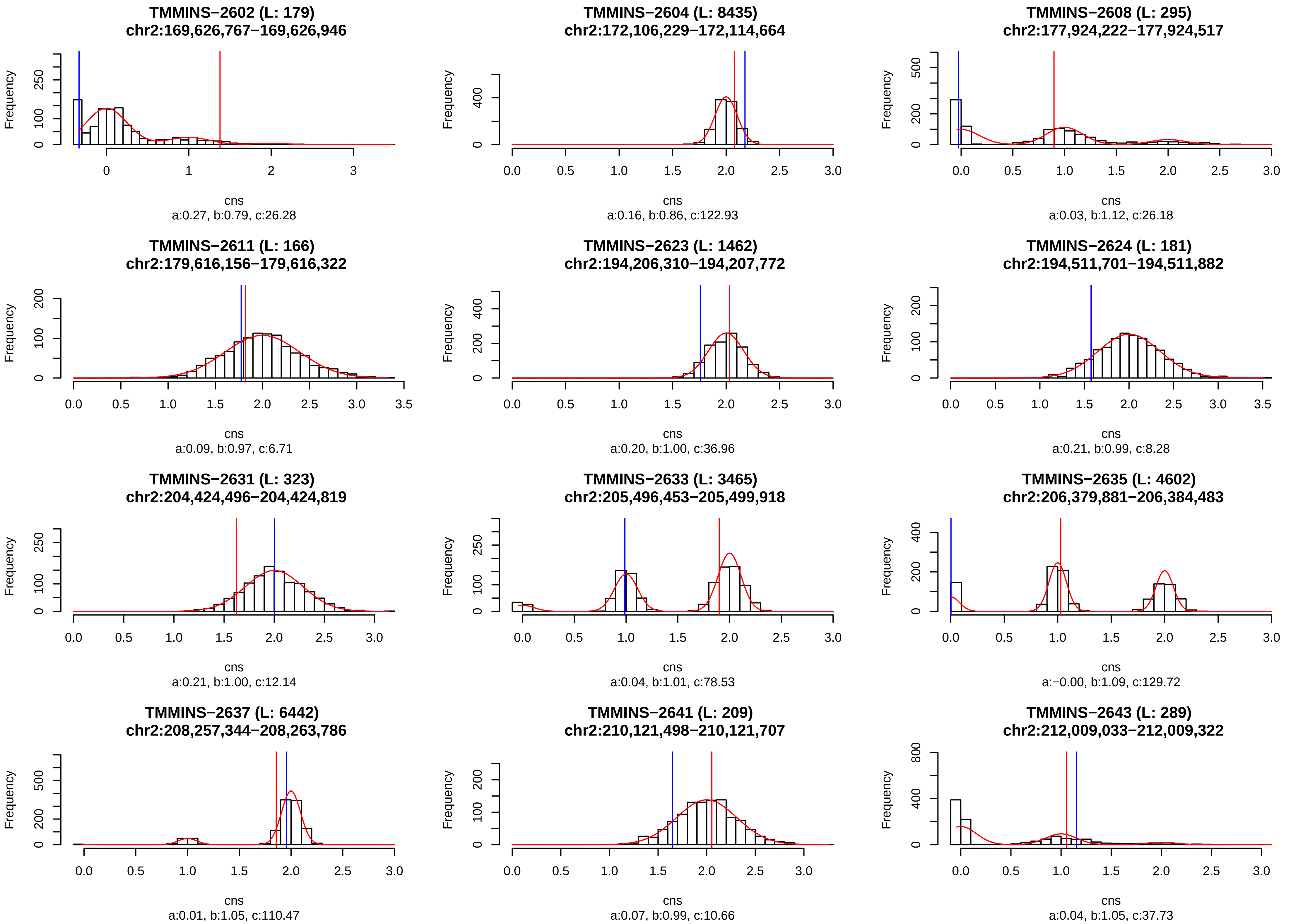
TMMINS−2602 (L: 179)
chr2:169,626,767−169,626,946
Frequency
cns
a:0.27, b:0.79, c:26.28
TMMINS−2604 (L: 8435)
chr2:172,106,229−172,114,664
Frequency
cns
a:0.16, b:0.86, c:122.93
TMMINS−2608 (L: 295)
chr2:177,924,222−177,924,517
Frequency
cns
a:0.03, b:1.12, c:26.18
TMMINS−2611 (L: 166)
chr2:179,616,156−179,616,322
Frequency
cns
a:0.09, b:0.97, c:6.71
TMMINS−2623 (L: 1462)
chr2:194,206,310−194,207,772
Frequency
cns
a:0.20, b:1.00, c:36.96
TMMINS−2624 (L: 181)
chr2:194,511,701−194,511,882
Frequency
cns
a:0.21, b:0.99, c:8.28
TMMINS−2631 (L: 323)
chr2:204,424,496−204,424,819
Frequency
cns
a:0.21, b:1.00, c:12.14
TMMINS−2633 (L: 3465)
chr2:205,496,453−205,499,918
Frequency
cns
a:0.04, b:1.01, c:78.53
TMMINS−2635 (L: 4602)
chr2:206,379,881−206,384,483
Frequency
cns
a:−0.00, b:1.09, c:129.72
TMMINS−2637 (L: 6442)
chr2:208,257,344−208,263,786
Frequency
cns
a:0.01, b:1.05, c:110.47
TMMINS−2641 (L: 209)
chr2:210,121,498−210,121,707
Frequency
cns
a:0.07, b:0.99, c:10.66
TMMINS−2643 (L: 289)
chr2:212,009,033−212,009,322
Frequency
cns
a:0.04, b:1.05, c:37.73

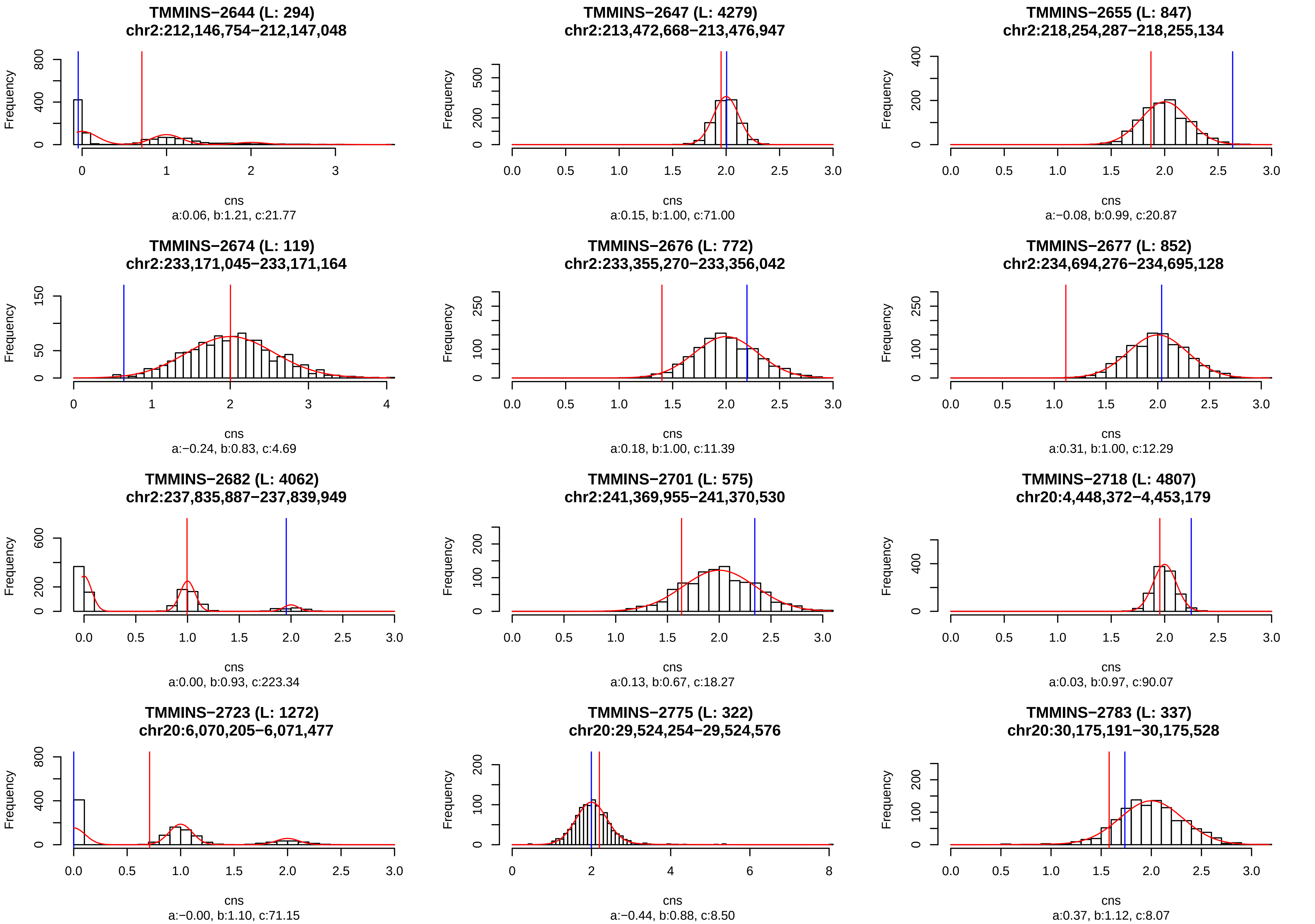
TMMINS−2644 (L: 294)
chr2:212,146,754−212,147,048
Frequency
cns
a:0.06, b:1.21, c:21.77
TMMINS−2647 (L: 4279)
chr2:213,472,668−213,476,947
Frequency
cns
a:0.15, b:1.00, c:71.00
TMMINS−2655 (L: 847)
chr2:218,254,287−218,255,134
Frequency
cns
a:−0.08, b:0.99, c:20.87
TMMINS−2674 (L: 119)
chr2:233,171,045−233,171,164
Frequency
cns
a:−0.24, b:0.83, c:4.69
TMMINS−2676 (L: 772)
chr2:233,355,270−233,356,042
Frequency
cns
a:0.18, b:1.00, c:11.39
TMMINS−2677 (L: 852)
chr2:234,694,276−234,695,128
Frequency
cns
a:0.31, b:1.00, c:12.29
TMMINS−2682 (L: 4062)
chr2:237,835,887−237,839,949
Frequency
cns
a:0.00, b:0.93, c:223.34
TMMINS−2701 (L: 575)
chr2:241,369,955−241,370,530
Frequency
cns
a:0.13, b:0.67, c:18.27
TMMINS−2718 (L: 4807)
chr20:4,448,372−4,453,179
Frequency
cns
a:0.03, b:0.97, c:90.07
TMMINS−2723 (L: 1272)
chr20:6,070,205−6,071,477
Frequency
cns
a:−0.00, b:1.10, c:71.15
TMMINS−2775 (L: 322)
chr20:29,524,254−29,524,576
Frequency
cns
a:−0.44, b:0.88, c:8.50
TMMINS−2783 (L: 337)
chr20:30,175,191−30,175,528
Frequency
cns
a:0.37, b:1.12, c:8.07

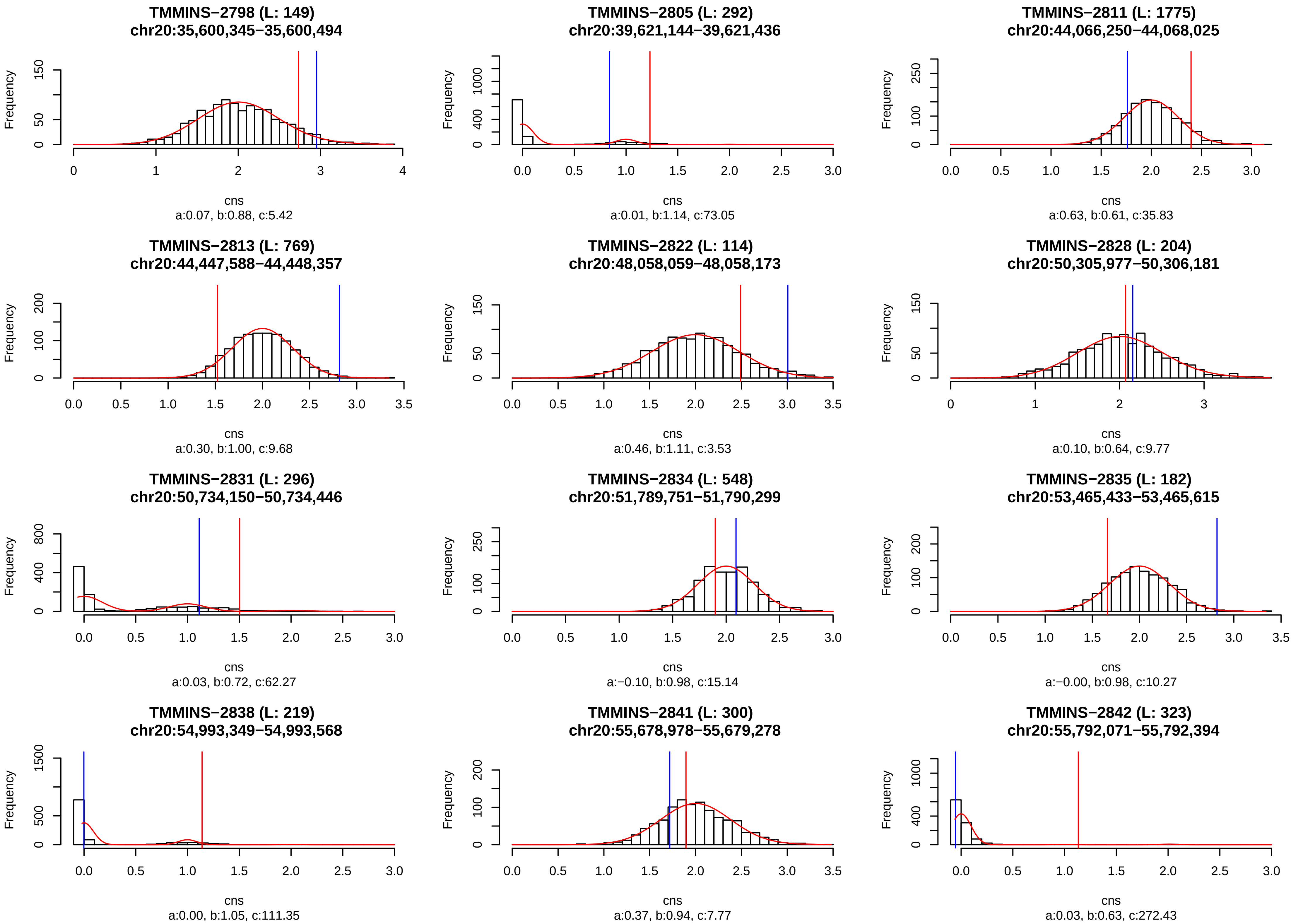
TMMINS−2798 (L: 149)
chr20:35,600,345−35,600,494
Frequency
cns
a:0.07, b:0.88, c:5.42
TMMINS−2805 (L: 292)
chr20:39,621,144−39,621,436
Frequency
cns
a:0.01, b:1.14, c:73.05
TMMINS−2811 (L: 1775)
chr20:44,066,250−44,068,025
Frequency
cns
a:0.63, b:0.61, c:35.83
TMMINS−2813 (L: 769)
chr20:44,447,588−44,448,357
Frequency
cns
a:0.30, b:1.00, c:9.68
TMMINS−2822 (L: 114)
chr20:48,058,059−48,058,173
Frequency
cns
a:0.46, b:1.11, c:3.53
TMMINS−2828 (L: 204)
chr20:50,305,977−50,306,181
Frequency
cns
a:0.10, b:0.64, c:9.77
TMMINS−2831 (L: 296)
chr20:50,734,150−50,734,446
Frequency
cns
a:0.03, b:0.72, c:62.27
TMMINS−2834 (L: 548)
chr20:51,789,751−51,790,299
Frequency
cns
a:−0.10, b:0.98, c:15.14
TMMINS−2835 (L: 182)
chr20:53,465,433−53,465,615
Frequency
cns
a:−0.00, b:0.98, c:10.27
TMMINS−2838 (L: 219)
chr20:54,993,349−54,993,568
Frequency
cns
a:0.00, b:1.05, c:111.35
TMMINS−2841 (L: 300)
chr20:55,678,978−55,679,278
Frequency
cns
a:0.37, b:0.94, c:7.77
TMMINS−2842 (L: 323)
chr20:55,792,071−55,792,394
Frequency
cns
a:0.03, b:0.63, c:272.43

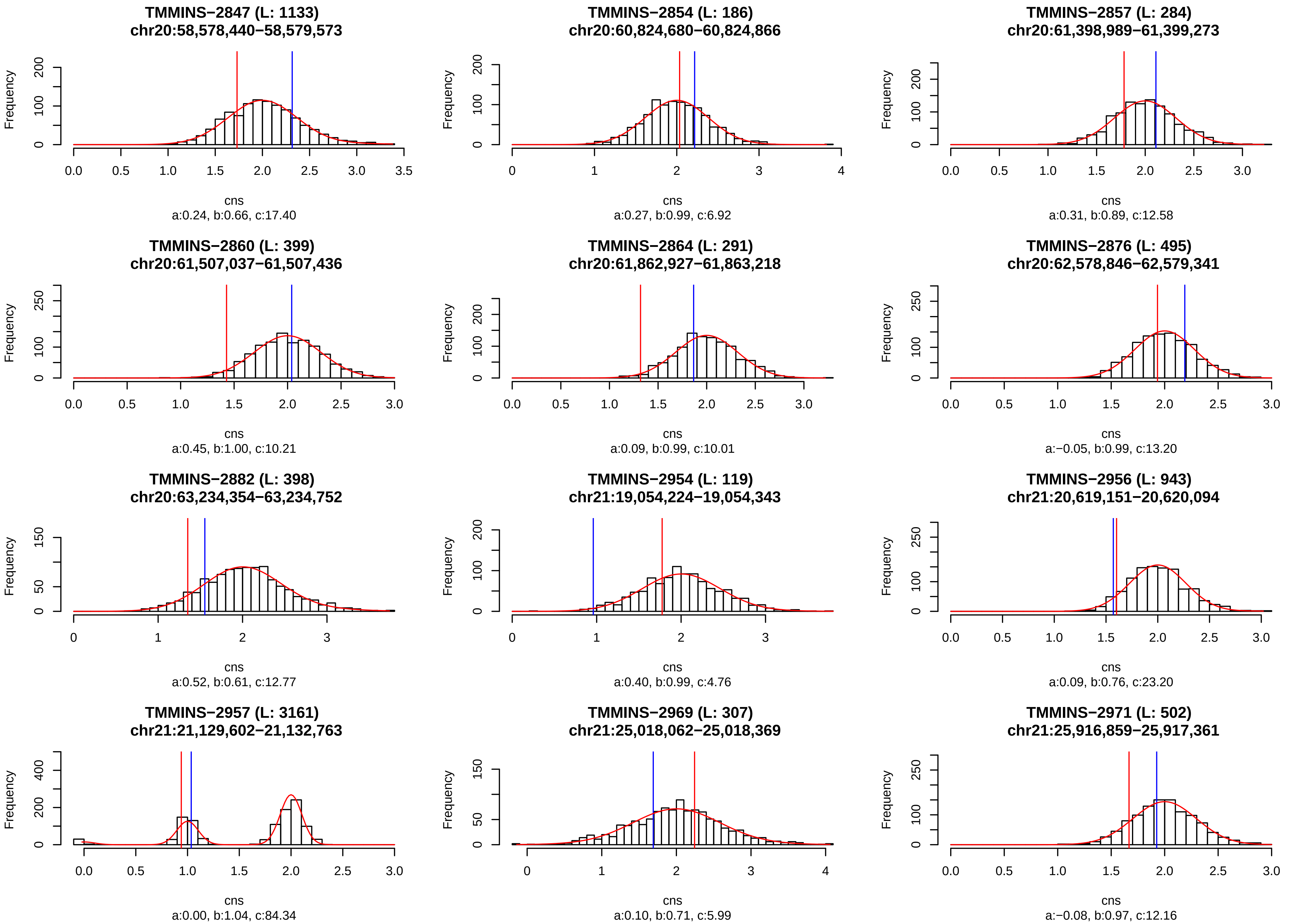
TMMINS−2847 (L: 1133)
chr20:58,578,440−58,579,573
Frequency
cns
a:0.24, b:0.66, c:17.40
TMMINS−2854 (L: 186)
chr20:60,824,680−60,824,866
Frequency
cns
a:0.27, b:0.99, c:6.92
TMMINS−2857 (L: 284)
chr20:61,398,989−61,399,273
Frequency
cns
a:0.31, b:0.89, c:12.58
TMMINS−2860 (L: 399)
chr20:61,507,037−61,507,436
Frequency
cns
a:0.45, b:1.00, c:10.21
TMMINS−2864 (L: 291)
chr20:61,862,927−61,863,218
Frequency
cns
a:0.09, b:0.99, c:10.01
TMMINS−2876 (L: 495)
chr20:62,578,846−62,579,341
Frequency
cns
a:−0.05, b:0.99, c:13.20
TMMINS−2882 (L: 398)
chr20:63,234,354−63,234,752
Frequency
cns
a:0.52, b:0.61, c:12.77
TMMINS−2954 (L: 119)
chr21:19,054,224−19,054,343
Frequency
cns
a:0.40, b:0.99, c:4.76
TMMINS−2956 (L: 943)
chr21:20,619,151−20,620,094
Frequency
cns
a:0.09, b:0.76, c:23.20
TMMINS−2957 (L: 3161)
chr21:21,129,602−21,132,763
Frequency
cns
a:0.00, b:1.04, c:84.34
TMMINS−2969 (L: 307)
chr21:25,018,062−25,018,369
Frequency
cns
a:0.10, b:0.71, c:5.99
TMMINS−2971 (L: 502)
chr21:25,916,859−25,917,361
Frequency
cns
a:−0.08, b:0.97, c:12.16

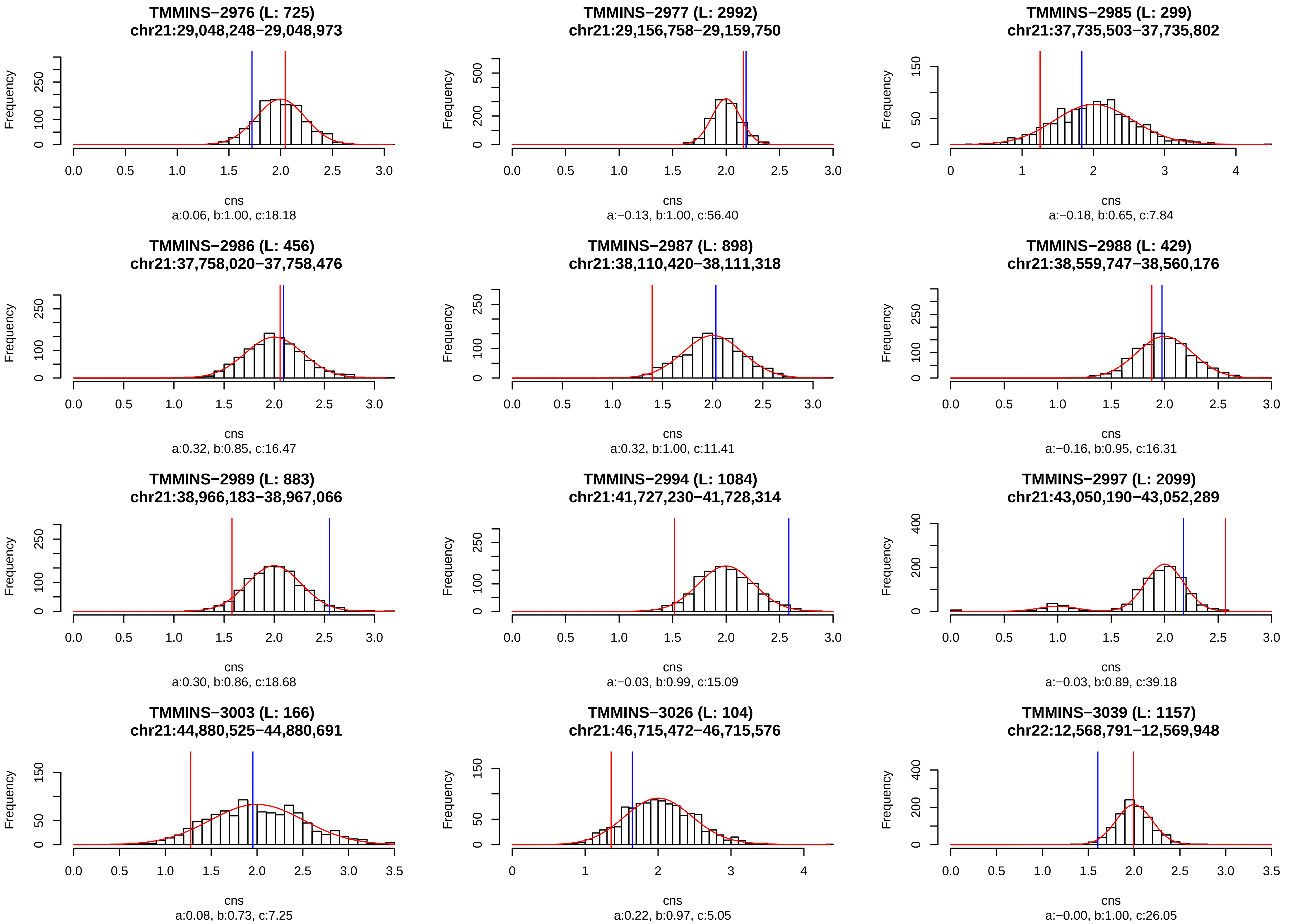
TMMINS−2976 (L: 725)
chr21:29,048,248−29,048,973
Frequency
cns
a:0.06, b:1.00, c:18.18
TMMINS−2977 (L: 2992)
chr21:29,156,758−29,159,750
Frequency
cns
a:−0.13, b:1.00, c:56.40
TMMINS−2985 (L: 299)
chr21:37,735,503−37,735,802
Frequency
cns
a:−0.18, b:0.65, c:7.84
TMMINS−2986 (L: 456)
chr21:37,758,020−37,758,476
Frequency
cns
a:0.32, b:0.85, c:16.47
TMMINS−2987 (L: 898)
chr21:38,110,420−38,111,318
Frequency
cns
a:0.32, b:1.00, c:11.41
TMMINS−2988 (L: 429)
chr21:38,559,747−38,560,176
Frequency
cns
a:−0.16, b:0.95, c:16.31
TMMINS−2989 (L: 883)
chr21:38,966,183−38,967,066
Frequency
cns
a:0.30, b:0.86, c:18.68
TMMINS−2994 (L: 1084)
chr21:41,727,230−41,728,314
Frequency
cns
a:−0.03, b:0.99, c:15.09
TMMINS−2997 (L: 2099)
chr21:43,050,190−43,052,289
Frequency
cns
a:−0.03, b:0.89, c:39.18
TMMINS−3003 (L: 166)
chr21:44,880,525−44,880,691
Frequency
cns
a:0.08, b:0.73, c:7.25
TMMINS−3026 (L: 104)
chr21:46,715,472−46,715,576
Frequency
cns
a:0.22, b:0.97, c:5.05
TMMINS−3039 (L: 1157)
chr22:12,568,791−12,569,948
Frequency
cns
a:−0.00, b:1.00, c:26.05

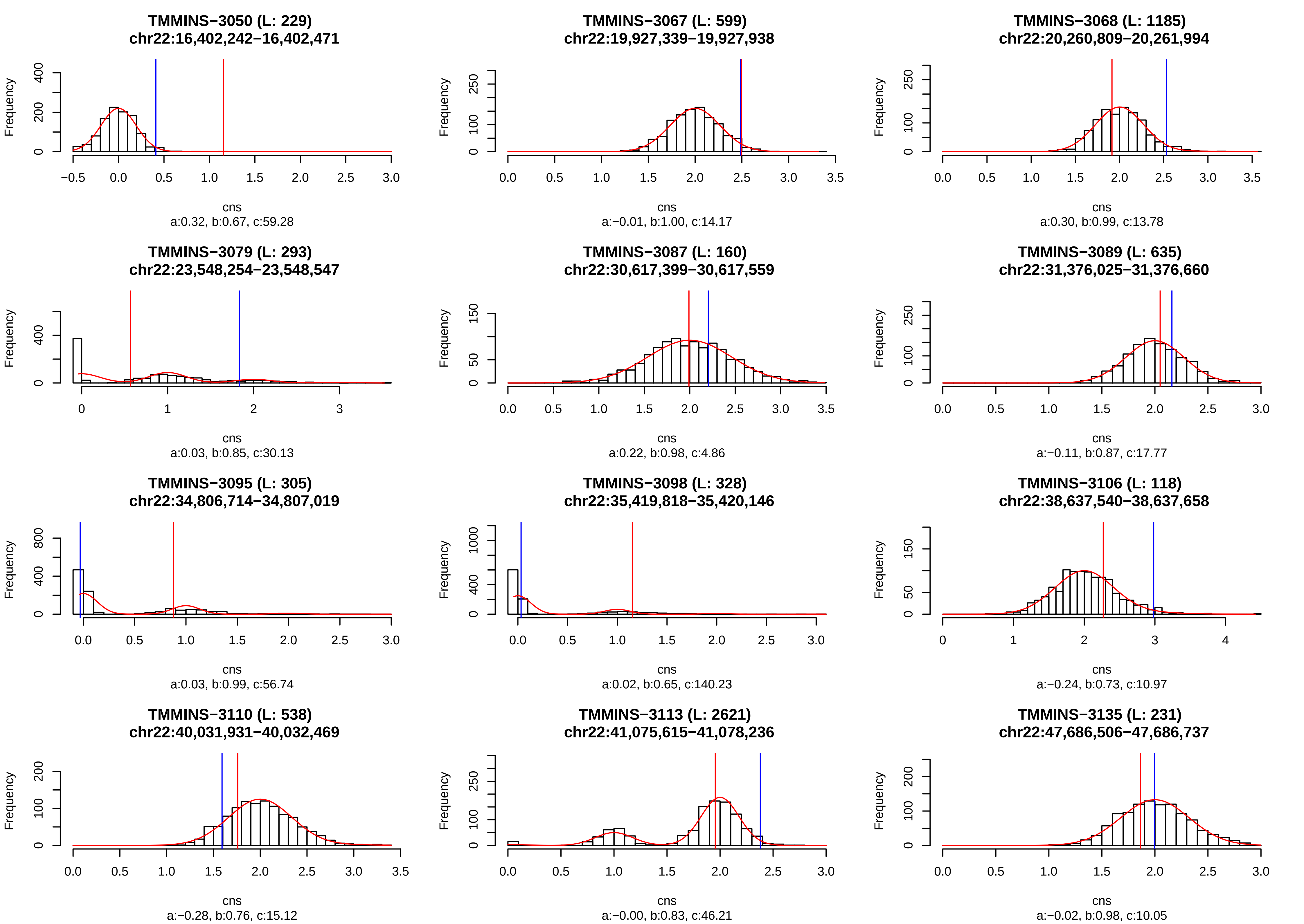
TMMINS−3050 (L: 229)
chr22:16,402,242−16,402,471
Frequency
cns
a:0.32, b:0.67, c:59.28
TMMINS−3067 (L: 599)
chr22:19,927,339−19,927,938
Frequency
cns
a:−0.01, b:1.00, c:14.17
TMMINS−3068 (L: 1185)
chr22:20,260,809−20,261,994
Frequency
cns
a:0.30, b:0.99, c:13.78
TMMINS−3079 (L: 293)
chr22:23,548,254−23,548,547
Frequency
cns
a:0.03, b:0.85, c:30.13
TMMINS−3087 (L: 160)
chr22:30,617,399−30,617,559
Frequency
cns
a:0.22, b:0.98, c:4.86
TMMINS−3089 (L: 635)
chr22:31,376,025−31,376,660
Frequency
cns
a:−0.11, b:0.87, c:17.77
TMMINS−3095 (L: 305)
chr22:34,806,714−34,807,019
Frequency
cns
a:0.03, b:0.99, c:56.74
TMMINS−3098 (L: 328)
chr22:35,419,818−35,420,146
Frequency
cns
a:0.02, b:0.65, c:140.23
TMMINS−3106 (L: 118)
chr22:38,637,540−38,637,658
Frequency
cns
a:−0.24, b:0.73, c:10.97
TMMINS−3110 (L: 538)
chr22:40,031,931−40,032,469
Frequency
cns
a:−0.28, b:0.76, c:15.12
TMMINS−3113 (L: 2621)
chr22:41,075,615−41,078,236
Frequency
cns
a:−0.00, b:0.83, c:46.21
TMMINS−3135 (L: 231)
chr22:47,686,506−47,686,737
Frequency
cns
a:−0.02, b:0.98, c:10.05

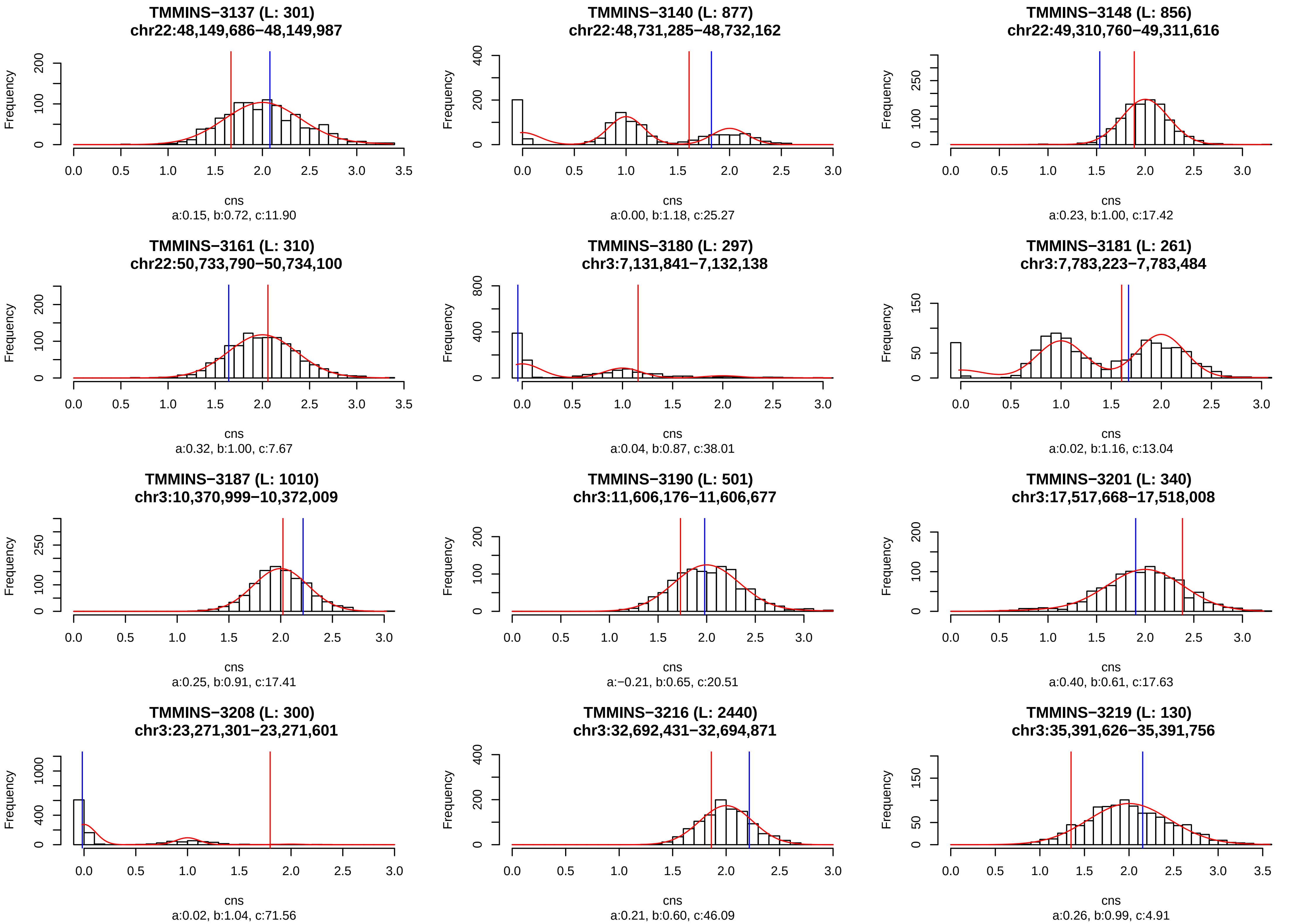
TMMINS−3137 (L: 301)
chr22:48,149,686−48,149,987
a:0.15, b:0.72, c:11.90
TMMINS−3140 (L: 877)
chr22:48,731,285−48,732,162
a:0.00, b:1.18, c:25.27
TMMINS−3148 (L: 856)
chr22:49,310,760−49,311,616
a:0.23, b:1.00, c:17.42
TMMINS−3161 (L: 310)
chr22:50,733,790−50,734,100
a:0.32, b:1.00, c:7.67
TMMINS−3180 (L: 297)
chr3:7,131,841−7,132,138
a:0.04, b:0.87, c:38.01
TMMINS−3181 (L: 261)
chr3:7,783,223−7,783,484
a:0.02, b:1.16, c:13.04
TMMINS−3187 (L: 1010)
chr3:10,370,999−10,372,009
a:0.25, b:0.91, c:17.41
TMMINS−3190 (L: 501)
chr3:11,606,176−11,606,677
a:−0.21, b:0.65, c:20.51
TMMINS−3201 (L: 340)
chr3:17,517,668−17,518,008
a:0.40, b:0.61, c:17.63
TMMINS−3208 (L: 300)
chr3:23,271,301−23,271,601
a:0.02, b:1.04, c:71.56
TMMINS−3216 (L: 2440)
chr3:32,692,431−32,694,871
a:0.21, b:0.60, c:46.09
TMMINS−3219 (L: 130)
chr3:35,391,626−35,391,756
a:0.26, b:0.99, c:4.91
Frequency
cns

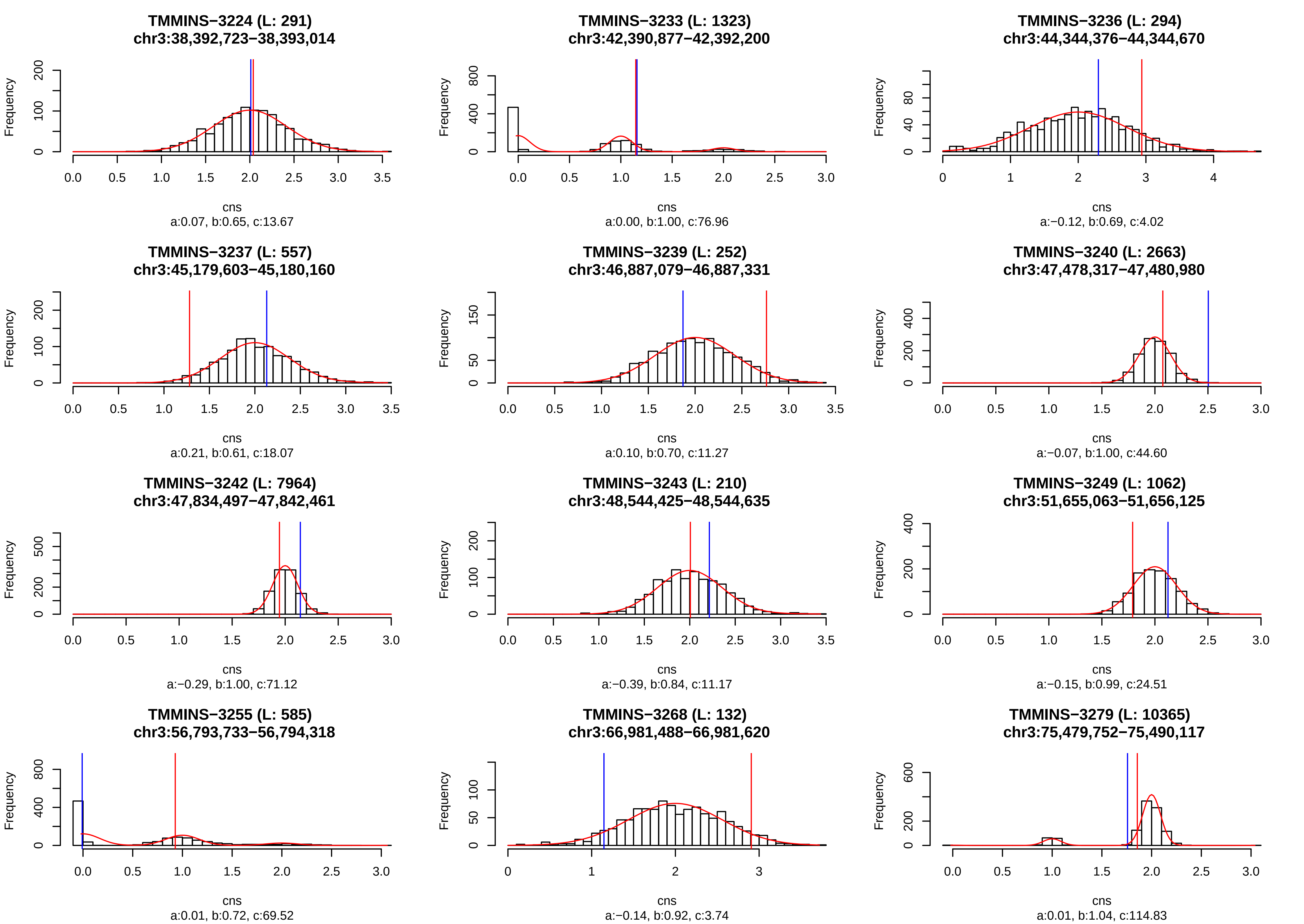
TMMINS−3224 (L: 291)
chr3:38,392,723−38,393,014
Frequency
cns
a:0.07, b:0.65, c:13.67
TMMINS−3233 (L: 1323)
chr3:42,390,877−42,392,200
Frequency
cns
a:0.00, b:1.00, c:76.96
TMMINS−3236 (L: 294)
chr3:44,344,376−44,344,670
Frequency
cns
a:−0.12, b:0.69, c:4.02
TMMINS−3237 (L: 557)
chr3:45,179,603−45,180,160
Frequency
cns
a:0.21, b:0.61, c:18.07
TMMINS−3239 (L: 252)
chr3:46,887,079−46,887,331
Frequency
cns
a:0.10, b:0.70, c:11.27
TMMINS−3240 (L: 2663)
chr3:47,478,317−47,480,980
Frequency
cns
a:−0.07, b:1.00, c:44.60
TMMINS−3242 (L: 7964)
chr3:47,834,497−47,842,461
Frequency
cns
a:−0.29, b:1.00, c:71.12
TMMINS−3243 (L: 210)
chr3:48,544,425−48,544,635
Frequency
cns
a:−0.39, b:0.84, c:11.17
TMMINS−3249 (L: 1062)
chr3:51,655,063−51,656,125
Frequency
cns
a:−0.15, b:0.99, c:24.51
TMMINS−3255 (L: 585)
chr3:56,793,733−56,794,318
Frequency
cns
a:0.01, b:0.72, c:69.52
TMMINS−3268 (L: 132)
chr3:66,981,488−66,981,620
Frequency
cns
a:−0.14, b:0.92, c:3.74
TMMINS−3279 (L: 10365)
chr3:75,479,752−75,490,117
Frequency
cns
a:0.01, b:1.04, c:114.83

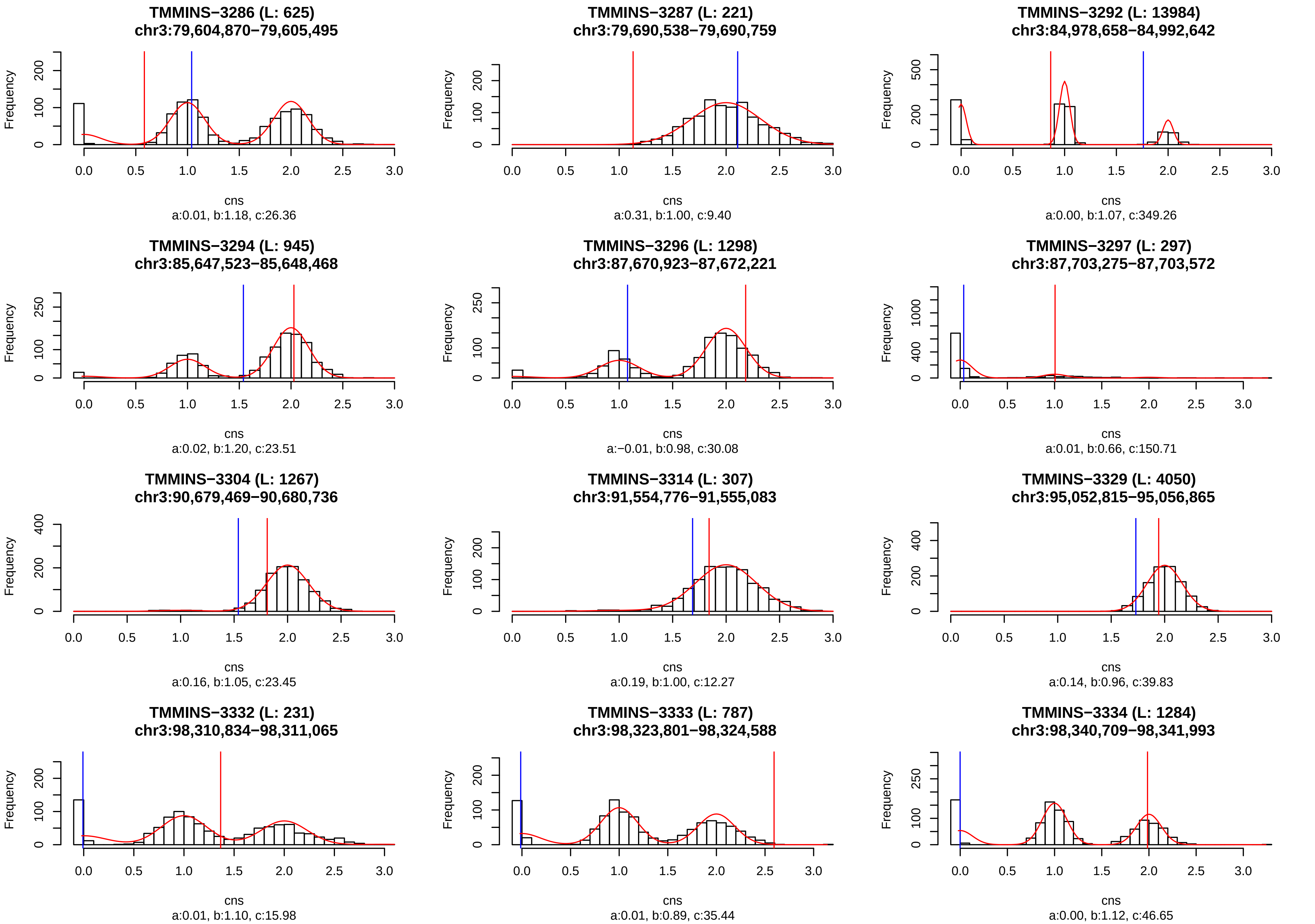

TMMINS−3286 (L: 625)
chr3:79,604,870−79,605,495
Frequency
cns
a:0.01, b:1.18, c:26.36
TMMINS−3287 (L: 221)
chr3:79,690,538−79,690,759
Frequency
cns
a:0.31, b:1.00, c:9.40
TMMINS−3292 (L: 13984)
chr3:84,978,658−84,992,642
Frequency
cns
a:0.00, b:1.07, c:349.26
TMMINS−3294 (L: 945)
chr3:85,647,523−85,648,468
Frequency
cns
a:0.02, b:1.20, c:23.51
TMMINS−3296 (L: 1298)
chr3:87,670,923−87,672,221
Frequency
cns
a:−0.01, b:0.98, c:30.08
TMMINS−3297 (L: 297)
chr3:87,703,275−87,703,572
Frequency
cns
a:0.01, b:0.66, c:150.71
TMMINS−3304 (L: 1267)
chr3:90,679,469−90,680,736
Frequency
cns
a:0.16, b:1.05, c:23.45
TMMINS−3314 (L: 307)
chr3:91,554,776−91,555,083
Frequency
cns
a:0.19, b:1.00, c:12.27
TMMINS−3329 (L: 4050)
chr3:95,052,815−95,056,865
Frequency
cns
a:0.14, b:0.96, c:39.83
TMMINS−3332 (L: 231)
chr3:98,310,834−98,311,065
Frequency
cns
a:0.01, b:1.10, c:15.98
TMMINS−3333 (L: 787)
chr3:98,323,801−98,324,588
Frequency
cns
a:0.01, b:0.89, c:35.44
TMMINS−3334 (L: 1284)
chr3:98,340,709−98,341,993
Frequency
cns
a:0.00, b:1.12, c:46.65

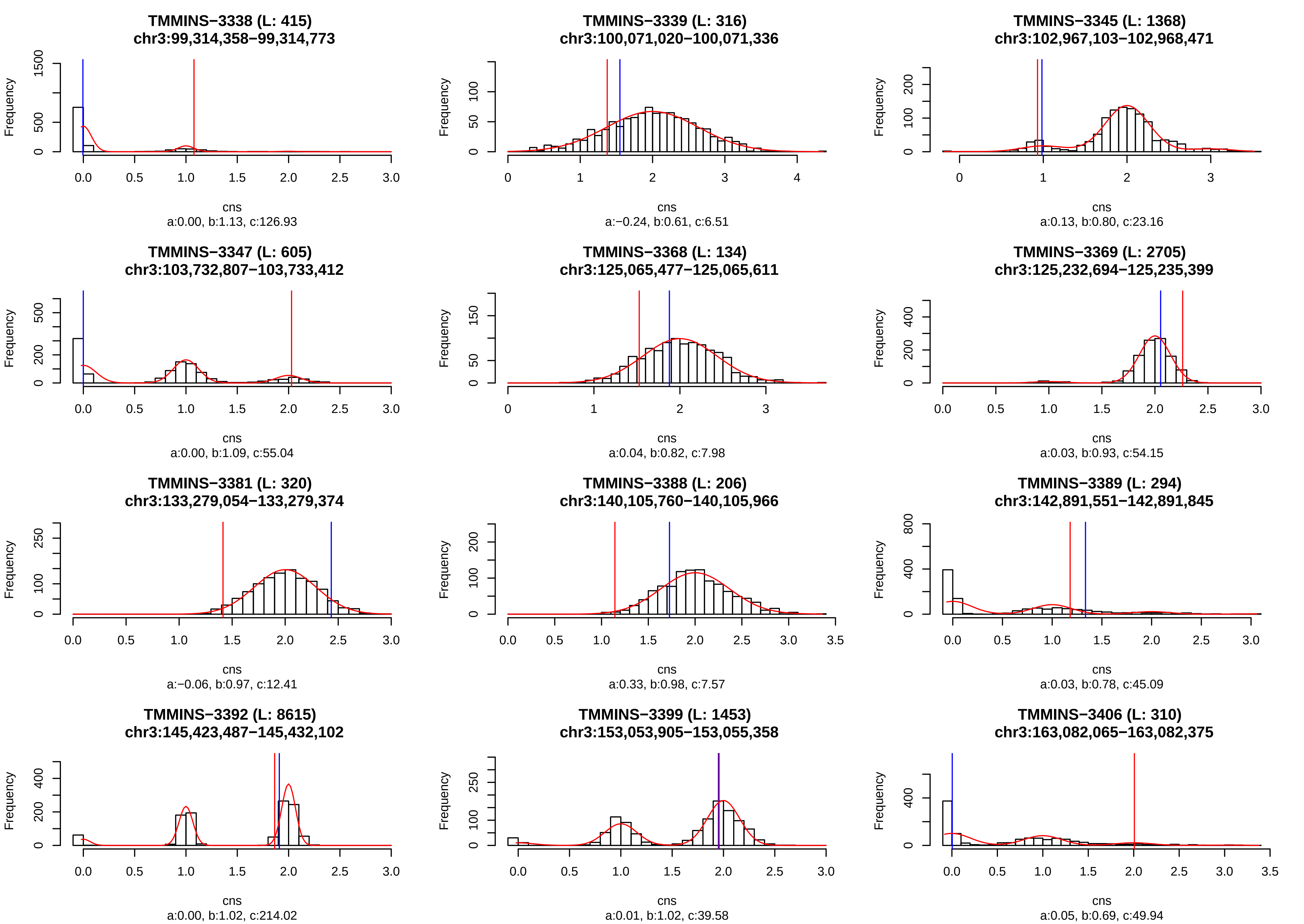
TMMINS−3338 (L: 415)
chr3:99,314,358−99,314,773
Frequency
cns
a:0.00, b:1.13, c:126.93
TMMINS−3339 (L: 316)
chr3:100,071,020−100,071,336
Frequency
cns
a:−0.24, b:0.61, c:6.51
TMMINS−3345 (L: 1368)
chr3:102,967,103−102,968,471
Frequency
cns
a:0.13, b:0.80, c:23.16
TMMINS−3347 (L: 605)
chr3:103,732,807−103,733,412
Frequency
cns
a:0.00, b:1.09, c:55.04
TMMINS−3368 (L: 134)
chr3:125,065,477−125,065,611
Frequency
cns
a:0.04, b:0.82, c:7.98
TMMINS−3369 (L: 2705)
chr3:125,232,694−125,235,399
Frequency
cns
a:0.03, b:0.93, c:54.15
TMMINS−3381 (L: 320)
chr3:133,279,054−133,279,374
Frequency
cns
a:−0.06, b:0.97, c:12.41
TMMINS−3388 (L: 206)
chr3:140,105,760−140,105,966
Frequency
cns
a:0.33, b:0.98, c:7.57
TMMINS−3389 (L: 294)
chr3:142,891,551−142,891,845
Frequency
cns
a:0.03, b:0.78, c:45.09
TMMINS−3392 (L: 8615)
chr3:145,423,487−145,432,102
Frequency
cns
a:0.00, b:1.02, c:214.02
TMMINS−3399 (L: 1453)
chr3:153,053,905−153,055,358
Frequency
cns
a:0.01, b:1.02, c:39.58
TMMINS−3406 (L: 310)
chr3:163,082,065−163,082,375
Frequency
cns
a:0.05, b:0.69, c:49.94

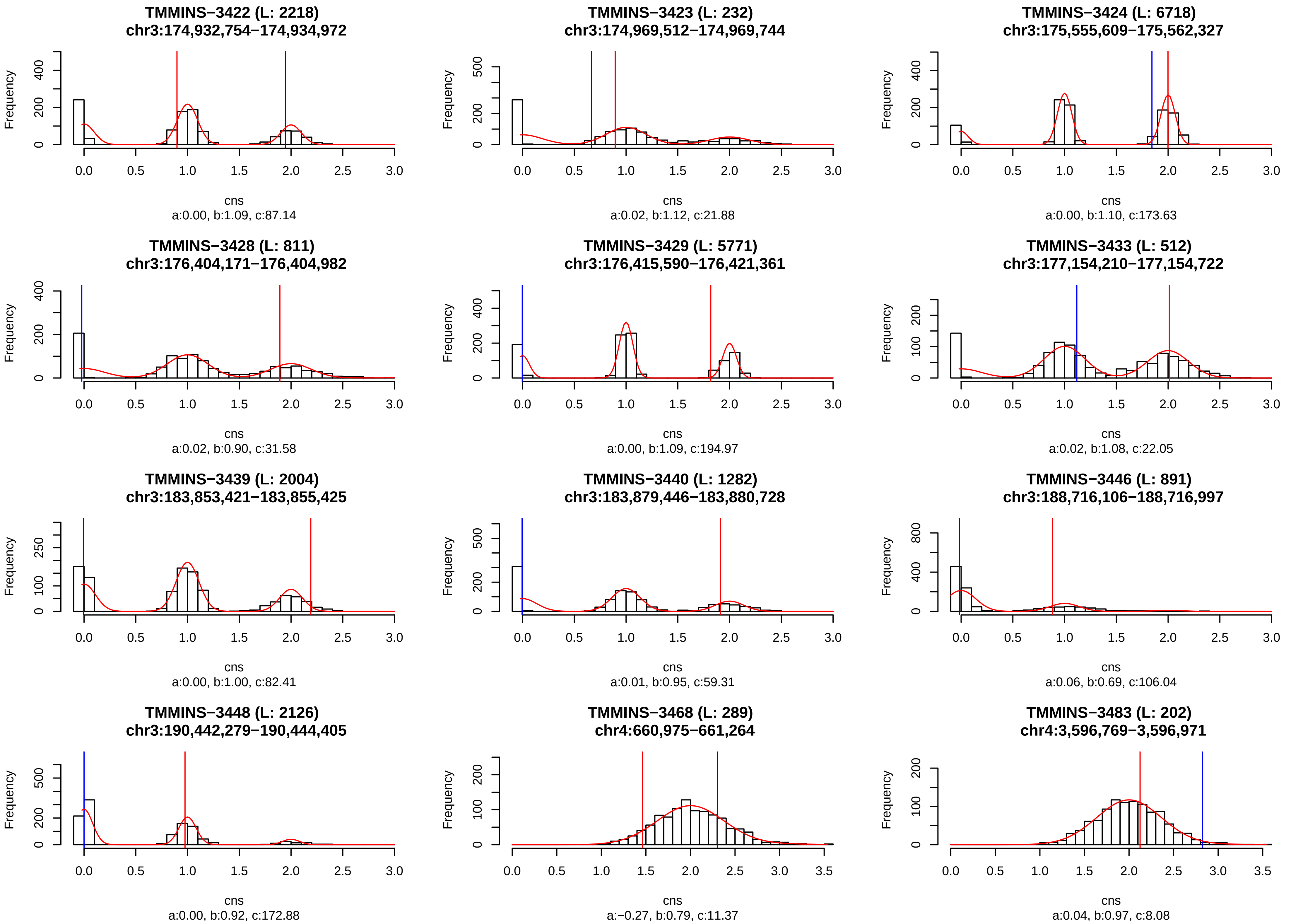
TMMINS−3422 (L: 2218)
chr3:174,932,754−174,934,972
Frequency
cns
a:0.00, b:1.09, c:87.14
TMMINS−3423 (L: 232)
chr3:174,969,512−174,969,744
Frequency
cns
a:0.02, b:1.12, c:21.88
TMMINS−3424 (L: 6718)
chr3:175,555,609−175,562,327
Frequency
cns
a:0.00, b:1.10, c:173.63
TMMINS−3428 (L: 811)
chr3:176,404,171−176,404,982
Frequency
cns
a:0.02, b:0.90, c:31.58
TMMINS−3429 (L: 5771)
chr3:176,415,590−176,421,361
Frequency
cns
a:0.00, b:1.09, c:194.97
TMMINS−3433 (L: 512)
chr3:177,154,210−177,154,722
Frequency
cns
a:0.02, b:1.08, c:22.05
TMMINS−3439 (L: 2004)
chr3:183,853,421−183,855,425
Frequency
cns
a:0.00, b:1.00, c:82.41
TMMINS−3440 (L: 1282)
chr3:183,879,446−183,880,728
Frequency
cns
a:0.01, b:0.95, c:59.31
TMMINS−3446 (L: 891)
chr3:188,716,106−188,716,997
Frequency
cns
a:0.06, b:0.69, c:106.04
TMMINS−3448 (L: 2126)
chr3:190,442,279−190,444,405
Frequency
cns
a:0.00, b:0.92, c:172.88
TMMINS−3468 (L: 289)
chr4:660,975−661,264
Frequency
cns
a:−0.27, b:0.79, c:11.37
TMMINS−3483 (L: 202)
chr4:3,596,769−3,596,971
Frequency
cns
a:0.04, b:0.97, c:8.08

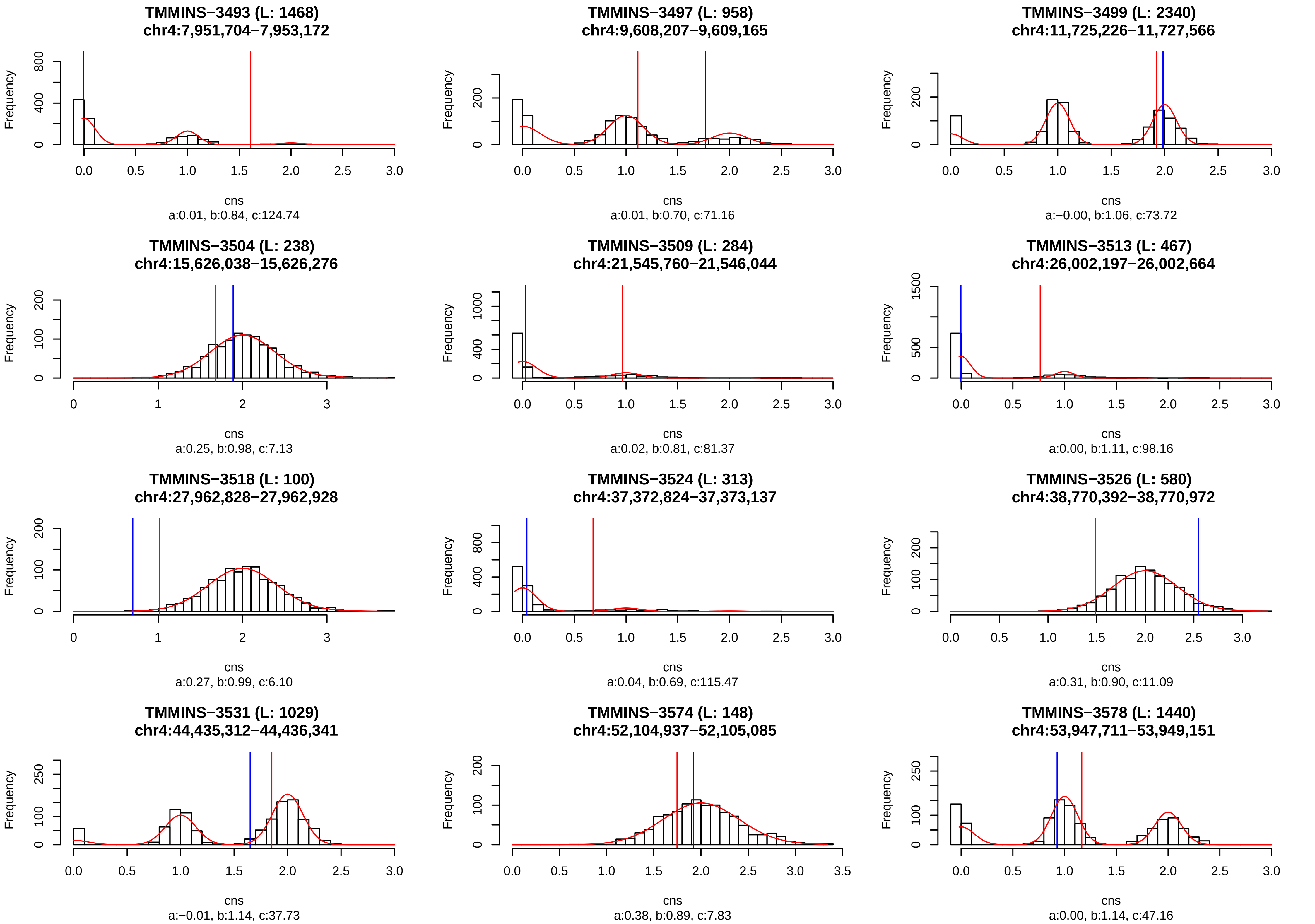
TMMINS−3493 (L: 1468)
chr4:7,951,704−7,953,172
Frequency
cns
a:0.01, b:0.84, c:124.74
TMMINS−3497 (L: 958)
chr4:9,608,207−9,609,165
Frequency
cns
a:0.01, b:0.70, c:71.16
TMMINS−3499 (L: 2340)
chr4:11,725,226−11,727,566
Frequency
cns
a:−0.00, b:1.06, c:73.72
TMMINS−3504 (L: 238)
chr4:15,626,038−15,626,276
Frequency
cns
a:0.25, b:0.98, c:7.13
TMMINS−3509 (L: 284)
chr4:21,545,760−21,546,044
Frequency
cns
a:0.02, b:0.81, c:81.37
TMMINS−3513 (L: 467)
chr4:26,002,197−26,002,664
Frequency
cns
a:0.00, b:1.11, c:98.16
TMMINS−3518 (L: 100)
chr4:27,962,828−27,962,928
Frequency
cns
a:0.27, b:0.99, c:6.10
TMMINS−3524 (L: 313)
chr4:37,372,824−37,373,137
Frequency
cns
a:0.04, b:0.69, c:115.47
TMMINS−3526 (L: 580)
chr4:38,770,392−38,770,972
Frequency
cns
a:0.31, b:0.90, c:11.09
TMMINS−3531 (L: 1029)
chr4:44,435,312−44,436,341
Frequency
cns
a:−0.01, b:1.14, c:37.73
TMMINS−3574 (L: 148)
chr4:52,104,937−52,105,085
Frequency
cns
a:0.38, b:0.89, c:7.83
TMMINS−3578 (L: 1440)
chr4:53,947,711−53,949,151
Frequency
cns
a:0.00, b:1.14, c:47.16

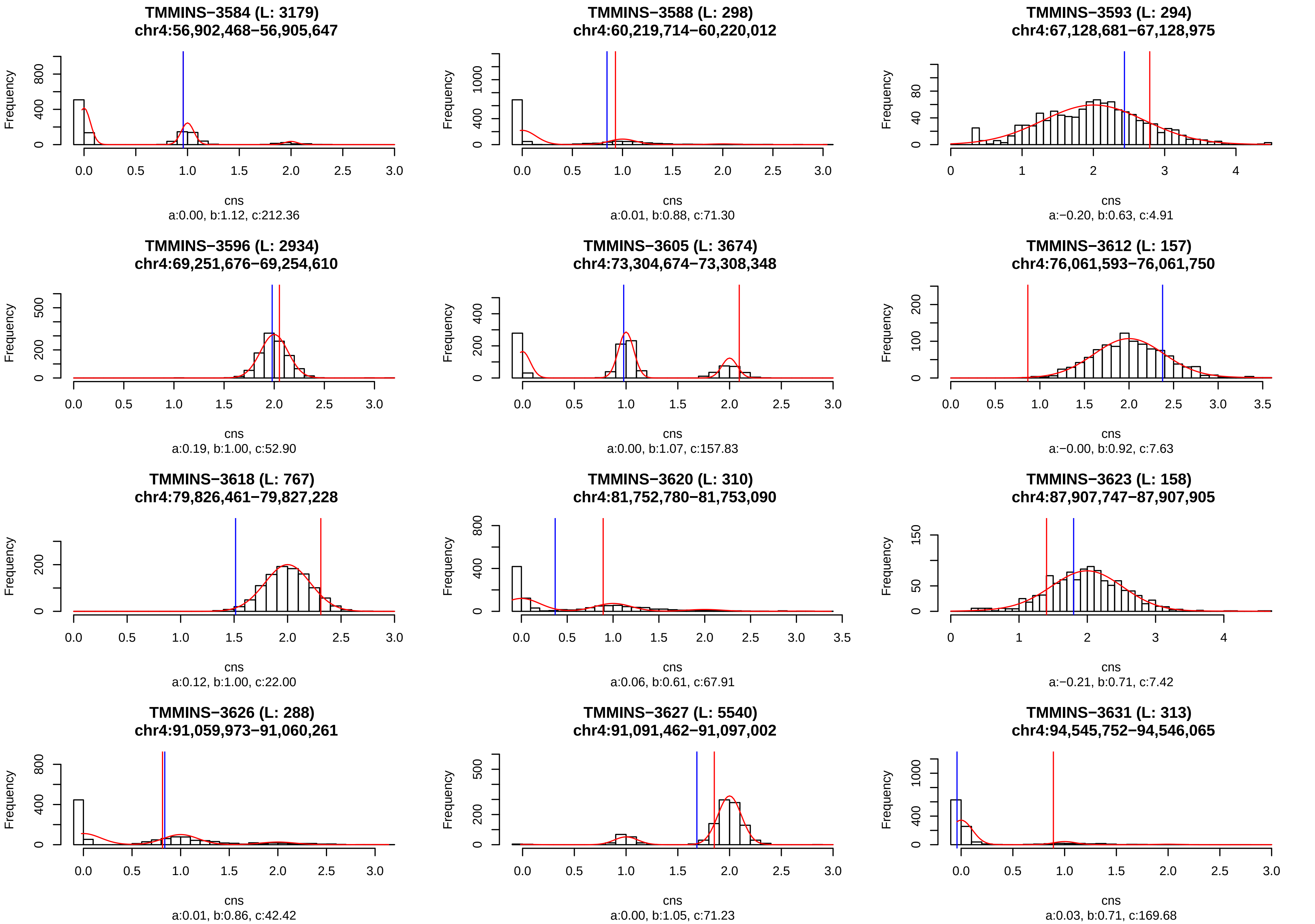
TMMINS−3584 (L: 3179)
chr4:56,902,468−56,905,647
Frequency
cns
a:0.00, b:1.12, c:212.36
TMMINS−3588 (L: 298)
chr4:60,219,714−60,220,012
Frequency
cns
a:0.01, b:0.88, c:71.30
TMMINS−3593 (L: 294)
chr4:67,128,681−67,128,975
Frequency
cns
a:−0.20, b:0.63, c:4.91
TMMINS−3596 (L: 2934)
chr4:69,251,676−69,254,610
Frequency
cns
a:0.19, b:1.00, c:52.90
TMMINS−3605 (L: 3674)
chr4:73,304,674−73,308,348
Frequency
cns
a:0.00, b:1.07, c:157.83
TMMINS−3612 (L: 157)
chr4:76,061,593−76,061,750
Frequency
cns
a:−0.00, b:0.92, c:7.63
TMMINS−3618 (L: 767)
chr4:79,826,461−79,827,228
Frequency
cns
a:0.12, b:1.00, c:22.00
TMMINS−3620 (L: 310)
chr4:81,752,780−81,753,090
Frequency
cns
a:0.06, b:0.61, c:67.91
TMMINS−3623 (L: 158)
chr4:87,907,747−87,907,905
Frequency
cns
a:−0.21, b:0.71, c:7.42
TMMINS−3626 (L: 288)
chr4:91,059,973−91,060,261
Frequency
cns
a:0.01, b:0.86, c:42.42
TMMINS−3627 (L: 5540)
chr4:91,091,462−91,097,002
Frequency
cns
a:0.00, b:1.05, c:71.23
TMMINS−3631 (L: 313)
chr4:94,545,752−94,546,065
Frequency
cns
a:0.03, b:0.71, c:169.68

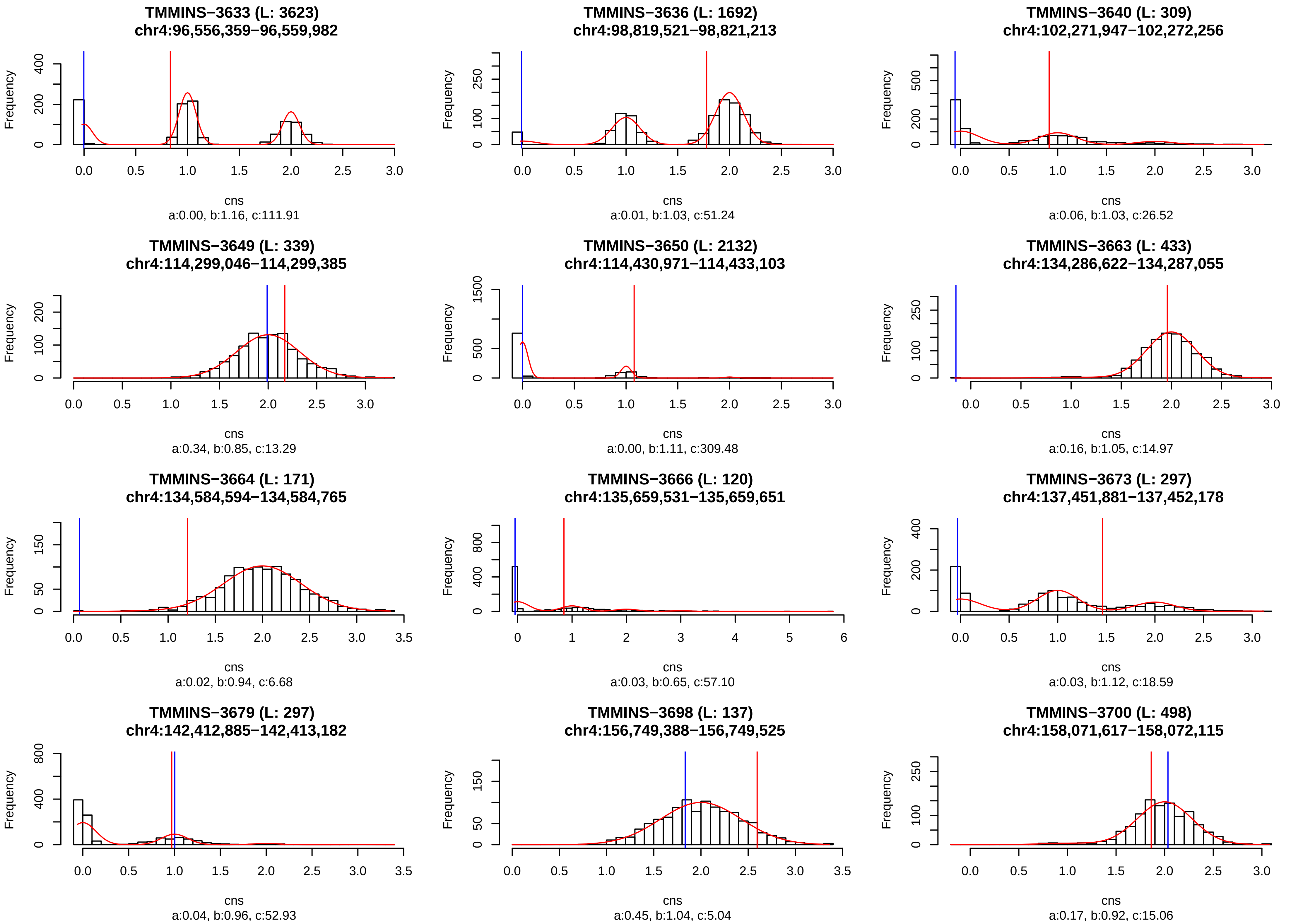
TMMINS−3633 (L: 3623)
chr4:96,556,359−96,559,982
Frequency
cns
a:0.00, b:1.16, c:111.91
TMMINS−3636 (L: 1692)
chr4:98,819,521−98,821,213
Frequency
cns
a:0.01, b:1.03, c:51.24
TMMINS−3640 (L: 309)
chr4:102,271,947−102,272,256
Frequency
cns
a:0.06, b:1.03, c:26.52
TMMINS−3649 (L: 339)
chr4:114,299,046−114,299,385
Frequency
cns
a:0.34, b:0.85, c:13.29
TMMINS−3650 (L: 2132)
chr4:114,430,971−114,433,103
Frequency
cns
a:0.00, b:1.11, c:309.48
TMMINS−3663 (L: 433)
chr4:134,286,622−134,287,055
Frequency
cns
a:0.16, b:1.05, c:14.97
TMMINS−3664 (L: 171)
chr4:134,584,594−134,584,765
Frequency
cns
a:0.02, b:0.94, c:6.68
TMMINS−3666 (L: 120)
chr4:135,659,531−135,659,651
Frequency
cns
a:0.03, b:0.65, c:57.10
TMMINS−3673 (L: 297)
chr4:137,451,881−137,452,178
Frequency
cns
a:0.03, b:1.12, c:18.59
TMMINS−3679 (L: 297)
chr4:142,412,885−142,413,182
Frequency
cns
a:0.04, b:0.96, c:52.93
TMMINS−3698 (L: 137)
chr4:156,749,388−156,749,525
Frequency
cns
a:0.45, b:1.04, c:5.04
TMMINS−3700 (L: 498)
chr4:158,071,617−158,072,115
Frequency
cns
a:0.17, b:0.92, c:15.06

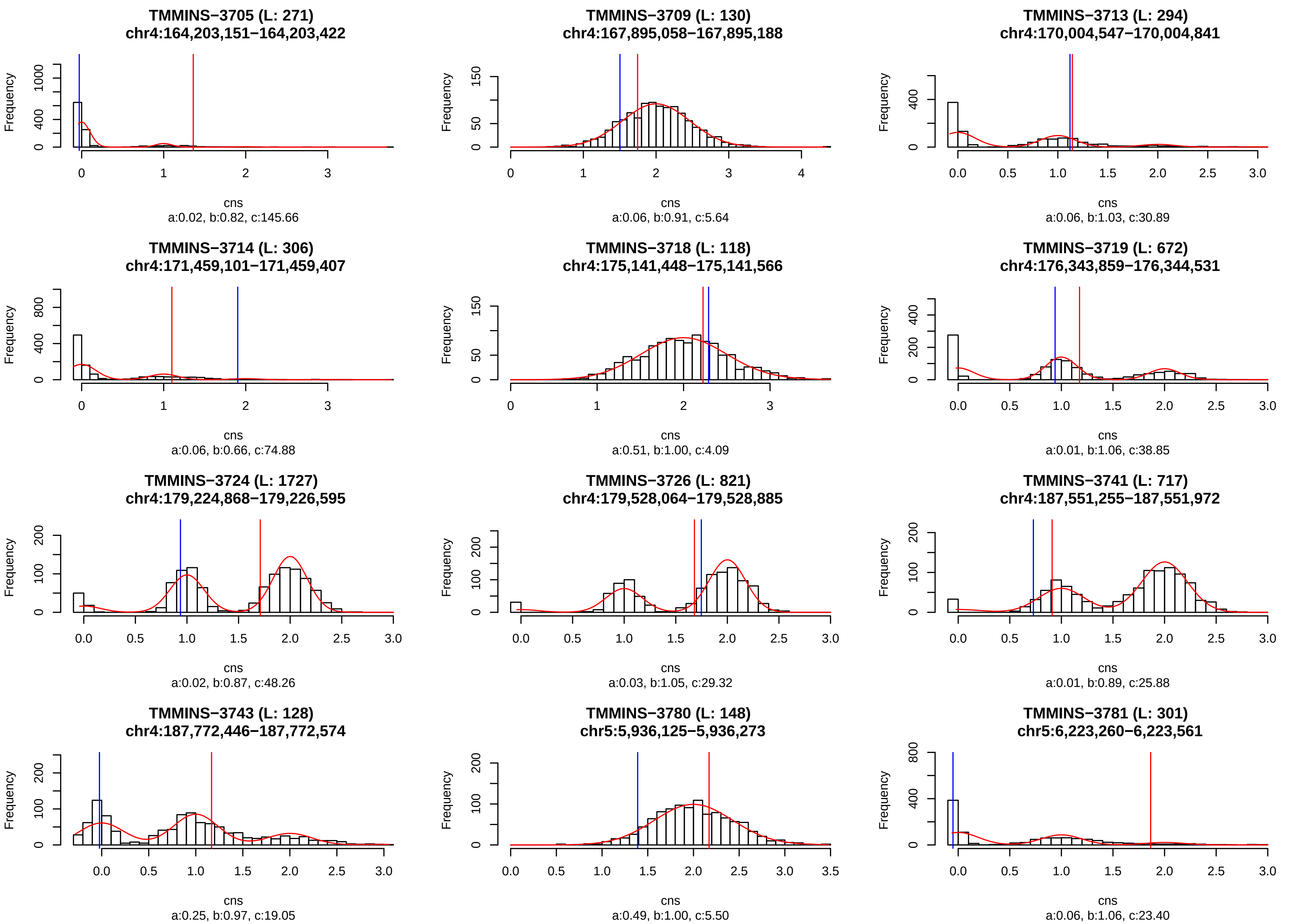
TMMINS−3705 (L: 271)
chr4:164,203,151−164,203,422
Frequency
cns
a:0.02, b:0.82, c:145.66
TMMINS−3709 (L: 130)
chr4:167,895,058−167,895,188
Frequency
cns
a:0.06, b:0.91, c:5.64
TMMINS−3713 (L: 294)
chr4:170,004,547−170,004,841
Frequency
cns
a:0.06, b:1.03, c:30.89
TMMINS−3714 (L: 306)
chr4:171,459,101−171,459,407
Frequency
cns
a:0.06, b:0.66, c:74.88
TMMINS−3718 (L: 118)
chr4:175,141,448−175,141,566
Frequency
cns
a:0.51, b:1.00, c:4.09
TMMINS−3719 (L: 672)
chr4:176,343,859−176,344,531
Frequency
cns
a:0.01, b:1.06, c:38.85
TMMINS−3724 (L: 1727)
chr4:179,224,868−179,226,595
Frequency
cns
a:0.02, b:0.87, c:48.26
TMMINS−3726 (L: 821)
chr4:179,528,064−179,528,885
Frequency
cns
a:0.03, b:1.05, c:29.32
TMMINS−3741 (L: 717)
chr4:187,551,255−187,551,972
Frequency
cns
a:0.01, b:0.89, c:25.88
TMMINS−3743 (L: 128)
chr4:187,772,446−187,772,574
Frequency
cns
a:0.25, b:0.97, c:19.05
TMMINS−3780 (L: 148)
chr5:5,936,125−5,936,273
Frequency
cns
a:0.49, b:1.00, c:5.50
TMMINS−3781 (L: 301)
chr5:6,223,260−6,223,561
Frequency
cns
a:0.06, b:1.06, c:23.40

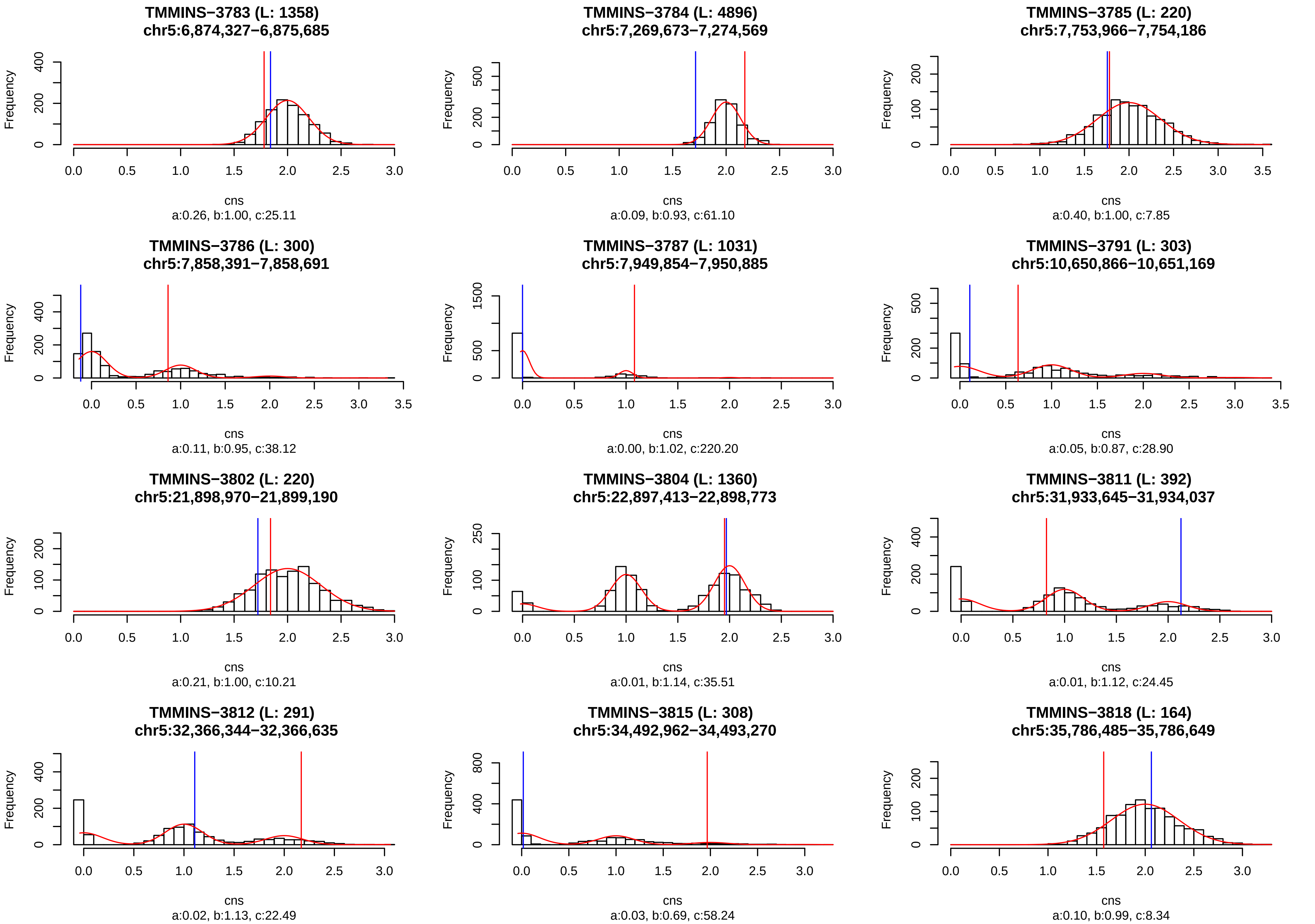
TMMINS−3783 (L: 1358)
chr5:6,874,327−6,875,685
Frequency
cns
a:0.26, b:1.00, c:25.11
TMMINS−3784 (L: 4896)
chr5:7,269,673−7,274,569
Frequency
cns
a:0.09, b:0.93, c:61.10
TMMINS−3785 (L: 220)
chr5:7,753,966−7,754,186
Frequency
cns
a:0.40, b:1.00, c:7.85
TMMINS−3786 (L: 300)
chr5:7,858,391−7,858,691
Frequency
cns
a:0.11, b:0.95, c:38.12
TMMINS−3787 (L: 1031)
chr5:7,949,854−7,950,885
Frequency
cns
a:0.00, b:1.02, c:220.20
TMMINS−3791 (L: 303)
chr5:10,650,866−10,651,169
Frequency
cns
a:0.05, b:0.87, c:28.90
TMMINS−3802 (L: 220)
chr5:21,898,970−21,899,190
Frequency
cns
a:0.21, b:1.00, c:10.21
TMMINS−3804 (L: 1360)
chr5:22,897,413−22,898,773
Frequency
cns
a:0.01, b:1.14, c:35.51
TMMINS−3811 (L: 392)
chr5:31,933,645−31,934,037
Frequency
cns
a:0.01, b:1.12, c:24.45
TMMINS−3812 (L: 291)
chr5:32,366,344−32,366,635
Frequency
cns
a:0.02, b:1.13, c:22.49
TMMINS−3815 (L: 308)
chr5:34,492,962−34,493,270
Frequency
cns
a:0.03, b:0.69, c:58.24
TMMINS−3818 (L: 164)
chr5:35,786,485−35,786,649
Frequency
cns
a:0.10, b:0.99, c:8.34

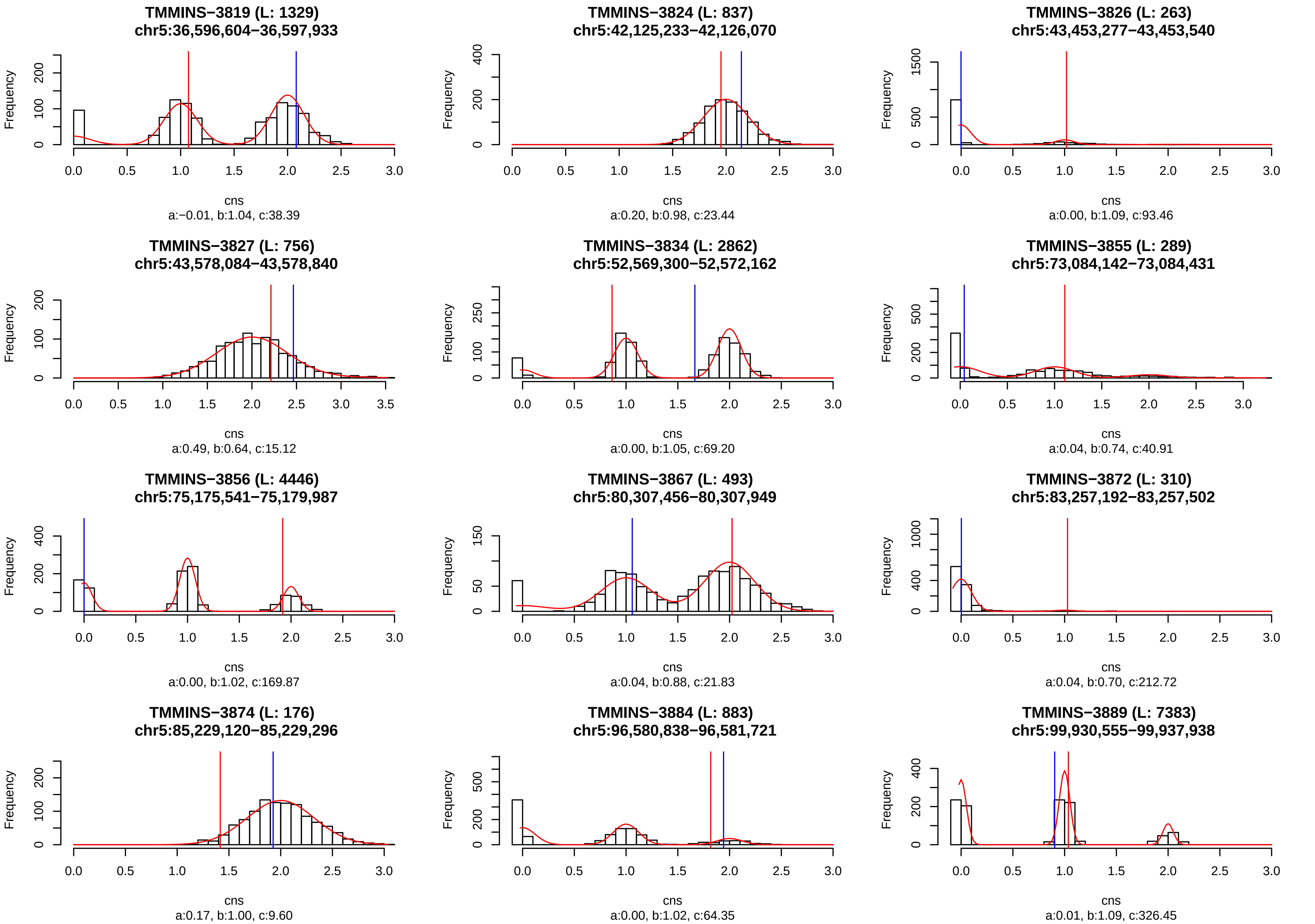
TMMINS−3819 (L: 1329)
chr5:36,596,604−36,597,933
Frequency
cns
a:−0.01, b:1.04, c:38.39
TMMINS−3824 (L: 837)
chr5:42,125,233−42,126,070
Frequency
cns
a:0.20, b:0.98, c:23.44
TMMINS−3826 (L: 263)
chr5:43,453,277−43,453,540
Frequency
cns
a:0.00, b:1.09, c:93.46
TMMINS−3827 (L: 756)
chr5:43,578,084−43,578,840
Frequency
cns
a:0.49, b:0.64, c:15.12
TMMINS−3834 (L: 2862)
chr5:52,569,300−52,572,162
Frequency
cns
a:0.00, b:1.05, c:69.20
TMMINS−3855 (L: 289)
chr5:73,084,142−73,084,431
Frequency
cns
a:0.04, b:0.74, c:40.91
TMMINS−3856 (L: 4446)
chr5:75,175,541−75,179,987
Frequency
cns
a:0.00, b:1.02, c:169.87
TMMINS−3867 (L: 493)
chr5:80,307,456−80,307,949
Frequency
cns
a:0.04, b:0.88, c:21.83
TMMINS−3872 (L: 310)
chr5:83,257,192−83,257,502
Frequency
cns
a:0.04, b:0.70, c:212.72
TMMINS−3874 (L: 176)
chr5:85,229,120−85,229,296
Frequency
cns
a:0.17, b:1.00, c:9.60
TMMINS−3884 (L: 883)
chr5:96,580,838−96,581,721
Frequency
cns
a:0.00, b:1.02, c:64.35
TMMINS−3889 (L: 7383)
chr5:99,930,555−99,937,938
Frequency
cns
a:0.01, b:1.09, c:326.45

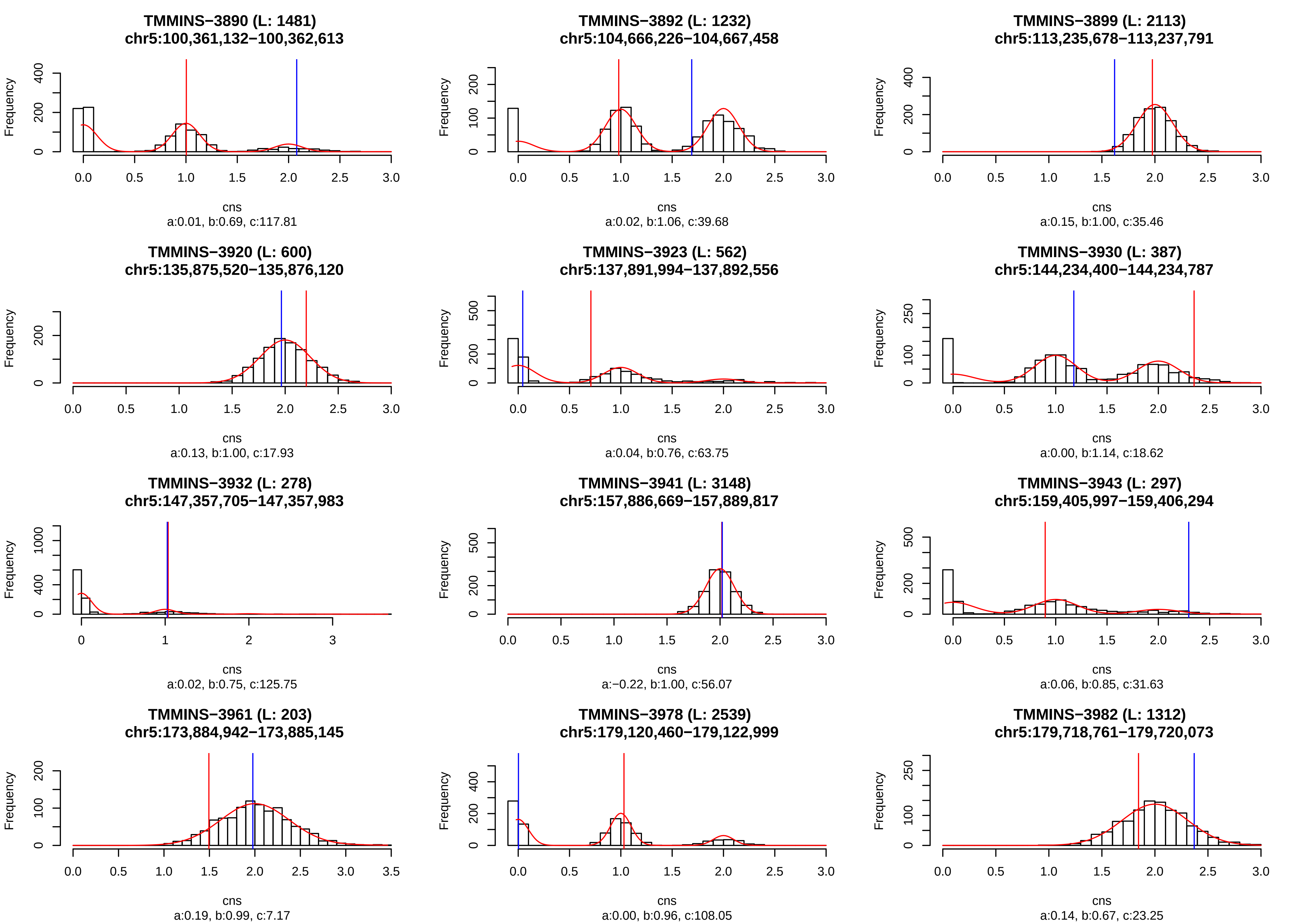
TMMINS−3890 (L: 1481)
chr5:100,361,132−100,362,613
Frequency
cns
a:0.01, b:0.69, c:117.81
TMMINS−3892 (L: 1232)
chr5:104,666,226−104,667,458
Frequency
cns
a:0.02, b:1.06, c:39.68
TMMINS−3899 (L: 2113)
chr5:113,235,678−113,237,791
Frequency
cns
a:0.15, b:1.00, c:35.46
TMMINS−3920 (L: 600)
chr5:135,875,520−135,876,120
Frequency
cns
a:0.13, b:1.00, c:17.93
TMMINS−3923 (L: 562)
chr5:137,891,994−137,892,556
Frequency
cns
a:0.04, b:0.76, c:63.75
TMMINS−3930 (L: 387)
chr5:144,234,400−144,234,787
Frequency
cns
a:0.00, b:1.14, c:18.62
TMMINS−3932 (L: 278)
chr5:147,357,705−147,357,983
Frequency
cns
a:0.02, b:0.75, c:125.75
TMMINS−3941 (L: 3148)
chr5:157,886,669−157,889,817
Frequency
cns
a:−0.22, b:1.00, c:56.07
TMMINS−3943 (L: 297)
chr5:159,405,997−159,406,294
Frequency
cns
a:0.06, b:0.85, c:31.63
TMMINS−3961 (L: 203)
chr5:173,884,942−173,885,145
Frequency
cns
a:0.19, b:0.99, c:7.17
TMMINS−3978 (L: 2539)
chr5:179,120,460−179,122,999
Frequency
cns
a:0.00, b:0.96, c:108.05
TMMINS−3982 (L: 1312)
chr5:179,718,761−179,720,073
Frequency
cns
a:0.14, b:0.67, c:23.25

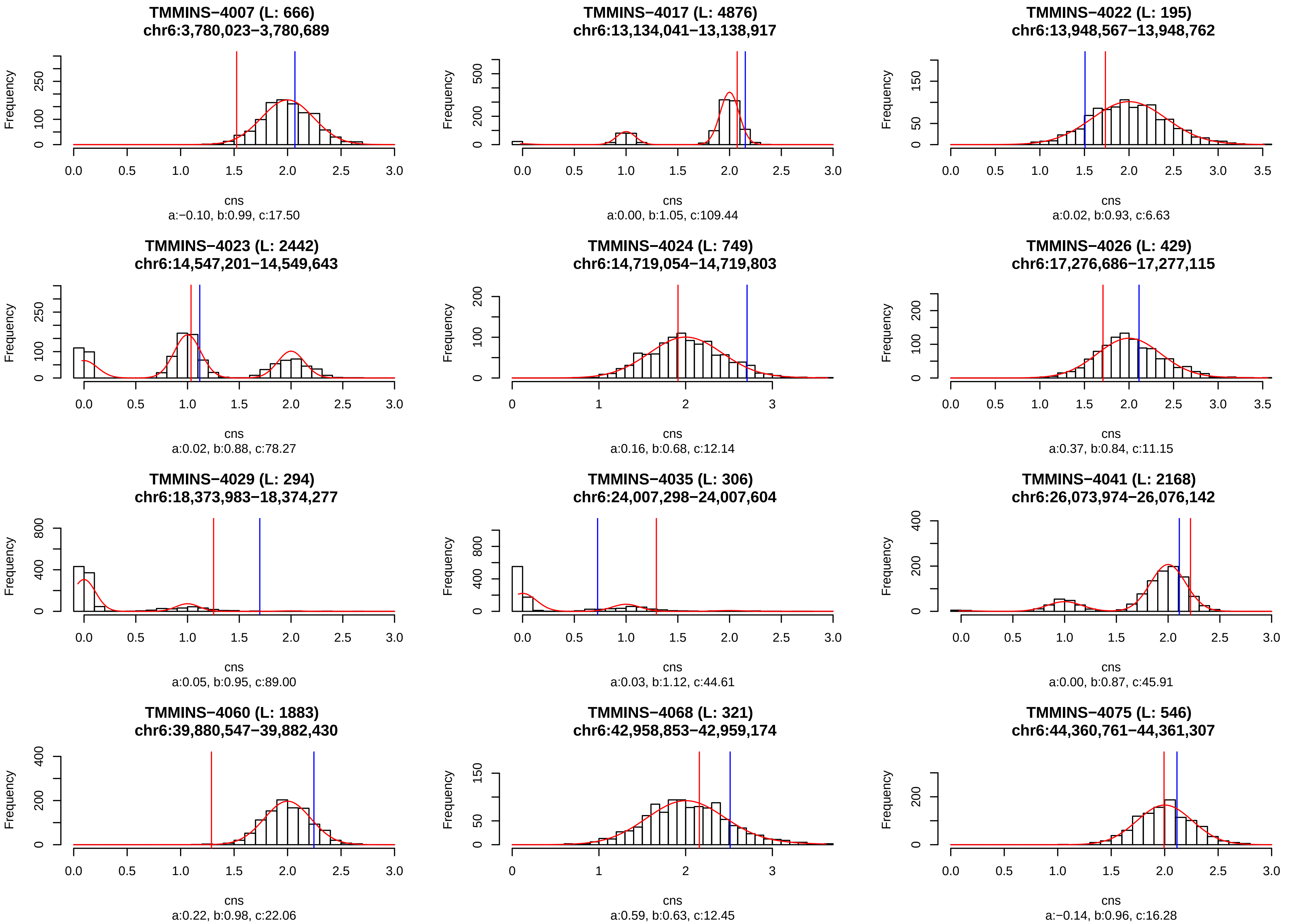

TMMINS−4007 (L: 666)
chr6:3,780,023−3,780,689
Frequency
cns
a:−0.10, b:0.99, c:17.50
TMMINS−4017 (L: 4876)
chr6:13,134,041−13,138,917
Frequency
cns
a:0.00, b:1.05, c:109.44
TMMINS−4022 (L: 195)
chr6:13,948,567−13,948,762
Frequency
cns
a:0.02, b:0.93, c:6.63
TMMINS−4023 (L: 2442)
chr6:14,547,201−14,549,643
Frequency
cns
a:0.02, b:0.88, c:78.27
TMMINS−4024 (L: 749)
chr6:14,719,054−14,719,803
Frequency
cns
a:0.16, b:0.68, c:12.14
TMMINS−4026 (L: 429)
chr6:17,276,686−17,277,115
Frequency
cns
a:0.37, b:0.84, c:11.15
TMMINS−4029 (L: 294)
chr6:18,373,983−18,374,277
Frequency
cns
a:0.05, b:0.95, c:89.00
TMMINS−4035 (L: 306)
chr6:24,007,298−24,007,604
Frequency
cns
a:0.03, b:1.12, c:44.61
TMMINS−4041 (L: 2168)
chr6:26,073,974−26,076,142
Frequency
cns
a:0.00, b:0.87, c:45.91
TMMINS−4060 (L: 1883)
chr6:39,880,547−39,882,430
Frequency
cns
a:0.22, b:0.98, c:22.06
TMMINS−4068 (L: 321)
chr6:42,958,853−42,959,174
Frequency
cns
a:0.59, b:0.63, c:12.45
TMMINS−4075 (L: 546)
chr6:44,360,761−44,361,307
Frequency
cns
a:−0.14, b:0.96, c:16.28

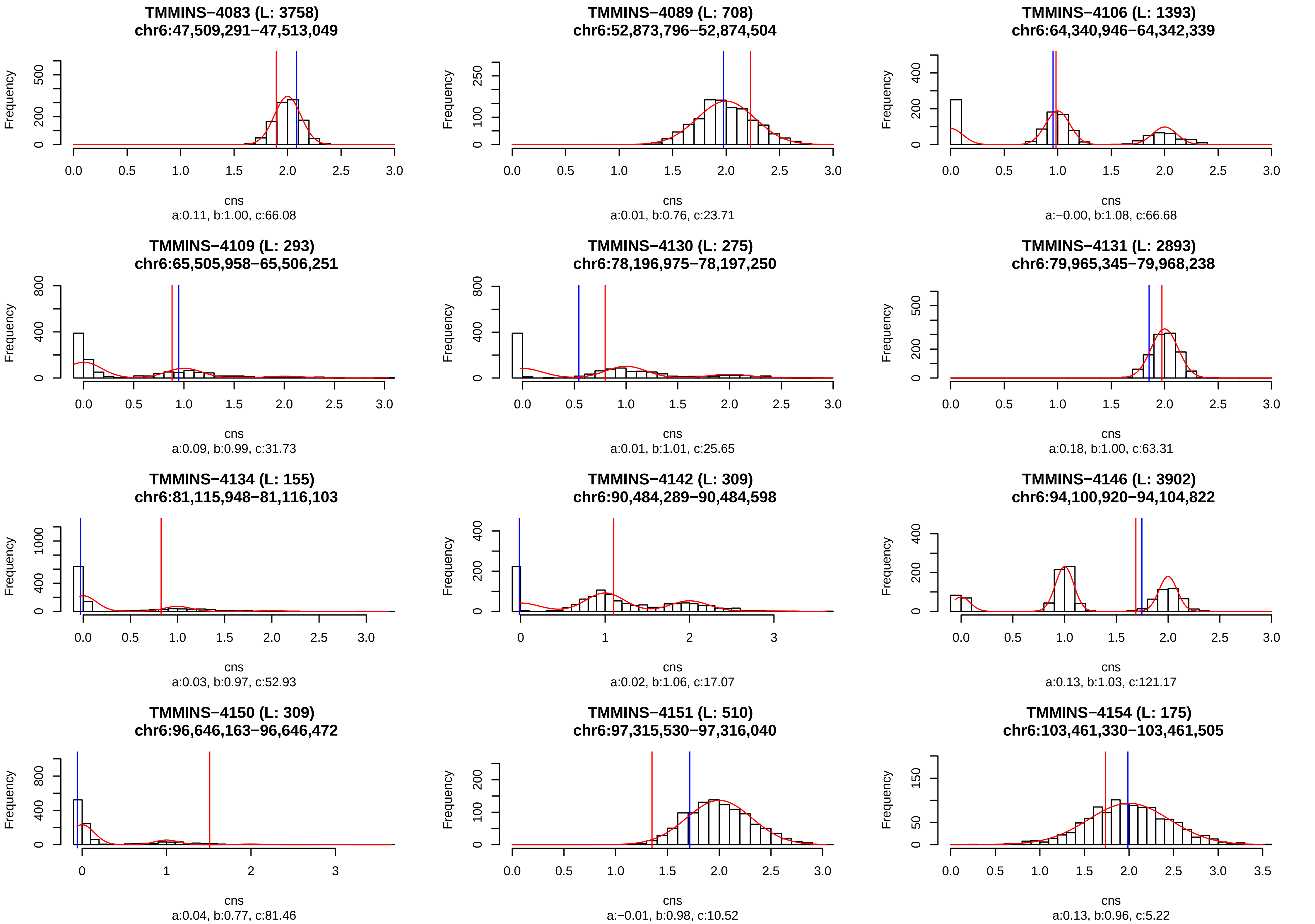
TMMINS−4083 (L: 3758)
chr6:47,509,291−47,513,049
Frequency
cns
a:0.11, b:1.00, c:66.08
TMMINS−4089 (L: 708)
chr6:52,873,796−52,874,504
Frequency
cns
a:0.01, b:0.76, c:23.71
TMMINS−4106 (L: 1393)
chr6:64,340,946−64,342,339
Frequency
cns
a:−0.00, b:1.08, c:66.68
TMMINS−4109 (L: 293)
chr6:65,505,958−65,506,251
Frequency
cns
a:0.09, b:0.99, c:31.73
TMMINS−4130 (L: 275)
chr6:78,196,975−78,197,250
Frequency
cns
a:0.01, b:1.01, c:25.65
TMMINS−4131 (L: 2893)
chr6:79,965,345−79,968,238
Frequency
cns
a:0.18, b:1.00, c:63.31
TMMINS−4134 (L: 155)
chr6:81,115,948−81,116,103
Frequency
cns
a:0.03, b:0.97, c:52.93
TMMINS−4142 (L: 309)
chr6:90,484,289−90,484,598
Frequency
cns
a:0.02, b:1.06, c:17.07
TMMINS−4146 (L: 3902)
chr6:94,100,920−94,104,822
Frequency
cns
a:0.13, b:1.03, c:121.17
TMMINS−4150 (L: 309)
chr6:96,646,163−96,646,472
Frequency
cns
a:0.04, b:0.77, c:81.46
TMMINS−4151 (L: 510)
chr6:97,315,530−97,316,040
Frequency
cns
a:−0.01, b:0.98, c:10.52
TMMINS−4154 (L: 175)
chr6:103,461,330−103,461,505
Frequency
cns
a:0.13, b:0.96, c:5.22

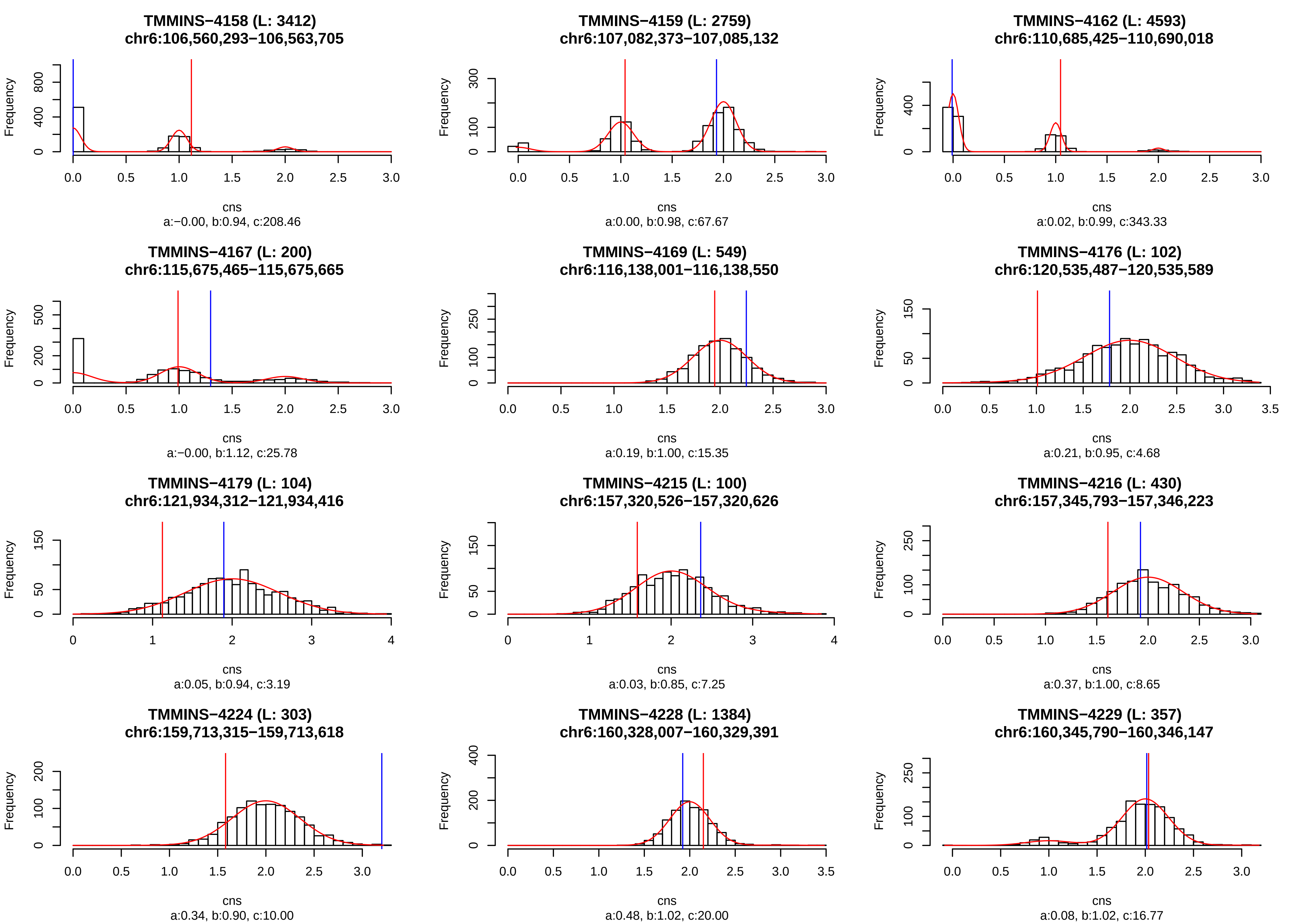
TMMINS−4158 (L: 3412)
chr6:106,560,293−106,563,705
a:−0.00, b:0.94, c:208.46
TMMINS−4159 (L: 2759)
chr6:107,082,373−107,085,132
a:0.00, b:0.98, c:67.67
TMMINS−4162 (L: 4593)
chr6:110,685,425−110,690,018
a:0.02, b:0.99, c:343.33
TMMINS−4167 (L: 200)
chr6:115,675,465−115,675,665
a:−0.00, b:1.12, c:25.78
TMMINS−4169 (L: 549)
chr6:116,138,001−116,138,550
a:0.19, b:1.00, c:15.35
TMMINS−4176 (L: 102)
chr6:120,535,487−120,535,589
a:0.21, b:0.95, c:4.68
TMMINS−4179 (L: 104)
chr6:121,934,312−121,934,416
a:0.05, b:0.94, c:3.19
TMMINS−4215 (L: 100)
chr6:157,320,526−157,320,626
a:0.03, b:0.85, c:7.25
TMMINS−4216 (L: 430)
chr6:157,345,793−157,346,223
a:0.37, b:1.00, c:8.65
TMMINS−4224 (L: 303)
chr6:159,713,315−159,713,618
a:0.34, b:0.90, c:10.00
TMMINS−4228 (L: 1384)
chr6:160,328,007−160,329,391
a:0.48, b:1.02, c:20.00
TMMINS−4229 (L: 357)
chr6:160,345,790−160,346,147
a:0.08, b:1.02, c:16.77
Frequency
cns

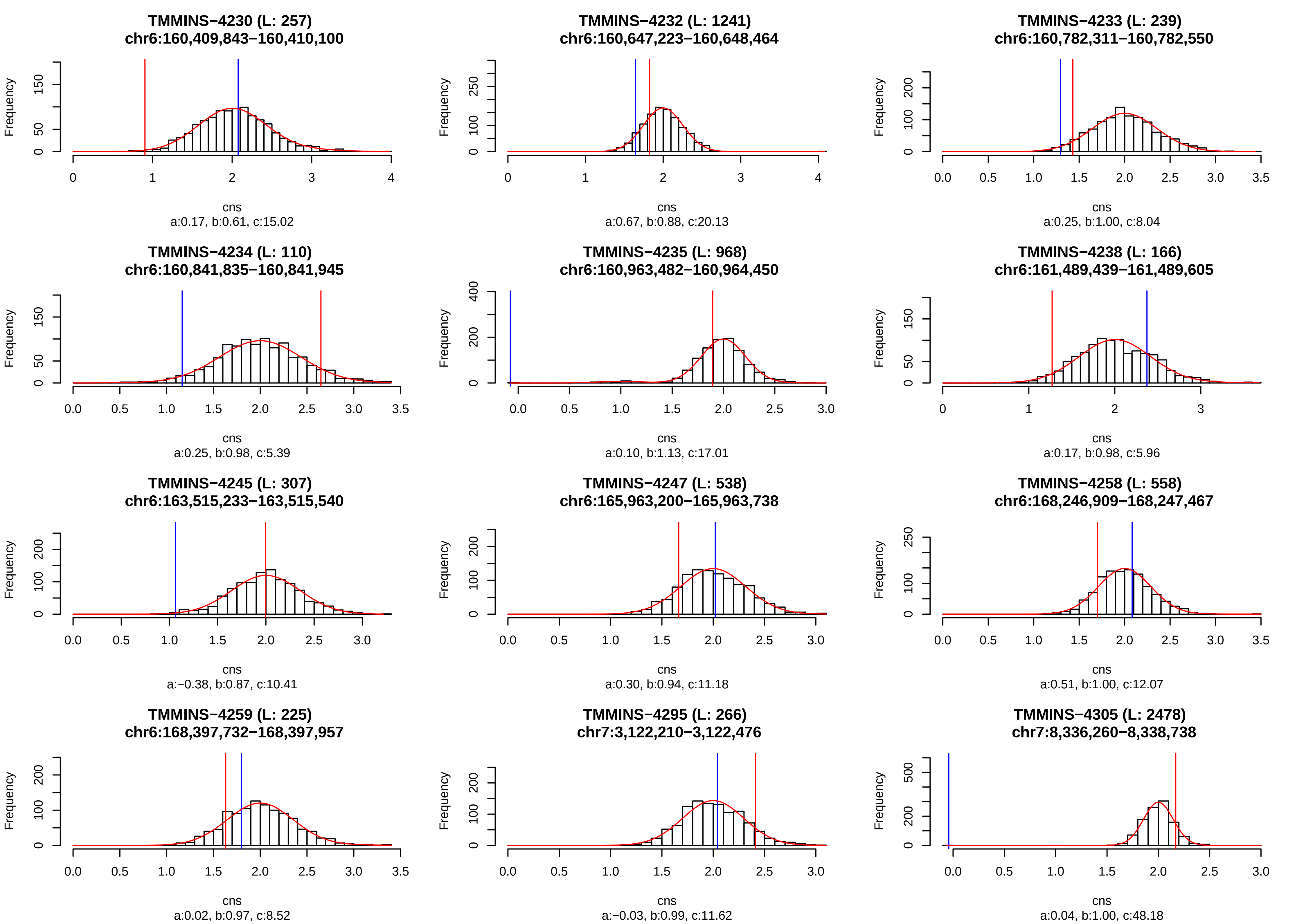
TMMINS−4230 (L: 257)
chr6:160,409,843−160,410,100
a:0.17, b:0.61, c:15.02
TMMINS−4232 (L: 1241)
chr6:160,647,223−160,648,464
a:0.67, b:0.88, c:20.13
TMMINS−4233 (L: 239)
chr6:160,782,311−160,782,550
a:0.25, b:1.00, c:8.04
TMMINS−4234 (L: 110)
chr6:160,841,835−160,841,945
a:0.25, b:0.98, c:5.39
TMMINS−4235 (L: 968)
chr6:160,963,482−160,964,450
a:0.10, b:1.13, c:17.01
TMMINS−4238 (L: 166)
chr6:161,489,439−161,489,605
a:0.17, b:0.98, c:5.96
TMMINS−4245 (L: 307)
chr6:163,515,233−163,515,540
a:−0.38, b:0.87, c:10.41
TMMINS−4247 (L: 538)
chr6:165,963,200−165,963,738
a:0.30, b:0.94, c:11.18
TMMINS−4258 (L: 558)
chr6:168,246,909−168,247,467
a:0.51, b:1.00, c:12.07
TMMINS−4259 (L: 225)
chr6:168,397,732−168,397,957
a:0.02, b:0.97, c:8.52
TMMINS−4295 (L: 266)
chr7:3,122,210−3,122,476
a:−0.03, b:0.99, c:11.62
TMMINS−4305 (L: 2478)
chr7:8,336,260−8,338,738
a:0.04, b:1.00, c:48.18
Frequency
cns

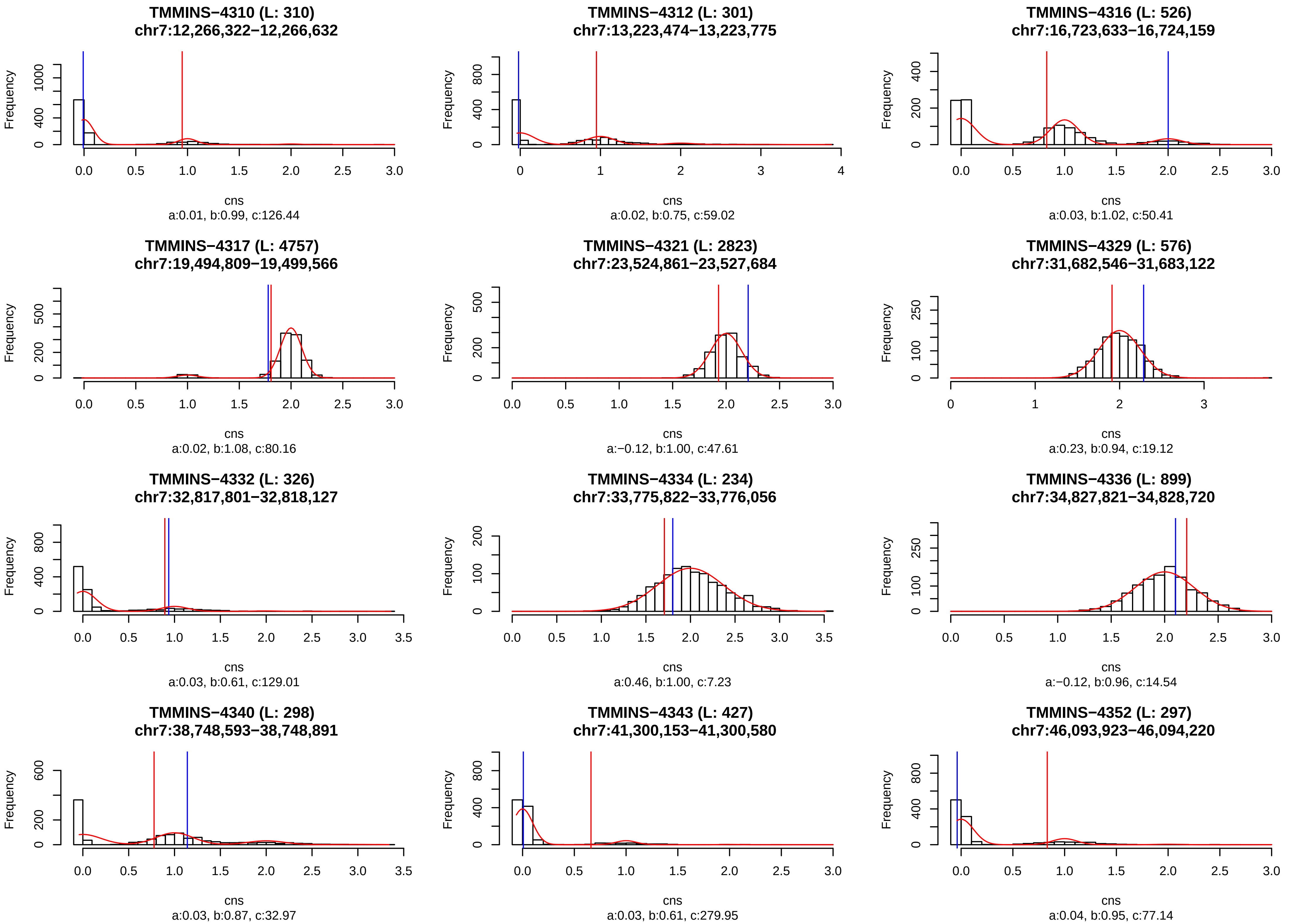
TMMINS−4310 (L: 310)
chr7:12,266,322−12,266,632
Frequency
cns
a:0.01, b:0.99, c:126.44
TMMINS−4312 (L: 301)
chr7:13,223,474−13,223,775
Frequency
cns
a:0.02, b:0.75, c:59.02
TMMINS−4316 (L: 526)
chr7:16,723,633−16,724,159
Frequency
cns
a:0.03, b:1.02, c:50.41
TMMINS−4317 (L: 4757)
chr7:19,494,809−19,499,566
Frequency
cns
a:0.02, b:1.08, c:80.16
TMMINS−4321 (L: 2823)
chr7:23,524,861−23,527,684
Frequency
cns
a:−0.12, b:1.00, c:47.61
TMMINS−4329 (L: 576)
chr7:31,682,546−31,683,122
Frequency
cns
a:0.23, b:0.94, c:19.12
TMMINS−4332 (L: 326)
chr7:32,817,801−32,818,127
Frequency
cns
a:0.03, b:0.61, c:129.01
TMMINS−4334 (L: 234)
chr7:33,775,822−33,776,056
Frequency
cns
a:0.46, b:1.00, c:7.23
TMMINS−4336 (L: 899)
chr7:34,827,821−34,828,720
Frequency
cns
a:−0.12, b:0.96, c:14.54
TMMINS−4340 (L: 298)
chr7:38,748,593−38,748,891
Frequency
cns
a:0.03, b:0.87, c:32.97
TMMINS−4343 (L: 427)
chr7:41,300,153−41,300,580
Frequency
cns
a:0.03, b:0.61, c:279.95
TMMINS−4352 (L: 297)
chr7:46,093,923−46,094,220
Frequency
cns
a:0.04, b:0.95, c:77.14

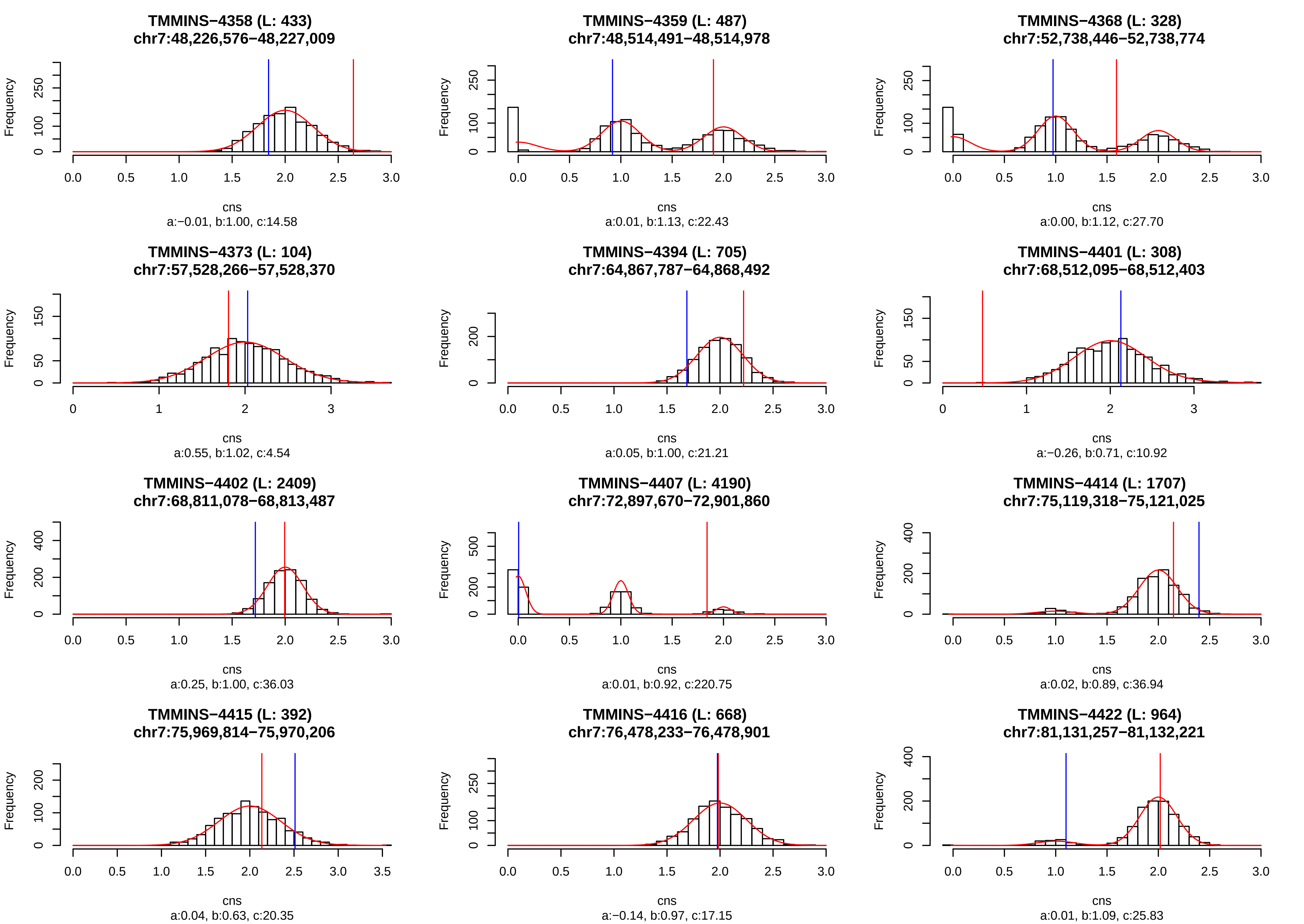
TMMINS−4358 (L: 433)
chr7:48,226,576−48,227,009
a:−0.01, b:1.00, c:14.58
TMMINS−4359 (L: 487)
chr7:48,514,491−48,514,978
a:0.01, b:1.13, c:22.43
TMMINS−4368 (L: 328)
chr7:52,738,446−52,738,774
a:0.00, b:1.12, c:27.70
TMMINS−4373 (L: 104)
chr7:57,528,266−57,528,370
a:0.55, b:1.02, c:4.54
TMMINS−4394 (L: 705)
chr7:64,867,787−64,868,492
a:0.05, b:1.00, c:21.21
TMMINS−4401 (L: 308)
chr7:68,512,095−68,512,403
a:−0.26, b:0.71, c:10.92
TMMINS−4402 (L: 2409)
chr7:68,811,078−68,813,487
a:0.25, b:1.00, c:36.03
TMMINS−4407 (L: 4190)
chr7:72,897,670−72,901,860
a:0.01, b:0.92, c:220.75
TMMINS−4414 (L: 1707)
chr7:75,119,318−75,121,025
a:0.02, b:0.89, c:36.94
TMMINS−4415 (L: 392)
chr7:75,969,814−75,970,206
a:0.04, b:0.63, c:20.35
TMMINS−4416 (L: 668)
chr7:76,478,233−76,478,901
a:−0.14, b:0.97, c:17.15
TMMINS−4422 (L: 964)
chr7:81,131,257−81,132,221
a:0.01, b:1.09, c:25.83
Frequency
cns

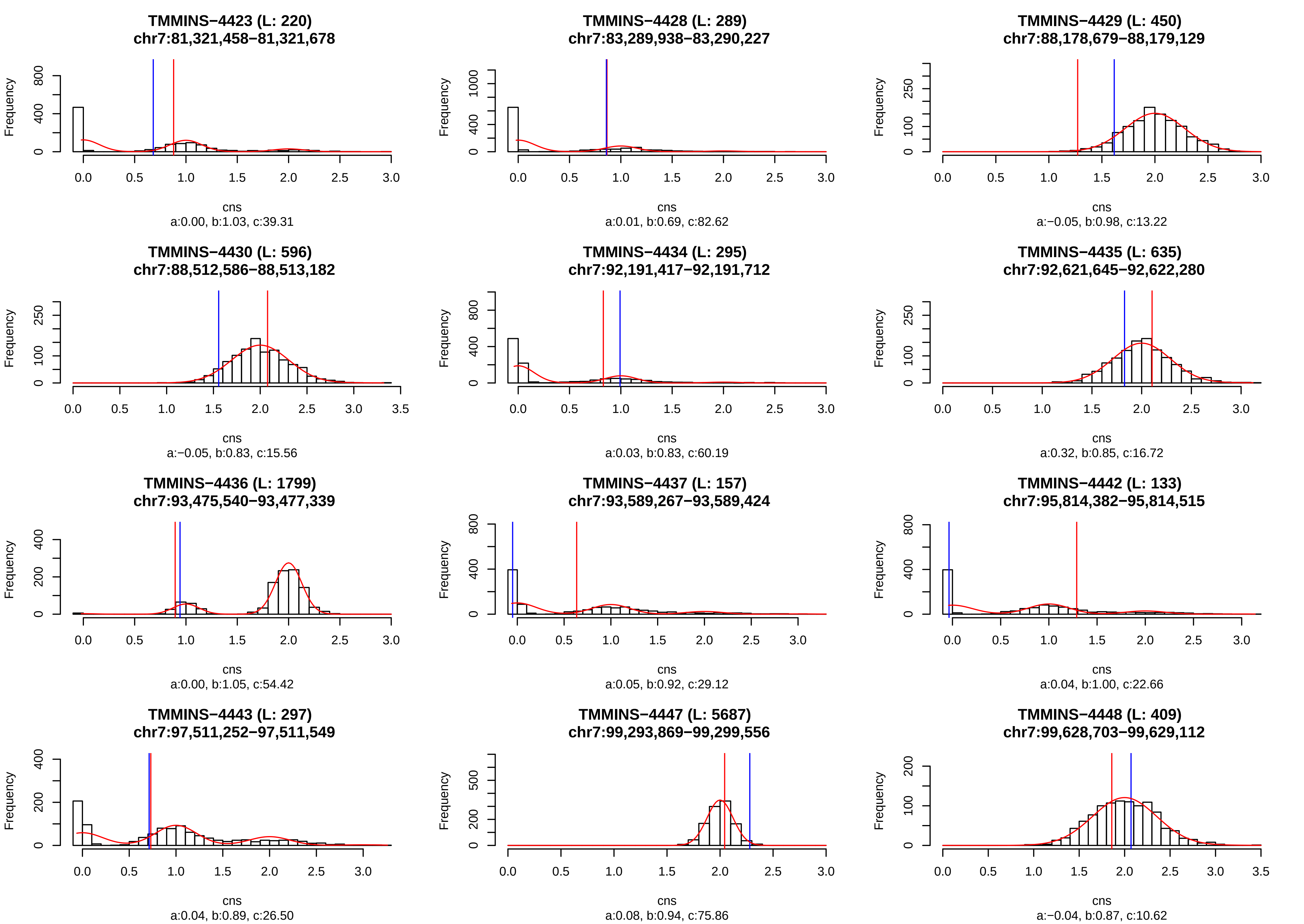
TMMINS−4423 (L: 220)
chr7:81,321,458−81,321,678
Frequency
cns
a:0.00, b:1.03, c:39.31
TMMINS−4428 (L: 289)
chr7:83,289,938−83,290,227
Frequency
cns
a:0.01, b:0.69, c:82.62
TMMINS−4429 (L: 450)
chr7:88,178,679−88,179,129
Frequency
cns
a:−0.05, b:0.98, c:13.22
TMMINS−4430 (L: 596)
chr7:88,512,586−88,513,182
Frequency
cns
a:−0.05, b:0.83, c:15.56
TMMINS−4434 (L: 295)
chr7:92,191,417−92,191,712
Frequency
cns
a:0.03, b:0.83, c:60.19
TMMINS−4435 (L: 635)
chr7:92,621,645−92,622,280
Frequency
cns
a:0.32, b:0.85, c:16.72
TMMINS−4436 (L: 1799)
chr7:93,475,540−93,477,339
Frequency
cns
a:0.00, b:1.05, c:54.42
TMMINS−4437 (L: 157)
chr7:93,589,267−93,589,424
Frequency
cns
a:0.05, b:0.92, c:29.12
TMMINS−4442 (L: 133)
chr7:95,814,382−95,814,515
Frequency
cns
a:0.04, b:1.00, c:22.66
TMMINS−4443 (L: 297)
chr7:97,511,252−97,511,549
Frequency
cns
a:0.04, b:0.89, c:26.50
TMMINS−4447 (L: 5687)
chr7:99,293,869−99,299,556
Frequency
cns
a:0.08, b:0.94, c:75.86
TMMINS−4448 (L: 409)
chr7:99,628,703−99,629,112
Frequency
cns
a:−0.04, b:0.87, c:10.62

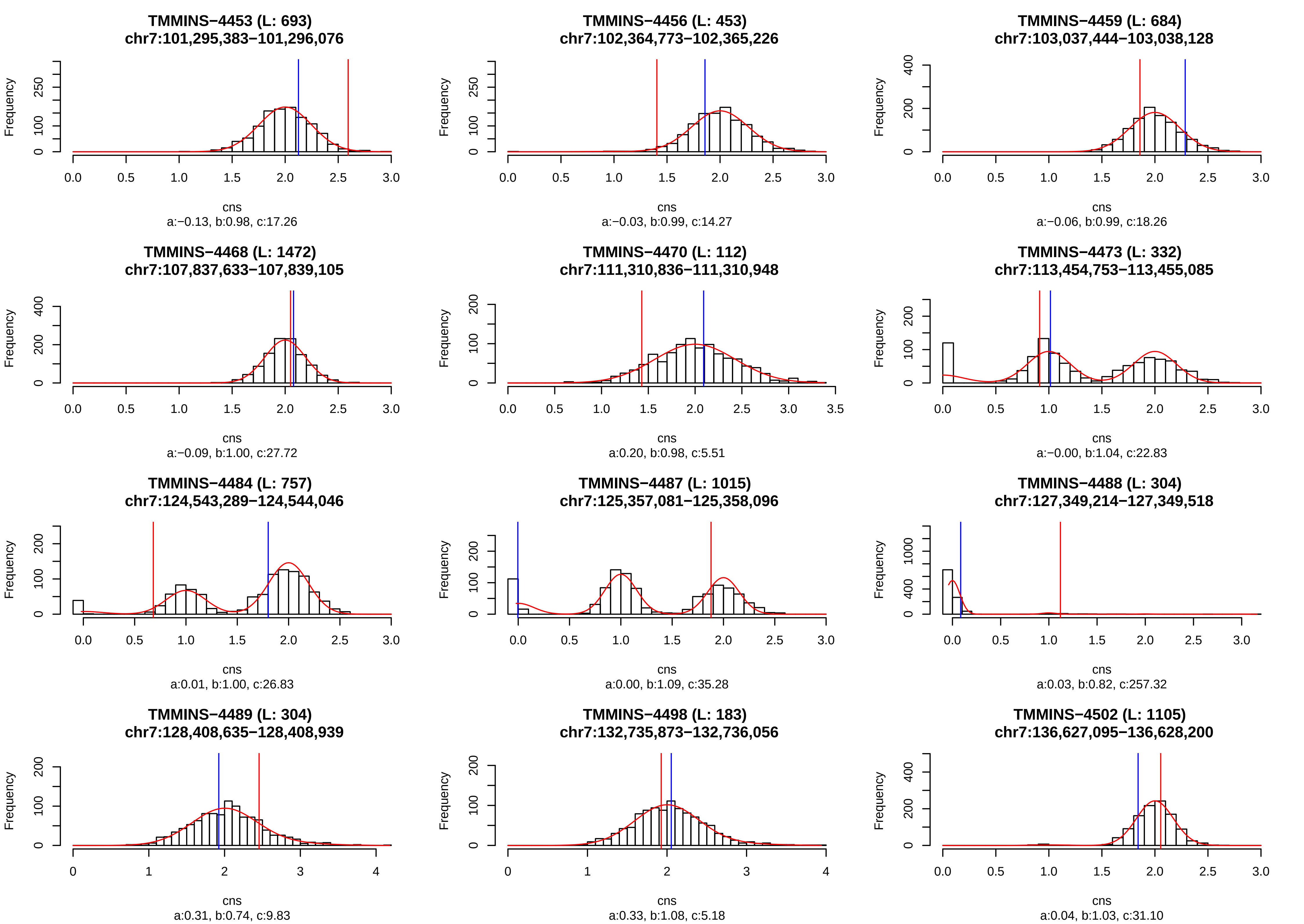
TMMINS−4453 (L: 693)
chr7:101,295,383−101,296,076
Frequency
cns
a:−0.13, b:0.98, c:17.26
TMMINS−4456 (L: 453)
chr7:102,364,773−102,365,226
Frequency
cns
a:−0.03, b:0.99, c:14.27
TMMINS−4459 (L: 684)
chr7:103,037,444−103,038,128
Frequency
cns
a:−0.06, b:0.99, c:18.26
TMMINS−4468 (L: 1472)
chr7:107,837,633−107,839,105
Frequency
cns
a:−0.09, b:1.00, c:27.72
TMMINS−4470 (L: 112)
chr7:111,310,836−111,310,948
Frequency
cns
a:0.20, b:0.98, c:5.51
TMMINS−4473 (L: 332)
chr7:113,454,753−113,455,085
Frequency
cns
a:−0.00, b:1.04, c:22.83
TMMINS−4484 (L: 757)
chr7:124,543,289−124,544,046
Frequency
cns
a:0.01, b:1.00, c:26.83
TMMINS−4487 (L: 1015)
chr7:125,357,081−125,358,096
Frequency
cns
a:0.00, b:1.09, c:35.28
TMMINS−4488 (L: 304)
chr7:127,349,214−127,349,518
Frequency
cns
a:0.03, b:0.82, c:257.32
TMMINS−4489 (L: 304)
chr7:128,408,635−128,408,939
Frequency
cns
a:0.31, b:0.74, c:9.83
TMMINS−4498 (L: 183)
chr7:132,735,873−132,736,056
Frequency
cns
a:0.33, b:1.08, c:5.18
TMMINS−4502 (L: 1105)
chr7:136,627,095−136,628,200
Frequency
cns
a:0.04, b:1.03, c:31.10

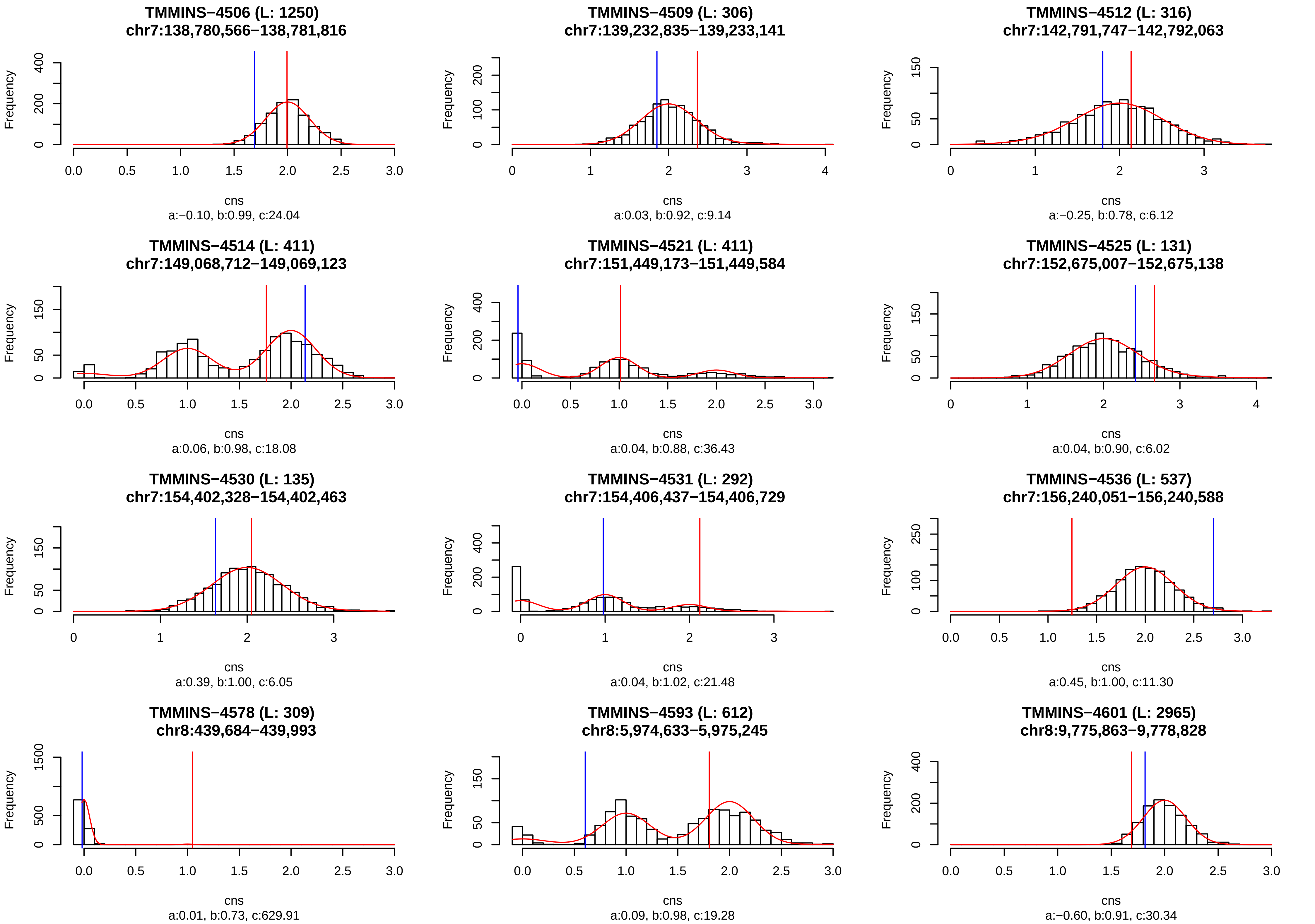
TMMINS−4506 (L: 1250)
chr7:138,780,566−138,781,816
Frequency
cns
a:−0.10, b:0.99, c:24.04
TMMINS−4509 (L: 306)
chr7:139,232,835−139,233,141
Frequency
cns
a:0.03, b:0.92, c:9.14
TMMINS−4512 (L: 316)
chr7:142,791,747−142,792,063
Frequency
cns
a:−0.25, b:0.78, c:6.12
TMMINS−4514 (L: 411)
chr7:149,068,712−149,069,123
Frequency
cns
a:0.06, b:0.98, c:18.08
TMMINS−4521 (L: 411)
chr7:151,449,173−151,449,584
Frequency
cns
a:0.04, b:0.88, c:36.43
TMMINS−4525 (L: 131)
chr7:152,675,007−152,675,138
Frequency
cns
a:0.04, b:0.90, c:6.02
TMMINS−4530 (L: 135)
chr7:154,402,328−154,402,463
Frequency
cns
a:0.39, b:1.00, c:6.05
TMMINS−4531 (L: 292)
chr7:154,406,437−154,406,729
Frequency
cns
a:0.04, b:1.02, c:21.48
TMMINS−4536 (L: 537)
chr7:156,240,051−156,240,588
Frequency
cns
a:0.45, b:1.00, c:11.30
TMMINS−4578 (L: 309)
chr8:439,684−439,993
Frequency
cns
a:0.01, b:0.73, c:629.91
TMMINS−4593 (L: 612)
chr8:5,974,633−5,975,245
Frequency
cns
a:0.09, b:0.98, c:19.28
TMMINS−4601 (L: 2965)
chr8:9,775,863−9,778,828
Frequency
cns
a:−0.60, b:0.91, c:30.34

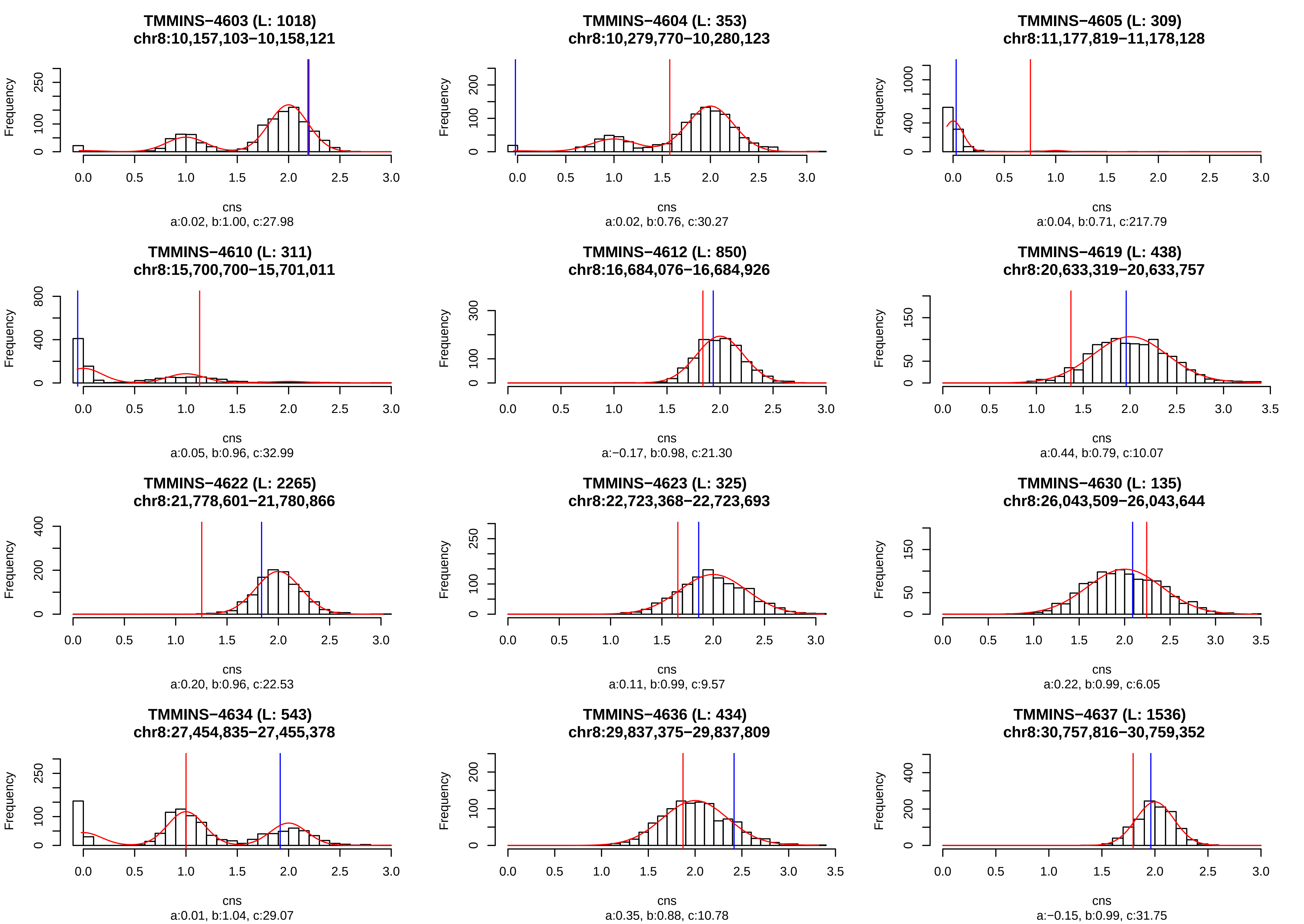
TMMINS−4603 (L: 1018)
chr8:10,157,103−10,158,121
Frequency
cns
a:0.02, b:1.00, c:27.98
TMMINS−4604 (L: 353)
chr8:10,279,770−10,280,123
Frequency
cns
a:0.02, b:0.76, c:30.27
TMMINS−4605 (L: 309)
chr8:11,177,819−11,178,128
Frequency
cns
a:0.04, b:0.71, c:217.79
TMMINS−4610 (L: 311)
chr8:15,700,700−15,701,011
Frequency
cns
a:0.05, b:0.96, c:32.99
TMMINS−4612 (L: 850)
chr8:16,684,076−16,684,926
Frequency
cns
a:−0.17, b:0.98, c:21.30
TMMINS−4619 (L: 438)
chr8:20,633,319−20,633,757
Frequency
cns
a:0.44, b:0.79, c:10.07
TMMINS−4622 (L: 2265)
chr8:21,778,601−21,780,866
Frequency
cns
a:0.20, b:0.96, c:22.53
TMMINS−4623 (L: 325)
chr8:22,723,368−22,723,693
Frequency
cns
a:0.11, b:0.99, c:9.57
TMMINS−4630 (L: 135)
chr8:26,043,509−26,043,644
Frequency
cns
a:0.22, b:0.99, c:6.05
TMMINS−4634 (L: 543)
chr8:27,454,835−27,455,378
Frequency
cns
a:0.01, b:1.04, c:29.07
TMMINS−4636 (L: 434)
chr8:29,837,375−29,837,809
Frequency
cns
a:0.35, b:0.88, c:10.78
TMMINS−4637 (L: 1536)
chr8:30,757,816−30,759,352
Frequency
cns
a:−0.15, b:0.99, c:31.75

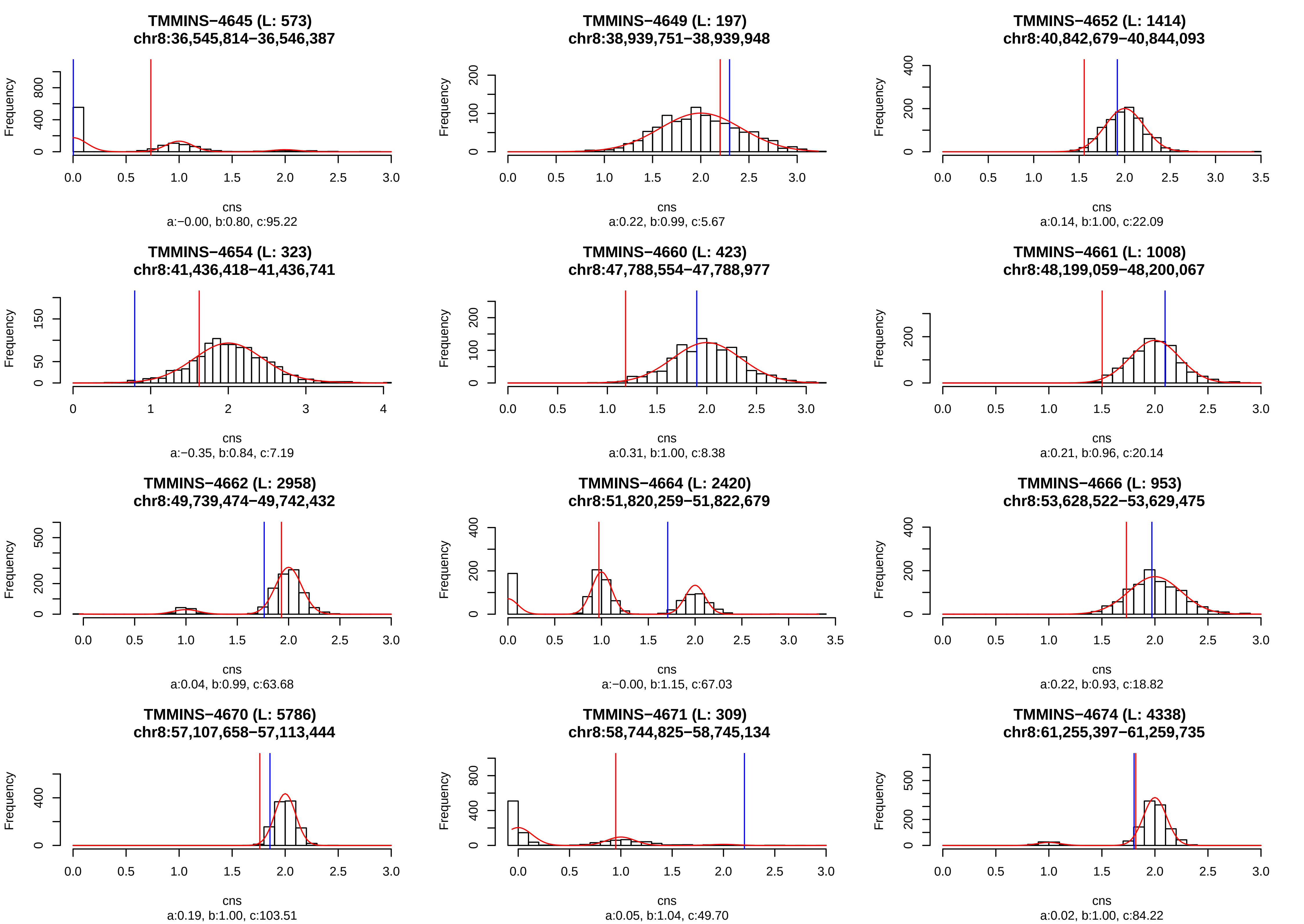
TMMINS−4645 (L: 573)
chr8:36,545,814−36,546,387
Frequency
cns
a:−0.00, b:0.80, c:95.22
TMMINS−4649 (L: 197)
chr8:38,939,751−38,939,948
Frequency
cns
a:0.22, b:0.99, c:5.67
TMMINS−4652 (L: 1414)
chr8:40,842,679−40,844,093
Frequency
cns
a:0.14, b:1.00, c:22.09
TMMINS−4654 (L: 323)
chr8:41,436,418−41,436,741
Frequency
cns
a:−0.35, b:0.84, c:7.19
TMMINS−4660 (L: 423)
chr8:47,788,554−47,788,977
Frequency
cns
a:0.31, b:1.00, c:8.38
TMMINS−4661 (L: 1008)
chr8:48,199,059−48,200,067
Frequency
cns
a:0.21, b:0.96, c:20.14
TMMINS−4662 (L: 2958)
chr8:49,739,474−49,742,432
Frequency
cns
a:0.04, b:0.99, c:63.68
TMMINS−4664 (L: 2420)
chr8:51,820,259−51,822,679
Frequency
cns
a:−0.00, b:1.15, c:67.03
TMMINS−4666 (L: 953)
chr8:53,628,522−53,629,475
Frequency
cns
a:0.22, b:0.93, c:18.82
TMMINS−4670 (L: 5786)
chr8:57,107,658−57,113,444
Frequency
cns
a:0.19, b:1.00, c:103.51
TMMINS−4671 (L: 309)
chr8:58,744,825−58,745,134
Frequency
cns
a:0.05, b:1.04, c:49.70
TMMINS−4674 (L: 4338)
chr8:61,255,397−61,259,735
Frequency
cns
a:0.02, b:1.00, c:84.22

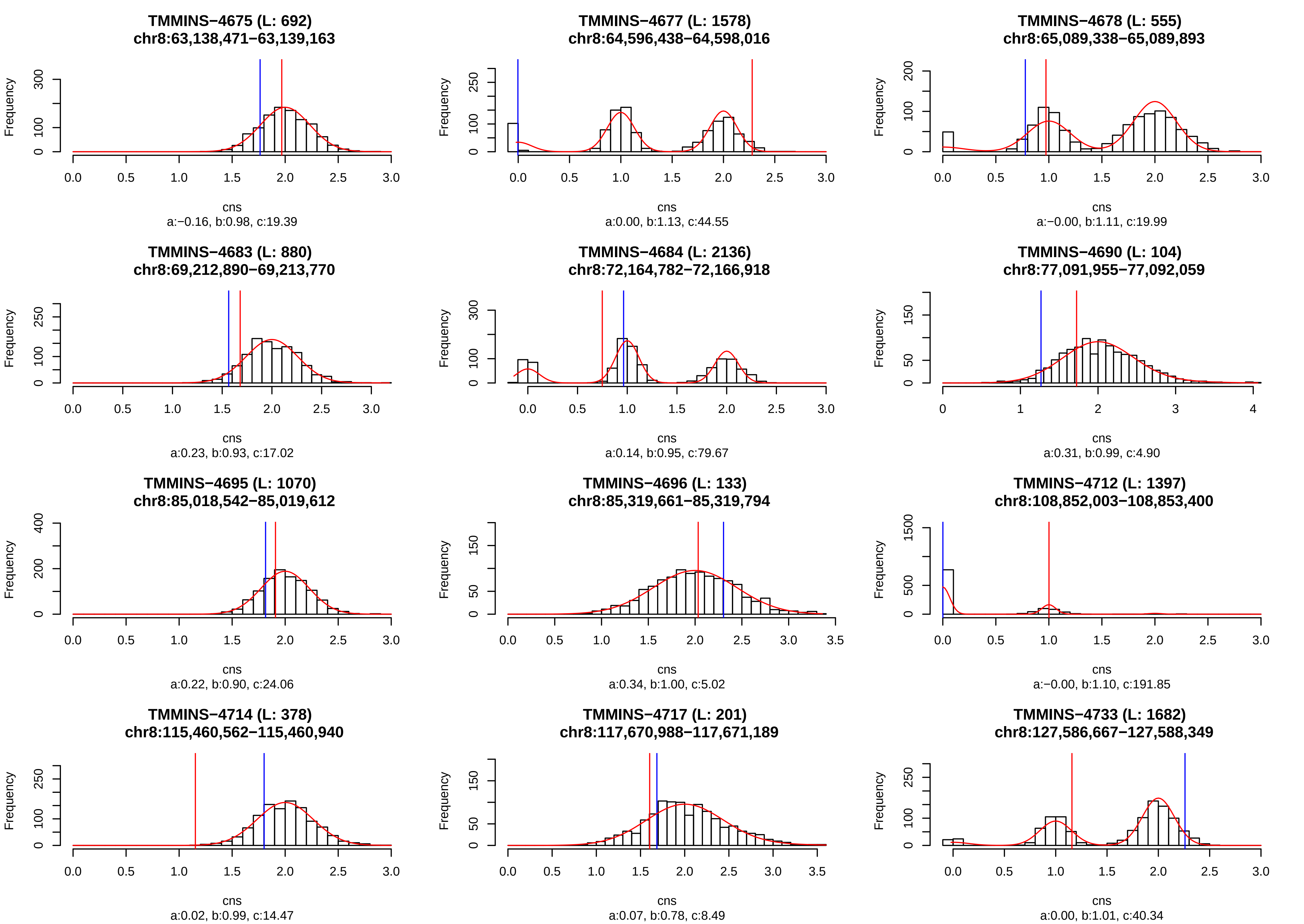
TMMINS−4675 (L: 692)
chr8:63,138,471−63,139,163
a:−0.16, b:0.98, c:19.39
TMMINS−4677 (L: 1578)
chr8:64,596,438−64,598,016
a:0.00, b:1.13, c:44.55
TMMINS−4678 (L: 555)
chr8:65,089,338−65,089,893
a:−0.00, b:1.11, c:19.99
TMMINS−4683 (L: 880)
chr8:69,212,890−69,213,770
a:0.23, b:0.93, c:17.02
TMMINS−4684 (L: 2136)
chr8:72,164,782−72,166,918
a:0.14, b:0.95, c:79.67
TMMINS−4690 (L: 104)
chr8:77,091,955−77,092,059
a:0.31, b:0.99, c:4.90
TMMINS−4695 (L: 1070)
chr8:85,018,542−85,019,612
a:0.22, b:0.90, c:24.06
TMMINS−4696 (L: 133)
chr8:85,319,661−85,319,794
a:0.34, b:1.00, c:5.02
TMMINS−4712 (L: 1397)
chr8:108,852,003−108,853,400
a:−0.00, b:1.10, c:191.85
TMMINS−4714 (L: 378)
chr8:115,460,562−115,460,940
a:0.02, b:0.99, c:14.47
TMMINS−4717 (L: 201)
chr8:117,670,988−117,671,189
a:0.07, b:0.78, c:8.49
TMMINS−4733 (L: 1682)
chr8:127,586,667−127,588,349
a:0.00, b:1.01, c:40.34
Frequency
cns

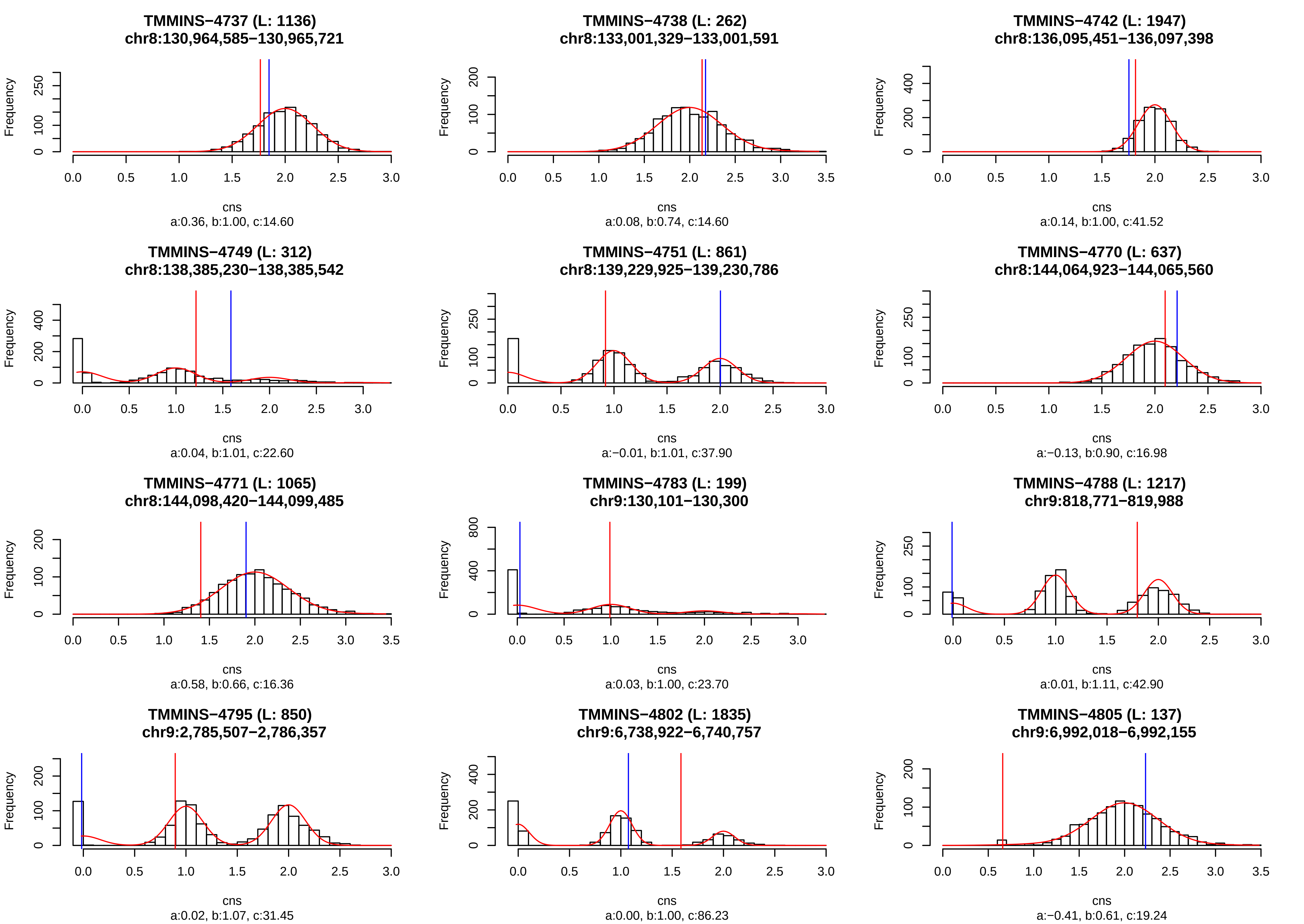
TMMINS−4737 (L: 1136)
chr8:130,964,585−130,965,721
Frequency
cns
a:0.36, b:1.00, c:14.60
TMMINS−4738 (L: 262)
chr8:133,001,329−133,001,591
Frequency
cns
a:0.08, b:0.74, c:14.60
TMMINS−4742 (L: 1947)
chr8:136,095,451−136,097,398
Frequency
cns
a:0.14, b:1.00, c:41.52
TMMINS−4749 (L: 312)
chr8:138,385,230−138,385,542
Frequency
cns
a:0.04, b:1.01, c:22.60
TMMINS−4751 (L: 861)
chr8:139,229,925−139,230,786
Frequency
cns
a:−0.01, b:1.01, c:37.90
TMMINS−4770 (L: 637)
chr8:144,064,923−144,065,560
Frequency
cns
a:−0.13, b:0.90, c:16.98
TMMINS−4771 (L: 1065)
chr8:144,098,420−144,099,485
Frequency
cns
a:0.58, b:0.66, c:16.36
TMMINS−4783 (L: 199)
chr9:130,101−130,300
Frequency
cns
a:0.03, b:1.00, c:23.70
TMMINS−4788 (L: 1217)
chr9:818,771−819,988
Frequency
cns
a:0.01, b:1.11, c:42.90
TMMINS−4795 (L: 850)
chr9:2,785,507−2,786,357
Frequency
cns
a:0.02, b:1.07, c:31.45
TMMINS−4802 (L: 1835)
chr9:6,738,922−6,740,757
Frequency
cns
a:0.00, b:1.00, c:86.23
TMMINS−4805 (L: 137)
chr9:6,992,018−6,992,155
Frequency
cns
a:−0.41, b:0.61, c:19.24

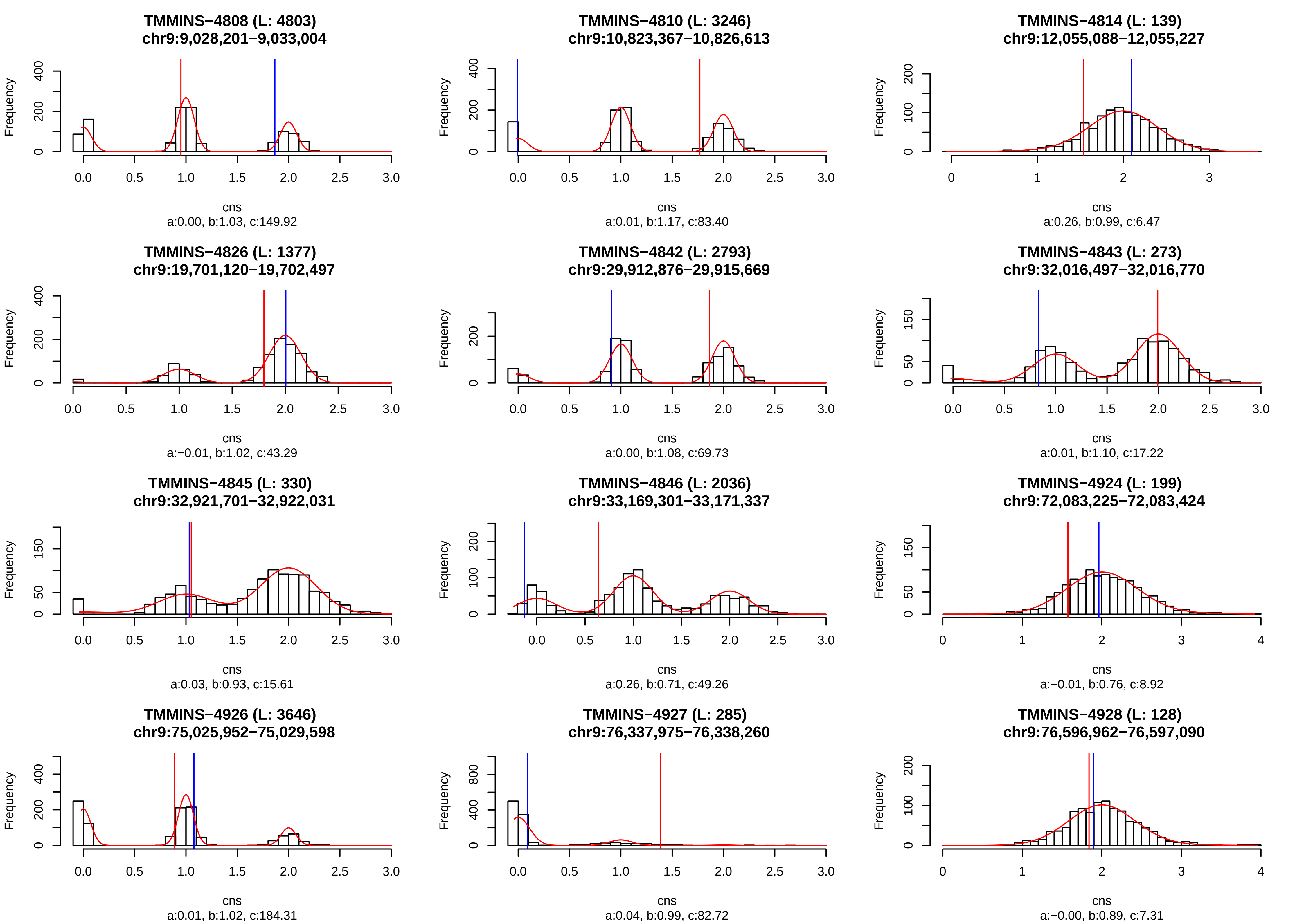
TMMINS−4808 (L: 4803)
chr9:9,028,201−9,033,004
Frequency
cns
a:0.00, b:1.03, c:149.92
TMMINS−4810 (L: 3246)
chr9:10,823,367−10,826,613
Frequency
cns
a:0.01, b:1.17, c:83.40
TMMINS−4814 (L: 139)
chr9:12,055,088−12,055,227
Frequency
cns
a:0.26, b:0.99, c:6.47
TMMINS−4826 (L: 1377)
chr9:19,701,120−19,702,497
Frequency
cns
a:−0.01, b:1.02, c:43.29
TMMINS−4842 (L: 2793)
chr9:29,912,876−29,915,669
Frequency
cns
a:0.00, b:1.08, c:69.73
TMMINS−4843 (L: 273)
chr9:32,016,497−32,016,770
Frequency
cns
a:0.01, b:1.10, c:17.22
TMMINS−4845 (L: 330)
chr9:32,921,701−32,922,031
Frequency
cns
a:0.03, b:0.93, c:15.61
TMMINS−4846 (L: 2036)
chr9:33,169,301−33,171,337
Frequency
cns
a:0.26, b:0.71, c:49.26
TMMINS−4924 (L: 199)
chr9:72,083,225−72,083,424
Frequency
cns
a:−0.01, b:0.76, c:8.92
TMMINS−4926 (L: 3646)
chr9:75,025,952−75,029,598
Frequency
cns
a:0.01, b:1.02, c:184.31
TMMINS−4927 (L: 285)
chr9:76,337,975−76,338,260
Frequency
cns
a:0.04, b:0.99, c:82.72
TMMINS−4928 (L: 128)
chr9:76,596,962−76,597,090
Frequency
cns
a:−0.00, b:0.89, c:7.31

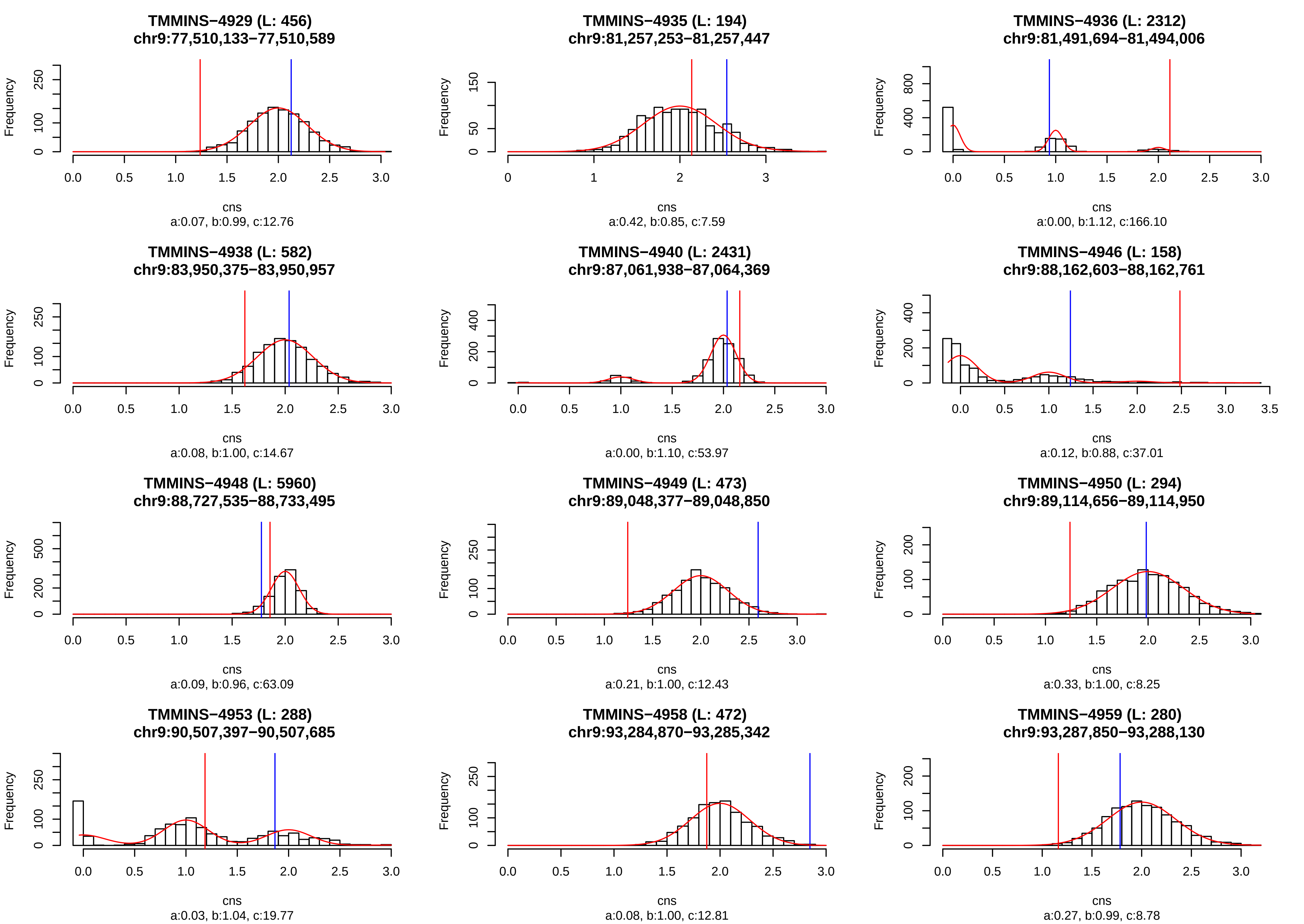
TMMINS−4929 (L: 456)
chr9:77,510,133−77,510,589
Frequency
cns
a:0.07, b:0.99, c:12.76
TMMINS−4935 (L: 194)
chr9:81,257,253−81,257,447
Frequency
cns
a:0.42, b:0.85, c:7.59
TMMINS−4936 (L: 2312)
chr9:81,491,694−81,494,006
Frequency
cns
a:0.00, b:1.12, c:166.10
TMMINS−4938 (L: 582)
chr9:83,950,375−83,950,957
Frequency
cns
a:0.08, b:1.00, c:14.67
TMMINS−4940 (L: 2431)
chr9:87,061,938−87,064,369
Frequency
cns
a:0.00, b:1.10, c:53.97
TMMINS−4946 (L: 158)
chr9:88,162,603−88,162,761
Frequency
cns
a:0.12, b:0.88, c:37.01
TMMINS−4948 (L: 5960)
chr9:88,727,535−88,733,495
Frequency
cns
a:0.09, b:0.96, c:63.09
TMMINS−4949 (L: 473)
chr9:89,048,377−89,048,850
Frequency
cns
a:0.21, b:1.00, c:12.43
TMMINS−4950 (L: 294)
chr9:89,114,656−89,114,950
Frequency
cns
a:0.33, b:1.00, c:8.25
TMMINS−4953 (L: 288)
chr9:90,507,397−90,507,685
Frequency
cns
a:0.03, b:1.04, c:19.77
TMMINS−4958 (L: 472)
chr9:93,284,870−93,285,342
Frequency
cns
a:0.08, b:1.00, c:12.81
TMMINS−4959 (L: 280)
chr9:93,287,850−93,288,130
Frequency
cns
a:0.27, b:0.99, c:8.78

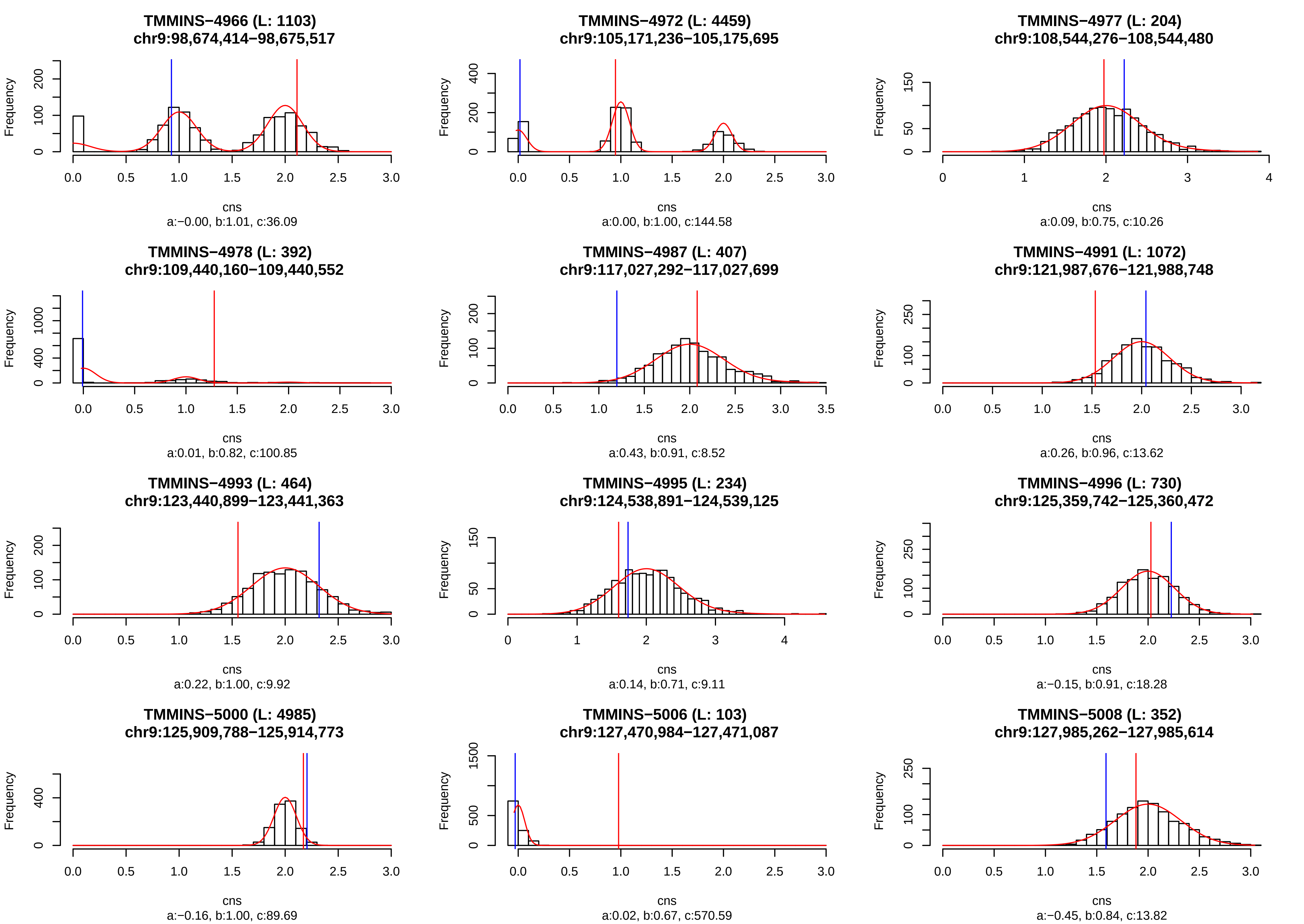
TMMINS−4966 (L: 1103)
chr9:98,674,414−98,675,517
Frequency
cns
a:−0.00, b:1.01, c:36.09
TMMINS−4972 (L: 4459)
chr9:105,171,236−105,175,695
Frequency
cns
a:0.00, b:1.00, c:144.58
TMMINS−4977 (L: 204)
chr9:108,544,276−108,544,480
Frequency
cns
a:0.09, b:0.75, c:10.26
TMMINS−4978 (L: 392)
chr9:109,440,160−109,440,552
Frequency
cns
a:0.01, b:0.82, c:100.85
TMMINS−4987 (L: 407)
chr9:117,027,292−117,027,699
Frequency
cns
a:0.43, b:0.91, c:8.52
TMMINS−4991 (L: 1072)
chr9:121,987,676−121,988,748
Frequency
cns
a:0.26, b:0.96, c:13.62
TMMINS−4993 (L: 464)
chr9:123,440,899−123,441,363
Frequency
cns
a:0.22, b:1.00, c:9.92
TMMINS−4995 (L: 234)
chr9:124,538,891−124,539,125
Frequency
cns
a:0.14, b:0.71, c:9.11
TMMINS−4996 (L: 730)
chr9:125,359,742−125,360,472
Frequency
cns
a:−0.15, b:0.91, c:18.28
TMMINS−5000 (L: 4985)
chr9:125,909,788−125,914,773
Frequency
cns
a:−0.16, b:1.00, c:89.69
TMMINS−5006 (L: 103)
chr9:127,470,984−127,471,087
Frequency
cns
a:0.02, b:0.67, c:570.59
TMMINS−5008 (L: 352)
chr9:127,985,262−127,985,614
Frequency
cns
a:−0.45, b:0.84, c:13.82

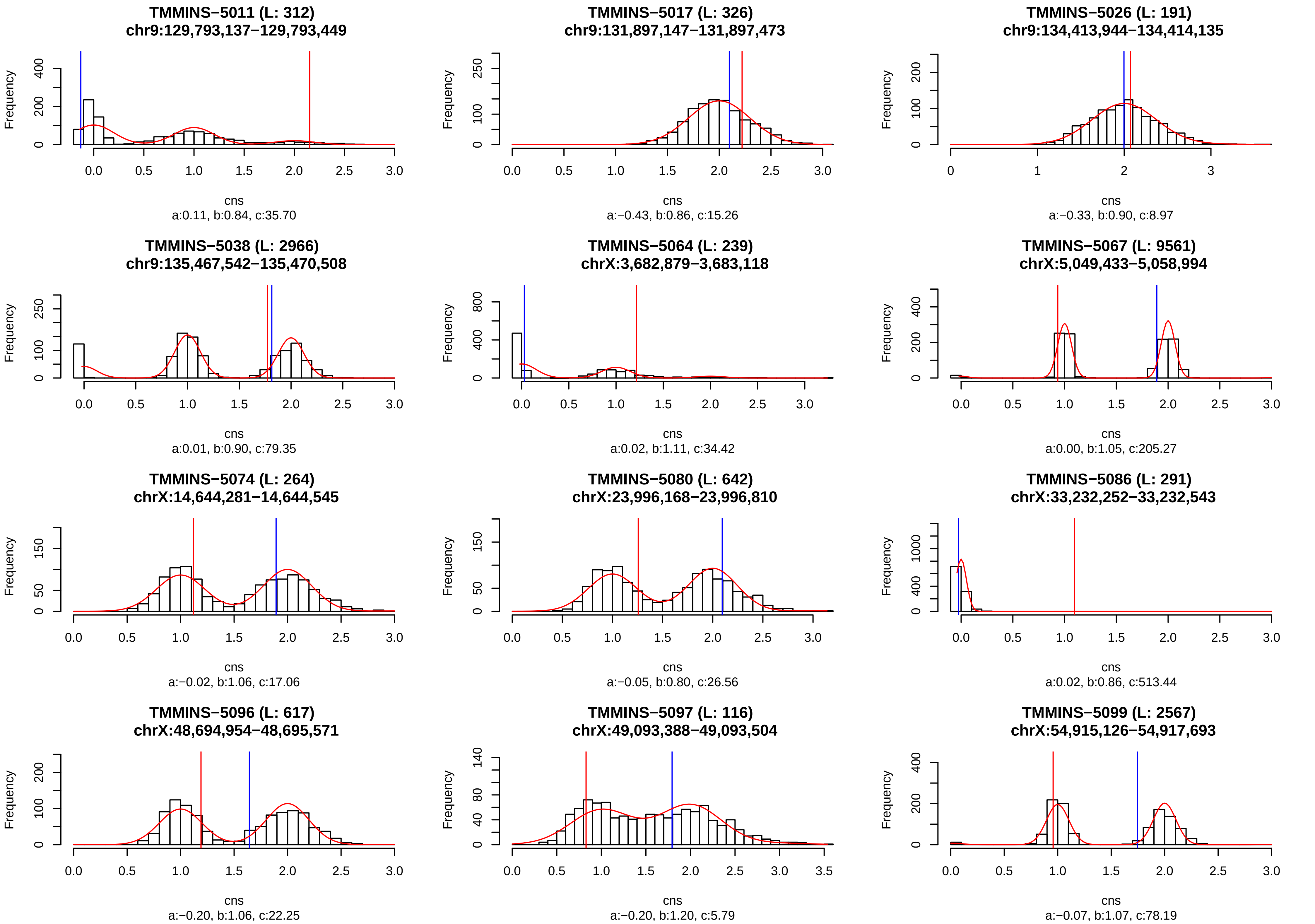
TMMINS−5011 (L: 312)
chr9:129,793,137−129,793,449
Frequency
cns
a:0.11, b:0.84, c:35.70
TMMINS−5017 (L: 326)
chr9:131,897,147−131,897,473
Frequency
cns
a:−0.43, b:0.86, c:15.26
TMMINS−5026 (L: 191)
chr9:134,413,944−134,414,135
Frequency
cns
a:−0.33, b:0.90, c:8.97
TMMINS−5038 (L: 2966)
chr9:135,467,542−135,470,508
Frequency
cns
a:0.01, b:0.90, c:79.35
TMMINS−5064 (L: 239)
chrX:3,682,879−3,683,118
Frequency
cns
a:0.02, b:1.11, c:34.42
TMMINS−5067 (L: 9561)
chrX:5,049,433−5,058,994
Frequency
cns
a:0.00, b:1.05, c:205.27
TMMINS−5074 (L: 264)
chrX:14,644,281−14,644,545
Frequency
cns
a:−0.02, b:1.06, c:17.06
TMMINS−5080 (L: 642)
chrX:23,996,168−23,996,810
Frequency
cns
a:−0.05, b:0.80, c:26.56
TMMINS−5086 (L: 291)
chrX:33,232,252−33,232,543
Frequency
cns
a:0.02, b:0.86, c:513.44
TMMINS−5096 (L: 617)
chrX:48,694,954−48,695,571
Frequency
cns
a:−0.20, b:1.06, c:22.25
TMMINS−5097 (L: 116)
chrX:49,093,388−49,093,504
Frequency
cns
a:−0.20, b:1.20, c:5.79
TMMINS−5099 (L: 2567)
chrX:54,915,126−54,917,693
Frequency
cns
a:−0.07, b:1.07, c:78.19

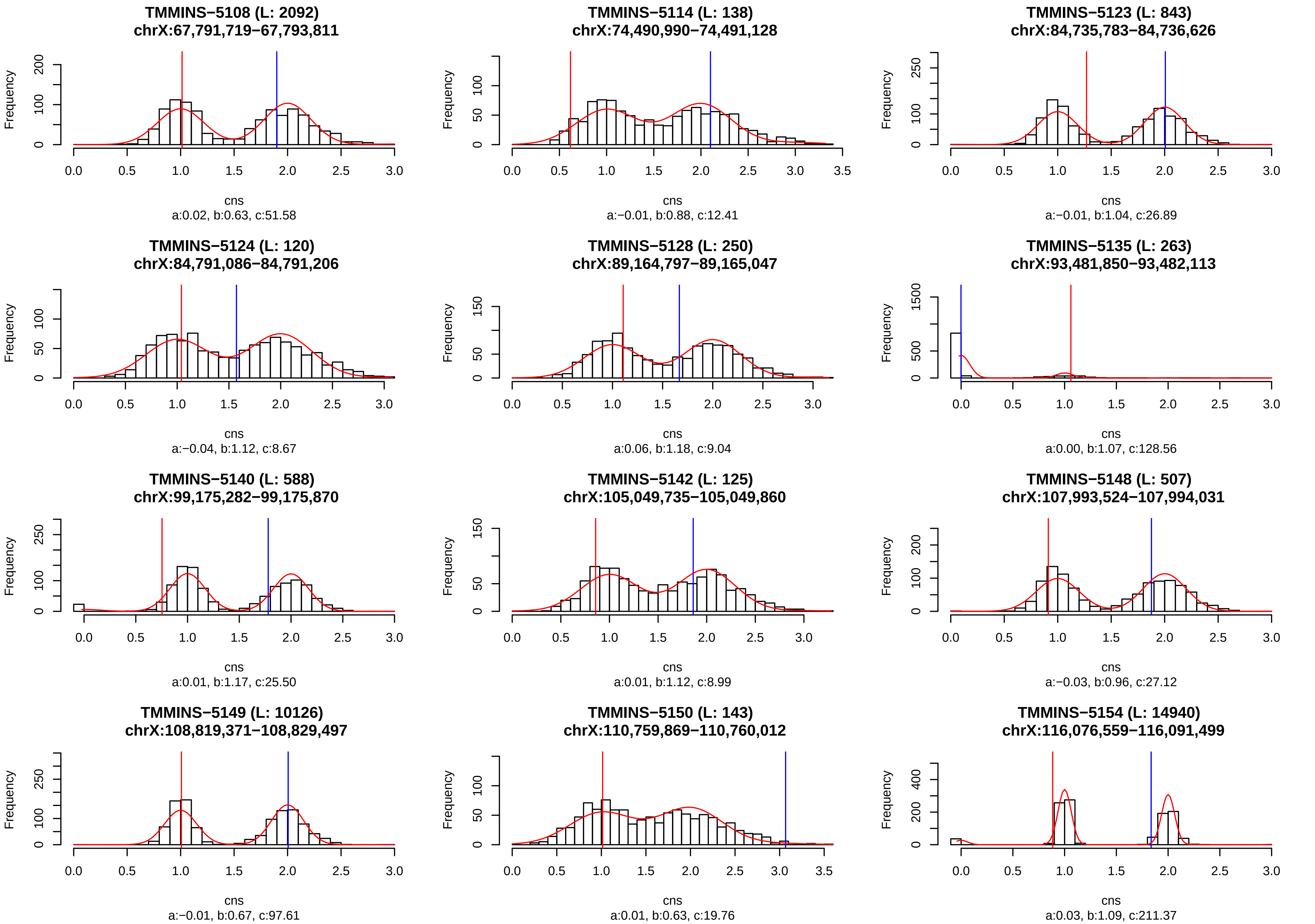
TMMINS−5108 (L: 2092)
chrX:67,791,719−67,793,811
Frequency
cns
a:0.02, b:0.63, c:51.58
TMMINS−5114 (L: 138)
chrX:74,490,990−74,491,128
Frequency
cns
a:−0.01, b:0.88, c:12.41
TMMINS−5123 (L: 843)
chrX:84,735,783−84,736,626
Frequency
cns
a:−0.01, b:1.04, c:26.89
TMMINS−5124 (L: 120)
chrX:84,791,086−84,791,206
Frequency
cns
a:−0.04, b:1.12, c:8.67
TMMINS−5128 (L: 250)
chrX:89,164,797−89,165,047
Frequency
cns
a:0.06, b:1.18, c:9.04
TMMINS−5135 (L: 263)
chrX:93,481,850−93,482,113
Frequency
cns
a:0.00, b:1.07, c:128.56
TMMINS−5140 (L: 588)
chrX:99,175,282−99,175,870
Frequency
cns
a:0.01, b:1.17, c:25.50
TMMINS−5142 (L: 125)
chrX:105,049,735−105,049,860
Frequency
cns
a:0.01, b:1.12, c:8.99
TMMINS−5148 (L: 507)
chrX:107,993,524−107,994,031
Frequency
cns
a:−0.03, b:0.96, c:27.12
TMMINS−5149 (L: 10126)
chrX:108,819,371−108,829,497
Frequency
cns
a:−0.01, b:0.67, c:97.61
TMMINS−5150 (L: 143)
chrX:110,759,869−110,760,012
Frequency
cns
a:0.01, b:0.63, c:19.76
TMMINS−5154 (L: 14940)
chrX:116,076,559−116,091,499
Frequency
cns
a:0.03, b:1.09, c:211.37

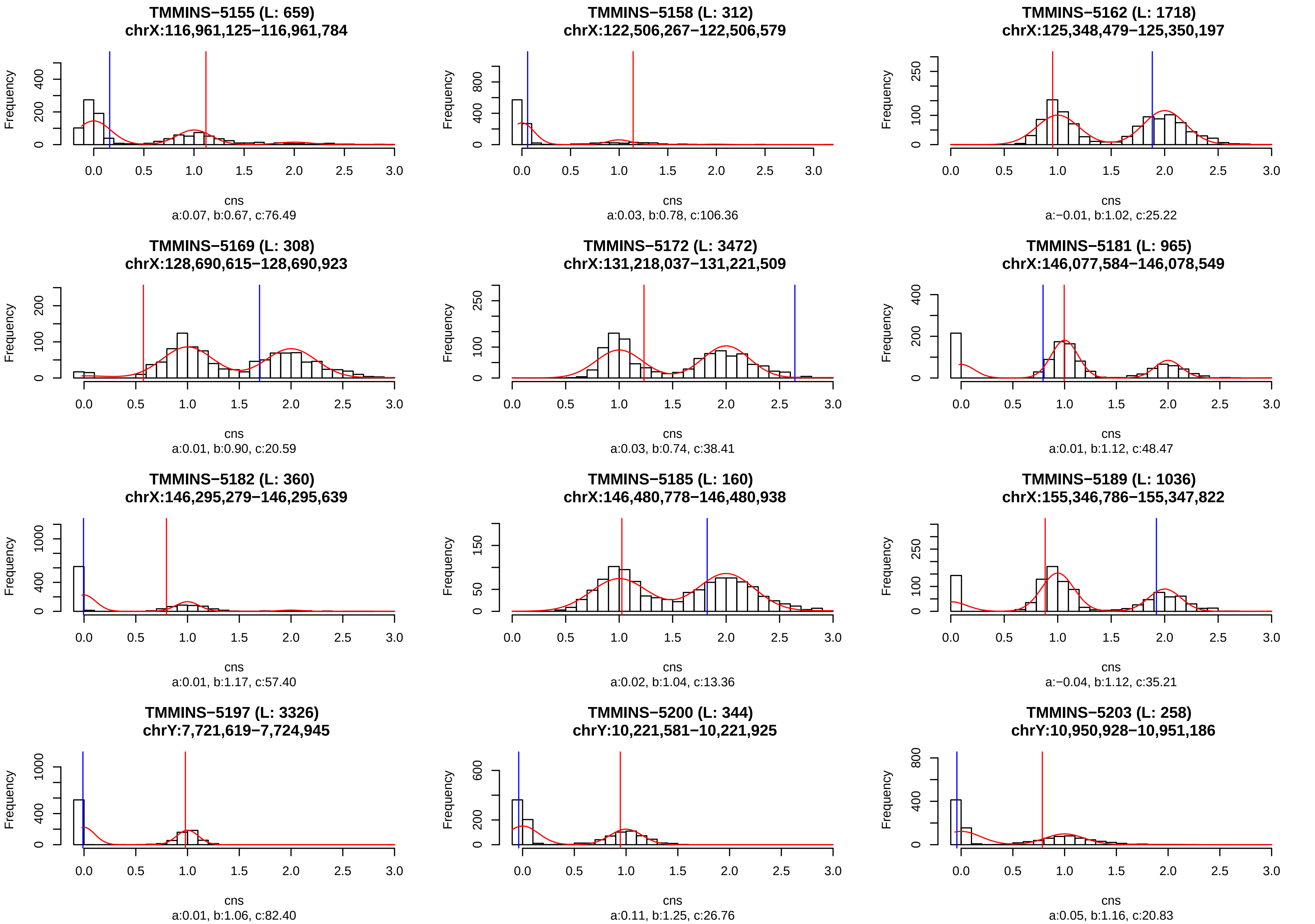
TMMINS−5155 (L: 659)
chrX:116,961,125−116,961,784
Frequency
cns
a:0.07, b:0.67, c:76.49
TMMINS−5158 (L: 312)
chrX:122,506,267−122,506,579
Frequency
cns
a:0.03, b:0.78, c:106.36
TMMINS−5162 (L: 1718)
chrX:125,348,479−125,350,197
Frequency
cns
a:−0.01, b:1.02, c:25.22
TMMINS−5169 (L: 308)
chrX:128,690,615−128,690,923
Frequency
cns
a:0.01, b:0.90, c:20.59
TMMINS−5172 (L: 3472)
chrX:131,218,037−131,221,509
Frequency
cns
a:0.03, b:0.74, c:38.41
TMMINS−5181 (L: 965)
chrX:146,077,584−146,078,549
Frequency
cns
a:0.01, b:1.12, c:48.47
TMMINS−5182 (L: 360)
chrX:146,295,279−146,295,639
Frequency
cns
a:0.01, b:1.17, c:57.40
TMMINS−5185 (L: 160)
chrX:146,480,778−146,480,938
Frequency
cns
a:0.02, b:1.04, c:13.36
TMMINS−5189 (L: 1036)
chrX:155,346,786−155,347,822
Frequency
cns
a:−0.04, b:1.12, c:35.21
TMMINS−5197 (L: 3326)
chrY:7,721,619−7,724,945
Frequency
cns
a:0.01, b:1.06, c:82.40
TMMINS−5200 (L: 344)
chrY:10,221,581−10,221,925
Frequency
cns
a:0.11, b:1.25, c:26.76
TMMINS−5203 (L: 258)
chrY:10,950,928−10,951,186
Frequency
cns
a:0.05, b:1.16, c:20.83

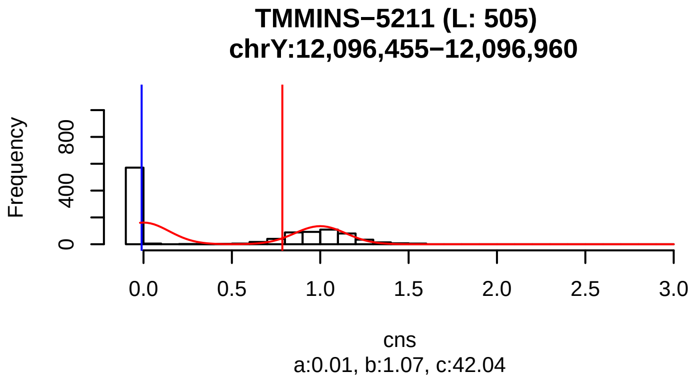
TMMINS−5211 (L: 505)
chrY:12,096,455−12,096,960
Frequency
0
400
800
0.0
0.5
1.0
1.5
2.0
2.5
3.0
cns
a:0.01, b:1.07, c:42.04

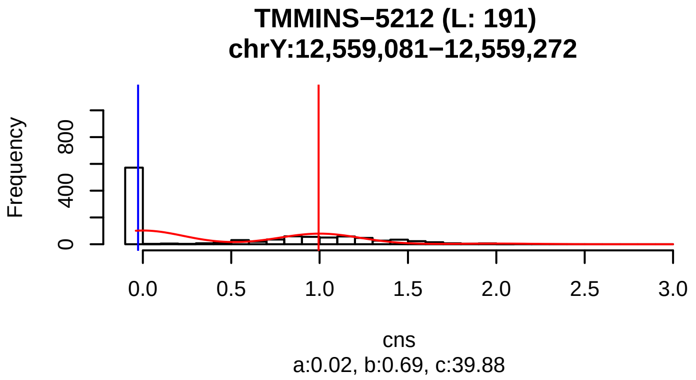
TMMINS−5212 (L: 191)
chrY:12,559,081−12,559,272
Frequency
0
400
800
0.0
0.5
1.0
1.5
2.0
2.5
3.0
cns
a:0.02, b:0.69, c:39.88

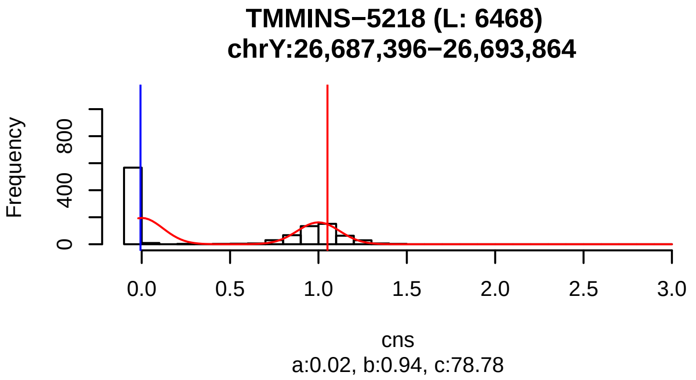
TMMINS−5218 (L: 6468)
chrY:26,687,396−26,693,864
Frequency
0
400
800
0.0
0.5
1.0
1.5
2.0
2.5
3.0
cns
a:0.02, b:0.94, c:78.78
